# The Health and Environmental Impacts, Safety, and Affordability of the EAT-Lancet Planetary Health Diet: A Multidisciplinary Systematic Review and Meta-Analysis

**DOI:** 10.1016/j.advnut.2026.100666

**Published:** 2026-06-01

**Authors:** Ruixin Zhu, Langrun Wang, Huiyu Chen, Jiale Ren, Ran Wang, Yifan Gong, Yiran Guan, Qingbo Wang, Meng Liu, Zhihu Xu, Liwei Zhang, Peng An, Jie Guo, Xueqin He, Miao Xie, Keji Li, J Alfredo Martinez, Anne Raben, Jennie Brand-Miller, Fazheng Ren, Jingjing He

**Affiliations:** 1 Key Laboratory of Precision Nutrition and Food Quality, Department of Nutrition and Health, China Agricultural University, Beijing, China; 2 Department of Nutrition and Food Hygiene, School of Public Health, Capital Medical University, Beijing, China; 3 Institute for Global Health and Development, Peking University, Beijing, China; 4 Climate, Air Quality Research Unit, School of Public Health and Preventive Medicine, Monash University, Melbourne, Victoria, Australia; 5 Laboratory of Biomass and Bioprocessing Engineering, College of Engineering, China Agricultural University, Beijing, China; 6 Department of Data Science and Engineering, College of Information and Electrical Engineering, China Agricultural University, Beijing, China; 7 Department of Nutrition and Food Hygiene, School of Public Health, Peking University, Beijing, China; 8 Centro de Investigacion Biomedica en Red Area de Fisiologia de la Obesidad y la Nutricion (CIBEROBN), Madrid, Spain; 9 Precision Nutrition and Cardiometabolic Health Program, IMDEA-Nutrition Institute (Madrid Institute for Advanced Studies), CEI UAM + CSIC, Madrid, Spain; 10 Department of Medicine and Endocrinology, University of Valladolid, Valladolid, Spain; 11 Department of Nutrition, Exercise and Sports, Faculty of Science, University of Copenhagen, Copenhagen, Denmark; 12 School of Life and Environmental Sciences and Charles Perkins Centre, the University of Sydney, Sydney, New South Wales, Australia

**Keywords:** EAT-Lancet reference diet, food system, nutrition, sustainability, environment, health

## Abstract

In 2019, the EAT-Lancet Commission proposed transition to the planetary health diet, whereas the health and environmental benefits, safety, and affordability of the diet have recently been challenged. This systematic review and meta-analysis aimed to provide a quantitative summary of the EAT-Lancet diet and population health and environmental sustainability; and to review safety (nutrient deficiencies) and affordability of the diet. We searched PubMed, Embase, Web of Science, CINAHL, Cochrane Library, and ClinicalTrials.gov from 1 January 2019 to 19 June 2025 for quantitative studies. Of the eligible studies, epidemiological studies on population health or environmental sustainability were further used for meta-analysis. The risk of bias of the included studies was assessed using the Newcastle–Ottawa Scale tool. A total of 227 studies were included in the systematic review and 79 of them were used for the meta-analysis. Most prospective cohort studies and half the cross-sectional studies were of high quality. The EAT-Lancet diet was associated with lower risks of all-cause mortality [pooled hazard ratio = 0.80, 95% confidence interval (CI): 0.76, 0.85; *n* = 17, 1,063,289 participants], cardiovascular disease (0.83, 95% CI: 0.74, 0.94; *n* = 10, 1,027,822 participants), and type 2 diabetes, but not risks of all cancers or dementia. The EAT-Lancet diet was associated with a reduction of 1.55 (0.22, 2.89) kg CO_2_ eq/d/person of greenhouse gas emission (*n* = 12, 882,673 participants), whereas no strong evidence supported the benefits to water or land use. On a global average, the EAT-Lancet diet would induce deficiencies in vitamin B_12_, calcium, iron, and zinc in adults, especially in pregnant women. Globally, 1.6–3.0 billion people were not able to afford the diet. In conclusion, adoption of the EAT-Lancet diet may modestly reduce global chronic disease and environmental burden, whereas nutrient deficiencies and economic inequality across regions should be addressed before dietary transition.

**Trial registration number:**

This study was registered at PROSPERO as CRD42024540867 (URL of registration: https://www.crd.york.ac.uk/PROSPERO/view/CRD42024540867).

## Introduction

**Statement of Significance**

The health and environmental benefits, nutrition, and affordability of the EAT-Lancet planetary health diet have recently been challenged. To our knowledge, to date, no systematic reviews and meta-analyses have yet systematically and quantitatively summarized the impacts of the EAT-Lancet planetary health diet on multiple health outcomes and the environment. Furthermore, no systematic reviews have examined the safety and affordability of the diet. To bridge the knowledge gap, we conducted a multidisciplinary and comprehensive meta-analysis and systematic review.

Food systems and dietary patterns play an important role in population health and environmental sustainability [1]. Current global food systems and dietary patterns, however, underpin unsustainable food production and unhealthy food consumption, posing global risks to both humans and the planet [1,2]. Consumption of unhealthy diets is associated with noncommunicable diseases and results in >10 million premature deaths each year [1,3]. Unsustainable food systems are one of the major causes of climate change [1,4,5], which is among leading dimensions that have transgressed planetary boundaries [6,7]. Earth’s natural resources and life support systems may be inadequate to sustain the growing global population in the near future. To achieve the United Nations Sustainable Development Goals and the Paris Agreement [1] by 2050 as planned, a “Great Food Transformation” and global dietary shifts are urgent.

In 2019, the EAT-Lancet planetary health diet (also known as the EAT-Lancet diet or the planetary health diet), a sustainable and healthy plant-based diet, was proposed by the EAT-Lancet Commission as a reference diet for global dietary shifts [1]. The EAT-Lancet planetary health diet is designed to improve population health and reduce the environmental effect of food systems by increasing intakes of healthy and environmentally friendly foods such as nonstarchy vegetables, fruits, legumes, and wholegrains as well as cutting down intakes of red meat, added sugar, and highly processed foods [1]. Global food system transformation and the EAT-Lancet planetary health diet have recently attracted a great deal of attention from the governments, policy makers, and experts [8]. The associations of the EAT-Lancet planetary health diet or sustainable food production with population and environmental impacts have been explored by many studies in recent years [8] and a recent meta-analysis showed that adherence to the EAT-Lancet planetary health diet was related to reduced odds of cancer, diabetes, cardiovascular disease (CVD), and mortality [9]. However, the relationship between the EAT-Lancet planetary health diet and other diseases such as liver diseases, mental disorders, and reproductive health has not been summarized and some studies have argued that the EAT-Lancet dietary pattern may not as good as expected [10,11]. In addition, as the EAT-Lancet planetary health diet is a plant-predominant diet with limited recommended intake of animal-based foods, concerns have been raised about the safety of the diet (e.g., deficiencies in nutrients that are generally found in animal-based foods, such as vitamin B_12_) [12]. Furthermore, affordability is one of the determinants for adoption of the EAT-Lancet planetary health diet globally, with some recent studies expressing concerns [13,14].

To date, no systematic reviews and meta-analyses have yet systematically and quantitatively summarized the impacts of the EAT-Lancet planetary health diet on multiple health outcomes and the environment. Furthermore, no systematic reviews have examined the safety and affordability of the diet. To bridge the knowledge gap, we conducted a multidisciplinary and comprehensive meta-analysis and systematic review to examine the associations of the EAT-Lancet planetary health diet with health and environmental impacts. In addition, we also aimed to systematically review safety (nutrient deficiencies) and affordability of the diet. Furthermore, the gap between current global diets and the EAT-Lancet planetary health diet was examined.

## Methods

### Study design

We conducted a systematic review and meta-analysis on the associations of EAT-Lancet planetary health diet with outcomes of interest in accordance with the Cochrane Handbook for Systematic Reviews of Interventions [15], the Meta-analysis Of Observational Studies in Epidemiology (MOOSE) [16], the PRISMA 2020 statement [17] and its explanation and elaboration [18], and the Population, Intervention, Comparison, and Outcome (PICO) framework. The protocol of the current study was registered in the PROSPERO registry (CRD42024540867).

### Outcomes

Primary outcomes were population health outcomes and environmental outcomes. In this study, the major health outcomes included mortality, cancers, CVD, cerebrovascular diseases, glucose-related outcomes, blood pressure–related outcomes, blood lipid–related outcomes, body weight–related outcomes, metabolic syndrome/cardiovascular–kidney–metabolic syndrome, liver diseases, mental disorders, aging, female and male reproductive health, physical function and performance, inflammatory bowel diseases, respiratory system diseases, and inflammatory factors. The major environmental outcomes included ecological footprint, natural resource consumption, biodiversity, environmental pollution, greenhouse gas emissions (GHGEs) and nitrogen emissions, land use, and water use. Secondary outcomes included safety (nutrient intake and deficiencies), affordability (cost of the EAT-Lancet planetary health diet), and the gap between current global diets and the EAT-Lancet planetary health diet.

### Search strategy and selection criteria

We conducted a preliminary search on PubMed to get an overview of studies on the EAT-Lancet planetary health diet. As the EAT-Lancet Commission’ report was first published in 2019 [1], the publication date of potential studies was limited from 1 January 2019 to 17 April 2024. In the formal search, we searched the following electronic databases from 1 January 2019 to 22 April 2024 to identify studies: PubMed, Ovid Embase, Web of Science, EBSCOhost CINAHL, Cochrane Library, ClinicalTrials.gov, and references of screened articles and previous systematic reviews and meta-analyses. To ensure a broad search, we based our search strategy on keywords of the exposure of interest, that is, the EAT-Lancet diet only. The EAT-Lancet diet-related keywords searched for were “EAT Lancet,” “EAT-Lancet,” “planetary health diet∗,” “reference diet∗,” “planetary diet∗,” “lancet diet∗,” etc. The search was updated on 19 June 2025 in case new papers came out. Detailed search strategies are provided in Supplemental Table 1 in Appendix 1 in Supplemental Materials I**.**

The selection criteria for the current systematic review were structured according to the PICO and its variant (i.e., population, exposure, and outcome) framework. Studies were eligible for inclusion if they: *1*) included the EAT-Lancet planetary health diet as an intervention group/exposure/independent variable; *2*) included the primary or secondary outcomes (i.e., population health, environmental sustainability, nutrient intakes and deficiencies, affordability, or gaps between current dietary patterns and the EAT-Lancet planetary health diet). Detailed inclusion criteria for each outcome of interest are described in Supplemental Table 2 in Appendix 2 in Supplemental Materials Ⅰ; *3*) were quantitative studies (e.g., clinical trials, cohort studies, cross-sectional studies, case–control studies, and modeling studies); *4*) were full length original articles/investigations, short/brief communications, letters/research letters, perspective, viewpoints, comments, reviews, or editorials as long as they presented original data; *5*) were written in English language. If the same database had been published in >1 publication, we included the one with more complete findings or longer follow-up or the greatest number of participants. There were no restrictions on population characteristics. Studies were excluded if they: *1*) were meta-analyses, protocols, methods papers, or reviews that did not provide original data; *2*) did not report any outcomes of interest; *3*) were animal studies or cell studies or case report or qualitative studies; *4*) were conference/meeting papers/abstracts or theses/dissertations, unpublished studies or gray literature, because the quality of those studies was not assessed by reviewers and editors; *5*) were not written in English language. In addition, if an outcome was reported in only a single study, that study was not included in the meta-analysis. After the removal of duplicates by a researcher (LW) using Endnote X9, 2 researchers (LW and HC) independently completed the electronic searches and reviewed titles and abstracts for eligible papers. To avoid missing any publication, we also checked the reference lists of extracted papers and recent reviews. The current meta-analysis included clinical trials, cohort studies, cross-sectional studies, and case–control studies with available data.

### Data extraction

Data extraction was conducted by 2 researchers (LW and HC) independently. Any disagreements were resolved in consultation with the principal investigator (JH). We extracted first author’s family name, publication year, country, baseline assessment period (if applicable), follow-up duration (if applicable), study type, sample size (if applicable), study participants’ characteristics (if applicable), dietary exposure or intervention/control group, dietary assessment methods, outcomes, adjusted confounding factors (if applicable), and main findings. Two reviewers (RZ and YGong) independently extracted effect size of outcomes [i.e., hazard ratio (HR) or risk ratio (RR) or odd ratio (OR) or *β* or mean values or median values along with 95% confidence interval (CI) or interquartile range (IQR) or SD or SE] from the studies included in meta-analysis. The Origin 2020 digitizer (OriginLab Corporation) was used to extract the outcome data from graphs.

### Risk of bias and certainty of evidence assessment

We assessed the risk of bias in the studies included in the meta-analysis, as there were no standard tools designed for some study types (e.g., modeling studies and comparative studies) for risk assessment. Two researchers (RZ and LW) independently assessed the risk of bias using the Newcastle–Ottawa Scale (NOS)-cohort studies tool [19] for prospective cohort studies, the NOS-case–control studies tool for case–control studies, and the adapted NOS tool for cross-sectional studies. The NOS tools consist of 3 domains: selection (max. 4 points for cohort and case–control studies and 3 points for cross-sectional studies), comparability (max. 2 points for all study types), and exposure or outcome (max. 3 points for cohort and case–control studies and 2 points for cross-sectional studies) [19]. Up to 9 points can be awarded to each cohort or case–control study and ≤7 points can be awarded to each cross-sectional study. An NOS score of ≥7 or ≤6 points was considered high and low quality, respectively.

The certainty of evidence on population health outcomes was assessed independently by two reviewers (LW and HC). We used the Grading of Recommendation, Assessment, Development, and Evaluation (GRADE) [20] with GRADEpro Guideline Development Tool. The certainty of the evidence was graded into 4 levels: high, moderate, low, or very low. Observational studies started at a rating of “low” certainty of evidence. Five downgraded and 3 upgraded criteria were then applied according to the GRADE handbook and Chiavaroli et al. [21]. The criteria for downgrades included: *1*) risk of bias (the majority of studies was considered to be at high risk of bias assessed by NOS); *2*) inconsistency (large unexplained heterogeneity, *I*^2^ ≥ 50%, *P* < 0.10); *3*) indirectness some limitations related to the participants, interventions, or outcomes that may affect generalizability of the result); *4*) imprecision {95% CIs overlap no effect (i.e., CI includes RR of 1.0) = 1 unless large populations (simple size ≥ 2000) and very low incidence; and the same logic for continuous outcomes [i.e., effect estimates overlapped minimally important differences (MIDs)] [21] for benefit or harm}, and *5*) publication bias (which we defined small study effects as results from a trim and fill analysis which showed imputed trials resulted in a different conclusion compared with the original data. We conducted trim and fill analyses if we identified evidence of publication bias by inspection of funnel plots and significance by Egger’s test at *P* < 0.05). The criteria for upgrades included: *1*) a large magnitude of effect (i.e., point estimate <0.5 or >2 in the absence of plausible confounding factors for dichotomous outcomes; or the effect size excluded 5 × MID for continuous outcomes); *2*) a dose–response gradient; and *3*) attenuation by plausible confounding factors. Any disagreements were resolved in consultation with the principal investigator (JH).

### Data synthesis and statistical analysis

We conducted a meta-analysis of observational studies (e.g., prospective cohort studies, cross-sectional studies, case–control studies, etc.) on population health and environmental sustainability (primary outcomes). If an outcome was reported in only a single study, that study was not included in the meta-analysis. As the effects of the EAT-Lancet planetary health diet were likely to be variable according to participants’ baseline characteristics and assessment methods of diet adherence, random effect models were used to pool study effect estimates for each primary outcome of interest [22]. The restricted maximum likelihood method was used to calculate between-study (heterogeneity) variance estimator, because the latter may be negatively biased in scenarios with small studies and in scenarios with a rare dichotomous outcome [23]. The Hartung–Knapp modification was used to calculate the CI (uncertainty) for the summary effect, as this method may result in fewer type 1 errors when study population sizes differ and study number is small [24,25]. For population health and environmental outcomes, results were expressed as HR or OR or *β* with 95% CI or mean with SD. Median with IQR were converted to mean with SD using an online Excel spreadsheet calculator [26]. Regarding statistical heterogeneity, Cochran’s Q statistic (significance was set at *P* < 0.10) and τ^2^ statistic, a between-study (heterogeneity) variance estimator, was used to identify inconsistency across the findings of the studies. *I*^2^ statistic was used to measure the proportion of the total variance because of between-study heterogeneity. We considered *I*^2^ thresholds of 0%∼25% as a small proportion, 25%∼75% as a moderate proportion, and 75%∼100% as a high proportion of the total variance because of between-study heterogeneity.

Sensitivity analyses were conducted to examine the robustness of the primary outcomes. We excluded one study at a time to check whether the observed association was driven by a single study. Prespecified subgroup analyses of sex and age were not conducted as very few studies reported the results of age or sex subgroup analyses. When 3 or more trial comparisons were available, small study effects (e.g., selection or publication bias) were investigated by visual inspection of Begg’s funnel plots and statistical asymmetry evaluated by Egger’s regression asymmetry test. All hypothesis tests were 2 sided. A *P* value < 0.05 was considered significant for all tests, except for Cochran’s Q test.

Global maps for primary and secondary outcomes were created. In the global maps, for health and environmental outcomes, the color intensity for a country/region represents the cumulative count of reported major beneficial health or environmental outcomes (predefined in the Methods section), with each outcome category per study counted once. Darker shades on the map indicate a greater number of such beneficial outcomes. Areas where null or harmful associations were also reported are overlaid with a textured pattern or a diagonal stripe pattern, the latter emphasized with thick diagonal stripes if null/harmful outcomes outnumbered beneficial ones. The nutrient deficiency risks are presented in a 2-panel figure, where the first panel uses color intensity to indicate the cumulative count of reported instances of nutrients potentially reaching higher intakes under the EAT-Lancet planetary health diet (compared with low adherence or national guidelines), with each nutrient per study counted once, and the second panel similarly represents nutrients at risk of lower intakes, with the same counting rule. For affordability, the color intensity corresponds to the number of studies deeming the diet “affordable” in a country/region; in cases where studies also report the diet as “unaffordable,” a diagonal stripe pattern is overlaid, and the actual counts of studies in both categories (“affordable” and “unaffordable”) are annotated on the map. The gaps between the diets are shown in another 2-panel figure, with one panel indicating the cumulative count of reported instances of food groups whose current average intake exceeds EAT-Lancet recommendations, with each food group per study counted once, and the other highlighting those with insufficient intake levels. Finally, results from studies that pooled multiple countries/regions without providing disaggregated data were summarized in a separate panel in the bottom right corner of each map.

Data were analyzed using Stata 18 (StataCorp) with the “meta set,” “meta esize,” “meta summarize,” “meta forestplot,” “meta funnelplot,” and “metabias” commands.

## Results

### Study characteristics

**FIGURE 1:**
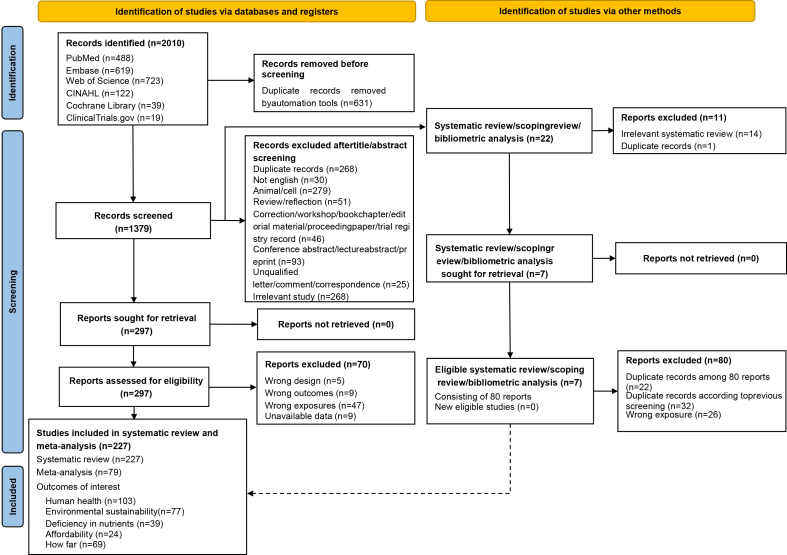
PRISMA flowchart of literature search.

The flow of literature search and selection process is shown in Figure 1. Of the 2010 reports identified, 1379 were screened and 297 were assessed for eligibility. In total, 70 studies were excluded on the basis of their design, outcomes, exposures, or unavailable data (5 of them had wrong study design, 9 of them reported wrong outcomes, 47 of them reported wrong exposures, and 9 of them did not have available data). Data from 227 studies [[11], [12], [13],^15^,[27], [28], [29], [30], [31], [32], [33], [34], [35], [36], [37], [38], [39], [40], [41], [42], [43], [44], [45], [46], [47], [48], [49], [50], [51], [52], [53], [54], [55], [56], [57], [58], [59], [60], [61], [62], [63], [64], [65], [66], [67], [68], [69], [70], [71], [72], [73], [74], [75], [76], [77], [78], [79], [80], [81], [82], [83], [84], [85], [86], [87], [88], [89], [90], [91], [92], [93], [94], [95], [96], [97], [98], [99], [100], [101], [102], [103], [104], [105], [106], [107], [108], [109], [110], [111], [112], [113], [114], [115], [116], [117], [118], [119], [120], [121], [122], [123], [124], [125], [126], [127], [128], [129], [130], [131], [132], [133], [134], [135], [136], [137], [138], [139], [140], [141], [142], [143], [144], [145], [146], [147], [148], [149], [150], [151], [152], [153], [154], [155], [156], [157], [158], [159], [160], [161], [162], [163], [164], [165], [166], [167], [168], [169], [170], [171], [172], [173], [174], [175], [176], [177], [178], [179], [180], [181], [182], [183], [184], [185], [186], [187], [188], [189], [190], [191], [192], [193], [194], [195], [196], [197], [198], [199], [200], [201], [202], [203], [204], [205], [206], [207], [208], [209], [210], [211], [212], [213], [214], [215], [216], [217], [218], [219], [220], [221], [222], [223], [224], [225], [226], [227], [228], [229], [230], [231], [232], [233], [234], [235], [236], [237], [238], [239], [240], [241], [242]] were included in the current systematic review, with 103 of them reporting population health outcomes, 77 of them reporting environmental outcomes, 39 of them focusing on nutrient intakes and deficiencies, 24 of them investigating affordability, and 69 of them examining the gaps between current global diets and the EAT-Lancet planetary health diet. Among these studies included in the systematic review, 79 observational studies [11,15,41,42,[46], [47], [48], [49],^51^,^52^,^62^,^63^,^69^,^76^,^80^,^90^,^91^,^96^,^101^,^102^,^110^,^111^,^115^,[117], [118], [119], [120],^122^,^125^,^127^,^128^,[132], [133], [134], [135],^137^,^143^,^147^,^148^,^151^,^154^,^155^,^159^,^161^,^163^,^171^,^173^,^177^,^178^,^180^,^181^,^184^,^188^,^191^,^194^,^195^,^199^,^203^,^205^,^207^,^209^,^215^,^222^,[225], [226], [227], [228], [229],[234], [235], [236], [237], [238], [239], [240],[243], [244], [245], [246]] were further used for meta-analysis. It is worth noting that no clinical trials met the inclusion criteria. Supplemental Tables 3–7 in Appendix 3 in Supplemental Materials Ⅰ show the detailed characteristics of all studies included in the meta-analysis and systematic review. Key excluded studies and reasons are listed in Supplemental Table 8 in Appendix 4 in Supplemental Materials Ⅰ.

### Risk of bias and certainty of evidence

Supplemental Tables 9 and 10 in Appendix 5 in Supplemental Materials Ⅰ show the risk of bias of studies included in this meta-analysis. According to the NOS criteria, most prospective cohort studies with population health and environmental outcomes were of high quality, whereas only half of the cross-sectional studies were of high quality. Specifically, 52 (96.3%) and 2 (3.7%) prospective cohort studies were judged as high and low quality, respectively; 10 (47.6%) and 11 (52.4%) cross-sectional were of high and low quality, respectively. The certainty of evidence of the studies included in this meta-analysis is shown in Supplemental Table 11 in Appendix 6 in Supplemental Materials Ⅰ. Most pooled results were of low or very low certainty according to GRADE.

### The EAT-Lancet planetary health diet and population health

Studies included in the present systematic review and meta-analysis reported the associations of the EAT-Lancet planetary health diet with all-cause and cause-specific mortality, cancers and subtypes, CVD, and cerebrovascular diseases, diabetes, obesity, metabolic dysfunction–associated steatotic liver disease, mental disorders, male reproductive health, and aging (Table 1 and Appendix 7 in Supplemental Materials Ⅰ), whereas no studies examined the associations of EAT-Lancet planetary health diet with female reproductive health or offspring health.

**TABLE 1:** Random effects meta-analysis for the association between adherence to the EAT-Lancet planetary health diet and population health

Outcome	Study design	Type of effect size	Forest plot	No. of studies	No. of comparisons	No. of participants	Effect size	LCI	UCI	Effect size (95% CI)	*P*	*P*_Heterogeneity	τ^2^	*I*^2^ (%)	Funnel plot	*P*_Egger	Leave-one-out plot	GRADE
1. Mortality																		
All-cause mortality	Prospective cohort	HR	S1-1	17	18	1,063,289	0.80	0.76	0.85	0.80 (0.76, 0.85)	<0.001	<0.001	0.007	75.7	S2-1	0.47	S3-1	Very Low
Cancer mortality				7	8	419,568	0.90	0.86	0.95	0.90 (0.86, 0.95)	0.001	0.270	<0.001	0.0	S2-2	0.211	S3-2	Low
Cardiovascular mortality				7	8	449,950	0.83	0.76	0.89	0.83 (0.76, 0.89)	0.001	0.135	0.003	38.3	S2-3	0.384	S3-3	Low
Respiratory disease mortality				2	2	269,661	0.66	0.04	9.70	0.66 (0.04, 9.70)	0.296	<0.001	0.087	97.2	—	—	—	—
2. Cancer																		
All cancers	Prospective cohort	HR	S1-2	2	2	536,218	0.92	0.70	1.21	0.92 (0.70, 1.21)	0.155	0.316	<0.001	0.7	—	—	—	—
Lung cancer				3	3	336,011	0.69	0.56	0.84	0.69 (0.56, 0.84)	0.016	0.645	<0.001	0.0	S2-4	0.646	S3-4	Low
Small-cell lung cancer				2	2	273,629	0.57	0.47	0.71	0.57 (0.47, 0.70)	0.019	0.948	<0.001	0.0	—	—	—	—
Breast cancer				3	3	547,004	0.96	0.81	1.14	0.96 (0.81, 1.14)	0.421	0.460	<0.001	0.0	S2-5	0.584	S3-5	Low
Colorectal cancer				4	4	812,074	0.87	0.81	0.93	0.87 (0.81, 0.93)	0.008	0.851	<0.001	0.0	S2-6	0.852	S3-6	Low
Prostate cancer				2	2	536,218	1.15	0.05	28.12	1.15 (0.05, 28.12)	0.678	0.022	0.105	80.8	—	—	—	—
3. Cardiovascular disease																		
Cardiovascular disease/events	Prospective cohort	HR	S1-3	10	11	1,027,822	0.83	0.74	0.94	0.83 (0.74, 0.94)	0.007	0.002	0.012	75.2	S2-7	0.608	S3-7	Very Low
Coronary artery disease				4	4	315,373	0.83	0.77	0.89	0.83 (0.77, 0.89)	0.003	0.717	<0.001	0.0	S2-8	0.756	S3-8	Low
Ischemic heart disease				2	2	160,234	0.74	0.55	1.01	0.74 (0.55, 1.01)	0.051	0.498	<0.001	0.0				—
Myocardial infarction				3	3	606,957	0.54	0.03	8.57	0.54 (0.03, 8.57)	0.438	0.000	1.154	99.4	S2-9	0.487	S3-9	Very Low
Atrial fibrillation				2	2	138,878	0.88	0.59	1.31	0.88 (0.59, 1.31)	0.156	0.439	<0.001	0.0				—
Heart failure				2	3	137,425	0.74	0.58	0.95	0.74 (0.58, 0.95)	0.034	0.441	<0.001	0.7	S2-10	0.082	S3-10	Low
4. Cerebrovascular disease																		
Stroke	Prospective cohort	HR	S1-4	8	8	1,051,321	0.90	0.84	0.97	0.90 (0.84, 0.97)	0.014	0.344	0.001	12.7	S2-11	0.505	S3-11	Low
Hemorrhagic stroke				3	4	619,364	0.86	0.38	1.93	0.86 (0.38, 1.93)	0.595	0.065	0.102	59.4	S2-12	0.117	S3-12	Low
Ischemic stroke				4	4	757,966	0.93	0.80	1.07	0.93 (0.80, 1.07)	0.194	0.456	0.001	9.5	S2-13	0.447	S3-13	Low
5. Glucose-related outcomes																		
Type 2 diabetes	Prospective cohort	HR	S1-5	8	8	551,687	0.75	0.57	0.98	0.75 (0.57, 0.98)	0.040	<0.001	0.094	96.5	S2-14	0.03	S3-14	Very Low
Hyperglycemia	Cross-sectional	OR	S1-6	2	2	7102	0.90	0.73	1.11	0.90 (0.73, 1.11)	0.315	0.797	<0.001	0.0	—	—	—	—
Fasting plasma glucose	Cross-sectional	*β*	S1-7	2	2	8767	−0.19	−2.80	2.43	−0.19 (−2.80, 2.43)	0.890	0.064	0.064	71.0	—	—	—	—
HOMA-IR				2	2	14,749	0.00	−0.02	0.03	0.00 (−0.02, 0.03)	0.936	0.460	<0.001	0.0	—	—	—	—
6. Blood pressure–related outcomes																		
High blood pressure	Cross-sectional	OR	S1-8	2	2	7102	0.98	0.16	6.23	0.98 (0.16, 6.23)	0.984	0.044	0.033	75.4	—	—	—	—
Systolic blood pressure	Prospective cohort	*β*	S1-9a	2	2	742	0.20	−10.34	10.74	0.20 (−10.34, 10.74)	0.971	<0.001	1.333	96.7	—	—	—	—
Diastolic blood pressure				2	2	744	0.44	−6.55	7.43	0.44 (−6.55, 7.43)	0.901	<0.001	0.603	99.7	—	—	—	—
Systolic blood pressure	Cross-sectional		S1-9b	3	3	22,681	−0.05	−0.18	0.07	−0.05 (−0.18, 0.07)	0.410	0.100	0.001	56.6	S2-15	0.104	S3-15	Very Low
Diastolic blood pressure				3	3	22,681	−0.02	−0.07	0.03	−0.02 (−0.07, 0.03)	0.428	0.100	<0.001	0.0	S2-16	0.469	S3-16	Very Low
7. Blood lipid–related outcomes																		
Triglycerides	Prospective cohort	*β*	S1-10a	2	2	992	1.00	0.90	1.10	1.00 (0.90, 1.10)	0.005	0.699	<0.001	0.0	—	—	—	—
LDL-C				2	2	987	0.52	−5.78	6.81	0.52 (−5.78, 6.81)	0.487	<0.001	0.490	100.0	—	—	—	—
HDL-C				3	3	1236	0.55	−0.50	1.60	0.55 (−0.50, 1.60)	0.152	<0.001	0.173	96.3	S2-17	0.266	S3-17	Very Low
Triglycerides	Cross-sectional		S1-10b	4	4	23,545	−0.14	−0.64	0.36	−0.14 (−0.64, 0.36)	0.439	0.077	0.036	51.3	S2-18	0.559	S3-18	Very Low
Total cholesterol				2	2	14,553	−0.42	−4.84	4.00	−0.42 (−4.84, 4.00)	0.442	0.001	0.220	90.2				—
LDL-C				3	3	15,410	−0.16	−0.66	0.34	−0.16 (−0.66, 0.34)	0.304	<0.001	0.035	97.9	S2-19	0.04	S3-19	Very Low
HDL-C				4	4	23,544	0.07	−0.13	0.27	0.07 (−0.13, 0.27)	0.340	<0.001	0.015	99.8	S2-20	0.288	S3-20	Very Low
8. Body weight–related outcome																		
Obesity	Prospective cohort	HR	S1-11	2	2	237,573	0.87	0.73	1.02	0.87 (0.73, 1.02)	0.057	0.492	<0.001	0.0	—	—	—	—
Overweight/obesity	Cross-sectional	OR	S1-12	4	6	61,588	0.87	0.76	0.99	0.87 (0.76, 0.99)	0.033	<0.001	0.013	81.2	S2-21	0.049	S3-21	Very Low
	Prospective cohort	PR	S1-13	1	2	3564	0.91	0.88	0.94	0.91 (0.88, 0.94)	<0.001	0.945	<0.001	0.0	—	—	—	—
Body weight	Prospective cohort	*β*	S1-14a	2	2	1004	0.31	−8.27	8.89	0.31 (−8.27, 8.89)	0.726	<0.001	0.904	99.2	—	—	—	—
BMI	Cross-sectional		S1-14b	4	4	22,794	−0.06	−0.26	0.14	−0.06 (−0.26, 0.14)	0.581	<0.001	0.011	99.4	S2-22	0.808	S3-22	Very Low
z-score BMI				2	2	1153	−0.05	−0.85	0.75	−0.05 (−0.85, 0.75)	0.903	0.132	0.005	56.0	—	—	—	—
Waist circumference				5	5	30,937	−0.10	−0.27	0.06	−0.10 (−0.27, 0.06)	0.227	<0.001	0.016	99.0	S2-23	0.571	S3-23	Very Low
9. Liver disease																		
MASLD	Prospective cohort	HR	S1-15	2	4	297,230	0.80	0.71	0.92	0.80 (0.71, 0.92)	0.013	0.285	0.003	31.7	S2-24	0.340	S3-24	Low
10. Respiratory system disease																		
Asthma	Cross-sectional	OR	S1-16	2	2	33,048	0.86	0.83	0.90	0.86 (0.83, 0.90)	<0.001	0.963	<0.001	0.0	—	—	—	—
11. Mental disorders																		
Dementia	Prospective cohort	HR	S1-17	2	2	216,791	0.96	0.85	1.10	0.96 (0.85, 1.10)	0.582	0.644	<0.001	0.0	—	—	—	—
Depression	Cross-sectional	OR	S1-18	5	5	90,199	0.73	0.65	0.81	0.73 (0.65, 0.81)	<0.001	0.634	<0.001	0.0	S2-25	0.754	S3-25	Low
Anxiety				2	2	6573	0.87	0.36	2.08	0.87 (0.36, 2.08)	0.748	0.490	<0.001	0.0	—	—	—	—
Global cognition	Prospective cohort	*β*	S1-19	2	2	3723	0.02	−0.02	0.05	0.02 (−0.02, 0.05)	0.905	0.489	<0.001	0.3	—	—	—	—
Executive function				2	2	12,367	0.01	−0.11	0.13	0.01 (−0.11, 0.13)	0.131	0.006	<0.001	86.9	—	—	—	—

Abbreviations: CI, confidence interval; HR, hazard ratio; MASLD, metabolic dysfunction-associated steatotic liver disease; OR, odds ratio; PR, prevalence ratio; RR, risk ratio.

With regard to the findings from meta-analysis, prospective cohort studies demonstrated that higher adherence to the EAT-Lancet planetary health diet was associated with a 20% reduction in the risk of all-cause mortality (pooled HR = 0.80, 95% CI: 0.76, 0.85, *P* < 0.001, *n* = 17, 1,063,289 participants, *I*^2^ = 75.7%, τ^2^ = 0.007, very low certainty) and a decrease in risks of mortality from cancer and CVD (Table 1 and Appendix 7 in Supplemental Materials Ⅰ). Moreover, a modeling study included in the current systematic review estimated that 1.8 million premature deaths could be avoided by adoption of the EAT-Lancet planetary health diet (Supplemental Table 3 in Appendix 3 in Supplemental Materials Ⅰ). The EAT-Lancet planetary health diet was associated with decreased risks of colorectal cancer (pooled HR = 0.87, 95% CI: 0.81, 0.93, *P* = 0.008, *n* = 4, 812,074 participants, *I*^2^ = 0, *τ*^2^ < 0.001, low certainty) and lung cancer (pooled HR = 0.69, 95% CI: 0.56, 0.84, *P* = 0.016, *n* = 3, 336,011 participants, *I*^2^ = 0, τ^2^ < 0.001, low certainty), especially small-cell lung cancer (pooled HR = 0.57, 95% CI: 0.47, 0.70, *P* = 0.019, *n* = 2, 273,629 participants, *I*^2^ = 0, τ^2^ < 0.001), but not the overall risk of cancers or breast cancer or prostate cancer.

Pooled results of prospective cohort studies showed that higher adherence to the EAT-Lancet planetary health diet was linked with a 17% decrease in CVD risk (pooled HR = 0.83, 95% CI: 0.74, 0.94, *P =* 0.007, *n* = 10, 1,027,822 participants, *I*^2^ = 75.2%, τ^2^ = 0.012, very low certainty), especially for coronary artery disease, heart failure, and stroke (Table 1 and Appendix 7 in Supplemental Materials Ⅰ). For CVD risk factors, pooled results from prospective cohort studies showed favorable associations of the EAT-Lancet planetary health diet with type 2 diabetes (pooled HR = 0.75, 95% CI: 0.57, 0.98, *P =* 0.040, *n* = 8, 551,687 participants, *I*^2^ = 96.5%, τ^2^ = 0.094, very low certainty), but not with levels of blood pressure, fasting plasma glucose, LDL cholesterol, and HDL cholesterol. In prospective cohort and cross-sectional studies, participants with higher EAT-Lancet planetary health diet adherence had lower risks of metabolic dysfunction–associated steatotic liver disease (pooled HR = 0.80, 95% CI: 0.71, 0.92, *P* = 0.013, *n* = 2, 297,230 participants, *I*^2^ = 37.1%, τ^2^ = 0.003), asthma, and depression (pooled HR = 0.73, 95% CI: 0.65, 0.81, *P* < 0.001, *n* = 5, 90,199 participants, *I*^2^ = 0, τ^2^ < 0.001), but not anxiety or dementia (Table 1). Studies included in the systematic review had similar findings (Supplemental Table 3 in Appendix 3 in Supplemental Materials Ⅰ).

**FIGURE 2:**
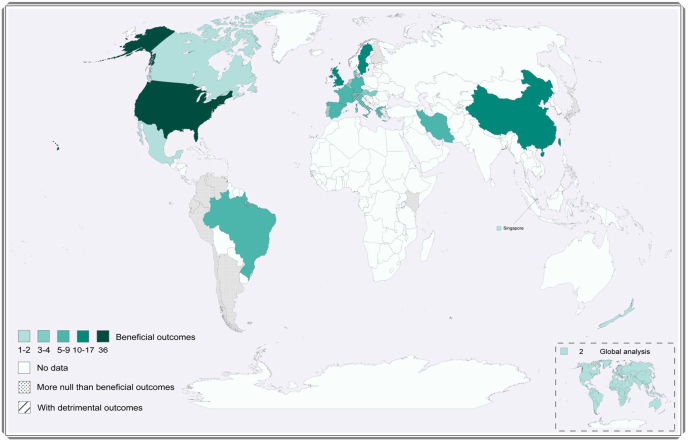
Global map of cumulative count of reported major beneficial population health outcomes associated with the EAT-Lancet planetary health diet.  Blank areas represent countries/regions with no reported health outcomes;  light-to-dark green shades denote the cumulative count of reported major beneficial health outcomes (1–2, 3–4, 5–9, 10–17, 36);  thin diagonal stripes indicate the presence of detrimental outcomes;  textured pattern indicates that null outcomes outnumber beneficial ones; global syntheses that could not be disaggregated by country/region are shown separately in the bottom right corner.

A global map (Figure 2) presents the cumulative count of reported major beneficial population health outcomes associated with the EAT-Lancet planetary health diet in countries and regions around the world. A considerable portion of the research is based on populations from the United States, the United Kingdom, China, Sweden, Iran, and Brazil. Except for Italy, the EAT-Lancet planetary health diet was either beneficial or neutral with respect to major health outcomes in all countries and regions studied. In these reports, the cumulative count of beneficial associations between the EAT-Lancet planetary health diet and major health outcomes outnumbered that of null associations.

### The EAT-Lancet planetary health diet and environmental sustainability

The associations of the EAT-Lancet planetary health diet with GHGE, land use, and water use are shown in Table 2 and Appendix 7 in Supplemental Materials Ⅰ. Higher adherence to the EAT-Lancet planetary health diet was associated with a reduction of 1.55 (95% CI: 0.22, 2.89) kg CO_2_ eq/d/person of GHGE (*P =* 0.027, *n* = 12, 882,673 participants, *I*^2^ = 100.0%, τ^2^ = 4.363). In addition, the EAT-Lancet diet was also related to a reduction of freshwater eutrophication, marine eutrophication, terrestrial acidification, and fertilizer needs. Higher adherence to the EAT-Lancet planetary health diet was less likely to reduce land use and blue water use. Modeling studies and comparative studies suggested that compared with current diets, the EAT-Lancet planetary health diet could reduce GHGE/carbon footprints, land use/land footprints, water use/blue water use/water footprints, and biodiversity threat footprints (Supplemental Table 4 in Appendix 3 in Supplemental Materials Ⅰ). Global modeling studies estimated that compared with the current diets, the EAT-Lancet planetary health diet would reduce agricultural GHGE in 101 countries as well as globally, whereas in primarily low-to-middle-income countries, agricultural GHGE would increase by 12%–283%. In addition, the water footprint would fall by 12% at a global level but increase for nearly 40% of the world’s population. The EAT-Lancet planetary health diet would cause a 3%–4% reduction of the expansion of agricultural land use globally but increase land demand in sub-Saharan Africa. No studies reported the associations of the EAT-Lancet planetary health with air pollution.

**TABLE 2:** Random effects meta-analysis for the association between adherence to the EAT-Lancet planetary health diet and environmental sustainability

Outcome	Study design	Type of effect size	Forest plot	No. of studies	No. of comparisons	No. of participants	Effect size	LCI	UCI	Effect size (95% CI)	*P*	*P* for Heterogeneity	τ^2^	*I*^2^ (%)	Funnel plot	*P* for Egger	Leave-one-out plot
1. Greenhouse gas emissions																	
Greenhouse gas emissions	Cross-sectional	Mean	S1-20	12	12	882,673	−1.55	−2.89	−0.22	−1.55 (−2.89, −0.22)	0.027	<0.001	4.363	100.0	S2-26	0.376	S3-26
Greenhouse gas emissions	Cross-sectional	*β*	S1-21	5	6	94,028	−0.66	−1.96	0.64	−0.66 (−1.96, 0.64)	0.249	<0.001	1.447	100.0	S2-27	0.314	S3-27
Carbon footprint				2	2	6689	−0.29	−4.07	3.50	−0.29 (−4.07, 3.50)	0.512	<0.001	0.169	95.4	—	—	—
2. Land use																	
Land use	Cross-sectional	Mean	S1-22	9	9	836,294	−5.21	−11.08	0.67	−5.21 (−11.08, 0.67)	0.075	<0.001	56.381	100.0	S2-28	0.423	S3-28
Land use	Cross-sectional	*β*	S1-23	3	4	64,708	−0.26	−0.87	0.36	−0.26 (−0.87, 0.36)	0.275	<0.001	0.144	97.6	S2-29	0.085	S3-29
3. Water use																	
Water footprint	Cross-sectional	Mean	S1-24	3	3	72,845	0.36	−0.15	0.88	0.36 (−0.15, 0.88)	0.094	<0.001	0.054	99.4	S2-30	0.568	S3-30
Blue water use				3	3	242,425	0.02	−0.31	0.35	0.02 (−0.31,0.35)	0.824	<0.001	0.017	99.1	S2-31	0.948	S3-31
Total water footprint	Cross-sectional	*β*	S1-25	3	3	69,946	−0.30	−1.84	1.23	−0.30 (−1.84, 1.23)	0.698	<0.001	0.359	100.0	S2-32	0.452	S3-32

Abbreviations: CI, confidence interval; Mdiff, mean difference.

**FIGURE 3:**
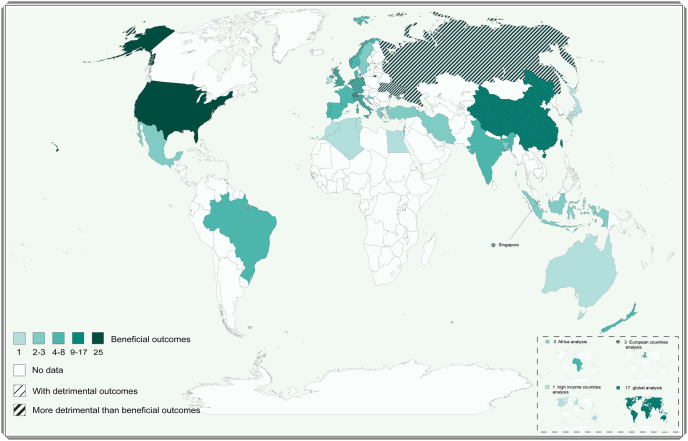
Global map of cumulative count of reported major beneficial environmental outcomes associated with the EAT-Lancet planetary health diet.  Blank areas represent countries/regions with no reported major environmental outcomes;  light-to-dark green shades denote the cumulative count of reported major beneficial health outcomes (1, 2–3, 4–8, 9–17, 25);  thin diagonal stripes indicate the presence of detrimental outcomes;  thick diagonal stripes indicate that detrimental outcomes outnumber beneficial ones; global/continental/high-income country syntheses that could not be disaggregated by country/region are shown separately in the bottom right corner.

The key findings on environmental sustainability across countries are shown in Figure 3, which presents the cumulative count of reported major beneficial environmental outcomes associated with the EAT-Lancet planetary health diet. Eleven studies are global analyses. Among single-country/region studies, the United States, China, the United Kingdom, Italy, and Denmark accounted for the largest proportion of research. In the included studies, the cumulative count of beneficial associations between the EAT-Lancet planetary health diet and major environmental outcomes outnumbered that of null and negative associations. Studies from several countries or regions such as Brazil, China, Germany, Italy, Singapore, Switzerland, the Netherlands, and the United Kingdom reported negative associations. China and Italy reported null associations.

### The EAT-Lancet planetary health diet and safety (nutrient deficiencies)

Supplemental Table 5 in Appendix 3 in Supplemental Materials Ⅰ shows the included studies on the EAT-Lancet planetary health diet and safety. Most included cross-sectional studies, modeling studies, and comparative studies showed that higher adherence to the EAT-Lancet planetary health diet was associated with higher intake of carbohydrate, plant proteins, and dietary fiber and lower consumption of animal proteins among children, adolescents, and adults. In addition, higher adherence to the EAT-Lancet planetary health diet was associated with lower intake of vitamins D and B_12_ (Supplemental Table 5 in Appendix 3 in Supplemental Materials Ⅰ).

**FIGURE 4:**
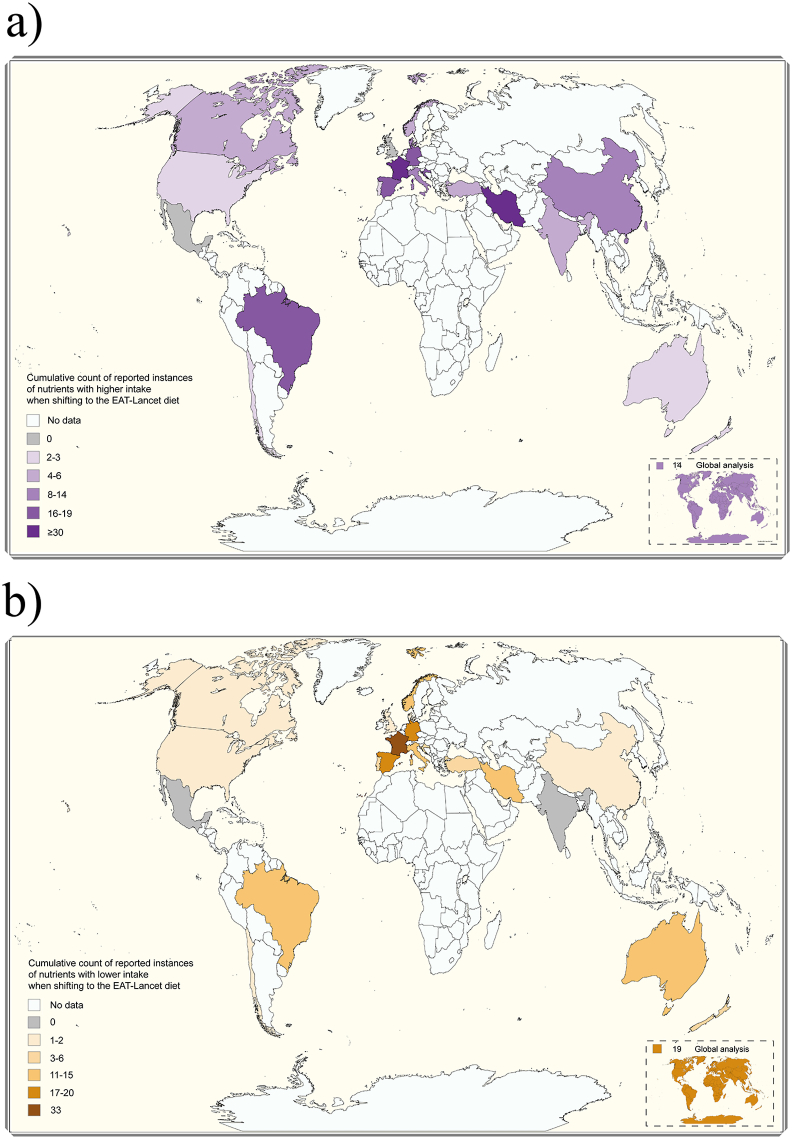
Global map of cumulative count of reported instances of nutrients at risk of higher or lower intakes under the EAT-Lancet planetary health diet.  Blank areas represent countries/regions with no reported nutrient changes when shifting to the EAT-Lancet planetary health diet.  Grey areas indicate countries/regions with reported nutrient change records but a cumulative count of zero; (A)  light-to-dark purple shades denote the cumulative count of reported instances of nutrients (2–3, 4–6, 8–14, 16–19, ≥30); (B)  light-to-dark amber shades denote the cumulative count of reported instances of nutrients (1–2, 3–6, 11–15, 17–20, 33); global syntheses that could not be disaggregated by country/region are shown separately in the bottom right corner, respectively.

Figure 4 shows a global map of cumulative counts of reported instances of nutrient deficiency risks (safety) associated with the EAT-Lancet planetary health diet. Most of the included studies are from France, Iran, Germany, and China (Supplemental Table 1 in Supplemental Materials Ⅱ). There were country- or region-specific nutrient deficiency patterns. For countries or regions such as Brazil, Canada, China, Croatia, Denmark, France, India, and Iran, shifts to the EAT-Lancet planetary health diet would result in a greater cumulative count of nutrients at risk of higher intakes (or sufficient) than at risk of lower intakes (or deficient). The opposite is true for some countries such as Australia and Norway, where transition to the EAT-Lancet planetary health diet would cause a greater cumulative count of nutrients being deficient than being sufficient. Globally, shifts to the EAT-Lancet planetary health diet would result in a greater cumulative count of nutrients at risk of lower intakes. In terms of micronutrient deficiencies, the EAT-Lancet planetary health diet was less likely to induce micronutrient deficiencies in Congo, Ecuador, Kenya, Sri Lanka, and Vietnam, whereas would result in deficiencies in micronutrient, particularly vitamins A, D, or B_12_ or calcium, zinc, or iodine, in Denmark, Norway, Spain, France, Croatia, Brazil, Italy, Australia, New Zealand, Mexico, and China among children, adolescents, and adults (Supplemental Table 5 in Appendix 3 in Supplemental Materials Ⅰ). On a global average, compared with globally harmonized recommended nutrient intakes for adults and women of reproductive age, the EAT-Lancet diet would not result in deficiencies in folate and vitamin A, but would induce deficiencies in vitamin B_12_, calcium, iron, and zinc. In particular, the EAT-Lancet planetary health diet may cause iodine deficiency in pregnant or lactating women and micronutrient deficiencies in women of reproductive age in some countries and regions (Supplemental Table 5 in Appendix 3 in Supplemental Materials Ⅰ).

### The EAT-Lancet planetary health diet and affordability

**FIGURE 5:**
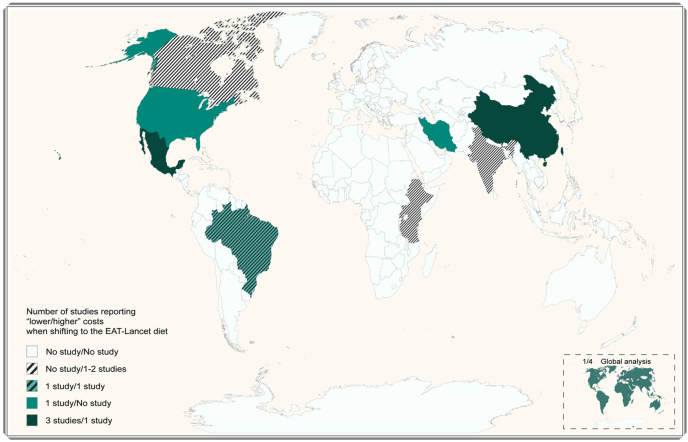
Global map of the number of studies reporting the EAT-Lancet planetary health diet as affordable.  Blank areas represent countries/regions with no reported studies on the affordability of the EAT-Lancet planetary health diet;  thick diagonal stripes represent that no study reported lower cost, and 1–2 studies reported higher costs when shifting to the EAT-Lancet planetary health diet;  represent that 1 study reported lower cost, and 1 study reported higher cost;  represent that 1 study reported lower cost, and no study reported higher cost;  represent that 3 studies reported lower costs, and 1 study reported higher cost; global syntheses that could not be disaggregated by country/region are shown separately in the bottom right corner.

Figure 5 presents a global map of the number of studies reporting the EAT-Lancet planetary health diet as affordable across different countries and regions. There are 5 global analyses. At the country level, China and Mexico contribute the most studies, with 4 publications each. Cross-sectional studies and modeling studies demonstrated that transition from the current diets or the diets recommended in dietary guidelines to the EAT-Lancet planetary health diet would result in higher cost of food consumption in many countries or regions, regardless of the income level. In particular, people in some high-income countries such as New Zealand and Canada, and some low-to-middle-income countries such as India, Ethiopia, Kenya, Tanzania, and Uganda would spend more if shift to the EAT-Lancet planetary health diet. However, for Mexico, the United States, Iran, and China, shifts to this diet would result in food expenditure savings. The detailed characteristics of the included studies on the EAT-Lancet planetary health diet and affordability are shown in Supplemental Table 6 in Appendix 3 in Supplemental Materials Ⅰ. Global modeling studies estimated that the cost of the EAT-Lancet planetary health diet would be higher in high-income countries than in low-income countries, and the cost was highest in the Latin America and Caribbean region and lowest in sub-Saharan Africa. Globally, ∼1.6–3.0 billion people were not able to afford the cost of the EAT-Lancet planetary health diet (Supplemental Table 6 in Appendix 3 in Supplemental Materials Ⅰ).

### Gaps between current global diets and the EAT-Lancet planetary health diet

**FIGURE 6:**
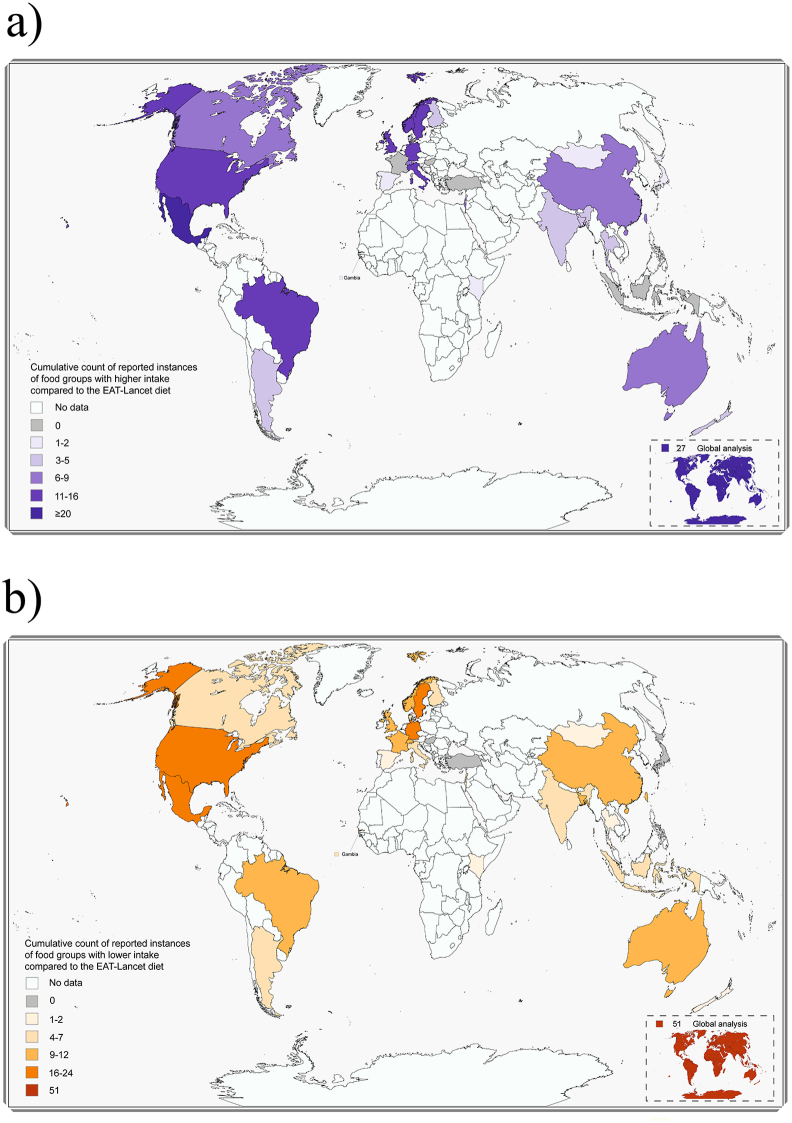
Global map of cumulative count of reported instances of food groups whose current average intake exceeds or falls short of the EAT-Lancet recommendations.  Blank areas represent countries/regions with no studies comparing the differences between the national current diet and the EAT-Lancet planetary health diet;  grey areas indicate countries/regions with reported nutrient change records but a cumulative count of zero; A)  light-to-dark purple shades denote the cumulative count of reported instances of food groups (1–2, 3–5, 6–9, 11–16, ≥20); (B)  light-to-dark amber shades denote the cumulative count of reported instances of food groups (1–2, 4–7, 9–12, 16–24, 51); Global syntheses that could not be disaggregated by country/region are shown separately in the bottom right corner, respectively.

Figure 6 presents a global map of the cumulative count of reported instances of food groups whose current average intake exceeds or falls short of the EAT-Lancet recommendations. Some countries or regions such as Canada, Ireland, Israel, Norway, and the United Kingdom would have a greater cumulative count of food groups whose current average intake exceeds the EAT-Lancet recommendations (Supplemental Table 2 in Supplemental Materials Ⅱ). In contrast, some countries or regions such as Australia, Bangladesh, China, France, Gambia, India, Indonesia, and Switzerland would experience the opposite pattern. At the global level, the cumulative count of food groups where average intake would exceed the EAT-Lancet recommendations is lower than those where it would fall short.

At the level of specific food groups, compared with the EAT-Lancet planetary health diet, current global dietary patterns generally contained higher red meat, animal fats, and starchy vegetables, and lower wholegrains, nonstarchy vegetables, and legumes among children, adolescents, and adults in both high-income and low-and-middle-income countries (Supplemental Table 7 in Appendix 3 in Supplemental Materials Ⅰ). At the regional level, compared with the EAT-Lancet recommendations, current North American diets contained excess eggs, poultry, and dairy but insufficient fish, fruit, and nuts. For Latin America, current diets comprised excess eggs and poultry, whereas insufficient dairy, fish, fruit, and nuts (Supplemental Table 7 in Appendix 3 in Supplemental Materials Ⅰ). Current European and central Asian diets consisted of excess eggs, poultry, and total dairy but insufficient fish, fruit, and nuts. Current East Asia Pacific and South Asian diets contained insufficient poultry, dairy, fruit, and nuts. Current Middle East and North African diets contained excess eggs and poultry, whereas insufficient dairy, fish, fruit, and nuts. Current diets in sub-Saharan Africa did not include sufficient eggs, poultry, dairy, fish, fruit, and nuts.

### Small study effects and sensitivity analysis

Appendix 8 in Supplemental Materials Ⅰ shows the assessments for small study effects. In terms of the studies included in the meta-analysis investigating the associations of EAT-Lancet planetary health diet with population health and environmental outcomes, no evidence for funnel plot asymmetry was found (Egger’s test, *P* > 0.05 for all). Sensitivity analyses showed that the results of associations between the EAT-Lancet diet and population health and environmental outcomes were not driven by a single study (Appendix 9 in Supplemental Materials Ⅰ).

## Discussion

In this multidisciplinary systematic review and meta-analysis of 227 studies, we found that adoption of the EAT-Lancet planetary health diet was associated with lower risks of mortality, CVD, type 2 diabetes, but not overall risks of cancer or dementia. Moreover, this diet was associated with reduced GHGE, whereas no strong evidence supported the benefits to water and land use. Compared with many national dietary guidelines, the EAT-Lancet planetary health diet would increase intakes of plant proteins and fiber, but potentially induce deficiencies in vitamin B_12_, calcium, iron, and zinc in adults, particularly women of reproductive age, in certain contexts. There still needs to be major changes across multiple sectors in many geographical areas to make the EAT-Lancet planetary diet affordable. Compared with the EAT-Lancet planetary health diet, current dietary patterns generally contained more red meat, animal fats, and starchy vegetables but lower wholegrains, nonstarchy vegetables, and legumes. More than half of studies in our meta-analysis were of high quality, whereas all the evidence was derived from observational studies, resulting in low or very low certainty.

The EAT-Lancet Commission and its proposal for sustainable food systems and diets will have a huge influence on academic research and agricultural and public health policy. Currently, the inclusion of the sustainability constraints in food-based dietary guidelines is in progress in 37 countries [247,248]. A recent systematic review, however, suggested that the EAT-Lancet Commission report and the EAT-Lancet planetary health diet had several limitations and they were limited to potential for unintended health consequences, lack of awareness of food affordability and accessibility, and bias toward high-income countries [8]. We therefore conducted a multidisciplinary and comprehensive systematic review and meta-analysis of data from both high- and low-and-middle-income countries to address these concerns.

In terms of population health, previous systematic reviews of observational studies indicated the favorable associations between the EAT-Lancet planetary health diet and type 2 diabetes and its complications [249,250]. Our review further examined other population health outcomes such as all-cause and cause-specific mortality, cancers, cerebro-CVD and related risk factors, liver diseases, and mental disorders. Moreover, we synthesized the data from 53 relevant studies and showed that the EAT-Lancet planetary health diet was associated with lower risks of mortality, CVD, type 2 diabetes, metabolic dysfunction–associated steatotic liver disease, asthma, and depression, but not with overall risks of cancers or dementia. Compared with the EAT-Lancet planetary health diet, the Mediterranean diet, a famous and widely recommended diet, has been found to reduce overall risks of cancer and CVD, metabolic or neurodegenerative diseases, especially dementia [251]. The United Nations Educational, Scientific and Cultural Organization Chair on Health Education and Sustainable Development is fostering a research project (“Planeterranea”), aiming to identify a healthy dietary pattern based on local foods with the same Mediterranean diet features and planetary health effects [252,253]. It would be interesting to compare the EAT-Lancet planetary health diet with the “Planeterranean” diet. There are large-scale, randomized trials examining the Mediterranean diet and health outcomes in children and adults [254,255]. In the present review, we attempted to include randomized trials on the EAT-Lancet planetary health diet and population health, but unfortunately found none. For this reason, the certain of the evidence was low or very low. Notably, female reproductive health (e.g., pregnancy rate, maternal outcomes, and neonatal outcomes) are vital to the development of a whole generation and therefore a country’s health and socioeconomic productivity. Common deficiencies at conception and in utero such as iron and iodine cause permanent cognitive deficits in children [256,257]. Previous meta-analyses have demonstrated that adherence to the Mediterranean diet was associated with lower risks of adverse pregnancy outcomes [254,258], whereas, to date, there are no studies examining the EAT-Lancet planetary health diet and female reproductive health and offspring health.

Some previous intervention studies demonstrated the beneficial effects of the Mediterranean diet on environmental sustainability [259]; however, few clinical trials focused on the environmental impact of the EAT-Lancet planetary health diet. Only one systematic review encompassing one relevant cross-sectional study summarized the associations of the EAT-Lancet diet with GHGE and land use [249]. That review showed that adherence to the diet was inversely associated with GHGE and land use. Our findings were summarized from many more studies, especially recent studies. Our meta-analysis demonstrated that higher adherence to the EAT-Lancet planetary health diet was related to lower GHGE, but we cannot yet conclude that the diet would save water or land. Conversely, our systematic review indicated that compared with current diets, the EAT-Lancet planetary health diet exhibited potential for reducing eutrophication and fertilizer needs as well as mitigating water and land use. The conflicting results regarding land and water use observed in the meta-analysis compared with the systematic review may be attributed to differences in study type and comparator. Specifically, studies included in the meta-analysis were all epidemiological in nature, whereas those in the systematic review included modeling and comparative studies. Findings from modeling studies tend to be optimized and ideal. In addition, studies included in the meta-analysis used lower adherence to the diet as a comparator, whereas most studies included in the systematic review used the current diet as a comparator. Moreover, the EAT-Lancet diet is not guaranteed to reduce water or land use because all agriculture inherently demands land and water. This resource requirement is an unavoidable feature of natural systems and any food production they support. In particular, some plant-based foods such as almonds have a high water footprint [260]. GHGE was the most extensively investigated outcome/indicator, whereas further exploration is needed for other environmental indicators, especially eutrophication, fertilizer needs, biodiversity, and air pollution. It should also be noted that our findings were based on adolescents and adults, whereas a recent review addressed infant and young child feeding recommendations from a planetary health perspective [261].

As a flexitarian diet, the EAT-Lancet planetary health diet has raised concerns about the extent to which the diet provides sufficient essential nutrients, particularly those generally found in higher quantities and in more bioavailable forms in animal-based foods. Many studies have reported nutrient intake in relation to the EAT-Lancet planetary health diet, but our study is the first to review previous studies systematically. Our systematic review of 24 relevant studies showed that on a global average, compared with globally harmonized recommended nutrient intakes for adults and women of reproductive age, the EAT-Lancet planetary health diet would not result in deficiencies in folate and vitamin A, but would induce deficiencies in vitamin B_12_, calcium, iron, and zinc. It, however, represents a global average estimate and should not be generalized to all countries or populations, as local variations in income levels, dietary habits, and food availability may significantly influence actual nutritional outcomes. Specifically, vitamin B_12_ deficiency is more likely in regions where strict vegan diets are common and where neither B_12_ fortification nor widespread supplement use is practiced [262]. Iron and calcium deficiencies tend to be more common in areas with historically low consumption of red meat, as well as among populations reliant on plant-based sources of iron whose diets lack enhancers such as vitamin C [[263], [264], [265]]. Zinc deficiency is more common in regions where diets are based largely on unfermented whole grains and legumes with high phytate content, which reduces zinc bioavailability [266]. Moreover, we observed country- or region-specific micronutrient deficiencies: the EAT-Lancet planetary health diet would additionally induce deficiencies in vitamin D or iodine in some high-income countries such as Australia, Italy, and Norway. For the sake of the next generation, adequate iodine intake is important before and during pregnancy and inadequate iodine intake has detrimental effects on fetus growth and brain development [168]. Furthermore, we show that women of reproductive age may not obtain adequate micronutrients from the EAT-Lancet planetary health diet. A previous study suggested modifications to the original EAT-Lancet planetary health diet to achieve micronutrient adequacy [12]. Our findings support that proposal and further suggest that country- or region-specific planetary dietary pattern may be needed and foods rich in vitamin B_12_, calcium, iron, zinc, vitamin D or iodine may be recommended for up to a third of the population (women of reproductive age).

Despite decades of agricultural development and a net increase in food availability worldwide, pronounced gaps in healthy food access and affordability remain [267]. The “Great Food Transformation” and the global diet shifts require that the necessary foods be affordable, whereas the EAT-Lancet Commission report did not take cost or affordability into consideration [13]. Inequities in healthy food affordability especially among disadvantaged populations can inform EAT-Lancet–aligned policies [267]. Our study was the first systematic review to evaluate the affordability of the EAT-Lancet planetary health diet from a global and regional perspective. Our summary data indicate that there still need to be major changes across multiple sectors in many geographical areas to make the EAT-Lancet planetary diet affordable. Although the cost of the EAT-Lancet planetary health diet is higher in high-income countries than in low-income countries, these expenses constitute only a small fraction of average incomes. However, for low-and-middle-income countries, especially the Latin America and Caribbean region, implementing such a diet may prove unaffordable because of economic constraints. A global analysis indicated that fruits and vegetables along with animal-source foods were the most expensive components of the EAT-Lancet planetary health diet; however, in low-and-middle-income countries vegetables tend to be more affordable, whereas animal-source foods remain equally or even more expensive than those in high-income countries [13]. It is worth noting that no single dietary model, including current mainstream diets, can resolve the hunger and nutritional access barriers caused by poverty [268]. Even a dietary model widely recognized as optimal for environmental sustainability cannot transcend existing socioeconomic structures to automatically provide an affordable solution for all humans [268]. In the transition toward a sustainable food system, economic accessibility is a key determinant as critical as health benefit, nutritional adequacy, and environmental sustainability, and it must be actively constructed through targeted social, economic, and policy interventions, such as income support, price subsidies, and social protection systems, rather than relying on the dietary model itself. Taking together, economic inequality poses as an obstacle hindering widespread adoption of the EAT-Lancet recommendations. To facilitate transition toward this dietary pattern in low-and-middle-income countries, the government should formulate relevant food production and agricultural policies to provide subsidies and support for nutrient-dense crops and healthy foods, particularly plant-based foods such as vegetables and fruits; additionally, the government should implement economic and social policies, such as income support or fiscal measures (e.g., taxation or subsidies), to enhance the purchasing power of vulnerable populations for healthy foods; furthermore, investments should be made in storage, transportation, and local market development to reduce the costs and postharvest losses of perishable healthy foods [267,269].

Our findings suggest that there is a gap between current global diets and the EAT-Lancet planetary health diet. To shift to the EAT-Lancet planetary health diet, individuals in both high-income and low-and-middle-income countries should increase their consumption of wholegrains, nonstarchy vegetables, and legumes while reducing their intake of red meat, animal fat, and starchy vegetables. Country- and region-specific food adjustments will be necessary for successful implementation. Transition to the EAT-Lancet planetary health diet encompasses more than simply adjusting food intake as identified by a recent systematic review, which highlights various barriers to adoption including financial constraints, lack of knowledge, nutrition, culture, enjoyment of meat, accessibility, affordability, personal ability, and media [270]. Governments, policy makers, and food companies can play a crucial role in overcoming barriers by providing financial incentives, improving public health and sustainable development education, understanding personal food choice, safeguarding cultural diversity, and producing plant-based meat alternatives for meat lovers [267,[271], [272], [273]].

Despite many epidemiological studies examining the EAT-Lancet planetary health diet and population health, unfortunately no study reported female reproductive health outcomes. Reports on liver diseases, mental disorders, respiratory system diseases, and immune system diseases are also very limited. Considering environmental sustainability, most studies paid attention to GHGE and water and land use, as suggested indicators such as biosphere integrity, novel entities, and impacts on natural resources are expected to be studied [274]. Future studies are expected to focus on these outcomes or indicators. In addition, randomized clinical trials examining the effects of the EAT-Lancet planetary health diet on population health and environmental sustainability are needed and the representation of low-and-middle-income populations needs to be improved. As in previous studies, food production and food consumption were studied separately, more comprehensive systematic reviews and meta-analyses integrating entire food chain to understand the whole-system effects of production and consumption choices (e.g., life cycle assessment [275,276]) on population and planetary health are needed. Policy studies and the EAT-Lancet Commission 2.0 should focus on improving equity and justice across resources and access to healthy, sustainable, and affordable foods and secure a shift to healthy and sustainable diets for all.

Our study has several strengths. First, we strictly adhered to guidelines for systematic reviews and meta-analyses (i.e., the Cochrane Handbook for Systematic Reviews of Interventions, MOOSE, and PRISMA 2020) and the PICO framework. We also employed recommended and appropriate statistical synthesis methods (i.e., restricted maximum likelihood method and Hartung–Knapp method). Second, we conducted a multidisciplinary and comprehensive systematic review and meta-analysis of recent studies to assess the EAT-Lancet planetary health diet. We not only provide deep insights into advantages (i.e., population health and environmental sustainability) and disadvantages (i.e., nutrient deficiencies and high cost in some regions) of adoption of the EAT-Lancet diet, but also highlighted the gap between current diets and the EAT-Lancet planetary health diet. Finally, our findings were robust and reliable, as most studies included in the current meta-analysis were of high quality and our results were consistent in the sensitivity analyses. Notwithstanding the strengths, certain limitations merit consideration. First, no randomized clinical trials were eligible to be included in our meta-analysis. Compared with randomized trials, the studies (i.e., prospective cohort studies and cross-sectional studies) included in this meta-analysis are prone to bias and may have lower certainty evidence. Second, some outcomes such as some specific cancers, male reproductive health, freshwater eutrophication, terrestrial acidification, and fertilizer needs were reported by one study only, which made data synthesis impossible. In addition, this study did not take into account some key determinants of global food system transformation and transition to the EAT-Lancet planetary health diet such as agricultural production, food processing, dietary culture, international trade, political factors, and geopolitical conflicts. Finally, in consideration of the unreliability of translation software and the reproducibility of this study, we included data published in English only. Articles written in other languages may provide new information.

In conclusion, this multidisciplinary systematic review and meta-analysis showed that adoption of the EAT-Lancet planetary health diet was associated with lower risks of mortality, CVD, diabetes, liver diseases, and depression as well as a reduction of GHGE, but no strong evidence supported the benefits of the EAT-Lancet planetary health diet regarding water and land use. Additionally, there were nutrient deficiencies in pregnant women and those of reproductive age in some countries and regions. Achieving affordability for this diet will require coordinated, major changes across multiple sectors and regions. There were gaps between current global diets and the EAT-Lancet planetary health diet in the most regions around the world. Our review supported the health and environment cobenefits of the EAT-Lancet planetary health diet, whereas more studies on the EAT-Lancet planetary health diet, female reproductive health and offspring health, as well as water and land use and other environmental outcomes (e.g., biodiversity) are needed. Governments and policy makers should address potential nutrient deficiencies in pregnant women and those of reproductive age, along with economic inequalities before transition to the EAT-Lancet planetary health diet.

## Author contributions

The authors’ responsibilities were as follows – RZ, JH, LW, FR, HC: contributed to the study conception and design; RZ: wrote the systematic review protocol; RZ, LW, HC: defined the search strings, executed the search, and exported the results; JH, HC: analyzed the data and performed the meta-analysis; RZ, LW: performed quality assessment; RZ: drafted the manuscript; LW, HC, JR, RW, YGong, YGuan, ML, QW, ZX, LZ, PA, JG, XH, MX, KL, JAM, AR, JB-M, FR, JH: critically revised the manuscript for important intellectual content; all authors: commented on the drafts and approved the final draft; JH, RZ: are the guarantors; JH, FR: attest that all listed authors meet authorship criteria and that no others meeting the criteria have been omitted; all authors: had full access to all the data in the study; JH: had final responsibility for the decision to submit for publication; and JH, HC: take responsibility for the accuracy of the data analysis.

## Data availability

The corresponding author (JH) should be contacted for any requests (e.g., template data collection forms; data extracted from included studies; data used for all analyses; analytic code; any other materials used in the current systematic review and meta-analysis).

## Declaration of generative AI and AI-assisted technologies in the writing process

The authors did not use any AI at all in the writing process.

## Funding

This work was supported by China Dairy Industry Association Dairy Science and Technology Innovation Fund-Mengniu Special Research Support Project (CDIAKCJJ-MN-2023-001), the 9th China Association for Science and Technology Youth Talent Promotion Project (2023–2026) (202404623140567), and the China Postdoctoral Science Foundation (Grant number: 2023T00376 and 2023M743787). The funders had no role in considering the study design or in the analysis, interpretation of data, writing of the report, or decision to submit the article for publication. National Key Research and Development Program: Precision Nutrition and Health Food Development and Industrial Demonstration for Typical Chronic Disease Populations (Grant No. 2024YFF1106000).

## Conflict of interest

AR has received honorariums from the International Sweeteners Association, Nestlé, and Unilever and research funds from Novo Nordisk A/S. JB-M reports a relationship with China National Research Institute of Food and Fermentation Industries that includes: consulting or advisory, speaking and lecture fees, and travel reimbursement. JB-M reports a relationship with Novo Foundation that includes: consulting or advisory. JB-M reports a relationship with Hachette Livre that includes: a coauthor of books about the glycemic index of foods and diabetes. She oversees a glycemic index testing service at the University of Sydney. She was President of the Glycemic Index Foundation between 2002 and 2024. She is on the Editorial Boards of the American Journal of Clinical Nutrition and the International Journal of Obesity. She is on the Scientific Board of Zoe Global, a personalized nutrition company. The other authors report no conflicts of interest.
