# Supplementary material for: The Health and Environmental Impacts, Safety, and Affordability of the EAT-Lancet Planetary Health Diet: A Multidisciplinary Systematic Review and Meta-Analysis
Source: Adv Nutr. 2026 Jun 1;17(7):100666. doi: 10.1016/j.advnut.2026.100666 (PMC13312486; doi:10.1016/j.advnut.2026.100666)
Supplement: Multimedia component 1 [file mmc1.docx]

Ruixin Zhu et al. The health and environmental impacts, safety, and affordability of the EAT-Lancet planetary health diet: a multidisciplinary systematic review and meta-analysis

**Supplementary materials Ⅰ**

**Content**

**[Appendix 1 Search strategy](#_Toc219108825)** [2](#_Toc219108825)

**[Appendix 2 Inclusion criteria](#_Toc219108826)** [4](#_Toc219108826)

**[Appendix 3 Detailed characteristics of all included studies](#_Toc219108827)** [5](#_Toc219108827)

**[Appendix 4 Key excluded studies](#_Toc219108828)** [143](#_Toc219108828)

**[Appendix 5 The Newcastle-Ottawa Scale quality assessment table for meta-analysis](#_Toc219108829)** [147](#_Toc219108829)

**[Appendix 6 GRADE assessment](#_Toc219108830)** [150](#_Toc219108830)

**[Appendix 7 Forest plots of the EAT-Lancet planetary health diet and population health and environment outcomes](#_Toc219108831)** [152](#_Toc219108831)

**[Appendix 8 Funnel plots of population health and environment outcomes](#_Toc219108832)** [171](#_Toc219108832)

**[Appendix 9 Sensitivity analysis](#_Toc219108833)** [187](#_Toc219108833)

**Appendix 1 Search strategy**

**Table S1. Search strategy**

| **PubMed** |  |  |
| --- | --- | --- |
| **Date** | **June 19, 2025** |  |
| Process | Search terms | No. of results |
| #1 | ("EAT Lancet"[Title/Abstract] OR"EAT-Lancet"[Title/Abstract] OR "planetary health diet*"[Title/Abstract] OR "reference diet*"[Title/Abstract] OR "planetary diet*"[Title/Abstract] OR "Lancet diet*"[Title/Abstract]) | 727 |
| #2 | (2019/1/1:2025/06/19[pdat]) |  |
| #3 | #1 AND #2 | 488 |
| **Embase** |  |  |
| **Date** | **June 19, 2025** |  |
| Process | Search terms | No. of results |
| #1 | ('eat lancet':ab,ti OR 'eat-lancet':ab,ti OR 'planetary health diet*':ab,ti OR 'reference diet*':ab,ti OR 'planetary diet*':ab,ti OR 'lancet diet*':ab,ti) | 886 |
| #2 | [01-01-2019]/sd NOT [19-06-2025]/sd |  |
| #3 | #1 AND #2 | 619 |
| **Web of Science** |  |  |
| **Date** | **June 19, 2025** |  |
| Process | Search terms | No. of results |
| #1 | TS=("EAT Lancet") OR TS=("EAT-Lancet") OR TS=("planetary health diet*") OR TS=("reference diet*") OR TS=("planetary diet*") OR TS=("lancet diet*") | 1558 |
| #2 | Timespan: 2019-01-01 to 2025-06-19 (Publication Date) | 732 |
| **EBSCOhost CINAHL** |  |  |
| **Date** | **June 19, 2025** |  |
| Process | Search terms | No. of results |
| #1 | XB "EAT Lancet" OR XB EAT-Lancet" OR XB “planetary health diet*” OR XB "reference diet*" OR XB planetary diet*" OR XB "Lancet diet*" | 207 |
| #2 | filter: 2019/01/01-2025/06/19 |  |
| #3 | #1 AND #2 | 122 |
| **Cochrane Library** |  |  |
| **Date** | **June 19, 2025** |  |
| Process | Search terms | No. of results |
| #1 | ("EAT Lancet"):ti,ab,kw OR ("EAT-Lancet"):ti,ab,kw OR ("planetary health diet"):ti,ab,kw OR ("reference diet"):ti,ab,kw OR ("planetary diet"):ti,ab,kw OR ("Lancet diet"):ti,ab,kw | 63 |
| #2 | Publication Date: 20190101-20250619 (Manually) | 39 |
| **ClinicalTrials.gov** |  |  |
| **Date** | **June 19, 2025** |  |
| Other Terms | Intervention/Treatment |  |
| EAT-Lancet diet (2) | EAT-Lancet diet (1) |  |
| EAT Lancet diet (2) | EAT Lancet diet (1) |  |
| Planetary health diet (8) | Planetary health diet (7) |  |
| Reference diet (9) | Reference diet (8) |  |
| Planetary diet (4) | Planetary diet (3) |  |
| Lancet diet (2) | Lancet diet (1) |  |
|  | Sum of clinical trials | 48 |
|  | Trials without duplicated | 19 |

**Appendix 2 Inclusion criteria**

**Table S2. Inclusion criteria for each outcome of interest**

| **Outcome/dependent variable** | **Definition of outcome** | **Exposure/intervention/independent variable** | **Study diet** | **Comparator** |
| --- | --- | --- | --- | --- |
| Population health | Population health includes physical health and mental health. Any human medical disease or condition is considered a population health outcome | EAT-Lancet planetary health diet | EAT-Lancet planetary health diet or higher adherence to the EAT-Lancet planetary health diet | National dietary guideline or  current dietary pattern or  lower adherence to the EAT-Lancet planetary health diet |
| Environmental sustainability | Planetary health includes environmental and eco­system health. Any environmental indicator is considered planetary health outcome | EAT-Lancet planetary health diet | EAT-Lancet planetary health diet or higher adherence to the EAT-Lancet planetary health diet | National dietary guideline or  current dietary pattern or  lower adherence to the EAT-Lancet planetary health diet |
| Safety (nutrient intakes and deficiencies) | Nutrient intakes and deficiencies indicate differences in intakes of macronutrients or micronutrients from the EAT-Lancet planetary diet vs the national dietary guidelines | EAT-Lancet planetary health diet | EAT-Lancet planetary health diet or higher adherence to the EAT-Lancet planetary health diet | National dietary guideline or  lower adherence to the EAT-Lancet planetary health diet |
| Affordability | Affordability is the cost of the EAT-Lancet planetary health diet per person or per household | EAT-Lancet planetary health diet | EAT-Lancet planetary health diet or higher adherence to the EAT-Lancet planetary health diet | National dietary guideline or  current dietary pattern or  lower adherence to the EAT-Lancet planetary health diet |
| Gaps between current diets and the EAT-Lancet planetary health diet | Gaps between the current diet and the EAT-Lancet planetary health diet indicate differences in food composition or nutrients of the EAT-Lancet planetary health diet versus the diet recommended by current dietary guidelines; or the proportion of individuals follow the EAT-Lancet planetary health diet in a country/area | EAT-Lancet planetary health diet | EAT-Lancet planetary health diet | Current dietary pattern |

**Appendix 3 Detailed characteristics of all included studies**

**Table S3. Summary of articles investigating the EAT-lancet planetary health diet and population health**

| **Study** | **Country** | **Baseline  assessment  period** | **Mean or median follow up  duration** | **Study type** | **Cohort size, n** | **Study subject** | **Study diet** | **Compared diet** | **Dietary assessment method** | **Outcomes** | **Adjustments** | **Main findings** | **Reference** |
| --- | --- | --- | --- | --- | --- | --- | --- | --- | --- | --- | --- | --- | --- |
| **Studies included in the systematic review and meta-analysis** | | | | | | | | | | | | |  |
| Knuppel 2019 | UK | 1993–2001 | 23.6 years | Prospective cohort study | 46,069 | General population/adults, females and males aged ≥ 20 years | Relatively higher 1/4 EAT-Lancet score, Q1–Q4 (4–9, 10, 11, and 12–14) | Relatively lower 1/4 EAT-Lancet score | European Prospective Investigation into Cancer and Nutrition-Oxford FFQ | Ischaemic heart disease, stroke, diabetes, and all-cause mortality (adjusted HR) | Age, total energy intake, education, Townsend deprivation index, alcohol intake, physical activity, smoking, in women, hormone replacement therapy uses and oral contraceptive use, and BMI | Higher adherence to the EAT-Lancet score was - associated with  28% lower risk of ischaemic heart disease; 59% lower risk of diabetes  - not associated with the risk of stroke  - not clearly associated with total mortality | [1] |
| Cacau 2021 | Brazil | 2008 | - | Cross-sectional analysis from cohort | 14,515 | General population/adults, females and males, aged 34–59 and ≥ 60 years | Relatively higher 1/5 EAT-Lancet diet score, Q1–Q5 (23.7–50.4, 50.5–56.9, 57.0–62.9, 63.0–69.9, and 70.0–109.9) | Relatively lower 1/5 EAT-Lancet diet score | A 114-item FFQ | BMI and waist circumference [adjusted β value (95% CIs)] overweight or obese (adjusted OR) | Age, sex, self-reported race, per capita family income, smoking, sporadic alcohol intake, diabetes, hypertension, dyslipidaemia total energy intake, and dietary changes | Higher adherence to the EAT-Lancet diet score was - associated with decrease obesity indicators 1) BMI (β = −0.50, 95% CI: –0.73 to –0.27); 2) waist circumference (β = −1.70, 95%CI: –2.28 to –1.12); 3) 24% less likely to be overweight (OR Q5 vs. Q1: 0.76, 95% CI: 0.67 to 0.85); 4) 24% less likely to be obesity (OR Q5 vs. Q1: 0.76, 95% CI: 0.65 to 0.88); 5) 14% less likely to have increased waist circumference (OR Q5 vs. Q1:0.86, 95% CI: 0.75 to 0.9); 6) 27% less likely to have substantially increased waist circumference (OR Q5 vs. Q1: 0.73, 95% CI: 0.64 to 0.83) | [2] |
| Berthy 2022 | France | 2009–2021 (ongoing) | 8.1 years | Prospective cohort study | 62,382 | General population/adults, females aged 46.6 (SD 25.8) years, males aged 41.7 (SD 24.8) years | Relatively higher 1/5 EAT-Lancet diet score, Q1–Q5 [mean 12.2 (SD 16.3), mean 34.7 (SD 3.7), mean 46.1 (SD 3.1), mean 58.0 (SD 3.9), and mean 81.9 (SD 17.9)] | Relatively lower 1/5 EAT-Lancet score | 24-h dietary recalls  (3 non-consecutive records) | Cancers and CVD (adjusted HR) | For all diseases: Age, sex, energy intake, education level, occupation, monthly household income, marital status, number of completed 24-h dietary recalls, physical activity, smoking status, alcohol consumption, height, family history of chronic disease, and BMI  - Colorectal, lung and prostate cancers are specific to:  Family history of cancer - Breast cancer is specific to:  Number of biological children, menopausal status, oral contraception and hormonal treatment of menopause - CVD is specific to:  Family history of diabetes, family history of stroke, baseline history of diabetes and history of hypertension | Higher adherence to the EAT-Lancet score was - associated with a lower risk of overall cancer among females (HR Q5 vs. Q1: 0.89, 95% CI: 0.75 to 1.05)  - not associated with a reduction in the risk of cancer and CVD, either combined or separately, in multivariable models. Alcohol consumption modified this association, with a significant reduction in risk observed among low drinkers (HR Q5 vs. Q1: 0.86, 95% CI: 0.73 to 1.02)  *both associations were largely attenuated by BMI | [3] |
| Cacau 2022 | Brazil | 2008 | - | Cross-sectional analysis from cohort | 14,155 | General population/adults, females and males, aged 35–74 years | Relatively higher 1/5 EAT-Lancet diet score, Q1–Q5 (range from 0– 150) | Relatively lower 1/5 EAT-Lancet diet score | A 114-item FFQ | Systolic blood pressure, diastolic blood pressure, total cholesterol, LDL-c, and non-HDL-c [adjusted β value (95% CIs)] | Age, sex, self-reported race, per capita income, smoking, alcohol consumption, physical activity level, and total daily energy intake | Higher adherence to the EAT-Lancet diet was - associated with lower levels of blood pres–sure, total cholesterol, LDL-c, and non-HDL-c 1) systolic blood pressure (β = −0.84, 95% CI: −1.66 to −0.01); 2) diastolic blood pressure (β = −0.70, 95% CI: −1.24 to −0.15); 3) total cholesterol (β = −3.15, 95% CI: −5.30 to −1.01); 4) LDL-C (β = −4.10, 95% CI: −5.97 to −2.23); 5) non-HDL-c (β = −2.57, 95% CI: −4.62 to −0.52)  - not associated with HDL-c, triglycerides and HOMA-IR | [4] |
| Ibsen 2022 | Denmark | 1993–1997 | 15 years | Prospective cohort study | 55,016 | General population/adults, females and males aged 50–64 years | Relatively higher 1/5 EAT-Lancet diet score, Q1–Q5 (0–7, 8, 9, 10, and 11–14) | Relatively lower 1/5 EAT-Lancet score | A 192-item semi-quantitative FFQ | Stroke subtypes (adjusted HR) | Age, sex, BMI, education, smoking status, physical activity, alcohol intake, use of hormone replacement therapy (in woman), waist circumference, history of hypertension, hypercholesterolemia, diabetes, and myocardial infarction | Higher adherence to the EAT-Lancet diet in midlife was - associated with a lower risk of stroke subtype, specifically subarachnoid hemorrhage (HR: 0.30, 95% CI: 0.12 to 0.73) | [5] |
| Lazarova 2022 | UK | 2004 and 2015 | - | Prospective cohort | 6,771 | General population/adults, females and males  – Canadian Community Health Survey– Nutrition 2004 cycle exclusion criteria: individuals <45 years and >80 years (for survival models) and pregnant and breastfeeding women  – Canadian Community Health Survey– Nutrition 2015 cycle exclusion criteria: individuals < 18 years and breastfeeding women | Relatively higher 1/4 EAT-Lancet score, Q1–Q4 [6.32, 8, 9, and 10 (median)] | Relatively lower 1/4 EAT-Lancet score | Two 24-h recalls | CVD incidence and mortality | Age, sex, dietary scores, lifestyle, sociodemographic characteristics, | Adherence to the EAT-Lancet diet was - not associated with CVD risk | [6] |
| Marchioni 2022 | Brazil | 2017–2018 | - | Cross-sectional study | 46,164 | General population/adults, females and males, aged ≥ 10 years | Relatively higher 1/4 EAT-Lancet score (range from 0– 140) | Relatively lower 1/4 EAT-Lancet score | Two 24-h recalls | Overweight/obesity | Age, sex, per capita income, and daily total energy intake | Higher adherence to the EAT-Lancet diet was - associated with a higher risk of obesity and being overweight (Q4 VS. Q1 OR: 1.002, 95% CI: 0.999 to 1.004) | [7] |
| Stubbendorff 2022 | Sweden | 1991–1996 | 20 years | Population-based prospective cohort study | 22,421 | General population/adults, females and males aged 45–73 years | Relatively higher 1/5 EAT-Lancet score, Q1–Q5 (≤ 13, 14–16, 17–19, 20–22, and ≥ 23) | Relatively lower 1/5 EAT-Lancet score | Assessment consists of 3 parts: 1) 7-day (consecutive days) food diary  2) 168-item FFQ 3) 60-minute interview | All-cause mortality, cancer mortality, and cardiovascular mortality (adjusted HR) | Age, sex, dietary assessment version, season, energy intake, leisure-time physical activity, smoking habits, alcohol consumption, educational level, and BMI | Those with the highest adherence to the EAT-Lancet diet was - associated with a 25% lower risk of mortality | [8] |
| Vallejo 2022 | Germany | 1985-2021 (ongoing) | 3 yearsi | Prospective cohort study | 298 | Young adults and adolescents, females and males aged ≥ 15 years | Relatively higher 1/3 EAT-Lancet diet score, male T1–T3 [median 9 range (7–9), median 10 range (10–10), and median 11 range (11–14)], female T1–T3 [median 9 range (6–10), median 11 range (11–11), and median 12 range (12–15)] | Relatively lower 1/3 EAT-Lancet score | 3-d weighted dietary record (2-5 sets) | Body weight, BMI, fat–free mass index, waist circumference, and body fat rate [adjusted mean value (95% CI) and β value (95% CI)] | Age, sex, total energy intake, follow-up time, birth weight, pregnancy duration, parental school and higher education, and participant school and higher education | Higher adherence to the EAT-Lancet diet was - inversely associated with anthropometric markers (body weight, BMI, fat-free mass index, waist circumference, and body fat rate) during young adulthood | [9] |
| Xu 2022 | UK | 2006–2010 | 10 years | Prospective cohort study | 59,849 | General population/adults, females and males aged 55.9 (SD 8.1) years | Relatively higher 1/3 EAT-Lancet diet score, T1–T3 (45.99% of total numbers of participants, 25.38% of total numbers of participants, and 28.63% of total numbers of participants) | Relatively lower 1/3 EAT-Lancet score | 24-h dietary recalls  (at least once) | T2D (adjusted HR) BMI and waist circumference [adjusted β value (95% CI)] | Age, sex, Townsend deprivation index, qualifications, and ethnicity. smoking, drinking, physical activity energy, BMI, and waist circumference | Higher adherence to EAT-Lancet diet was - associated with a lower risk of T2D. A one-point increase in the diet score were associated with a 6% decrease in risk of T2D (HR: 0.94, 95% CI: 0.91 to 0.97) | [10] |
| Cacau 2023 | 10 European countries | 2006–2007 | - | Cross-sectional study | 637 | General population/adolescents, females and males, aged 12.5–17.5 years | Relatively higher EAT-Lancet diet score (range from 0–150) | Relatively lower EAT-Lancet diet score | 24-h dietary recalls & The German Food Code and Nutrient Database (Bundeslebensmittelschlüssel, vII.3.1, Karlsruhe, Germany) | Non-ideal cardiovascular health status, high blood pressure, high blood cholesterol (adjusted OR) | Age, sex, socio-economic status, and total energy intake | Higher adherence to the EAT-Lancet diet score was - associated with a better cardiovascular health status among European adolescents 1) lower probability of a non-ideal cardiovascular health status (OR: 0.84, 95% CI: 0.75 to 0.94) 2) lower probability of high blood pressure (OR: 0.87, 95% CI: 0.79 to 0.96) 3) lower probability of high blood cholesterol (OR: 0.88, 95% CI: 0.78 to 0.99) | [11] |
| Colizz 2023 | New Zealand | 1993–1997 | 15.1 years | Prospective cohort study | 35,496 | General population/adults, females and males aged 20–70 years | Relatively higher 1/4 EAT-Lancet diet score, Q1–Q4 (32–66, 67–73, 74–79, and 80–117) | Relatively lower 1/4 EAT-Lancet score | A 178-item semi-quantitative FFQ | CVD, coronary heart disease, and stroke subtypes (adjusted HR) | Age, sex, educational level, smoking status, alcohol consumption, physical activity, energy intake, BMI, hypertension, and total cholesterol levels | High adherence to the EAT-Lancet diet was - associated with a lower risk of  CVD (HR Q4 vs. Q1: 0.86, 95% CI: 0.78 to 0.94);  coronary heart disease (HR Q4 vs. Q1: 0.88, 95% CI: 0.78 to 1.00) - associated with 11% lower risks of total stroke (HR Q4 vs. Q1: 0.89, 95% CI: 0.72 to 1.10) | [12] |
| Karavasiloglou 2023 | UK | 2006–2010 | 11.5 years | Population-based prospective cohort study | 473,836 | General population/adults, females and males aged 56.50 (SD 8.08) years | Relatively higher 1/3 EAT-Lancet diet score, T1–T3 (0–4, 5–7, and 8–11) | Relatively lower 1/3 EAT-Lancet score | UK Biobank Touchscreen questionnaire | Cancers (all incident cancer diagnoses, breast cancer, colorectal cancer, and prostate cancer), cardiovascular events (incident major cardiovascular events, total stroke, Ischemic stroke, hemorrhagic stroke, myocardial infarction), and all-cause mortality (adjusted HR) | For all diseases: age, sex, and region plus further adjustment for smoking status, BMI, physical activity, highest level of attained education, Townsend deprivation index, and alcohol intake status  - Major cardiovascular events and stroke subtypes are specific to:  first-degree family history for heart disease and stroke | Higher adherence to the EAT-Lancet diet was - associated with a lower risk of  cancer (HR T3 VS. T1: 0.99, 95% CI: 0.98 to 0.99);  all-cause mortality (HR T3 VS. T1: 0.98, 95% CI: 0.98 to 0.99)  - not associated with major cardiovascular events (HR T3 VS. T1: 1.00, 95% CI: 0.98 to 1.01) | [13] |
| Langmann 2023 | Denmark | 1993–1997 | 15 years | Prospective cohort study | 54,232 | General population/adults, females and males aged 50–64 years | Relatively higher 1/5 EAT-Lancet diet score, Q1–Q5 (0–7, 8, 9, 10, and 11–14) | Relatively lower 1/5 EAT-Lancet score | A 192-item self- administered FFQ | T2D (adjusted HR) | Age, physical activity, education, smoking status, alcohol intake, sex, history of hypertension, history of hypercholesterolemia, waist circumference, BMI, and energy intake | Higher adherence to the EAT-Lancet diet was - associated with a lower risk of developing T2D (HR Q5 VS. Q1: 0.78, 95% CI 0.71 to 0.86) in a middle-aged Danish population | [14] |
| Langmann 2023 | Denmark | 1993–1997 | 5 years | Prospective cohort study | 44,194 | General population/adults, females and males aged 50–64 years | Relatively higher 1/5 EAT-Lancet diet score, Q1–Q5 (0–7, 8, 9, 10, and 11–14) | Relatively lower 1/5 EAT-Lancet score | A 192-item self- administered FFQ | Obesity and waist circumference (adjusted RR) body weight and waist circumference [adjusted β value (95% CI)] | Obesity and elevated waist circumference: sex, age at inclusion, physical activity, education, smoking status, alcohol intake, previous history of hypertension, hypercholesterolemia, diabetes, stroke, and acute myocardial infarction before baseline  body weight and waist circumference: baseline measures of body weight or waist circumference respectively, sex, age at inclusion, physical activity, education, smoking status, alcohol intake, previous history of hypertension, hypercholesterolemia, diabetes, stroke, acute myocardial infarction before baseline, and energy intake | Higher adherence to the EAT-Lancet diet was - associated with lower  follow-up waist circumference (β = –0.38, 95% CI: –0.69 to 0.07); - associated with reduced risk of  obesity (RR Q5 VS. Q1: 0.89, 95% CI: 0.82 to 0.98);  elevated waist circumference (RR Q5 VS. Q1: 0.95, 95% CI: 0.93 to 0.96)  - not associated with follow-up weight (β = –0.08, 95% CI: –0.27 to 0.1) | [15] |
| López 2023 | Mexico | 2006–2013 (ongoing) | 2.2 years | Prospective cohort study | 115,314 | Female teachers in public school, aged ≥ 35 years | Relatively higher 1/4 EAT-Lancet diet score, Q1–Q4 (0–4, 5–6, 7–8, and 9–13) | Relatively lower 1/4 EAT-Lancet score | A 140-item FFQ | T2D (adjusted HR) | Age, total energy intake, socioeconomic status score, family history of diabetes, region, smoking status, physical activity, menopausal status, and alcohol intake | Higher adherence to the EAT-Lancet diet was - associated with a lower risk of T2D incidence among women (HR Q4 VS. Q1: 0.90, 95% CI: 0.75 to 1.10) in a middle-income country | [16] |
| Quarpong 2023 | Kenya | 2014 | - | Cross-sectional study | 242 | Lactating mothers, aged 16–44 years | Relatively higher 1/2 EAT-Lancet diet score (0–2 and 3–5) | Relatively lower 1/2 EAT-Lancet diet score | A single quantitative 24 h recall | BMI (adjusted OR) | Age of lactating mother, number of months post‐partum and household hunger | Adherence to the EAT‐Lancet diet was - not associated with BMI | [17] |
| Ren 2023 | USA | 1993–2001 | 8.8 years | Prospective cohort study | 98,415 | General population/adults, females and males aged 65.5 (SD 5.7) years | Relatively higher 1/4 EAT-Lancet diet score, Q1–Q4 (≤ 18, 19–21, 22–24, and ≥ 25) | Relatively lower 1/4 EAT-Lancet score | A 124-item FFQ | Colorectal cancer (adjusted HR) | Age, sex, race, BMI, smoking status, pack–year of smoking, drinking status, physical activity level, aspirin and ibuprofen consumption, family history of colorectal cancer, history of diverticulitis, colorectal polyps, colon comorbidities, energy intake from diet, protein intake from diet, carbohydrate intake from diet, and fat intake from diet | Higher adherence to EAT-Lancet diet was - associated with reduced incidence of colorectal cancer (HR Q4 vs. Q1: 0.81, 95% CI: 0.67 to 0.98) | [18] |
| Xiao 2023 | USA | 1993–2001 | Lung cancer 8.8 years, lung cancer mortality 15.1 years | Prospective cohort study | 98,415 | General population/adults, females and males aged 55–74 or 65.5  (SD 5.7) years | Relatively higher 1/4 EAT-Lancet diet score, Q1–Q4 (4–9, 10, 11, and 12–13) | Relatively lower 1/4 EAT-Lancet score | A 137-item self- administered FFQ | Lung cancer and lung cancer mortality (adjusted HR) | Age, sex, race, education levels, occupation situation, BMI at baseline, BMI at age 20, weight change, trail arm, smoking status, daily cigarette consumption, history of drinking alcohol, alcohol consumption, aspirin use, family history of lung cancer, history of hypertension, history of diabetes, history of chronic bronchitis, history of emphysema, total energy intake, and physical activity level | Higher adherence to EAT-Lancet diet was - associated with a lower risk of  lung cancer (HR Q4 VS. Q1: 0.73, 95% CI: 0.60 to 0.89);  mortality of lung cancer (HR Q4 VS. Q1: 0.74, 95% CI: 0.59 to 0.9) | [19] |
| Ye 2023 | Singapore | 1993–1998 | 23.4 years | Population-based prospective cohort study | 63,257 | General population/adults, females and males aged 45–74 years | Relatively higher 1/5 EAT-Lancet diet score, Q1–Q5 (13–47, 47–53, 53–58, 58–63, and 63–95) | Relatively lower 1/5 EAT-Lancet diet score | A 165-item face-to-face semi-quantitative FFQ | All-cause mortality, CVDs mortality, cancer mortality, and respiratory diseases mortality (adjusted HR) | Age, sex, energy intake, dialect group, BMI, smoking status, alcohol frequency, physical activity, sleep duration, and self-reported history of physician–diagnosed hypertension and diabetes | Higher adherence to EAT-Lancet diet was - associated with lower risk of  all-cause mortality (HR Q5 VS. Q1: 0.85, 95% CI: 0.81 to 0.89);  CVD mortality (HR Q5 VS. Q1: 0.79, 95% CI: 0.73 to 0.85);  cancer mortality (HR Q5 VS. Q1: 0.93, 95% CI: 0.86 to 1.00);  respiratory disease mortality (HR Q5 VS. Q1: 0.81, 95% CI: 0.74 to 0.89) | [20] |
| Zhang 2023 | Sweden | 1991–1996 | 24.9 years | Population-based prospective cohort study | 23,877 | General population/adults, females and males aged 57.9 (SD 7.7) years | Relatively higher 1/5 EAT-Lancet diet score, Q1–Q5 (≤ 13, 14–16, 17–19, 20–22, and ≥23) | Relatively lower 1/5 EAT-Lancet diet score | Assessment consists of 3 parts: 1) 7-day (consecutive days) food diary  2) a 168-item FFQ 3) 60-minute interview | Coronary events | Age, sex, dietary assessment version, season, total energy intake, leisure–time physical activity, alcohol consumption, smoking status, educational level, and BMI | Higher adherence to EAT-Lancet diet was - associated with a lower risk of coronary events (HR Q5 VS. Q1: 0.80, 95% CI: 0.67 to 0.96) | [21] |
| Zhang 2023 | Sweden | 1991–1996 | 22.9 years | Population-based prospective cohort study | 24,713 | General population/adults, females and males aged 58.0 (SD 7.7) years | Relatively higher 1/5 EAT-Lancet diet score, Q1–Q5 (≤ 13, 14–16, 17–19, 20–22, and ≥ 23) | Relatively lower 1/5 EAT-Lancet diet score | Assessment consists of 3 parts: 1) 7-day (consecutive days) food diary  2) a 168-item FFQ 3) 60-minute interview | Atrial fibrillation (adjusted HR) | Age, sex, dietary assessment version, season, total energy intake, leisure time physical activity, alcohol consumption, smoking status, educational level, BMI, diabetes, hypertension, and lipid- lower ing medication | Higher adherence to the EAT-Lancet score was - associated with 18% lower risk of atrial fibrillation. (HR Q5 VS. Q1: 0.84, 95% CI: 0.73 to 0.98) | [22] |
| Zhang 2023 | Sweden | 1991–1996 | 24.3 years | Population-based prospective cohort study | 24,494 | General population/adults, females and males aged 58.1 (SD 7.7) years | Relatively higher 1/5 EAT-Lancet diet score, Q1–Q5 (≤ 13, 14–16, 17–19, 20–22, and ≥ 23) | Relatively lower 1/5 EAT-Lancet diet score | Assessment consists of 3 parts: 1) 7-day (consecutive days) food diary  2) a 168-item FFQ 3) 60-minute interview | T2D (adjusted HR) | Age, sex, dietary assessment version, season, total energy intake, leisure–time physical activity, alcohol consumption, smoking status, educational level, family history of diabetes, lipid- lowering medication, hypertension at baseline, history of CVD and cancer, and BMI | Higher adherence to the EAT-Lancet diet was - associated with a lower risk of T2D (HR Q5 VS. Q1: 0.82, 95% CI: 0.70 to 0.96) | [23] |
| Bui 2024 | USA | 1986 and 1991 | 34 years | Population-based prospective cohort study | 206,404 | General population/adults, females and males, aged 25–75 years | Relatively higher 1/5 EAT-Lancet diet score, Q1–Q5 (58–66, 67–74, 73–80, 78–86, and 86–97) | Relatively lower 1/5 EAT-Lancet diet score | ˃ 130-item self-administered FFQs | All-cause mortality, CVD mortality, cancer mortality, respiratory disease mortality, neurodegenerative disease mortality, and infectious disease mortality (adjusted HR) | Age in months, follow-up cycles, race, marriage status, living status, neighborhood socioeconomic status z-score, menopausal status, multivitamin use, aspirin use, total energy intake, baseline BMI, smoking status, alcohol drinking, physical activity, history of hypertension, history of hypercholesterolemia, family history of myocardial infarction, family history of diabetes, and family history of cancer | Higher adherence to the EAT-Lancet diet was  - associated with a lower risk of  all-cause mortality (HR Q5 VS. Q1: 0.77, 95% CI: 0.75 to 0.80);  CVD mortality (HR Q5 VS. Q1: 0.86, 95% CI: 0.81 to 0.91);  cancer mortality (HR Q5 VS. Q1: 0.90, 95% CI: 0.85 to 0.95);  respiratory disease mortality (HR Q5 VS. Q1: 0.53, 95% CI: 0.48 to 0.59);  neurodegenerative disease mortality (HR Q5 VS. Q1: 0.72, 95% CI: 0.67 to 0.78);  infectious disease mortality (HR Q5 VS. Q1: 0.78, 95% CI: 0.63 to 0.98) | [24] |
| Cai 2024 | China | 1997–2015 | 9.86 years (median) | Prospective cohort study | 16,029 | General population/adults, females and males aged 18–65 years | Relatively higher 1/4 EAT-Lancet diet score, Q1–Q4 (9.42–50.03, 47.5–59.33, 54.21–68.26, and 62.54–110.89) | Relatively lower 1/4 EAT-Lancet diet score | 3-day 24-hour recall | All-cause mortality, CVD, and T2D (adjusted HR) | Age, sex, dietary energy, educational level, physical activity, household income, smoking status, and alcohol consumption | Higher adherence to EAT-Lancet diet was - associated with a lower risk of  T2D (HR Q4 VS. Q1: 0.35, 95% CI: 0.28 to 0.44);  CVD (HR Q4 VS. Q1: 0.49, 95% CI: 0.37 to 0.66)  - not associated with a lower risk of  all-cause mortality (HR Q4 VS. Q1: 0.85, 95% CI: 0.67 to 1.07) | [25] |
| Chen 2024 | China | 1997, 2000, 2004, 2006, 2009, and 2011 | 2 years | Prospective cohort study | 9,364 | General population/adults, females and males, aged ≥ 45 years | Relatively higher 1/4 EAT-Lancet score, Q1–Q4 (median 44, median 50, median 55, median 62) | Relatively lower 1/4 EAT-Lancet score | 3 day 24-h dietary recalls | Mortality (adjusted HR) | Age, age-square, sex, residence, education, household  income per capita, total energy intake, smoking status, alcohol intake, physical activity, hypertension, and diabetes | Higher adherence to the EAT-Lancet score was  - associated with a lower risk of mortality (HR Q4 VS. Q1: 0.79, 95% CI: 0.63 to 0.99) | [26] |
| de Oliveira Neta 2024 | Brazil | 2019–2020 | - | Cross-sectional study | 398 | Noninstitutionalized adults and elderly people, female and male | Relatively higher EAT-Lancet diet score (range 0–150) | Relatively lower EAT-Lancet diet score | A 24-hour dietary recalls | Diastolic blood pressure, systolic blood pressure, fasting blood glucose, total cholesterol, LDL-c, HDL-c, triglycerides, diabetes mellitus, dyslipidemia, and arterial hypertension | Age, income, and BMI | Higher adherence to the EAT-Lancet diet was  - associated with  higher diastolic blood pressure (β: 0.46, 95% CI: 0.02 to 0.90);  lower systolic blood pressure (β: –0.14, 95% CI: –0.25 to –0.01);  lower total cholesterol (β: –0.80, 95% CI: –1.31 to –0.46);  lower LDL-c (β: –0.10, 95% CI: –0.19 to –0.08);  - associated with a lower risk of diabetes mellitus (β: –5.58, 95% CI: –8.20 to –0.95);  lower risk of dyslipidemia (β: –4.19, 95% CI: –8.48 to –0.09)  - not associated with  fasting blood glucose (β: 0.12, 95% CI: –0.26 to 0.49);  HDL-c (β: –0.02, 95% CI: –0.45 to 0.49);  triglycerides (β: –0.21, 95% CI: –0.55 to 0.14);  lower risk of arterial hypertension (β: –1.63, 95% CI: –6.00 to 2.78) | [27] |
| Frank 2024 | USA | 2015–2018 | - | Cross-sectional study | 8,128 | Civilian, non-institutionalized population/adults, females and males, aged ≥ 60 years | Relatively higher 1/5 EAT-Lancet diet score, Q1–Q5 (18.5–54.0, 54.5–62.0, 62.5–69.0, 69.5–77.0, and 77.5–125.0) | Relatively lower 1/5 EAT-Lancet diet score | A 24-h dietary recall | Waist circumference, blood pressure, HDL-c, fasting plasma glucose and triglycerides [difference (95% CI)] | Age, sex, income, education, race/ethnicity, and total energy intake | Higher adherence to the EAT-Lancet diet was  - associated with lower predicted probability of cardiometabolic risk 1) low HDL-c (mean differences Q5 VS. Q1: 36.6%, 95% CI: 34.1 to 39.1%); 2) high fasting plasma glucose (mean differences Q5 VS. Q1: 62.4%, 95% CI: 59.8 to 65.0%) | [28] |
| Gonçalves 2024 | Brazil | 2008–2010 | 9 years | Population-based prospective cohort study | 11,737 | General population/adults, females and males, aged 35–74 years | Relatively higher 1/5 EAT-Lancet diet score, Q1–Q5 (26.2–50.5, 50.5–57.0, 57.0–63.0, 63.0–69.9, and 69.9–110.0) | Relatively lower 1/5 EAT-Lancet diet score | A 114-item FFQ | Memory, verbal fluency, executive function, and global cognition | Age, sex, education, income, race, alcohol consumption, smoking, diabetes, hypertension, CVD, physical activity, depressive symptoms, BMI and total calories | Higher adherence to the EAT-Lancet diet was  - not associated with  slower decline of memory (β = 0.000, 95% CI: 0.000-0.001);  verbal fluency (β = 0.000, 95% CI: –0.000 to 0.001);  executive function (β = -0.001 95% CI: –0.002 to 0.000);  global cognition (β = 0.000, 95% CI: 0.000 to 0.001) | [29] |
| Guzmán-Castellanos 2024 | Spain | 1999 | 11.5 years | Prospective cohort study | 18,656 | General population/adults, females and males aged ≥ 20 years | Relatively higher 1/4 EAT-Lancet diet score, Q1–Q4 (7–18, 19–21, 22–23, and 24–37) | Relatively lower 1/4 EAT-Lancet diet score | A self-administered 136-item FFQ | CVD (adjusted HR) | Age, sex, total energy intake, educational level, alcohol intake, accumulated smoking habit, physical activity, BMI, snacking between  meals, watching television, time spent sitting, following a special diet at baseline, hypertension, family history  of CVD, any diagnosis of diabetes, hypercholesterolemia, depression,  dyslipidemia, and cancer | Higher adherence to EAT-Lancet diet was not associated with a lower risk of CVD (HR Q4 VS. Q1: 0.77, 95% CI: 0.51–1.18) | [30] |
| Huang 2024 | USA | 2005–2018 | - | Cross sectional study | 32,388 | General population/adults, females and males aged ≥ 20 years (Pregnant women excluded) | Relatively higher 1/4 EAT-Lancet score (range from 0– 140) | Relatively lower 1/4 EAT-Lancet score | FFQ from database | Asthma (adjusted OR) and BMI [adjusted β value (95% CI)] | Age, gender, education level, marital, family povertyto-income ratio, race, smoking, drinking, energy intake, hypertension, diabetes, and high cholesterol | Higher adherence to the EAT-Lancet score was - associated with  lower asthma  (OR Q4 VS. Q1: 0.86, 95% CI: 0.75 to 0.98);  lower BMI (β = –0.46, 95% CI: –0.73 to –0.56) | [31] |
| Kamrani 2024 | Iran | 2017–2020 | - | Cross-sectional study | 4,579 | General population/adults, females and males aged 30–70 years | Relatively higher 1/4 EAT-Lancet diet score, Q1–Q4 [mean 83.3 (SD 6.4), mean 97.6 (SD 3.6), mean 110.6 (SD 4.0), and mean 113.2 (SD 13.9)] | Relatively lower 1/4 EAT-Lancet score | A 118-item semi-quantitative FFQ | Depression, anxiety, and stress (adjusted OR) | Age, sex, BMI, physical activity, education level, smoking, alcohol, drugs, residence type, ethnic, family history of psychiatric disorder, and energy intake | Higher adherence to EAT-Lancet diet was - associated with a lower risk of  depression (OR Q4 VS. Q1: 0.65, 95% CI: 0.48 to 0.88);  anxiety (OR Q4 VS. Q1: 0.81, 95% CI: 0.62 to 1.07);  stress (OR Q4 VS. Q1: 0.67, 95% CI: 0.46 to 0.99) | [32] |
| Klapp 2024 | USA | 1993–1996 | 10 years | Population-based prospective cohort study | 193,379 | General population/adults, females and males, aged around 60 years | Relatively higher 1/3 EAT-Lancet diet score, T1–T3 (9–23, 24–26, and 27–42) | Relatively lower 1/3 EAT-Lancet diet score | A semi-quantitative FFQ 24-h dietary recall | Obesity and T2D (adjusted HR) | Follow-up (time metric), sex, age, education, smoking status, physical activity, alcohol intake, and ethnicity | Higher adherence to the EAT-Lancet diet was  - associated with a lower risk of  obesity (HR T3 VS. T1: 0.86, 95% CI: 0.83 to 0.90);  T2D (HR T3 VS. T1: 0.97, 95% CI: 0.94 to 0.99) | [33] |
| Li 2024 | UK | 2006–2010 | 13.7 years | Population-based prospective cohort study | 459,502 | General population/adults, females and males, aged 40–69 years | Relatively higher 1/5 EAT-Lancet diet score, Q1–Q5 (0–2.5, 2.5–3, 3–3.67, 3.67–4.25, and 4.25–9) | Relatively lower 1/5 EAT-Lancet diet score | 24-h dietary recalls | Chronic liver disease, severe liver disease, liver-related death, liver cancer, hepatocellular carcinoma, and intrahepatic cholangiocarcinoma (adjusted HR) | Recruitment age, sex, race, Townsend deprivation index, smoking and drinking status, educational attainment, physical activity, and family history of cancer | Higher adherence to the EAT-Lancet diet was  - associated with a lower risk of  chronic liver disease (HR Q5 VS. Q1: 0.62, 95% CI: 0.47 to 0.81);  severe liver disease (HR Q5 VS. Q1: 0.67, 95% CI: 0.53 to 0.85);  - not associated with a lower risk of  liver-related death (HR Q5 VS. Q1: 0.82, 95% CI: 0.56 to 1.20);  liver cancer (HR Q5 VS. Q1: 0.86, 95% CI: 0.59 to 1.26);  hepatocellular carcinoma (HR Q5 VS. Q1: 0.92, 95% CI: 0.49 to 1.72);  intrahepatic cholangiocarcinoma (HR Q5 VS. Q1: 0.77, 95% CI: 0.44 to 1.35) | [34] |
| Liu 2024 | China | 2006–2010 | 9.5 years | Prospective cohort study | 175,214 | General population/adults, females and males aged 37–73 years | Relatively higher 1/5 EAT-Lancet diet score, Q1–Q5 (≤ 8, = 9, = 10, = 11, and ≥ 12) | Relatively lower 1/5 EAT-Lancet diet score | A 24-h dietary recall | Lung cancer incidence, lung cancer mortality (adjusted HR) | Age, sex, race, Townsend deprivation index, total energy intake, smoking status, pack-years of smoking, alcohol intake, physical activity, BMI, polygenic risk score for lung cancer, first 10 principal components of ancestry, and genotype measurement batch | Higher adherence to the EAT-Lancet score was  - associated with a lower risk of  lung cancer incidence (HR Q5 VS. Q1: 0.64, 95% CI: 0.51 to 0.80);  lung cancer mortality (HR Q5 VS. Q1: 0.65, 95% CI: 0.48 to 0.88) | [35] |
| Lu 2024 | UK | 2006–2010 | 11.6 years | Population-based prospective cohort study | 180,446 | General population/adults, females and males, aged 37–73 years | Relatively higher 1/4 EAT-Lancet diet score, Q1–Q4 (≤ 9, = 10, = 11, and ≥ 12) | Relatively lower 1/4 EAT-Lancet diet score | 24-h dietary recalls | Depression and anxiety | Age, sex, Townsend scores, ethnicity, smoking status, alcohol intake, physical activity, hypertension, BMI, and total energy intake | Higher adherence to the EAT-Lancet diet was  - associated with a lower risk of  depression (HR Q4 VS. Q1: 0.806, 95% CI: 0.730 to 0.890);  anxiety (HR Q4 VS. Q1: 0.818, 95% CI: 0.751 to 0.892) | [36] |
| Masip 2024 | Canada | 2009–2010 | - | Cross-sectional study | 7,037 | General population/adults, females and males aged 40–69 years | Relatively higher EAT-Lancet diet score (range from 0–150) | Relatively lower EAT-Lancet diet score | A 164-item semi-quantitative FFQ | BMI, waist circumference, body fat percentage [adjusted β value (95% CI)] | Age, sex, ethnicity, alcohol intake, smoking, education, sleeping time, income, anxiety,  physical activity, and energy misreporter status | Higher adherence to EAT-Lancet diet was - associated with   lower BMI (β = −0.11, 95% CI: −0.13 to −0.09);  lower waist circumference (β = −0.12, 95% CI: −0.14 to −0.10);  lower body fat percentage (β = −0.10, 95% CI: −0.12 to −0.08) | [37] |
| Morcel 2024 | 9 European countries | 2006–2007 | 11 years | Nested longitudinal cohort study | 236 | Young adolescents, females and males, aged 12.5–17.5 years | Relatively higher EAT-Lancet diet score (range from 0–150) | Relatively lower EAT-Lancet diet score | 24-h dietary recalls (three times) | HDL | Salt intake, eicosapentaenoic acid and docosahexaenoic acid intakes, fructose from processed food intake, diet quality index, healthful plant-based diet index, ultra-processed food consumption, planetary health diet index, nutritional knowledge test,moderate-to-vigorous physical activity, sedentary time, cardiorespiratory ﬁtness, upper body muscular strength by hand-grip test, and lower body explosive strength by standing broad jump | Higher adherence to the EAT-Lancet diet score was - associated with lower HDL (β = 0.21, 95% CI: 0.03 to 0.39) | [38] |
| Pitt 2024 | Sweden | 1987–1997 | 22 years | Population-based prospective cohort study | 68,175 | General population/adults, females and males aged 58–63 years | Relatively higher 1/4 EAT-Lancet diet score, Q1–Q4 (≤ 6, 7–8, 9–10, and ≥ 11) | Relatively lower 1/4 EAT-Lancet score | A 96-item FFQ | All-cause mortality, cardiovascular mortality, and cancer mortality (adjusted HR) | Age at baseline, education level, living alone, smoking status, BMI, walking/cycling, exercise, supplement use, history of hypertension, hypercholesterolemia, energy intake, excluding energy from alcohol, alcohol consumption, moderate drinkers, or heavy drinkers | Higher adherence to the EAT-Lancet diet was - associated with a reduction in risk of all-cause and cardiovascular–mortality  1) men (all-cause mortality): HR Q4 VS. Q1: 0.93, 95% CI: 0.90 to 0.97;  2) women (cardiovascular–mortality): HR Q4 VS. Q1: 0.91, 95% CI: 0.87 to 0.95;  3) men (all-cause mortality): HR Q4 VS. Q1: 0.94, 95% CI: 0.88 to 1.00;  4) women (cardiovascular–mortality): HR Q4 VS. Q1: 0.93, 95% CI: 0.87 to 1.00 | [39] |
| Quartiroli 2024 | Italy | 1987–1992 | 22.6 years | Prospective cohort study | 10,786 | Healthy women, aged 35–69 | Relatively higher 1/3 EAT-Lancet diet score, T1–T3 (4–9, 10, and 11–12) | Relatively lower 1/3 EAT-Lancet diet score | A semi-quantitative FFQ | Breast cancer (adjusted HR)  hs-CRP, TNF-α, IL-6, leptin, and adiponectin [adjusted β value (95% CI)] | Breast cancer: age, nonalcoholic energy intake, age at menarche, menopausal status, parity, age at first birth, smoking status, education, and BMI  hs-CRP, TNF-α, IL-6, leptin, and adiponectin: age, menopausal status, and the time gap between recruitment | Higher adherence to the EAT-Lancet diet was  - associated with  higher hs-CR (β = 1.10, 95% CI: 0.02 to 0.18)  - not associated with  a lower risk of breast cancer (HR Q5 VS. Q1: 1.10, 95% CI: 0.88 to 1.39);  TNF-α (β = 0.03, 95% CI: –0.07 to 0.11);  IL-6 (β = 0.06, 95% CI: –0.20 to 0.07);  leptin (β = 0.00, 95% CI: -0.07 to 0.08);  adiponectin (β = –0.04, 95% CI: –0.1 to 0.02) | [40] |
| Ren 2024 | USA | 1993–2001 | 8.84 years | Population-based prospective cohort study | 101,755 | General population/adults, females and males, aged 55–74 years | Relatively higher 1/4 EAT-Lancet diet score, Q1–Q4 (6–17, 18–21, 22–23, and 24–40) | Relatively lower 1/4 EAT-Lancet diet score | A 124-item FFQ | Head and neck cancers, oral cavity and pharynx cancers, and larynx cancer (adjusted HR) | Age, sex, ethnicity, education levels, family history of head and neck cancers, BMI at baseline, smoking status, pack-years, drink alcohol, diary alcohol intakem, and total energy intake | Higher adherence to the EAT-Lancet diet was  - associated with a lower risk of  head and neck cancers (HR Q4 VS. Q1: 0.52, 95% CI: 0.34 to 0.80);  oral cavity and pharynx cancers (HR Q4 VS. Q1: 0.52, 95% CI: 0.31 to 0.88);  - not associated with a lower risk of larynx cancer (HR Q4 VS. Q1: 0.50, 95% CI: 0.23 to 1.08) | [41] |
| Rodrigues 2024 | Portugal | 2014–2015 | - | Cross sectional study | 660 | School-aged children, aged 7–12 years | Relatively higher 1/4 EAT-Lancet diet score, Q1–Q4 (≤ 25.8, 25.8–31.8, 31.8–41.2, and ˃ 41.2) | Relatively lower 1/4 EAT-Lancet score | A 24-h dietary recall | Airway Inflammation, asthma definitions, Medical diagnosis with asthma symptoms or +BD, Medical diagnosis and under asthma treatment (adjusted OR) | Sex, age, supplementation use, parental education, physical activity, mother smoking during pregnancy, breastfeeding, atopy, and BMI | Higher adherence to the EAT-Lancet score was  - not associated with  airway Inflammation (OR Q4 VS. Q1: 0.53, 95% CI: 0.22 to 1.27);  asthma definitions (OR Q4 VS. Q1: 1.52, 95% CI: 0.47 to 4.96);  medical diagnosis with asthma symptoms or +BD (OR Q4 VS. Q1: 0.84, 95% CI: 0.32 to 2.25);  medical diagnosis and under asthma treatment (OR Q4 VS. Q1: 1.18, 95% CI: 0.31 to 4.48) | [42] |
| Sawicki 2024 | USA | NHS Ⅰ: 1976;  NHS Ⅱ: 1989;  HPFS: 1986 | NHS Ⅰ: 30.0 years;  NHS Ⅱ: 26.0 years;  HPFS: 30.0 years | Prospective cohort study | NHS Ⅰ: 62,919;  NHS Ⅱ: 88,535;  HPFS: 342,164 | NHS Ⅰ: female registered nurses, aged 30–55 years;  NHS Ⅱ: female registered nurses, aged 25–42 years;  HPFS: male health professionals, aged 40–75 | Relatively higher 1/5 EAT-Lancet score (range from 0– 140) | Relatively lower 1/5 EAT-Lancet score | A semi-quantitative FFQ (more than 110 items) | CVD, coronary heart disease, stroke, ischaemic stroke (adjusted HR) | Inverse variance-weighted fixed effects meta-analysis of age-adjusted cohort-specific hazard ratios, energy intake, alcohol, multivitamin use, aspirin use, smoking, physical activity, marital status, family history of T2D, family history of CVD, postmenopausal hormone use, oral contraceptive use, race, hypertension, hypercholesterolaemia, and incident diabetes | Higher adherence to the EAT-Lancet score was  - associated with lower risk of  CVD (HR Q5 VS. Q1: 0.83, 95% CI: 0.78 to 0.89);  coronary heart disease (HR Q5 VS. Q1: 0.81, 95% CI: 0.74 to 0.88);  stroke (HR Q5 VS. Q1: 0.86, 95% CI: 0.78 to 0.95);  ischaemic stroke (HR Q5 VS. Q1: 0.86, 95% CI: 0.75 to 0.99) | [43] |
| Shojaei 2024 | Iran | 2017–2020 | - | Cross-sectional study | 6,465 | General population/adults, females and males aged 30–70 years | Relatively higher 1/4 EAT-Lancet diet score, Q1–Q4 [mean 41.5 (SD 2.9), mean 48.6 (SD 1.6), mean 54.5 (SD 1.8), and mean 64.2 (SD 5.3)] | Relatively lower 1/4 EAT-Lancet score | A 130-item FFQ | Metabolic syndrome, abdominal obesity, hyperglycemia, high blood pressure, hypo-HDL cholesterolemia, and hypertriglyceridemia (adjusted OR) | Age, sex, education level, wealth score index, smoking status, sleep time, and physical activity level, and energy intake | Higher adherence to EAT-Lancet diet was - associated with a lower risk of   metabolic syndrome (OR Q4 VS. Q1: 0.74, 95% CI: 0.60 to 0.92);  abdominal obesity (OR Q4 VS. Q1: 0.89, 95% CI: 0.79 to 0.98);  hypo-HDL cholesterolemia (OR Q4 VS. Q1: 0.74, 95% CI: 0.61 to 0.90)  - not associated with a lower risk of  hyperglycemia (OR Q4 VS. Q1: 0.91, 95% CI: 0.77 to 1.08);  high blood pressure (OR Q4 VS. Q1: 1.17, 95% CI: 0.89 to 1.53);  hypertriglyceridemia (OR Q4 VS. Q1: 1.07, 95% CI: 0.89 to 1.28) | [44] |
| Sotos-Prieto 2024 | UK | 2009–2012 | 9.4 years | Population-based prospective cohort study | 118,469 | General population/adults, females and males aged 40–69 years | Relatively higher 1/4 EAT-Lancet diet score, Q1–Q4 (21.1–71.1, 71.1–80.7, 80.7–89.9, and 89.9–128.5) | Relatively lower 1/4 EAT-Lancet score | A 24-h dietary recall | Total CVD, myocardial infarction, stroke (adjusted HR) | Sex, age, ethnicity, education, deprivation index, region of assessment, smoking status, energy intake, alcohol intake, physical activity, BMI, diabetes, cancer, hypertension, cholesterol-lowering medication, number of medications, and vitamin supplement use | Higher adherence to the EAT-Lancet score was  - associated with lower risk of  total CVD (HR Q4 VS. Q1: 0.86, 95% CI: 0.79 to 0.94);  myocardial infarction (HR Q4 VS. Q1: 0.88, 95% CI: 0.80 to 0.97);  stroke (HR Q4 VS. Q1: 0.82, 95% CI: 0.70 to 0.97) | [45] |
| Teixeira 2024 | Portugal | 2005–2006 | - | Population-based prospective cohort study | 3,564 | Infants (enrolled) | Relatively higher EAT-Lancet diet score (range from 0–130) | Relatively lower EAT-Lancet diet score | A 24-h dietary recall | Obesity - BMI ≥ 97th percentile, waist circumference ≥ 90th percentile, triglycerides ≥ 150 mg/dl, HDL-c < 40 ml/dl, systolic blood pressure ≥ 130 mmHg or diastolic blood pressure ≥ 85 mmHg, glucose ≥ 100 mg/dl, and metabolic syndrome (adjusted PR) | Mothers’ age, education, gestational diabetes and pre-pregnancy body mass index, child’s sex, exact age, practice of sports, tanner pubertal stage and for the total grams of the remaining foods (not included in the WISH score), outcome under study at age 7 | For children aged 10  Higher adherence to the EAT-Lancet score was  - not associated with a lower risk of  obesity - BMI ≥ 97th percentile, (PR each 10-point increase in EAT-Lancet score: 0.92, 95% CI: 0.83 to 1.01);  waist circumference ≥ 90th percentile, (PR each 10-point increase in EAT-Lancet score: 0.94, 95% CI: 0.86 to 1.02);  triglycerides ≥ 150 mg/dl, (PR each 10-point increase in EAT-Lancet score: 1.10, 95% CI: 0.25 to 4.80);  HDL-c < 40 ml/dl, (PR each 10-point increase in EAT-Lancet score: 1.07, 95% CI:0.91 to 1.27);  systolic blood pressure≥ 130 mmHg or diastolic blood pressure ≥ 85 mmHg, (PR each 10-point increase in EAT-Lancet score: 0.95, 95% CI: 0.77 to 1.17);  glucose ≥ 100 mg/dl, (PR each 10-point increase in EAT-Lancet score: 0.96, 95% CI: 0.76 to 1.21);  metabolic syndrome, (PR each 10-point increase in EAT-Lancet score: 0.98, 95% CI: 0.81 to 1.18)  For children aged 13  Higher adherence to the EAT-Lancet score was  - associated with a lower risk of  waist circumference ≥ 90th percentile, (PR each 10-point increase in EAT-Lancet score: 0.91, 95% CI: 0.82 to 1.00);  metabolic syndrome, (PR each 10-point increase in EAT-Lancet score: 0.83, 95% CI: 0.70 to 0.97)  - not associated with a lower risk of  obesity - BMI ≥ 97th percentile, (PR each 10-point increase in EAT-Lancet score: 0.91, 95% CI:0.81 to 1.03);  triglycerides ≥ 150 mg/dl, (PR each 10-point increase in EAT-Lancet score: 0.85, 95% CI: 0.67 to 1.08);  HDL-c < 40 ml/dl, (PR each 10-point increase in EAT-Lancet score: 1.00, 95% CI:0.90 to 1.14);  systolic blood pressure ≥ 130 mmHg or diastolic blood pressure ≥ 85 mmHg, (PR each 10-point increase in EAT-Lancet score: 0.89, 95% CI:0.74 to 1.08);  glucose ≥ 100 mg/dl, (PR each 10-point increase in EAT-Lancet score: 0.99, 95% CI: 0.49 to 1.15); | [46] |
| Teixeira 2024 | Portugal | 2015–2016 | - | Cross-sectional study | 1,153 | Children and adolescents, aged 3–17 years | Relatively higher EAT-Lancet diet score (range from 0–130) | Relatively lower EAT-Lancet diet score | Two non-consecutive 24-h recalls | Children-z-score BMI and adolescents-z-score BMI | Total grams of the remaining foods (not included in the scores), child’ sex and physical activity, parents’ maximum education level and mother’s age | Higher adherence to the EAT-Lancet diet was  - not associated with  children-z-score BMI (β = –0.121, 95% CI: –0.252 to 0.011);  adolescents-z-score BMI (β = 0.006, 95% CI: –0.094 to 0.106) | [47] |
| van Soest 2024 | The Netherlands | 2008–2013 | - | Cross-sectional study | 630 | Older adults, females and males aged ≥ 65 years | Relatively higher EAT-Lancet diet score (range from 0–42) | Relatively lower EAT-Lancet diet score | A 190-item FFQ | Global cognition, episodic memory, attention and working memory, information processing speed, and executive functioning | Age, gender, education, apolipoprotein E4 carrier status, BMI, physical activity, smoking, and alcohol consumption | Higher adherence to the EAT-Lancet diet was  - associated with a lower risk of  decline of global cognition (β = 0.04, 95% CI: 0.00 to 0.08);  decline of information processing speed (β = 0.06, 95% CI: 0.00 to 0.12);  decline of executive functioning (β = 0.07, 95% CI: 0.01 to 0.12)  - not associated with a lower risk of  decline of episodic memory (β = 0.00, 95% CI: –0.06 to 0.05);  decline of attention and working memory (β = 0.04, 95% CI: –0.03 to 0.11) | [48] |
|  | The Netherlands | 2008–2013 | 2 years | Prospective cohort study | 302 | Older adults, females and males aged ≥ 65 years | Relatively higher EAT-Lancet diet score (range from 0–42) | Relatively lower EAT-Lancet diet score | A 190-item FFQ | Global cognition, episodic memory, attention and working memory, information processing speed, and executive functioning | Age, gender, education, apolipoprotein E 4 carrier status, BMI, physical activity, smoking, and alcohol consumption | Higher adherence to the EAT-Lancet diet was  - associated with a lower risk of  decline of global cognition (β = 0.05, 95% CI: 0.02 to 0.08);  decline of attention and working memory (β = 0.11, 95% CI: 0.04 to 0.18);  decline of executive functioning (β = 0.07, 95% CI: 0.02 to 0.12)  - not associated with a lower risk of  decline of episodic memory (β = 0.02, 95% CI: –0.05 to 0.08);  decline of information processing speed (β = 0.02, 95% CI: –0.03 to 0.08) |  |
| Wu 2024 | UK | 2006–2010 | 11.6 years | Population-based prospective cohort study | 105,752 | General population/adults, females and males, aged 39–72 years | Relatively higher 1/4 EAT-Lancet diet score, Q1–Q4 (9–21, 22–24, 25–27, and 28–41) | Relatively lower 1/4 EAT-Lancet diet score | 24-h dietary recalls | Metabolic dysfunction-associated steatotic liver disease | Age, sex, BMI, total energy intake, smoking status, alcohol intake, educational level, Townsend deprivation index, physical activity, hypertension, diabetes, cancer, CVD, fasting duration, spectrometer, metabolic dysfunction-associated steatotic liver disease-polygenic risk score, ﬁrst 10 principal components of ancestry, and genotype measurement batch | Higher adherence to the EAT-Lancet diet was  - associated with a lower risk of head and neck cancers (HR Q4 VS. Q1: 0.79, 95% CI: 0.66 to 0.95) | [49] |
| Xia 2024 | UK | 2006–2010 | 13.15 years (median) | Prospective cohort study | 197,391 | General population/adults, females and males aged 40–69 years | Relatively higher 1/3 EAT-Lancet diet score, T1–T3 | Relatively lower 1/3 EAT-Lancet diet score | the Oxford Web, a web based 24-h recall questionnaire | Crohn’s disease AND ulcerative colitis (adjusted HR) | Age, sex, assessment center, sociodemographic characteristic, lifestyle facto, medication, and comorbidities | Higher adherence to EAT-Lancet diet was - not associated with  Crohn’s disease (HR T3 VS. T1: 1.09, 95% CI: 0.80 to 1.48);  ulcerative colitis (HR T3 VS. T1: 1.02, 95% CI: 0.83 to 1.27) | [50] |
| Ye 2024 | UK | 2006–2010 | 9.9 years | Population-based prospective cohort study | 114,165 | General population/adults, females and males, aged 40–69 years | Relatively higher 1/5 EAT-Lancet diet score, Q1–Q5 (10–44, 44–52, 52–59, 59–67, and 67–110) | Relatively lower 1/5 EAT-Lancet diet score | 24-h dietary recalls (at least two times) | CVD, ischemic heart disease, atrial fibrillation, heart failure, and stroke (adjusted HR) | Age, sex, energy intake, ethnicity, educational level, smoking status, alcohol consumption, physical activity, Townsend deprivation index, and family history of CVDs | Higher adherence to the EAT-Lancet diet was  - associated with a lower risk of  CVD (HR Q5 VS. Q1 0.79, 95% CI: 0.74 to 0.84);  ischemic heart disease (HR Q5 VS. Q1 0.73, 95% CI: 0.67 to 0.79);  atrial fibrillation (HR Q5 VS. Q1 0.90, 95% CI: 0.82 to 0.99);  heart failure (HR Q5 VS. Q1 0.69, 95% CI: 0.59 to 0.82)  - not associated with a lower risk of stroke (HR Q5 VS. Q1 0.88, 95% CI: 0.75 to 1.04) | [51] |
| Zhang 2024 | Sweden | 1991–1996 | 25 years | Population-based prospective cohort study | 23,260 | General population/adults, females and males aged 57.8 (SD 7.6) years | Relatively higher 1/5 EAT-Lancet diet score, Q1–Q5 (≤ 13, 14–16, 17–19, 20–22, and ≥ 23) | Relatively lower 1/5 EAT-Lancet diet score | Assessment consists of 3 parts: 1) 7-day (consecutive days) food diary  2) a 168-item FFQ 3) 60-minute interview | Heart failure (adjusted HR) | Age, sex, dietary assessment version, season, total energy intake, leisure–time physical activity, alcohol consumption, smoking status, educational level, BMI, baseline diabetes, hypertension, use of lipid lowering drugs, and family history of myocardial infarction | Higher adherence to the EAT-Lancet diet was - associated with a lower risk of heart failure (HR Q5 vs. Q1: 0.93, 95% CI: 0.88 to 0.97) | [52] |
| Zhang 2024 | China & UK | TCLSIH cohort:2013–ongoing,  GNHS cohort: 2008–2013;  UK Biobank: 2006–2010 | TCLSIH cohort: 4.2 years (median);  GNHS cohort: 5.9 years (median);  UK Biobank: 10.5 years (median); | Prospective cohort study | Total: 191,478; TCLSIH cohort: 15,263;  GNHS cohort: 1137 ;  UK Biobank: 175,078 | General population/adults, females and males, TCLSIH cohort: aged ≥ 18 years;  GNHS cohort: aged 40–75 years;  UK Biobank: aged 39–72 years; | TCLSIH cohort & UK Biobank: Relatively higher 1/3 EAT-Lancet diet score, T1–T3 (≤ 10, 11 and ≥ 12)  GNHS cohort: Relatively higher 1/3 EAT-Lancet diet score, T1–T3 (≤ 9, 10 and ≥ 11) | Relatively lower 1/3 EAT-Lancet score | TCLSIH cohort & GNHS cohort: semi-quantitative FFQs  UK Biobank: 24-h dietary recalls (5 time points using the Oxford WebQ) | Metabolic dysfunction-associated steatotic liver disease (MASLD) (adjusted HR) | Age, sex, and baseline body mass index, smoking status, alcohol drinking status, educational level, household income per month, Townsend deprivation index (only in the UK Biobank), physical activity, depression, dietary supplement use, family history of disease (including cardiovascular disease, hypertension, and diabetes), total energy intake, hypertension, hyperlipidemia, and diabetes | Higher adherence to the EAT-Lancet score was  - associated with lower risk of MASLD  TCLSIH cohort: (HR T3 VS. T1: 0.87, 95% CI: 0.78 to 0.96);  GNHS cohort: (HR T3 VS. T1: 0.79, 95% CI: 0.64 to 0.98);  UK Biobank: (HR T3 VS. T1: 0.73, 95% CI: 0.63 to 0.85) | [53] |
|  | China | 2018 | - | Cross-sectional study | PERSONS cohort: 228 | General population/adults, females and males, aged 39.0 (SD 12.5) years | Relatively higher EAT-Lancet diet score | Relatively lower EAT-Lancet diet score | semi-quantitative FFQs | Metabolic dysfunction–associated steatotic liver disease (MASLD), Steatosis, Balloonin, Lobular inflammation, Fibrosis, controlled attenuation parameter (CAP); liver stiffness measurement [adjusted β value (95% CI)] | Age and sex, smoking status, alcohol drinking status, educational level, physical activity, depression, total energy intake, body mass index, hypertension, hyperlipidemia, and diabetes | Higher adherence to the EAT-Lancet score was  - associated with lower CAP (β = −5.895, 95% CI: −10.014 to −1.775)  - not associated with  MASLD (β = −0.067, 95% CI: −0.228 to 0.094);  steatosis (β = −0.055, 95% CI: −0.144 to 0.034);  ballooning (β = 0.002, 95% CI: −0.063 to 0.068);  lobular inflammation (β = −0.015, 95% CI: −0.087 to 0.058);  Fibrosis (β = 0.028, 95% CI: −0.074 to 0.129);  LSM (β = −0.155, 95% CI: −0.596 to 0.285) |  |
| Zhang 2024 | Singapore | 1993–1998 | 20 years | Prospective cohort study | 16,736 | General population/adults, females and males aged 45–74 years | Relatively higher 1/5 EAT-Lancet diet score, Q1–Q5 (16–49, 50–54, 55–59, 60-64, and 65–92) | Relatively lower 1/5 EAT-Lancet diet score | A 165-item FFQ | Cognitive impairment (adjusted OR) | Age at follow-up 3 visit, sex, dialect group, education level, year of recruitment, marital status, cigarette smoking, physical activity level, alcohol consumption, sleep duration, BMI, total energy intake, diabetes, CVD, and cancer | Higher adherence to the EAT-Lancet diet score was - associated with lower risk of poor cognitive function (OR Q5 vs. Q1: 0.89, 95% CI: 0.83 to 0.96) in non-carriers of apolipoprotein E ε4 allele | [53] |
| Zhao 2024 | UK | 2006–2010 | 12.24 years (median) | Prospective cohort study | 210,944 | General population/adults, females and males, aged 56.2 (SD 7.9) years | Relatively higher 1/3 EAT-Lancet diet score, T1–T3 (≤ 10, 11 and ≥ 12) | Relatively lower 1/3 EAT-Lancet score | 24-h dietary recalls | all-cause dementia (adjusted HR) | Sex, age, energy intake, body mass index, alcohol consumption, smoke status, ethnic, total cholesterol, HDL-c, LDL-c, depressive mood, apolipoprotein E ɛ4 carrier status, socioeconomic status (only in all population), family history of dementia, and prevalent comorbidities (hearing impairment, hypertension, T2D, car- diovascular disease) and medication used (hypertension and diabetes) | Higher adherence to the EAT-Lancet score was  - associated with lower risk of all-cause dementia in individuals with high socioeconomic status (HR T3 VS. T1: 0.60, 95% CI: 0.40 to 0.88) | [54] |
| Berthy 2025 | France | 2009–2022 | 8.79 years (median) | Prospective cohort study | 88,964 | General population/adults, females and males aged ≥ 18 years | Relatively higher 1/5 EAT-Lancet diet score, Q1–Q5 (cut-offs for quintiles of EAT-Lancet diet score were 192.2/25.5/38.3/49.1/62.8/332.2 for females and 148.7/22.6/34.9/44.9/57.7/ 332.2 for males) | Relatively lower 1/5 EAT-Lancet diet score | Three nonconsecutive web-based 24-hour dietary records | Incident T2D (adjusted HR) | Age, sex, energy intake without alcohol,  number of 24 hour dietary records, family history of diabetes, season of inclusion in the cohort, education level, occupational status, monthly household  income, cohabiting status, physical activity, smoking status, number of smoked cigarettes in pack-years, and alcohol consumption | Higher adherence to EAT-Lancet diet was  - direct associated with a lower risk of T2D  (HR Q5 VS. Q1: 0.71, 95% CI: 0.56 to 0.89) | [55] |
| Chen 2025 | USA | 1999–2018 | 10.4 years (median) | Prospective cohort study | 3,442 | General population/adults, females and males aged 62.5 (SE 0.33) years | Relatively higher 1/5 EAT-Lancet diet score, Q1–Q5 (range from 0– 10) | Relatively lower 1/5 EAT-Lancet diet score | Self-reported  dietary data | All-cause mortality, cancer mortality, and non-cancer mortality (adjusted HR) | Age, sex, race/ethnicity, marital status, educational level, poverty income ratio, physical activity, alcohol use, smoke, BMI, energy, baseline year, hypertension, diabetes mellitus, and CVD | Higher adherence to EAT-Lancet diet was - associated with a lower risk of  all-cause mortality (HR Q5 VS. Q1: 0.67, 95% CI: 0.53 to 0.83)  non-cancer mortality (HR Q5 VS. Q1: 0.62, 95% CI: 0.47 to 0.81)  - not associated with a lower risk of  cancer mortality (HR Q5 VS. Q1: 0.79, 95% CI: 0.52 to 1.20) | [56] |
| Dehnavi 2025 | Iran | 2022–2023 | - | Cross-sectional study | 398 | Elderly individuals, females and males aged 60–84 years | Relatively higher 1/3 EAT-Lancet diet score, T1–T3 (≤ 53, 53–61 and ≥ 61) | Relatively lower 1/3 EAT-Lancet diet score | A semi-quantitative 168-item FFQ | BMI ≥ 27.5, waist circumference ≥ 95, abdominal volume index ≥ 3.17, body roundness index ≥ 5.20, body shape  index ≥ 0.08; conicity Index: placement in the third tertile of dietary diversity is reported as the dependent variable (adjusted OR) | Age, gender, energy intake, physical activity, marital status, socio-economic status, household size, smoking status, and supplement intake | Higher adherence to the EAT-Lancet score was - associated with lower   BMI (OR T3 VS. T1: 0.31, 95% CI: 0.17 to 0.56);  waist circumference (OR T3 VS. T1: 0.53, 95% CI: 0.32 to 0.90);  body roundness index (OR T3 VS. T1: 0.43, 95% CI: 0.25 to 0.75)  - not associated with  conicity Index (OR T3 VS. T1: 1.04, 95% CI: 0.62 to 1.75);  abdominal volume index (OR T3 VS. T1: 0.94, 95% CI: 0.54 to 1.65);  body shape index (OR T3 VS. T1: 1.66, 95% CI: 0.97 to 2.86) | [57] |
| Han 2025 | USA | 2005–2018 | 8.5 years | Prospective cohort study | 30,521 | General population/adults, females and males aged ≥ 20 years | Relatively higher 1/4 EAT-Lancet diet score, Q1–Q4 (≤ 40, 40–50, 50–61, and ≥ 61) | Relatively lower 1/4 EAT-Lancet diet score | Two 24-hour dietary recalls | All-cause mortality, CVD mortality, cancer mortality, and other-cause mortality (adjusted HR) | Age, sex, NHANES cycle, race, body mass index, energy intake, drinking or smoking status, physical activity level, diabetes, and hypertension | Higher adherence to EAT-Lancet diet was - associated with a lower risk of   all-cause mortality (HR Q4 VS. Q1: 0.64, 95% CI: 0.54–0.75);  CVD mortality (HR Q4 VS. Q1: 0.73, 95% CI: 0.55–0.97);  cancer mortality (HR Q4 VS. Q1: 0.68, 95% CI: 0.52–0.89);  other-cause mortality (HR Q4 VS. Q1: 0.58, 95% CI: 0.46–0.72) | [58] |
| Hu 2025 | UK | 2006–2010 | 13.05 years (median) | Prospective cohort study | 177,441 | General population/adults, females and males aged 37–73 years | Relatively higher 1/5 EAT-Lancet diet score, Q1–Q5 (divide into 5  parts by number of people evenly) | Relatively lower 1/5 EAT-Lancet diet score | Semi-quantitative 24-h food recall questionnaire (Oxford WebQ) | Colorectal cancer (adjusted HR) | Age, sex, BMI, total energy intake, the ﬁrst 10 genetic principal  components, income, education levels, Townsend deprivation index, smoking  status, alcohol intake, and family history of cancer | Higher adherence to EAT-Lancet diet was associated with a lower risk of colorectal cancer (HR Q5 VS. Q1: 0.87, 95% CI: 0.76 to 0.99) | [59] |
| Jiang  2025 | USA | 2005–2018 | - | Cross-sectional study | 30,446 | General population/adults, females and males aged ≥ 20 years | Relatively higher 1/4 EAT-Lancet diet score, Q1–Q4 (range from 0–140) | Relatively lower 1/4 EAT-Lancet diet score | Two days of 24-h dietary recall | Depression (adjusted OR) | Age, gender, education level, marital, ratio of family income to poverty, race, obesity, Smoking,  Physical activity, hypertension, diabetes, and high cholesterol | Higher adherence to EAT-Lancet diet was associated with a lower risk of depression (OR Q4 VS. Q1: 0.73, 95% CI: 0.58 to 0.98) | [60] |
| Karavasiloglou 2025 | UK | 2006–2010 | 11.5 years | Prospective cohort study | 25,348 | General population/adults, females and males aged 60.1 (SD 7.0) years | Relatively higher 1/3 EAT-Lancet diet score, T1–T3 (1–4, 5, and 6–11) | Relatively lower 1/3 EAT-Lancet diet score | the UK Biobank Touchscreen questionnaire | All-cause mortality, cancer mortality, and CVD mortality (adjusted HR) | Age, sex, and region plus further adjustment for education, Townsend deprivation index, smoking status, body mass index, physical activity, alcohol  consumption, self-reported changes in the diet in the past 5 years, and time between the initial cancer diagnosis and study recruitment | Higher adherence to EAT-Lancet diet was - associated with a lower risk of  all-cause mortality (HR T3 VS. T1: 0.90, 95% CI: 0.84 to 0.99);  cancer mortality (HR T3 VS. T1: 0.92, 95% CI: 0.85 to 0.99)  - not associated with a lower risk of  CVD mortality (HR T3 VS. T1: 0.95, 95% CI: 0.82 to 1.09) | [61] |
| Lan 2025 | USA | 2005–2018 | 14 years | Prospective cohort study | 25,312 | General population/adults, females and males aged 47.85 (SE 0.25) years | Relatively higher 1/4 EAT-Lancet diet score, Q1–Q4 [mean 38.9 (SD 24.5), mean 39.9 (SD 25.1), mean 39.3 (SD 24.7), and mean 41.0 (SD 25.8)] | Relatively lower 1/4 EAT-Lancet score | Self-reported data across 14 dietary groups | All-cause mortality with depression (adjusted HR) | Age, sex, race/ethnicity, marital status, education, PIR, smoking, drinking, physical activity, BMI, and uric acid | Higher adherence to EAT-Lancet diet was - associated with a lower risk of all-cause mortality with depression (HR Q4 VS. Q1: 0.55, 95% CI: 0.35 to 0.87) | [62] |
|  | USA | 2005–2018 | - | Cross-sectional study | 25,312 | General population/adults, females and males aged 47.85 (SE 0.25) years | Relatively higher 1/4 EAT-Lancet diet score, Q1–Q4 [mean 38.9 (SD 24.5), mean 39.9 (SD 25.1), mean 39.3 (SD 24.7), and mean 41.0 (SD 25.8)] | Relatively lower 1/4 EAT-Lancet score | Self-reported data across 14 dietary groups | Depression (adjusted OR) | Age, sex, race/ethnicity, marital status, education, PIR, smoking, drinking, physical activity, BMI, and uric acid | Higher adherence to EAT-Lancet diet was associated with a lower risk of depression (OR Q4 VS. Q1: 0.66, 95% CI: 0.55 to 0.86) |  |
| Martins 2025 | Switzerland | 2009–2012 | 7.9 years | Prospective cohort study | 3,866 | General population/adults, females and males aged 35–75 years | Relatively higher 1/3 EAT-Lancet diet score, T1–T3 (≤ 18, 19–21, and ≥ 22) | Relatively lower 1/3 EAT-Lancet diet score | A 97-item semiquantitative FFQ | Cardiovascular events, and all-cause mortality (adjusted HR);  Changes of BMI, waist circumference, waist-to-hip ratio, systolic blood pressure, diastolic blood pressure, total cholesterol, HDL-c, LDL-c, and triglycerides [T3 VS. T1, mean (SD)] | For cardiovascular events and all-cause mortality:  Age, sex, education  level, total energy intake, sedentary lifestyle, smoking, and alcohol consumption, hypercholesterolemia or lipidlowering, medication use, hypertension or antihypertensive medication use, diabetes or glucose-lowering medication use, and BMI;  For other indicators: Age, sex, education level, total energy intake, sedentary lifestyle, smoking, alcohol consumption, hypercholesterolemia or lipidlowering medication use, hypertension or antihypertensive medication use, and BMI | Higher adherence to EAT-Lancet diet was - associated with  a lower risk of all-cause mortality (HR T3 VS. T1: 0.70, 95% CI: 0.50 to 0.98);  changes of waist circumference [T3, 0.26 (7.25) VS. T1, 1.59 (7.05), *P* < 0.001];  changes of waist-to-hip ratio [T3, −0.03 (0.08) VS. T1, −0.02 (0.06), *P* < 0.001];  changes of total cholesterol [T3, −0.46 (1.02) VS. T1, −0.57 (0.59), *P* = 0.014];  changes of triglyceride [T3, 0.03 (0.78) VS. T1, −0.04 (0.88), *P* = 0.041]  - not associated with  a lower risk of cardiovascular events (HR T3 VS. T1: 1.09, 95% CI: 0.81 to 1.46);  changes of BMI [T3, 0.40 (2.24) VS. T1, 0.49 (2.14), *P* = 0.433];  changes of systolic blood pressure [T3, −0.45 (9.66) VS. T1, −0.15 (10.70), *P* = 0.591];  changes of diastolic blood pressure [T3, 4.10 (15.93) VS. T1, 4.47 (14.27), *P* = 0.565];  changes of HDL-c [T3, −0.07 (0.28) VS. T1, −0.09 (0.27), *P* = 0.283];  changes of LDL-c [T3, −0.46 (0.87) VS. T1, −0.40 (0.91), *P* = 0.107] | [63] |
| Nair 2025 | USA | 2010–2013 | 5 years | Prospective cohort study | 735 | South Asian  ethnicity, females and males aged 40–84 years | Relatively higher EAT-Lancet diet score (range from 0–140) | Relatively lower EAT-Lancet diet score | A 163-item semi-quantitative FFQ | Fasting glucose, glycated hemoglobin, HDL-c, LDL-c, triglycerides, body weight, BMI, waist circumference, systolic blood pressure, and diastolic blood  pressure [adjusted β value or % increase (SE)] | For all indicators:  Age, sex, study site, education, smoking status, alcohol, family history of diabetes, years, lived in United States, physical activity, reﬁned grain  intake, total energy, sum of cultural traditional measures,  baseline value of the respective cardiometabolic risk factor by using multivariable linear regression, diabetes medication use, cholesterol-lowering medication use, hypertension medication use, BMI and waist circumference;  Specific for BMI, and body weight: subtract BMI and waist circumference;  Specific for waist circumference: subtract waist circumference | Higher adherence to EAT-Lancet diet was - associated with  lower glycated hemoglobin [β = −0.08 (SE 0.04), *P* = 0.033];  lower body weight [β = −0.37 (SE 0.12), *P* = 0.003];  lower BMI [β = −0.08 (SE 0.04), *P* = 0.026];  lower waist circumference [β = −0.49 (SE 0.17), *P* = 0.005];  lower systolic blood pressure [β = −0.66 (SE 0.30), *P* = 0.028];  higher HDL-c [β = 0.40 (SE 0.17), *P* = 0.02]  - not associated with  fasting glucose [β = −0.29 (SE 0.15), *P* = 0.052];  triglycerides [β = 1.99 (SE 2.56), *P* = 0.44];  LDL-c [β = 0.02 (SE 0.01), *P* = 0.06];  diastolic blood pressure [β = −0.11 (SE 0.06), *P* = 0.080] | [64] |
|  | USA | 2010–2013 | - | Cross-sectional study | 891 | South Asian  ethnicity, females and males aged 40–84 years | Relatively higher EAT-Lancet diet score (range from 0–140) | Relatively lower EAT-Lancet diet score | A 163-item semi-quantitative FFQ | Hypertension, fatty liver, metabolic syndrome, overweight, obesity, incident hypertension, and incident diabetes (adjusted OR);  Coronary artery calcium score, internal carotid intima media thickness, common carotid intima media thickness, fasting glucose, glycated hemoglobin, β-cell function, HOMA-IR, triglycerides, HDL-c, LDL-c, CRP, adiponectin, body weight, BMI, waist circumference, subcutaneous fat area, visceral fat area, pericardial fat volume, and hepatic fat attenuation [adjusted β value or % increase (SE)] | For all diseases and indicators:  Age, sex, study site, education, smoking status,  alcohol, family history of diabetes, years lived in United States, physical activity,  reﬁned grain intake, total energy, the sum of cultural traditional measures, use of diabetes medication, cholesterol-lowering medication, hypertension medication, and BMI;  Specific for BMI, and body weight: subtract BMI and waist circumference;  Specific for waist circumference, subcutaneous fat area, and visceral fat area: subtract waist circumference | Higher adherence to EAT-Lancet diet was - associated with  a lower risk of overweight (OR per 10 EAT-Lancet diet score increase: 0.77, 95% CI: 0.74 to 0.85);  a lower risk of obesity (OR per 10 EAT-Lancet diet score increase: 0.80, 95% CI: 0.71 to 0.92);  a lower risk of incident diabetes (OR per 10 EAT-Lancet diet score increase: 0.80, 95% CI: 0.54 to 0.86);  lower fasting glucose [β = −0.45 (SE 0.22), *P* = 0.034];  lower glycated hemoglobin [β = −0.49 (SE 0.22), *P* = 0.042];  lower LDL-c [β = −0.015 (SE 0.007), *P* = 0.023];  lower CRP [β = −5.40 (SE 2.42), *P* = 0.026];  lower body weight [β = −0.59 (SE 0.26), *P* = 0.021];  lower BMI [β = −0.27 (SE 0.11), *P* = 0.011];  lower waist circumference [β = −0.25 (SE 0.11), *P* = 0.029];  lower visceral fat area [β = −1.37 (SE 0.69), *P* = 0.045];  lower pericardial fat volume [β = −0.58 (SE 0.29), *P* = 0.047];  higher adiponectin [β = 4.67 (SE 2.02), *P* = 0.021]  - not associated with  a lower risk of hypertension (OR per 10 EAT-Lancet diet score increase: 1.09, 95% CI: 0.90 to 1.32);  a lower risk of fatty liver (OR per 10 EAT-Lancet diet score increase: 1.04, 95% CI: 0.76 to 1.43);  a lower risk of metabolic syndrome (OR per 10 EAT-Lancet diet score increase: 0.85, 95% CI: 0.69 to 1.06);  a lower risk of incident hypertension (OR per 10 EAT-Lancet diet score increase: 0.91, 95% CI: 0.66 to 1.10);  coronary artery calcium score [β = 0.027 (SE 0.014), *P* = 0.057];  internal carotid intima media thickness [β = −0.12 (SE 1.06), *P* = 0.91];  common carotid intima media thickness [β = −1.26 (0.80), *P* = 0.11];  β-cell function [β = −4.65 (SE 3.95), *P* = 0.22];  HOMA-IR [β = −1.86 (SE 2.52), *P* = 0.45];  triglycerides [β = 2.17 (SE 1.82), *P* = 0.24];  HDL-c [β = 0.008 (SE 0.005), *P* = 0.079];  subcutaneous fat area [β = −0.86 (SE 0.49), *P* = 0.077];  hepatic fat attenuation [β = 0.10 (SE 0.12), P = 0.077] |  |
| Samuelsson 2025 | Sweden | 1991–1996 | 18 years | Prospective cohort study | 25,898 | General population/adults, females and males aged 45–73 years | Relatively higher EAT-Lancet diet score (including 7 versions score, Knuppel, Trijsburg, Hanley-Cook, Kesse-Guyot, Stubbendorff, Colizzi, and Bui) | Relatively lower EAT-Lancet diet score | A 7-day food diary and a 168-item FFQ | All-cause dementia, Alzheimer’s disease, and vascular dementia (adjusted HR) | Age, sex, season, dietary method version, energy intake, education,  smoking, alcohol consumption, physical activity, and BMI | Higher adherence to EAT-Lancet diet  - may be associated with a lower risk of  all-cause dementia (Knuppel, HR per 10% EAT-Lancet diet score increase: 0.96, 95% CI: 0.92 to 1.01)//(Trijsburg, HR per 10% EAT-Lancet diet score increase: 0.99, 95% CI: 0.96 to 1.03)//(Hanley-Cook, HR per 10% EAT-Lancet diet score increase: 0.97, 95% CI: 0.93 to 1.00)//(Kesse-Guyot, HR per 10% EAT-Lancet diet score increase: 0.94, 95% CI: 0.90 to 0.99)//(Stubbendorff, HR per 10% EAT-Lancet diet score increase: 0.99, 95% CI: 0.94 to 1.04)//(Colizzi, HR per 10% EAT-Lancet diet score increase: 0.98, 95% CI: 0.93 to 1.02)//(Bui, HR per 10% EAT-Lancet diet score increase: 0.98, 95% CI: 0.92 to 1.03);  Alzheimer’s disease (Knuppel, HR per 10% EAT-Lancet diet score increase: 0.96, 95% CI: 0.89 to 1.03)//(Trijsburg, HR per 10% EAT-Lancet diet score increase: 0.98, 95% CI: 0.93 to 1.04)//(Hanley-Cook, HR per 10% EAT-Lancet diet score increase: 0.97, 95% CI: 0.91 to 1.03)//(Kesse-Guyot, HR per 10% EAT-Lancet diet score increase: 0.87, 95% CI: 0.80 to 0.95)//(Stubbendorff, HR per 10% EAT-Lancet diet score increase: 0.98, 95% CI: 0.90 to 1.07)//(Colizzi, HR per 10% EAT-Lancet diet score increase: 0.96, 95% CI: 0.89 to 1.03)//(Bui, HR per 10% EAT-Lancet diet score increase: 0.93, 95% CI: 0.84 to 1.03)  - was not associated with a lower risk of  vascular dementia (Knuppel, HR per 10% EAT-Lancet diet score increase: 0.97, 95% CI: 0.86 to 1.09)//(Trijsburg, HR per 10% EAT-Lancet diet score increase: 0.99, 95% CI: 0.90 to 1.09)//(Hanley-Cook, HR per 10% EAT-Lancet diet score increase: 1.01, 95% CI: 0.92 to 1.11)//(Kesse-Guyot, HR per 10% EAT-Lancet diet score increase: 0.96, 95% CI: 0.84 to 1.10)//(Stubbendorff, HR per 10% EAT-Lancet diet score increase: 0.96, 95% CI: 0.84 to 1.10)//(Colizzi, HR per 10% EAT-Lancet diet score increase: 0.99, 95% CI: 0.89 to 1.11)//(Bui, HR per 10% EAT-Lancet diet score increase: 0.94, 95% CI: 0.81 to 1.10) | [65] |
| Shan 2025 | USA | 2001 | 18 years | Prospective cohort study | 33,824 | Black females, aged 21–69 years | Relatively higher 1/5 EAT-Lancet diet score, Q1–Q5 (< 71.2, 71.2–78.2, 78.2–84.4, 24.4–91.4, and ≥ 91.4) | Relatively lower 1/5 EAT-Lancet diet score | the Black Women’s Health Study FFQ | All-cause mortality, CVD mortality, and cancer-specific mortality (adjusted HR) | Age, energy, education, BMI, smoking status, alcohol consumption, and physical activity | Higher adherence to EAT-Lancet diet was - associated with a lower risk of   all-cause mortality (HR Q5 VS. Q1: 0.82, 95% CI: 0.71 to 0.94);  CVD mortality (HR Q5 VS. Q1: 0.74, 95% CI: 0.55 to 0.98)  - not associated with a lower risk of  cancer-specific mortality (HR Q5 VS. Q1: 0.91, 95% CI: 0.74 to 1.12) | [66] |
| Tabatabaei 2025 | Iran | 2018– 2019 | - | Cross-sectional study | 1,970 | General population/adults, females and males aged ≥ 18 years | Relatively higher 1/3 EAT-Lancet diet score, T1–T3 (–160.44 to –9.81, –9.77 to 10.06, 10.15 to 105.63) | Relatively lower 1/3 EAT-Lancet diet score | A 110-item semi-quantitative FFQ | Depression and anxiety (adjusted OR) | Age, sex, energy, marital status, smoking, physical activity, and socioeconomic status and center effect | Higher adherence to EAT-Lancet diet was - not associated with a lower risk of   depression (OR T3 VS. T1: 0.78, 95% CI: 0.57 to 1.05);  anxiety (OR T3 VS. T1: 0.81, 95% CI: 0.61 to 1.09) | [67] |
| Tan 2025 | USA | 2005–2018 | - | Cross-sectional study | 27,756 | General population/adults, females and males aged ≥ 20 years | Relatively higher 1/4 EAT-Lancet diet score, Q1–Q4 | Relatively lower 1/4 EAT-Lancet diet score | A 24-h dietary recall | Depressive symptoms (adjusted OR) | Age, gender, race, poverty-to-income ratio, education, BMI, smoking, and hypertension | Higher adherence to EAT-Lancet diet was associated with a lower risk of depression (OR Q4 VS. Q1: 0.72, 95% CI: 0.62 to 0.83) | [68] |
| Tang 2025 | China | 1989–2015 | 12 years (median) | Prospective cohort study | 3,404 | General population/adults, females and males aged ≥ 60 years, with normal cognition at baseline | Relatively higher 1/5 EAT-Lancet diet score, Q1–Q5 [mean 66.2 (SD 5.5), mean 70.5 (SD 0.1), mean 71.2 (SD 0.3), mean 74.7 (SD 1.9) and mean 85.6 (SD 5.9)] | Relatively lower 1/5 EAT-Lancet score | A 3-day 24 h dietary recalls | Memory, attention, calculation and cognition [adjusted β value (95% CI)] | Age, gender, education, marriage, and residency, region, household income per capita, smoking, alcohol consumption, and BMI | Higher adherence to EAT-Lancet diet was - associated with   slower memory decline (β = 0.025, 95% CI: 0.007 to 0.042);  slower global cognitive decline (β = 0.020, 95% CI: 0.004 to 0.037);  lower global cognitive decline (β = 0.020, 95% CI: 0.004 to 0.037)  - not associated with  attention (β = 0.011, 95% CI: –0.018 to 0.004);  calculation (β = 0.024, 95% CI: –0.001 to 0.048) | [69] |
| Watanabe 2025 | Japan | 2011–2012 | 4.75 years (median) | Prospective cohort study | 8,043 | General population/adults, females and males aged ≥ 65 years | Relatively higher 1/4 EAT-Lancet diet score, Q1–Q4 (≤ 25, 26–27, 28–30, and ≥ 31) | Relatively lower 1/4 EAT-Lancet diet score | A 47-item semi-quantitative FFQ | All-cause mortality and functional disability (adjusted HR) | Age, sex, and population density, BMI, family structure, economic status, educational attainment, smoking status, alcohol consumption status, physical activity, sitting time, sleep time, denture use, medication use, number of chronic diseases, frailty status, energy intake, green tea consumption, and coffee consumption | Higher adherence to EAT-Lancet diet was - not associated with  all-cause mortality (HR Q4 VS. Q1: 0.85, 95% CI: 0.67 to 1.09);  functional disability (HR Q4 VS. Q1: 0.88, 95% CI: 0.74 to 1.05) | [70] |
| Wu 2025 | China | 1997–2015 | 10 years | Prospective cohort study | 14,652 (new-onset cardiometabolic diseases); 15,318 (all-cause mortality) | General population/adults, females and males aged ≥ 18 years | Relatively higher 1/4 EAT-Lancet diet score, Q1–Q4 (PHDI and WISH) | Relatively lower 1/4 EAT-Lancet diet score | 3-day consecutive 24-h dietary recalls, and consumption of cooking oils and condiments was determined at the household level using a food weighing method | Myocardial infarction, T2DM, stroke, and all-cause mortality (adjusted HR) | For all diseases: Age, sex, BMI, region, urbanization index, educational level, physical activity, baseline hypertension, smoking status, alcohol intake, and total energy intake  Specific for stroke:  the sodium: potassium ratio | Higher adherence to EAT-Lancet diet was  - associated with  myocardial infarction (PHDI, HR Q4 VS. Q1: 0.14, 95% CI: 0.07 to 0.29); (WISH, HR Q4 VS. Q1: 0.68, 95% CI: 0.48 to 0.96);  T2D (PHDI, HR Q4 VS. Q1: 0.68, 95% CI: 0.57 to 0.82); (WISH, HR Q4 VS. Q1: 0.81, 95% CI: 0.67 to 0.96);  all-cause mortality (PHDI, HR Q4 VS. Q1: 0.60, 95% CI: 0.46 to 0.80); (WISH, HR Q4 VS. Q1: 0.80, 95% CI: 0.68 to 0.95)  - not associated with  stroke (PHDI, HR Q4 VS. Q1: 0.82, 95% CI: 0.60 to 1.10); (WISH, HR Q4 VS. Q1: 0.87, 95% CI: 0.65 to 1.16) | [71] |
| Zhan 2025 | US | 2005–2018 | - | Cross-sectional study | 27,181 | General population/adults, females and males aged > 21 years | Relatively higher 1/5 EAT-Lancet diet score, Q1–Q5 | Relatively lower 1/5 EAT-Lancet score | Two 24-hour dietary recall | Systolic blood pressure, diastolic blood pressure, HDl-c, triglyceride, LDL-c, glycohemoglobin, fasting plasma glucose, BMI, waist circumference [adjusted β value (95% CI)]; obesity, and abdominal obesity (adjusted PR) | Age group, sex, total energy intake, race and ethnicity, family income to poverty ratio, household size, educational level, marital status, smoking status, alcohol intake, physical activity, dietary supplement use, BMI category, and NHANES cycle;  BMI and waist circumference were not adjusted for BMI category | Higher adherence to EAT-Lancet diet was - associated with   higher HDL-c (β = 0.31, 95% CI: 0.15 to 0.47);  lower triglyceride (β = −22, 95% CI: −3.9 to −5.1);  lower BMI (β = −2.2, 95% CI: −3.0 to −1.5);  waist circumference (β = −6.1, 95% CI: −7.8 to −4.5);  obesity (PR Q5 VS. Q1: 0.59, 95% CI: 0.50 to 0.69);  abdominal obesity (PR Q5 VS. Q1: 0.74, 95% CI: 0.66 to 0.82)  - not associated with  systolic blood pressure (β = 0.03, 95% CI: −1.7 to 1.8);  diastolic blood pressure (β = −0.12, 95% CI: −1.4 to 1.2);  LDL-c (β = −0.76, 95% CI: −6.1 to 4.6);  glycohemoglobin (β = −0.03, 95% CI: −0.10 to 0.03);  fasting plasma glucose (β = 0.66, 95% CI: −2.5 to 3.8) | [72] |
|  | US | 2005–2018 | 12.58 years | Prospective cohort study | 27,181 | General population/adults, females and males aged > 21 years | Relatively higher 1/5 EAT-Lancet diet score, Q1–Q5 | Relatively lower 1/5 EAT-Lancet score | Two 24-hour dietary recall | all-cause mortality (adjusted HR) | Age group, sex, race and ethnicity, family income to poverty ratio, household size, educational level, marital status, smoking status, alcohol intake, physical activity, and dietary supplement use | Higher adherence to EAT-Lancet diet was associated with a lower risk of all-cause mortality (HR Q5 VS. Q1: 0.67, 95% CI: 0.46 to 0.98) |  |
| **Studies included in the systematic review only** | | | | | | | | | | | | |  |
| Sheng 2021 | China | 2020 | - | Modelling study | - | General population of all ages | EAT-Lancet diet | a. the Chinese Food Guide Pyramid  b. the mediterranean diet  c. the flexitarian diet China Statistical Yearbook (National Bureau of Statistics of China, 1998–2020) | Food consumption data | Chronic diseases | - | Adherence to the EAT-Lancet diet would be  - associated with lower risk of premature deaths and the mortality rates  - 1.8 million premature deaths and 19.2% lower mortality rates could be avoided by people aged 20 and above  - 0.7 million premature deaths and 19.5% lower mortality rates could be avoided by people aged from 30 to 69 | [73] |
| Koelman 2023 | Germany | 1994–1998 and 2001–2005 | T0 (baseline) to T1 (2001–2005): 6.8-years (mean); T1 to T2 (2010–2012) : 8.6-years (mean); and  T2 to T3 (2013): 1.4 years (mean) | Prospective cohort study | 636 | General population/adults, females and males, aged 35–64 years | Relatively higher adherence to EAT-Lancet diet (stable high, stable low, increasing, and decreasing, defined as scoring above/below baseline median at baseline and 6.8–years later) | Relatively lower adherence to EAT-Lancet diet | A 148-item semi-quantitative FFQ | Chemerin and hs-CRP [difference (95% CI)] | Age, sex, BMI, waist circumference, recreational sports (hours/week), educational attainment, smoking status, and prevalent diseases [hypertension (+ antihypertensive medication), T2D, cancer (except non-melanoma skin cancer), CVD | Higher adherence to the EAT-Lancet diet was  - associated with minor and not statistically significant reduction in the concentrations of inflammatory biomarkers on the long run | [74] |
| Neta 2023 | Brazil | 2019–2020 | - | Cross-sectional study | 411 | Adults, females and males aged ≥ 20 years | Relatively higher EAT-Lancet diet score (range from 0–140) | Relatively lower EAT-Lancet diet score | 24-h recalls | BMI (pearson correlation analysis) | - | Higher adherence to the EAT-Lancet diet was - associated with a lower BMI (r = –0.052 *P* = 0.036) | [75] |
| Suikki 2023 | Finland | 2000–2014 | 7 years | Population-based prospective cohort study | 4,371 | General population/adults, females and males aged 30–74 years | Relatively higher EAT-Lancet diet score (range from 0–13) | Relatively lower EAT-Lancet diet score | A 128-130 item FFQ | Body weight, waist circumference, and BMI [adjusted β value (95% CI)] | BMI and waist circumference: sex, age, log-transformed baseline body weight /BMI/ waist circumference, education, smoking, and leisure-time physical activity  body weight: sex, age, log-transformed baseline body weight /BMI/ waist circumference, education, smoking, leisure-time physical activity, and log-transformed baseline height | Higher adherence to EAT-Lancet diet was - not associated with anthropometric changes  1) body weight (β = −0.04, 95% CI: –0.19 to 0.11); 2) BMI (β = 0.05, 95% CI: –0.20 to 0.10); 3) waist circumference (β = −0.08, 95% CI: –0.22 to 0.06) | [76] |
| Zhang 2023 | China | 2022 | - | Case-control study | 1,012 | General population of all ages | Relatively higher 1/3 EAT-Lancet diet score, T1–T3 (≤ 26, 27–30, and > 30) | Relatively lower 1/3 EAT-Lancet diet score | A 114-item FFQ | Glioma risk | Age, BMI, occupation, education level, household income, high-risk residential areas, smoking status, alcohol consumption, history of allergies, history of head trauma, family history of cancer, physical activity, and energy intake | Adherence to the EAT-Lancet diet was - not associated with glioma risk | [77] |
| Ataei Kachouei 2024 | Iran | 2019 | - | Case-control study | 3,185 | General population, women aged ≤ 70 years, men aged ≤ 60 years | ELD-I: Relatively higher 1/4 EAT-Lancet diet score, Q1–Q4 [mean -59.0 (SD 38.0), mean -14.4 (SD 5.6), mean 6.4 (SD 5.6), and mean 34.1 (SD 15.8)]  ELD-MIV:  Relatively higher 1/4 EAT-Lancet diet score, Q1–Q4 (1.0–3.5, 3.6–7.0, 7.1–10.5, and 10.6–14) | Relatively lower 1/4 EAT-Lancet diet score | 110-item semi-quantitative FFQ | Premature coronary artery disease (PCAD) (adjusted OR) | Age, sex, ethnicity and energy, education, smoking, alcohol, physical activity, and marital  status, BMI, fasting blood sugar, high density lipoprotein cholesterol, low density of lipoprotein cholesterol,  total cholesterol, triglyceride, systolic and diastolic blood pressure and aspirin use | Higher adherence to the EAT-Lancet score was  - associated with lower risk of premature coronary artery disease (PCAD) (ELD-I: Q4 VS. Q1 OR: 0.29, 95% CI: 0.21 to 0.3) (ELD-MIV: Q4 VS. Q1 OR: 0.39, 95% CI: 0.29 to 0.52)  - associated with lower risk of severe premature coronary artery disease (PCAD) (ELD-I: Q4 VS. Q1 OR: 0.22, 95% CI: 0.17 to 0.28) (ELD-MIV: Q4 VS. Q1 OR: 0.28, 95% CI: 0.22 to 0.36) | [78] |
| Chang 2024 | China | 2011 | - | Cross-sectional study | 12,809 | General population/adults, females and males, aged ≥ 18 years (except pregnant  or lactating women participants with implausible energy intakes) | Relatively higher EAT-Lancet diet score | Relatively lower EAT-Lancet diet score | A 24-h dietary recall; the food inventory at household level | Coronary heart disease, T2D, and colorectal cancer (diet-related summed risks SR) | - | Higher adherence to the EAT-Lancet score was  - associated with a lower risk of  coronary heart disease (SR: 0.89, 95% CI: 0.75 to 1.0);  T2D (SR: 0.46, 95% CI: 0.22 to 1.06);    colorectal cancer (SR: 0.59, 95% CI: 0.39 to 0.97） | [79] |
| Davila-Cordova 2024 | Spain | 2021–2023 | - | Cross-sectional study | 200 | Reproductive-age men, aged 18–40 years | Relatively higher 1/3 EAT-Lancet diet score, T1–T3 (< 7, 7–8 and ≥ 8) | Relatively lower 1/3 EAT-Lancet diet score | A validated, semi-quantitative 143-item FFQ | Total sperm count, sperm concentration, sperm vitality, total motility, progressive motility, non-progressive motility, and normal sperm morphology [adjusted β value (95% CI)] | Age, smoking status, education, BMI, physical activity, sleeping hours, energy intake, and sexual abstinence | Higher adherence to EAT-Lancet diet was  - not associated with   total sperm count (β = 1.2, 95%CI: −1.0 to 3.3);  sperm concentration (β = 0.5, 95%CI: −0.7 to 1.7);  sperm vitality (β = 0.0, 95%CI: −0.5 to 0.4);  total motility (β = 2.6, 95%CI: −4.1 to 9.2);  progressive motility (β = 2.0, 95%CI: −4.7 to 8.7);  non-progressive motility (β = −0.1, 95%CI: −2.6 to 2.5);  normal sperm morphology (β = −0.3, 95%CI: −0.8 to 0.2) | [80] |
| Gu 2024 | Global | 2018 | - | Cross-sectional study | 171 countries (representing 98% of global population) | General population/adults, females and males aged 25–75 years | Relatively higher EAT-Lancet diet score (range from 0–140) | Relatively lower EAT-Lancet diet score | Food and Agriculture  Organization’s (FAO) Food Balance Sheets and Global Dietary Database (GDD) | Population attributable fraction (PAF) and preventable  deaths (95% CI) | - | Higher adherence to the EAT-Lancet diet (score = 140) would be associated with a lower risk of  all causes mortality (PAF% = 38.7, 95% CI: 35.5 to 41.9; preventable deaths = 21,125,986, 95% CI: 19,366,794 to 22,885,177);  nontraumatic causes mortality (PAF% = 38.3, 95% CI: 34.8 to 41.7; preventable deaths = 19,235,773, 95% CI: 17,506,346 to 20,965,199);  CVD mortality (PAF% = 21.2, 95% CI: 13.5 to 29.0; preventable deaths = 3,747,538, 95% CI: 2,380,238 to 5,114,838);  cancer mortality (PAF% = 22.0, 95% CI: 14.5 to 29.4; preventable deaths = 2,023,787, 95% CI: 1,336,756 to 2,710,819);  respiratory diseases mortality (PAF% = 66.6, 95% CI: 60.6 to 72.5; preventable deaths = 2,723,230, 95% CI: 2,480,356 to 2,966,104);  neurodegenerative diseases mortality (PAF% = 44.2, 95% CI: 36.4 to 52.1; preventable deaths = 1,011,197, 95% CI: 831,800 to 1,190,594);  infectious diseases mortality (PAF% = 31.1, 95% CI: 6.1 to 56.1; preventable deaths = 2,335,658, 95% CI: 459,763 to 4,211,552) | [81] |
| Hargous 2024 | Chile | 2016 | - | Cross-sectional study | 961 | Children, aged 3–6 years | Relatively higher EAT-Lancet diet score (range from 0–150) | Relatively lower EAT-Lancet diet score | Single multiple-pass 24-h dietary recall | Overweight and obesity | Day of the dietary recall, type of eating pattern, and type of diet | Higher adherence to the EAT-Lancet diet was  -not associated with a lower risk of  overweight (β: –0.1, 95% CI: –2.29 to 2.09);  obesity (β: 1.17, 95% CI: –1.44 to 3.78) | [82] |
| Huang 2024 | USA | 1999–2018 | - | Cross-sectional study | 44,925 | General population/adults, females and males aged 48.0 (SD 17.1) years | Relatively higher EAT-Lancet diet score (range from 0–140) | Relatively lower EAT-Lancet diet score | Two semi-quantitative 24-h diet recall questionnaires | Phenotypic age, biological age, telomere length, and klotho concentration. [adjusted β value (95% CI)] | Age, gender, education level, marital, PIR, race, smoking, drinking, hypertension, diabetes, and high cholesterol | Higher adherence to EAT-Lancet diet was - associated with   a decrease of phenotypic age (β = −0.20, 95% CI: −0.31 to −0.10);  a decrease of biological age (β = −0.54, 95% CI: −0.69 to −0.38);  an increase of telomere length (β = 6.2, 95% CI: 1.0 to 11.0)  - not associated with  telomere length (β = 0.01, 95%CI: −0.01 to −0.04) | [83] |
| Mohammadi 2024 | Iran | 2008–2010 | - | Case-control study | 213 | Recruited from three general hospitals and 19 CRC surgery departments, females and males aged 40–69 years | Relatively higher 1/3 EAT-Lancet diet score, T1–T3 (≤ 53, 54–62, and ≥ 63) | Relatively lower 1/3 EAT-Lancet diet score | A semi-quantitative FFQ | Colorectal cancer (adjusted OR) | Income, smoking status, family history of CRC in first- and second-degree  relatives, and taking ibuprofen, aspirin, and acetaminophen | Higher adherence to EAT-Lancet diet was associated with a lower risk of colorectal cancer (OR: 0.41, 95% CI: 0.18 to 0.91) | [84] |
| Teixeira 2024 | Portugal | 2005–2006 | - | Cross-sectional study | 3,564 | Infants (enrolled) | Relatively higher EAT-Lancet diet score (range from 0–130) | Relatively lower EAT-Lancet diet score | A 24-h dietary recall | Obesity - BMI ≥ 97th percentile, waist circumference ≥ 90th percentile, triglycerides ≥ 150 mg/dl, HDL-cholesterol < 40 ml/dl, systolic blood pressure ≥ 130 mmHg or diastolic blood pressure ≥ 85 mmHg, glucose ≥ 100 mg/dl, and metabolic syndrome (adjusted PR) | Mothers’ age, education, gestational diabetes and pre-pregnancy body mass index, child’s sex, exact age, practice of sports, and tanner pubertal stage and for the total grams of the remaining foods (not included in the WISH score) | For children aged 7 years  Higher adherence to the EAT-Lancet score was  - associated with a lower risk of  waist circumference ≥ 90th percentile, (PR each 10-point increase in EAT-Lancet score: 0.90, 95% CI: 0.83 to 0.98)；  - not associated with a lower risk of  obesity - BMI ≥ 97th percentile (PR each 10-point increase in EAT-Lancet score PR:0.97, 95% CI: 0.90 to 1.05)；  triglycerides ≥ 150 mg/dl, (PR each 10-point increase in EAT-Lancet score: 0.97, 95%CI: 0.81 to 1.16)；  HDL-c < 40 ml/dl, (PR each 10-point increase in EAT-Lancet score:0.96, 95%CI: 0.84 to 1.10)；  systolic blood pressure ≥ 130 mmHg or diastolic blood pressure ≥ 85 mmHg, (PR each 10-point increase in EAT-Lancet score:0.95, 95%CI: 0.75 to 1.19)；  glucose ≥ 100 mg/dl, (PR each 10-point increase in EAT-Lancet score: 1.13, 95%CI: 0.88 to 1.44)；  metabolic syndrome, (PR each 10-point increase in EAT-Lancet score:0.92, 95%CI: 0.71 to 1.20) | [46] |
| Ye 2024 | China | 2019 | - | Modelling study | - | General population of all ages | EAT-Lancet diet | a. Chinese dietary guidelines (CDGs)  b. WHO guidelines | National Bureau of Statistics of China (NBSC) and Food and Agriculture Organization (FAO) statistics | Health target attainment (%) | - | Higher adherence to EAT-Lancet diet would be  - associated with reduced mortality (Compared to CDGs and WHO guidelines in five regions: north, central, east, south, and southwest) | [85] |
| Cai 2025 | UK | 2006–2010 | 12.54 years (median) | Prospective cohort study | 141,562 | General population/adults, females and males aged 37–73 years | Relatively higher 1/4 EAT-Lancet diet score, Q1–Q4 (Stubbendorff, 9–21, 22–24, 25–27, and 28–41);  Relatively higher 1/4 EAT-Lancet diet score, Q1–Q4 (Knuppel, ≤ 8, 9, 10 and ˃ 10) | Relatively lower 1/4 EAT-Lancet diet score | 24-h dietary recall | Biological age acceleration (KDM-BA & PhenoAge, [adjusted β value (95% CI)] and additional life expectancy gain at 45 & 65 [adjusted years (95% CI)] | For biological age acceleration:  Age, sex, ethnicity, Townsend Deprivation  Index, physical activity, smoking status, alcohol intake frequency, household incom, college education, sleep quality score, BMI, total energy intake,  baseline hypertension, baseline hyperlipidemia, and baseline diabetes | Higher adherence to EAT-Lancet diet was  - associated with a lower risk of biological age acceleration  Stubbendorff, (β = –1.37, 95% CI: –1.51 to –1.24);  Knuppel, (β = –0.93, 95% CI: –1.00 to –0.86)  - associated with a favorable life expectancy  at 45 (mean = 1.13, 95% CI: 0.71 to 1.55);  at 65 (mean = 1.00, 95% CI: 0.63 to 1.37) | [86] |
| Carvalho 2025 | Portugal | 2015–2016 | - | Cross-sectional study | 3,852 | General population, aged 3 months–84 years | Relatively higher 1/3 EAT-Lancet diet score, T1–T3 [mean 21.9 (95% CI 21.5 to 22.4), mean 35.0 (95% CI 34.7 to 35.2), mean 50.2 (95% CI 49.5 to 50.9)] | Relatively lower 1/3 EAT-Lancet diet score | Two non-consecutive dietary interviews | BMI class – overweight, BMI class – obese, and chronic disease (adjusted OR) | Age, sex, and educational  level | Higher adherence to the EAT-Lancet score was - associated with lower risk of  BMI class - overweight (OR T3 VS. T1: 0.67, 95% CI: 0.55 to 0.81);  BMI class - obese (OR T3 VS. T1: 0.79, 95% CI: 0.63 to 0.98);  chronic disease (OR T3 VS. T1: 0.56, 95% CI: 0.47 to 0.67) | [87] |
| Damigou 2025 | Greece | 2002 | 20 years | Prospective cohort study | 1,988 | General population/adults, females and males aged 58 (SD 11) years (with CVD event) and 38 (SD 9) years (without CVD event) | Relatively higher EAT-Lancet diet score (range from 0–42) | Relatively lower EAT-Lancet diet score | A 156-item validated semiquantitative FFQ | CVD incidence (adjusted HR) | Age, sex, medical history of hypertension, hypercholesterolemia, diabetes, family history of cardiovascular disease, energy intake, smoking, physical activity, and BMI status | Higher adherence to EAT-Lancet diet was associated with a lower risk of CVD (HR per EAT-Lancet diet score increase: 0.83, 95% CI: 0.77 to 0.90) | [88] |
| Aznar de la Riera 2025 | Spain | 2015–2017 | - | Cross-sectional study | 2,497 | General population/adults, females and males aged ≥ 65 years | Relatively higher 1/4 EAT-Lancet diet score, Q1–Q5 [for growth differentiation factor‑15 & IL-6: mean 81.8 (SD 4.8), mean 90.3 (SD 1.8), mean 96.3 (SD 1.8), and mean 105.4 (SD 5.1)]; [hs-CRP: mean 81.4 (SD 5.2), mean 90.3 (SD 1.8), mean 96.3 (SD 1.8), and mean 104.9 (SD 4.9)] | Relatively lower 1/4 EAT-Lancet score | A computerized,  face-to-face, validated dietary history | Growth differentiation factor-15, IL-6, and hs-CRP concentration [mean percentage differences (95% CI)] | For all indicators: tobacc, alcohol consumption, BMI, energy intake, hours of TV, physical activity, diabetes, CVD, systolic blood pressure, glucose, LDL-C, log-transformed creatinine, N-terminal pro-B-type natriuretic peptid, and high-sen-sitivity cardiac troponin T  - growth differentiation factor-15 is specific to: IL-6;  - IL-6 is specific to: growth differentiation factor-15;  - hs-CRP is specific to: growth differentiation factor-15 and IL-6 | Higher adherence to the EAT-Lancet score was associated with  lower growth differentiation factor-15 (mean percentage differences Q5 VS. Q1: −6.0%, 95% CI: −11% to −1.9%);  lower IL-6 (mean percentage differences Q5 VS. Q1: −9.8%, 95% CI: −15.2% to −4.0%);  lower hs-CRP (mean percentage differences Q5 VS. Q1: −24.9%, 95% CI: −39.2% to −9.7%) | [89] |
| Deng 2025 | China | 2008–2013 | 11 years | Prospective cohort study | 3,742 | General population/adults, females and males aged 40–75 years | Relatively higher EAT-Lancet diet score (range from 0 to 14) | Relatively lower EAT-Lancet diet score | A semi-quantitative 79-item FFQ | α-2-macroglobulin, retinol-binding protein 4, TBC1 domain family member 31, and adenylate kinase 4 [adjusted β value (95% CI)] | Baseline protein abundances or Lancet-protein index | Higher adherence to the EAT-Lancet diet was  - associated with  higher α-2-macroglobulin (β: 0.12, 95% CI: 0.05 to 0.20);  lower retinol-binding protein 4 (β: −0.14, 95% CI: −0.24 to −0.04);  lower TBC1 domain family member 31 (β: −0.11, 95% CI: −0.22 to 0.00);  lower adenylate kinase 4 (β: −0.19, 95% CI: −0.30 to −0.08);  - not associated with hypertension and dyslipidemia | [90] |
| Gong 2025 | USA | 2005–2018 | - | Cross-sectional study | 30,304 | General population/adults, females and males aged ≥ 20 years | Relatively higher 1/3 EAT-Lancet diet score, T1–T3 (33%, 33% and 34% of total population) | Relatively lower 1/3 EAT-Lancet diet score | Planetary Health Diet Index (PHDI), Dietary Inflammatory Index (DII) | COPD (adjusted OR) | Age, gender, education level, marital, family  poverty income ratio, race, obesity, hypertension, diabetes, and high cholesterol | Higher adherence to EAT-Lancet diet was associated with a lower risk of COPD (OR T3 VS. T1: 0.73, 95% CI: 0.59 to 0.90) | [91] |
| Harrison 2025 | Germany | 2022 | 3 months | Quasi-experimental study | 563 | Hospital staff and other regular consumers of cafeteria meals, females and males aged ≥ 20 years | Relatively higher EAT-Lancet diet score (range from 0–42) | Relatively lower EAT-Lancet diet score | Semi-quantitative 116- item FFQ | Mental wellbeing, physical wellbeing (Global Self-Rated Health, physical functioning, and bodily pain), and role Limitations (Role-Physical and Role-Emotional) score [adjusted (Global Self-Rated Health only) β value (95% CIs)] | Age, moderate-intensity physical activity and gender | Higher adherence to EAT-Lancet diet was - associated with  higher mental wellbeing score (β = 0.13, 95% CI: 0.03 to 0.23);  higher Global Self-Rated Health score (β = 0.43, 95%CI: 0.14 to 0.71);  - not associated with   physical functioning score (β = 0.09, 95% CI: −0.07 to 0.25);  bodily pain score (β = −0.22, 95%CI: −0.50 to 0.07);  Role-Physical score (β = −0.16, 95% CI: −0.53 to 0.20);  Role-Emotional score (β = 0.20, 95% CI: −0.21 to 0.62) | [92] |
| Lei 2025 | China | 1997–2015 | 8.1 years | Prospective cohort study | 11,402 | General population/adults, females and males aged ≥ 18 years | Relatively higher 1/4 EAT-Lancet diet score, Q1–Q4 (16–39, 40–44, 45–50, and 51–95) | Relatively lower 1/4 EAT-Lancet score | Self-reported 24-h dietary recalls | Hypertension (adjusted HR) | Age, sex, region, urban/rural, diabetes, systolic blood pressure, diastolic blood pressure, body mass index, smoking, alcohol drinking, education,  physical activity, dietary intake of sodium, potassium, dietary ˙, energy, protein, fat, and carbohydrate | Higher adherence to the EAT-Lancet score was - associated with lower risk of hypertension (HR Q5 VS. Q1:0.79, 95% CI: 0.71 to 0.87) | [93] |
| Li 2025 | USA | 1999–2020 | 10.3 years for all-cause mortality; 7.4 years for CVD mortality (median) | Prospective cohort study | 25,717 | General population/adults, females and males aged 39.5 (SE 0.2) years | Relatively higher 1/2 EAT-Lancet diet score (≥ 24 and < 24) | Relatively lower 1/2 EAT-Lancet diet score | A validated 24-h dietary recall | All-cause mortality, CVD mortality, and heart disease mortality (adjusted HR) | Age, sex, race/ethnicity, marital status,  poverty income ratio, education level, smoking status, alcohol drinking status,  leisure time physical activity, and total energy intake | Lower adherence to EAT-Lancet diet was - associated with a higher risk of all-cause mortality (HR: 1.25, 95% CI: 1.05 to 1.50)  - not associated with a higher risk of  CVD mortality (HR: 1.45, 95% CI: 0.96 to 2.19);  heart disease mortality (HR: 1.00, 95% CI: 0.98 to 2.69) | [94] |
|  | USA | 1999–2020 | - | Cross-sectional study | 25,717 | General population/adults, females and males aged 39.5 (SE 0.2) years | Relatively higher 1/2 EAT-Lancet diet score (≥ 24 and < 24) | Relatively lower 1/2 EAT-Lancet diet score | A validated 24-h dietary recall | Obesity, diabetes, and hypertension (adjusted OR) | Age, sex, race/ethnicity, marital status,  poverty income ratio, education level, smoking status, alcohol drinking status,  leisure time physical activity, total energy intake Diet scores were further  adjusted in models assessing long working hours, and working hours | Lower adherence to EAT-Lancet diet was - associated with a higher risk of  obesity (OR: 1.31, 95% CI: 1.21 to 1.42);  diabetes (OR: 1.34, 95% CI: 1.17 to 1.53);  hypertension (OR: 1.12, 95% CI: 1.03 to 1.20) |  |
| Mansouri 2025 | Iran | 2022 | - | Cross-sectional study | 109 | Recruited from Motahari and Imam Reza  clinics, females and males aged ≥ 18 years | Relatively higher 1/2 EAT-Lancet diet score (> 74 and ≤ 74) | Relatively lower 1/2 EAT-Lancet diet score | A 168-item FFQ | Sarcopenia (adjusted OR) | Age, sex, smoking, fat and energy intake | Higher adherence to EAT-Lancet diet was associated with a lower risk of sarcopenia (OR: 0.25, 95% CI: 0.07 to 0.88) | [95] |
| Qiu 2025 | USA | 2005–2018 | - | Cross-sectional study | 15,865 | General population/adults, females and males aged ≥ 18 years | Relatively higher 1/5 EAT-Lancet diet score, Q1–Q5 (20%, 20%, 19%, 20%, and 21% of total population) | Relatively lower 1/5 EAT-Lancet diet score | A 24-hour dietary recalls | Metabolic dysfunction-associated steatotic liver disease (adjusted OR) | Age, gender, race, education, family poverty income ratio, marital status, smoking, alanine aminotransferase, aspartate  aminotransferase,  diabetes, hypertension, cardiovascular outcomes, and total cholesterol | Higher adherence to EAT-Lancet diet was associated with a lower risk of metabolic dysfunction-associated steatotic liver disease (OR Q5 VS. Q1: 0.61, 95% CI: 0.51 to 0.73) | [96] |
| Samuelsson 2025 | Sweden | 2014–2016 | - | Cross-sectional study | 615 | Participants without dementia, females and males aged 70.5 (SD 0.3) | Relatively higher EAT-Lancet diet score (range from 0–42) | Relatively lower EAT-Lancet diet score | A semi-structured  face-to-face interview | Total cortical thickness, AD-signature cortical thickness, hippocampal volume, small vessel disease, brain age gap, and cognitive performance [adjusted β value or % increase (95% CI)];  Cerebral small vessel disease (adjusted OR) | For all indicators:  sex, education, physical activity,  smoking, BMI, diabetes, and hypertension;  Specific for AD-signature cortical thickness: surface area;  Sepcific for Hippocampal volume:  total intracranial volume | Higher adherence to EAT-Lancet diet was - associated with   higher total mean cortical thickness (β = 0.012, 95% CI: 0.004 to 0.019);  higher AD-signature cortical thickness (β = 0.011, 95% CI: 0.002 to 0.020)  - not associated with  hippocampal volume (β = 6.96, 95% CI: −23.55 to 37.48);  brain age gap (β = −0.118, 95% CI: −0.288 to 0.052);  cognitive performance (β = 0.023, 95% CI: −0.031 to 0.077) | [97] |
| Tang 2025 | Global (185 countries) and USA | 1990–2018 NHANES 2005–2018 (individual) | - | Mixed (global trend analysis + individual cross-sectional) | Global: 185 countries; Individual: 45,460 (NHANES) | General population/adults, females and males | Relatively higher EAT-Lancet diet score (range from 0–130) | Relatively lower EAT-Lancet diet score | Global: PHDI from Global Dietary Database;  Individual: PHDI-US from NHANES 24-h recalls | Global: Incidence, prevalence, mortality, DALYs of CKM diseases;  Individual: CKM syndrome stages, all-cause/CVD mortality | Age, sex, race/ethnicity, education level, marital status, smoking and drinking status, physical activity, and NHANES cycles | Higher PHDI scores were generally associated with lower  - CKM disease burdens, although these  associations varied by disease subtype;  In individual-level analysis, each 10-point increase in PHDI was linked to a 13.7% reduction in stage 3/4 CKM syndrome risk, an 11.1% reduction in stage 4 CKM syndrome risk, and lower incidences and mortality rates for cardiovascular diseases, metabolic diseases, and chronic kidney disease | [98] |
| Vargas-Quesada 2025 | Latin America (8 countries: Argentina, Brazil, Chile, Colombia, Costa Rica, Ecuador, Peru, Venezuela) | 2014–2015 | - | Cross-sectional multicentric study | 6,683 | General population/adults, females and males aged 15–65 years | Relatively higher 1/5 EAT-Lancet diet score, Q1–Q5 (PHDI, ELDI, WISH, and HSDI) | Relatively lower 1/5 EAT-Lancet diet score | Two nonconsecutive 24-h dietary recalls | Overweight/obesity (adjusted PR) | Age, sex, total energy intake, country, socioeconomic status, and physical activity level | Higher adherence to EAT-Lancet diet was - not associated with overweight/obesity  PHDI (PR Q5 VS. Q1: 1.001, 95% CI: 0.999 to 1.004);  ELDI (PR Q5 VS. Q1: 1.000, 95% CI: 0.994 to 1.006);  WISH (PR Q5 VS. Q1: 1.000, 95% CI: 0.998 to 1.001);  HSDI (PR Q5 VS. Q1: 1.002, 95% CI: 0.986 to 1.017) | [99] |
| Wei 2025 | USA | 1993–2001 | 8.84 years | Prospective cohort study | 101,755 | General population/adults, females and males aged 55–74 years | Relatively higher 1/4 EAT-Lancet diet score, Q1–Q4 (≤ 18, 19–21, 22–24, and ≥ 25) | Relatively lower 1/4 EAT-Lancet diet score | the Baseline Questionnaire and the Diet History Questionnaire | Renal cancer (adjusted HR) | Age, sex, race, BMI, smoking, drinking status, pack-years cigarettes smoking, alcohol consumption, history of hypertension, history of diabetes, ibuprofen use, family history of renal cancer, and energy intake from diet | Higher adherence to EAT-Lancet diet Higher adherence to EAT-Lancet diet was associated with lower risk of renal cancer (adjusted HR: 0.65, 95%CI: 0.49–0.88) | [100] |
| Ye 2025 | UK | 2006–2010 | 13.15 years (median) | Prospective cohort study | 187,558 | General population/adults, females and males aged 37–73 years | Relatively higher 1/4 EAT-Lancet diet score, Q1–Q4 (≤ 8, 8, 9, and ≥ 10) | Relatively lower 1/4 EAT-Lancet diet score | the Oxford Web, a web based 24-h recall questionnaire | Inflammatory bowel disease, Crohn’s disease, ulcerative colitis (adjusted HR) | Age, sex, race, Townsend Deprivation Index, university degree, BMI, physical activity, sleep duration, alcohol drinking, and smoking status | Higher adherence to EAT-Lancet diet was - not associated with  inflammatory bowel disease (HR Q4 VS. Q1: 0.87, 95% CI: 0.75 to 1.02);  ulcerative colitis (HR Q4 VS. Q1: 0.88, 95% CI: 0.73 to 1.06);  Crohn’s disease (HR Q4 VS. Q1: 0.79, 95% CI: 0.62 to 1.02) | [101] |
| Zhang 2025 | UK | 2006–2010 | 12.58 years | Prospective cohort study | 7,525 | General population/adults, females and males aged 37–73 years | Relatively higher 1/3 EAT-Lancet diet score, T1–T3 (5–8, 91, and 10–14) | Relatively lower 1/3 EAT-Lancet diet score | the Oxford WebQ | Diabetic microvascular complications (adjusted HR) | Age, sex, ethnicity, diabetes duration, sex, ethnicity, Townsend Deprivation Index, university education, household income, smoking and drinking status, body mass index, physical activity at goal, total cholesterol, triglycerides, low-density lipoprotein cholesterol, hypertension, dyslipidemia, chronic kidney disease, coronary heart disease, stroke, insulin therapy, noninsulin therapy, antihypertensive drugs and cholesterol-lowering drugs | Higher adherence to EAT-Lancet diet was - associated with  diabetic microvascular complications (HR T3 VS. T1: 0.76, 95% CI: 0.64 to 0.88) | [102] |

Abbreviations: BMI, Body mass index; BW, body weight; CI, confidential interval; hs–CRP, C–reactive protein; COPD, chronic obstructive pulmonary disease; CVD, cardiovascular disease; FFQ, food frequency questionnaire; HDL, high-density lipoprotein; HDL-c, high-density lipoprotein–cholesterol; non-HDL-c, non-high-density lipoprotein–cholesterol; HOMA-IR, Homeostatic Model Assessment for Insulin Resistance; HR, hazard ratio; IL-6, Interleukin-6; LDL-c, low-density lipoprotein–cholesterol; OR, odds ratio; Q, quantile; RR, relative risk; SD, standard deviation; T, tertile; T2D, Type 2 diabetes; TNF-α, tumor necrosis factor-α

**Table S4. Summary of articles investigating the EAT-lancet planetary health diet and environmental sustainability**

| **Study** | **Country** | **Assessment period** | **Study type** | **Cohort size, n** | **Study subject** | **Study diet** | **Compared diet** | **Dietary data sources or assessment method** | **Calculation methods of planetary health indicators** | **Outcomes** | **Adjustments** | **Main findings** | **Reference** |
| --- | --- | --- | --- | --- | --- | --- | --- | --- | --- | --- | --- | --- | --- |
| **Studies included in the systematic review and meta-analysis** | | | | | | | | | | | | |  |
| Cacau 2021 | Brazil | 2008–2010 | Cross-sectional study | 14,779 | General population/adults, females and males, aged 35–74 years | Relatively higher 1/5 EAT-Lancet diet score, Q1–Q5 (range from 0–150) | Relatively lower 1/5 EAT-Lancet diet score | A 114-item FFQ | Database: Environmental Footprints of Food and Culinary Preparations Consumed in Brazil | Greenhouse gas emissions | Age and sex | Higher adherence to the EAT-Lancet diet was associated with  - lower greenhouse gas emissions (β = –1.3075, 95% CI: –1.5603 to –1.0546, *P* < 0.001) | [103] |
| Kesse-Guyot 2021 | France | 2009–2014 | Cross-sectional study | 29,210 | General population/adults, females and males aged 53.5 (SD 14.0) years | Relatively higher 1/5 EAT-Lancet diet score, Q1–Q5 (≤ 4.35, 4.35–21.46, 21.46–37.67, 37.67–59.74, and > 59.74) | Relatively lower 1/5 EAT-Lancet score | A 264-item FFQ | The DIALECTE tool and the partial ReCiPe index | Cumulative energy demand, greenhouse gas emissions and land occupation [  adjusted mean value (95% CI)] | Daily energy intake | Higher adherence to the EAT-Lancet diet was associated with  - lower greenhouse gas emissions (56%); - lower cumulative energy demand (31%); - lower land occupation (54%) | [104] |
| Laine 2021 | 10 European countries: Denmark, France, Germany, Greece, Italy, the Netherlands, Norway, Spain, Sweden, and the UK | 1991–2000 | Cross-sectional study | 443,991 | General population/adults, females and males aged 18–99 years | Relatively higher EAT-Lancet diet score | Relatively lower EAT-Lancet diet score | Semi-quantitative FFQ | The SHARP indicators Database | Greenhouse gas emissions and land use (adjusted HR) | Age at recruitment (continuous), marital status, education, physical activity, smoking status, and body-mass index (BMI continuous) | Higher adherence to the EAT-Lancet diet was associated with - lower greenhouse gas emissions (50%); - lower land use (up to 62%) | [105] |
| Tepper 2022 | Israel | 2018–2020 | Cross-sectional study | 525 | General population/adults, females and males aged 20–66 years | Relatively higher 1/3 EAT-Lancet diet score, T1–T3 | Relatively lower 1/3 EAT-Lancet diet score | A 116– item FFQ database from the Israeli Ministry of Health | Land footprint: FAOSTAT  Water footprint: A database on virtual water (Access in: doi: 15:401–15. doi: 10.1007/s10021-011-9517-8)  Carbon footprint: two types of data sources (Access in: doi: 10.1021/es4025113) and life cycle assessment database in Israel | Land footprint, water footprint, and greenhouse gas footprints | - | Higher adherence to the EAT-Lancet diet was associated with - lower land use; - lower greenhouse gas emissions; - higher water use | [106] |
| Vallejo 2022 | Germany | 1985–2021 (ongoing) | Cross-sectional study | 298 | 298 participants, aged 16.7 years | Relatively higher 1/3 EAT-Lancet diet score, male T1–T3 [median 9 range (7–9), median 10 range (10–10), and median 11 range (11–14)], female T1–T3 [median 9 range (6–10), median 11 range (11–11), and median 12 range (12–15)] | Relatively lower 1/3 EAT-Lancet score | 3-d weighted dietary record (2–5 sets) | The SHARP indicators database | Greenhouse gas emissions and land use [β value (95% CI)] | Age, sex, birth weight, gestational age, socioeconomic factors, follow-up time, and total energy intake | Higher adherence to the EAT-Lancet diet was associated with - lower greenhouse gas emissions (β = –0.22, 95% CI: –0.30, –0.14); - lower land use (β = –0.40, 95% CI: –0.52, –0.29) | [9] |
| Colizz 2023 | New Zealand | 1993–1997 | Cross-sectional study | 35,496 | General population/adults, females and males aged 20–70 years | Relatively higher 1/4 EAT-Lancet diet score, Q1–Q4 (32–66, 67–73, 74–79, and 80–117) | Relatively lower 1/4 EAT-Lancet diet score | A 178-item semi-quantitative FFQ | Life cycle assessment database (Dutch) | Greenhouse gas emissions, land use, blue water use, freshwater eutrophication, marine eutrophication, and terrestrial acidification [mean value (SD), β value (95% CI), and adjusted percent difference (95% CI)] | Age, sex, and energy intake, educational level, smoking, alcohol consumption, physical activity, and energy intake, the cardiovascular risk factors BMI, cholesterol level, and hypertension | Higher adherence to the EAT-Lancet diet was associated with - lower greenhouse gas emissions 2.4% (95% CI, −5.0 to 0.2); - lower LU 3.9% (95% CI, −5.2 to −2.6) - lower freshwater eutrophication 0.5% (95% CI, −2.6 to 1.6); - lower marine eutrophication 3.3% (95% CI, −5.8 to −0.8); - lower terrestrial acidification 7.7% (95% CI, −10.8 to −4.6); - higher blue water use 32.1 % (95% CI, 28.5 to 35.7) | [12] |
| Mangone 2023 | Italy | 1993–1998 | Cross-sectional study | 47,749 | General population/adults, females and males aged 50.5 years (mean) | Relatively higher 1/4 EAT-Lancet diet score, Q1–Q4 (4–9, 10, 11, and 12–14) | Relatively lower 1/4 EAT-Lancet diet score | A 148-item face-to-face semi-quantitative FFQ | The SHARP indicators database | Greenhouse gas emissions and land use (median (Q1, Q4)] | Age, sex, BMI, physical activity, education, smoking | Higher EAT-Lancet diet score was associated with - higher greenhouse gas emissions; - higher land use | [107] |
| Ye 2023 | Singapore | 1993–1998 | Cross-sectional study | 63,257 | General population/adults, females and males aged 45–74 or 55.0 (IQR 49.0–62.0) years | Relatively higher 1/5 EAT-Lancet diet score, Q1–Q5 (40–46, 49–52, 54–57, 59–62, 65–71) | Relatively lower 1/5 EAT-Lancet diet score | A 165-item face-to-face semi-quantitative FFQ | The China Health and Nutrition Survey, the Water Footprint Network Database and the FAOSTAT | Greenhouse gas emissions, land use and total water footprint [adjusted β value (95% CI)] | Age, sex, and total energy intake | Higher adherence to the EAT-Lancet diet was associated with - lower greenhouse gas emissions (β = −0.13, 95% CI: −0.14 to −0.12); - higher land use (β = 0.12, 95% CI: 0.11 to 0.13); - higher water footprint (β = 0.29, 95% CI: 0.28 to 0.31) | [20] |
| Bui 2024 | USA | 1986 and 1991 | Population-based prospective cohort study | 206,404 | General population/adults, females and males, aged 25–75 years | Relatively higher 1/5 EAT-Lancet diet score, Q1–Q5 (58–66, 67–74, 73–80, 78–86, and 86–97) | Relatively lower 1/5 EAT-Lancet diet score | ˃ 130-item self-administered FFQs | Nutrition Questionnaire Service waist | Greenhouse gas emissions, fertiliser needs, cropland needs, and irrigation water needs | Daily calorie intake | Adopting the EAT-Lancet diet was associated with - lower greenhouse gas emissions (-28.9%);  - lower fertiliser needs (-21.3%); - lower cropland needs (-53.7%);  - lower irrigation water needs (-13.5%) | [24] |
| Chen 2024 | China | 1997, 2000, 2004, 2006, 2009, and 2011 | Prospective cohort study | 9,364 | General population/adults, females and males, aged ≥ 45 years | Relatively higher 1/4 EAT-Lancet score, Q1–Q4 (median 44, median 50, median 55, median 62) | Relatively lower 1/4 EAT-Lancet score | 3 day 24-h dietary recalls | Chinese Food Life Cycle Assessment Database (CFLCAD) | All-cause mortality (adjusted HR) | Total energy intake | Higher adherence to the EAT-Lancet score was  - associated with lower greenhouse gas emissions (HR Q4 vs. Q1: 0.22, 95% CI: 0.19 to 0.25, *P* < 0.0001);  - associated with lower total water footprint (HR Q4 vs. Q1: 0.53, 95% CI: 0.49 to 0.56, *P* < 0.0001);  - associated with lower and appropriation (HR Q4 vs. Q1: 0.27, 95% CI: 0.23 to 0.30, *P* < 0.0001) | [26] |
| Frank 2024 | USA | 2015–2018 | Cross-sectional study | 8,128 | General population/adults, females (non-pregnant or lactating) and males aged ≥ 20 years | Relatively higher 1/5 EAT-Lancet diet score, Q1–Q5 (18–125) | Relatively lower 1/5 EAT-Lancet diet score | 24-h dietary recalls and an unannounced phone call three to ten days after the initial face-to-face interview | The *dataFRIENDS* (National Health and Nutrition Examination Survey) | Greenhouse gas emissions [adjusted mean value (SE)] | Total energy intake | Higher adherence to the EAT-Lancet diet was associated with - lower greenhouse gas emissions (mean: –0.4, 95% CI: –0.5 to –0.3) | [108] |
| Shojaei 2024 | Iran | 2017–2020 | Cross-sectional study | 6,465 | General population/adults, females and males aged 30–70 years | Relatively higher 1/4 EAT-Lancet diet score, Q1–Q4 [mean 41.5 (SD 2.9), mean 48.6 (SD 1.6), mean 54.5 (SD 1.8), and mean 64.2 (SD 5.3)] | Relatively lower 1/4 EAT-Lancet score | A 130-item FFQ | BCFNDOUBLEPYRAMIDDATABASE | Carbon footprint, and water footprint [adjusted β value (95% CI)] | Age and sex | Higher adherence to the EAT-Lancet diet would be associated with  - lower carbon footprint (β = −0.121, 95% CI: −0.001 to 0.000);  - lower water footprint (β = −0.055, 95% CI: −0.077 to −0.034) | [44] |
| Teixeira 2024 | Portugal | 2015–2016 | Cross-sectional study | 1,153 | Children and adolescents, aged 3–17 years | Relatively higher EAT-Lancet diet score | Relatively lower EAT-Lancet diet score | Two non-consecutive 24-h recalls | SHARP-Indicators database (including life cycle assessment data) | Children- greenhouse gas emissions, children-land use, adolescents- greenhouse gas emissions, and adolescents-land use | Total grams of the remaining food | Adopting the EAT-Lancet diet was associated with - lower children- greenhouse gas emissions (β = –0.243, 95% CI: –0.435 to –0.051);  - lower children-land use (β = –0.512, 95% CI: –0.706 to –0.317);  - lower adolescents- greenhouse gas emissions (β = –0.203, 95% CI: –0.412 to 0.006);  - lower adolescents-land use (β = –0.461, 95% CI: –0.816 to –0.105) | [47] |
| Jovanović 2025 | Croatia | 2023–2024 | Cross-sectional study | 224 | University students, females and males, aged 19–27 years | Relatively higher 1/4 EAT-Lancet diet score, Q1–Q4 (20-25,46-55, 56-65, and 66-100) | Relatively lower 1/4 EAT-Lancet diet score | 98-item semi-quantitative Food Frequency Ques-  tionnaire (FFQ) | SHARP Indicators Database (Version 2) [Dataset]; DANS Data Station Life Sciences: Dubai, United Arab Emirates, 2019.; SU-EATABLE LIFE: A  Comprehensive Database of Carbon and Water Footprints of Food Commodities; Figshare: London, UK, 2021. | Carbon footprint, water footprint, ecological footprint | gender, level of study , body mass index classification, and physical activity level | Higher adherence to the Planetary Health Diet was associated with  - lower carbon footprint (β = −7.94, p < 0.001)  - lower water footprint (β = −13.88, p < 0.001)  - lower ecological footprint (β = −3.15, p < 0.001) | [109] |
| Wu 2025 | China | 1997–2015 | Cross-sectional study | 14,652 (new-onset cardiometabolic diseases); 15,318 (all-cause mortality) | General population/adults, females and males aged ≥ 18 years | Relatively higher 1/4 EAT-Lancet diet score, Q1–Q4 (PHDI and WISH) | Relatively lower 1/4 EAT-Lancet diet score | 3-day consecutive 24-h dietary recalls, and consumption of cooking oils and condiments was determined at the household level using a food weighing method | Based on a previously established method (doi: 10.1016/j.jenvman.2019.03.106) | Greenhouse gas emissions [adjusted mean value (95% CI)] | Age, sex, BMI, region, urbanization index, educational level, physical activity, baseline hypertension, smoking status, alcohol intake, and total energy intake | Higher adherence to the EAT-Lancet diet was associated with - lower greenhouse gas emissions (PHDI, 17%); (WISH, 15%) | [71] |
| Zhan 2025 | US | 2005–2018 | Cross-sectional study | 27,181 | General population/adults, females and males aged > 21 years | Relatively higher 1/5 EAT-Lancet diet score, Q1–Q5 | Relatively lower 1/5 EAT-Lancet score | Two 24-hour dietary recall | the Food Recall Impacts on the database of Food Recall Impacts on the Environment for Nutrition and Dietary Studies (dataFRIENDS) version 2 | Greenhouse gas emissions [adjusted mean value (95% CI)] and [adjusted β value (95% CI)] | - | Higher adherence to the EAT-Lancet diet was associated with - lower greenhouse gas emissions (25%); (β = –0.59, 95% CI: –0.73 to –0.45) | [72] |
| **Studies included in the systematic review only** | | | | | | | | | | | | |  |
| Bayer 2020 | Spain | 2019–2019 | Comparative analysis | - | General population of all ages | EAT-Lancet diet  a. Planetary Health Diet 2221 (pre-COVID period)  b. Planetary Health Diet 1970 (COVID-Lockdown period) | a. pre-COVID diets for March 2019  b. pre-COVID diets for April 2019  c. COVID diet  d. alternative diet based on National Dietary Guidelines  e. alternative diet based on Planetary Health Diet | 2018 food- away-from-home consumption (MAPA, Ministerio de Agricultura, 2019) | Blue water footprint and land use (Access in: <https://doi.org/10.1016/j.coesh.2019.11.001>)  Food loss and waste (Access in: <https://doi.org/10.1016/j.foodpol.2018.08.007>) | Global warming potential, blue water footprint and land use | - | Higher adherence to the EAT-Lancet diet during Pre-COVID period was associated with  - lower global warming potential [Planetary Health Diet 2221(21.90)< National Dietary Guidelines; 2221(28.20)<APR19(33.70)<MAR19(33.50)] - lower blue water footprint [Planetary Health Diet 2221(2.22)< National Dietary Guidelines; 2221(2.24)<APR19(2.23)<MAR19(2.24)] - lower land use [National Dietary Guidelines 2221(33.00)<COVID(38.60)<APR19(36.40)<MAR19(36.50)] Higher adherence to the EAT-Lancet diet during COVID–Lockdown period was associated with  - lower global warming potential [Planetary Health Diet 1970(21.20)< National Dietary Guidelines 1970(24.90)<COVID(34.00)]; - lower blue water footprint [Planetary Health Diet 1970(1.98)< National Dietary Guidelines 1970(1.99)<COVID(2.36)]; - lower land use [Planetary Health Diet 1970(31.40)< National Dietary Guidelines 1970(29.30)< Planetary Health Diet 2221(32.80)] | [110] |
| Bozeman III 2020 | USA | - | Modelling study | - | General population of all ages | Relatively higher EAT-Lancet diet score | Relatively lower EAT-Lancet diet score | Food Commodity Intake Database (What We Eat in America-Food Commodity Intake Database) | Life cycle assessment database | Land, greenhouse gas andwater | Baseline consumption rate | Adopting the EAT-Lancet diet would be associated with  - lower land food consumption impacts (36.1%); - lower greenhouse gas emissions food consumption impacts (31.8%); - lower water food consumption impacts (30.4%) | [111] |
| Forber 2020 | UK | 1942–2050 | Modelling study | - | General population of all ages | EAT-Lancet diet | Current diet | UK National Food Survey (1974-2016, including takeaway data) | McCance and Widdowson’s The Composition of Foods Integrated dataset P burden entering wastewater treatment works = per capita dietary P consumption was multiplied by UK population | Dietary phosphorus burden, phosphorus burden entering wastewater treatment works | - | Higher adherence to the EAT-Lancet diet would be associated with - higher phosphorus entering wastewater treatment works (35%) in 2050; - higher dietary phosphorus burden (67%) | [112] |
| Prag 2020 | Denmark | 2013–2030 | Modelling study | - | General population of all ages | EAT-Lancet diet | Current diet | The EAT-Lancet Commission’s recommendations (Access in: https://www.thelancet.com/journals/lancet/article/PIIS0140-6736(18)31788-4/abstract) FAOSTAT (2020) | SEGES (2019) | Greenhouse gas emissions | - | Higher adherence to the EAT-Lancet diet would be associated with - lower greenhouse gas emissions [13.6–20.2 Mt CO2e (58.2 to 86.5%)] | [113] |
| Semba 2020 | Global | 2014–2017 | Modelling study | - | General population of all ages | EAT-Lancet diet | Current diet | 2014–2017 food balance sheets from FAO | Mendeley Data (https://data.mendeley.com/datasets/g8n8w8snmj/3) | Greenhouse gas emissions | - | Higher adherence to the EAT-Lancet diet would be associated with - 12-283% greenhouse gas emissions increase in 36 low- and middle-income countries (to 1.43 GtCO2e yr^−1^) | [114] |
| Pee 2021 | Indonesia | 2018 and 2019 | Modelling study | - | General population of all ages | EAT-Lancet diet | 13 dietary scenarios (2 current, 7 optimized, 3 increasingly plant-based, 2 EAT-Lancet) | Current Indonesian diet modelled from loss-adjusted food availability from food balance sheets | Study model developed for Kim et al. (Access in: https://www.sciencedirect.com/science/article/pii/S0959378018306101) | Greenhouse gas emissions, and water footprints | - | Higher adherence to the EAT-Lancet diet would be associated with - lower greenhouse gas emissions; - lower water footprint | [115] |
| Kassem 2020 | Denmark | 2009–2013 | Comparative analysis | - | General population of all ages | EAT-Lancet diet | Insects_REF diet | FAOSTAT (2019) | Average water footprints per ton of commodity for Denmark (Access in: https://www.sciencedirect.com/science/article/pii/S0959378018306101)  water footprints data for average seafood production worldwide  (Access in: https://digitalcommons.unl.edu/cgi/viewcontent.cgi?article=1078&context=wffdocs)  water footprints data specifications are specifically available on wheat  (Access in: https://www.sciencedirect.com/science/article/pii/S0048969715304666?via%3Dihub) | Water footprints | - | Higher adherence to the EAT-Lancet diet was associated with - lower water footprint [682 l/cap/d (26%)] | [116] |
| Kidd 2021 | New Zealand | 2002 and 2008–2009 | Modelling study | - | 210–237 households | EAT-Lancet diet | a. Current diet b. Healthy diet (2015 New Zealand Eating and Activity Guidelines) | Current diet: Survey data from the most recent NZ National Nutrition Survey (2008/2009) Healthy diet: 2015 New Zealand Eating and Activity Guidelines EAT-Lancet diet : Modelled from the EAT-Lancet ‘Planetary Diet’ guidelines | A recently published (2020) life cycle assessment database | Greenhouse gas emissions | - | Adopting the EAT-Lancet diet would be associated with - lower greenhouse gas emissions on the 20–year global warming potential (current diet: 597 kgCO2e healthy diet: 452 kgCO2e EAT-Lancet diet: 263 kgCO2e); - lower greenhouse gas emissions on the 100–year global warming potential (current diet: 356 kgCO2e healthy diet: 326 kgCO2e EAT-Lancet diet: 233 kgCO2e) | [117] |
| Philippidis 2021 | Global | 2011 | Modelling study | - | General population of all ages | EAT-Lancet diet | Business as usual scenario diet (2050) | Modular Applied GeNeral Equilibrium Tool nutrition module (access in: https://edepot.wur.nl/292724) and satellite data based on FAO nutritive factors for different food-types | Global trade analysis project database | Blue water, agricultural land, and greenhouse gas emissions | - | Adopting the EAT-Lancet diet by 2050 would be associated with Global: - a reduction of agricultural land use (8%);  - a reduction of greenhouse gas emissions (9%); - a reduction of permanent pastureland (21%); - an increase in blue water (5%); - an increase in cropland requirements (5%)  Regional: - a reduction of blue water consumption in Oceania and the EU and agricultural land savings in Latin America and Oceania; - a increases in cropland the EU and North Africa; - a reduction in greenhouse gas emissions in all regions, particularly in Latin America | [118] |
| Feng 2021 | China | - | Modelling study | - | General population of all ages | EAT-Lancet diet | Current diet | China Statistical Yearbook (National Bureau of Statistics of China, 1998–2020 | FAOSTAT | Greenhouse gas emissions | - | Higher adherence to the EAT-Lancet diet would be associated with - lower greenhouse gas emissions (32%) | [73] |
| Vanham 2021 | Nine countries (Spain, France, Italy, Greece, Turkey, Egypt, Tunisia, Algeria and Morocco) | 2011–2013 | Comparative analysis | - | - | EAT-Lancet diet | Mediterranean diet | FAO food balance sheets (2011–2013) | FAO food balance sheets (2011–2013) and the international water footprints database for crops and crop products and for livestock products | Water footprints | - | Adopting the EAT-Lancet diet was associated with - a reduction of 17–48% water footprints at a global level for all mediterranean nations | [119] |
| Vitale 2021 | Italy | - | Observational study | - | - | Relatively higher EAT-Lancet diet score | Relatively lower EAT-Lancet diet score | FAO food balance sheets | Life cycle assessment database | Greenhouse gas emissions | - | Adopting the EAT-Lancet diet was associated with - a reduction of the nearly 50% water footprints | [120] |
| Franco 2022 | Spain | - | Comparative analysis | - | General population of all ages | EAT-Lancet diet | Spanish dietary pattern | 2019 Household Consumption Survey of the Ministry of Agriculture, Fisheries and Food, BEDCA food composition database | Life cycle assessment database | Carbon footprint and water footprints | - | Higher adherence to the EAT-Lancet diet was associated with - lower carbon footprint (2.13 kgCO2eq·person^−1^·day^−1^); - lower water footprints (3056 L·person^−1^·day^−1^) | [121] |
| Cleghorn 2022 | New Zealand | - | Modelling study | - | General population of all ages (devided into four groups: Ma ̄ori males, Ma ̄ori females, non-Ma ̄ori males and non-Ma ̄ori females) | Relatively higher EAT-Lancet diet score | Relatively lower EAT-Lancet diet score | Adult Nutrition Survey (2008/09) | New Zealand Ministry for the Environment | Greenhouse gas emissions | - | Adopting the EAT-Lancet diet would be associated with  - lower greenhouse gas emissions (37%–67%) | [122] |
| Dhar 2022 | India | 2014–2050 | Modelling study | - | General population of all ages | EAT-Lancet diet | Current diet | Food balance sheets from FAO | The religion-sensitive N-Calculator method developed by Dhar et al. (Access in: https://www.mdpi.com/2072-6643/13/6/1926) | Food Nitrogen Footprint | - | Higher adherence to the EAT-Lancet diet would be associated with - lower food Nitrogen footprint (13%) | [123] |
| Jha 2022 | India | 2050 | Modelling study | - | General population of all ages | EAT-Lancet diet | Current diet | Future trends in food demand based on A cross-country regression (Access in: https://journals.plos.org/plosone/article?id=10.1371/journal.pone.0139201) | Model of Agricultural Production and its Impacts on the Environment | Greenhouse gas emissions, land use and water use | - | Higher adherence to the EAT-Lancet diet would be associated with - lower greenhouse gas emissions (30%); - lower annual blue water use (38%); - lower pasture area (50%) | [124] |
| Read 2022 | USA | 2021 | Modelling study | - | - | EAT-Lancet diet score | Current diet | USEEIO version 2.0 input-output model | FAOSTAT | Land footprint, biodiversity footprint | - | Higher adherence to the EAT-Lancet diet would be associated with - lower land footprint (44.8%); - lower threats to biodiversity footprint (29.7%) | [125] |
| Sun 2022 | 54 high-income nations | - | Modelling study | - | - | EAT-Lancet diet | High-income nations’ diet | Food balance sheets from FAO (2010) | FAOSTAT and Food and Agriculture Biomass Input–Output dataset | Greenhouse gas emissions, carbon sequestration | Energy intake | Higher adherence to the EAT-Lancet diet would be associated with (per year) - lower greenhouse gas emissions produced by agriculture (61.5% or 0.75GtCO2); - higher carbon sequestration (mean:98.3, 95%CI: 55.6–143.7 GtCO2) | [126] |
| Sun 2022 | European Union, UK, Ukraine, Russia | - | Modelling study | - | - | EAT-Lancet diet | Current diet | Food balance sheets from FAO and the EAT-Lancet diet per person per day scaled by population in the year 2010 | Food and Agriculture Biomass Input–Output dataset | Blue water, greenhouse gas emissions, carbon sequestration | - | Adopting the EAT-Lancet diet in the European Union and the United Kingdom alone would almost compensate for all production deficits from Russia and Ukraine, which would be associated with (per year) - higher blue water use (4.1 Gm3); - higher greenhouse gas emissions (0.22 GtCO2e); - higher carbon sequestration (17.4 GtCO2e) | [127] |
| Taherzadeh 2022 | USA | 2017–2018 | Observational study | 7,418 | General population/adults, children and infants, females and males | EAT-Lancet diet | Current diet | 24-h dietary recalls (twice) and National Health and Nutrition Examination Survey data (2017–2018) | Poore and Nemecek’s meta- analysis of food system environmental foot–printing studies | Greenhouse gas footprints, land footprints, and water footprints | Age and sex | Adopting the EAT-Lancet diet was associated with - a reduction of the 70.3% overshoot of the dietary greenhouse gas footprint; - a reduction of the 63.1% overshoot of the dietary water footprints; - a reduction of the 73.5% overshoot of the dietary land footprint | [128] |
| Tucci 2022 | Italy | - | Comparative analysis | - | - | EAT-Lancet diet | Italian Dietary Guidelines | Italian National Food Consumption Survey Italian National Food Consumption Survey 2005–06 data [software for nutritional assessment (MètaDieta professional 4.1.1 METEDA Srl-Roma, Italy)] | Multilevel carbon and water footprint dataset of food commodities | Carbon footprint, water footprints | - | Adopting the EAT-Lancet diet was associated with (compared to IDG): - lower carbon footprint (2.82 ± 1.07 and 3.74 ± 0.92 kg CO2 equivalents/day; *P* < 0.05);  - not associated with the water footprints | [129] |
| Tuninetti 2022 | Global | 1961–2018 | Comparative analysis | - | - | EAT-Lancet diet | Current diet | Food balance sheets from FAOSTAT | CWASI database | Water footprints | - | Adopting the EAT-Lancet diet was associated with - a net reduction of 12% water footprints at a global level (172 countries) | [130] |
| Bellamy 2023 | UK | - | Comparative analysis | 113 | Members of Community Supported Agriculture schemes and the general population (Community Supported Agriculture n = 46, control group n = 67) | EAT-Lancet diet | Community Supported Agriculture group diet | Intake24 software， Semi-structured interviews (three consecutive days) | The 2019 EAT Lancet Commission dietary guidelines | Greenhouse gas emissions | - | Higher adherence to the EAT-Lancet diet was associated with - lower greenhouse gas emissions (28%) | [131] |
| Clay 2023 | Australia | - | Case report study | - | A 71‐year‐old male | EAT-Lancet diet | Current diet | Current diet: 2011–2013 Australian Health Survey  EAT-Lancet diet: the SYMBIOTiC (SYMptom, microBIOme and dieT in chronic kidney) study | Gold standard metric of global warming potential | Climate footprints (carbon dioxide equivalents (CO2e) ) | dietary quantities for the Planetary Health Diet, usual renal diet and plant‐based renal diet | Adopting the EAT-Lancet diet was associated with - lower production of CO2e per day than the current Australian diet (1.04 kg per day 56%) | [132] |
| Gatto 2023 | Global | 2030 | Modelling study | - | General population of all ages | EAT-Lancet diet | BAU scenario diet | The FAO global flows of food loss and waste database (2019) and literature review | Modular Applied GeNeral Equilibrium Tool | Land use and greenhouse gas emissions | - | Adopting the EAT-Lancet diet would be associated with - a reduction of the expansion of agricultural land use globally (3.8%–3.2%); - an increase in greenhouse gas emissions globally (1.7%, equal to 961 million tonnes of CO2 equivalents); - an increase in land demand in sub-Saharan Africa (2.4%, 100 million hectares) | [133] |
| Geibel 2023 | EU27 (focused on Ireland and Denmark) | 2050 | Modelling study | - | General population of all ages | EAT-Lancet diet | Benchmark diet 2050 | Modular Applied GeNeral Equilibrium Tool model (global trade analysis project 10 database) | Modular Applied GeNeral Equilibrium Tool | Greenhouse gas emissions | - | Adopting the EAT-Lancet diet would be associated with - a reduction of total agricultural emissions (26.4% in Ireland, 21.7% in Denmark, and 29.3% in EU-27); - a reduction of Methane emissions (more than 30% in Ireland and Denmark, and 41.0% in EU-27); - a reduction of Nitrous oxide emissions (19.4% in Ireland, 10.5% in Denmark, and 17.6% in EU-27); - a reduction of total emissions (including livestock production) (9.0% in Ireland, 4.8% in Denmark, and 3.7% in EU-27); - an increase in CO2 emissions (1.8% in Ireland, and 5.8% in Denmark) | [134] |
| Kopainsky 2023 | Europe | 2023–2050 | Modelling study | 32 | Countries in Europe | EAT-Lancet diet | - | The Swiss national dietary guidelines | FAOSTAT | Greenhouse gas emissions | - | Higher adherence to the EAT-Lancet diet would be associated with - lower greenhouse gas emissions | [135] |
| Navarre 2023 | Global | - | Comparative analysis | 204 nations | General population of all ages | EAT-Lancet diet | BAU scenario diet | Data S1.csv (Access in: https://doi.org/10.5281/  zenodo.7405302) | FAOSTAT | Land use | - | Higher adherence to the EAT-Lancet diet was associated with - lower land use (9%) | [136] |
| Neff 2023 | USA | - | Comparative analysis | - | General population/adults who attempted weight loss | EAT-Lancet diet | Weight‐loss diets | 1‐week meal plans | United States Department of Agriculture food composition databases | Greenhouse gas emissions, water footprints | - | Higher adherence to the EAT-Lancet diet was associated with - lower greenhouse gas emissions (4.4 times the EAT‐Lancet 2050 target); - lower water footprints (49%) | [137] |
| Schön 2023 | Germany | - | Observational study | - | - | EAT-Lancet diet | Current Diet | German Nutrition Society’s recommendations | Regional data from the state of Hesse, Germany | Land consumption | - | Adopting the EAT-Lancet diet was associated with - a reduction in the pastureland consumption (14%–65%); - a reduction in the cropland consumption (14%–47%) | [138] |
| Stewart 2023 | UK | 1986–2017 | Modelling study | - | General population of all ages | EAT-Lancet diet | Current diet | Food balance sheets from FAO | FAOSTAT | Greenhouse gas emissions | - | Higher adherence to the EAT-Lancet diet would be associated with - lower greenhouse gas emissions (32%) | [139] |
| Stone 2022 | USA | - | Modelling study | - | General population of all ages | EAT-Lancet diet | Current diet | FoodCarbonScope^TM^ | FoodCarbonScope^TM^ | Global warming potential, energy consumption, water use and land use | - | Higher adherence to the EAT-Lancet diet would be associated with - lower global warming potential (–47%); - lower energy consumption (–44 %); - lower water use (–68 %); - lower land use (–83 %) | [140] |
| Colombo 2023 | Sweden | 2010–2011 | Modelling study | 1,797 | General population/adults, females and males aged 18–80 years | Relatively higher 1/3 EAT-Lancet diet score, T1–T3 (< 4, 4–7, and > 7) | Relatively lower 1/3 EAT-Lancet diet score | Nationally representative Swedish dietary survey Riksmaten Vuxna 2010–11 (Riksmaten Adults) | Climate Database developed and maintained by the Research Institutes of Sweden | Climate footprints [carbon dioxide equivalents (CO2e)] | Intake of the food groups included in the cluster analysis, age, income, sex, and CO2eq | Adopting the EAT-Lancet diet would be associated with - lower climate impacts (up to 53%) | [141] |
| Bakman 2024 | Brazil | 2017–2018 | Comparative analysis | - | General population/adults and adolescents, aged over 10 years | EAT-Lancet diet | Current diet | the Family Budget Research 2017–2018 | the ReCiPe 2016 Midpoint Hierarchist method | Climate change, land use, soil acidification, water eutrophication, and water consumption | - | Adopting the EAT-Lancet diet was associated with - lower climate changes (–50%);  - lower land use (–50%);  - higher soil acidification (49%);  - higher water eutrophication (50%);  - higher water consumption (38%) | [142] |
| Bertoldo 2024 | USA | 2022 | Comparative analysis | 19 universities dining programs | - | EAT-Lancet diet | Current Procurement Portfolio | Automated Scope 3 Tool for Tracking Emissions from Food (TASTE Food);  University dining programs in an Excel spreadsheet | World Resources Institute Cool Food Calculator | Greenhouse gas emissions | - | Higher adherence to the EAT-Lancet diet was associated with - lower greenhouse gas emissions (46.1%) | [143] |
| Cai 2024 | China | 1997–2015 | Prospective cohort study | 16,029 | General population/adults, aged 18–65 years | Relatively higher EAT-Lancet diet score | Relatively lower EAT-Lancet diet score | 3-day 24-hour recall | Chinese Food Life Cycle Assessment Database  (CFLCAD) | Greenhouse gas emissions, total water use, and land use | Age, sex, BMI, dietary energy, physical activity, household income, educational level, residence location, dietary knowledge, smoking habits, and alcohol  consumption | Higher adherence to the EAT-Lancet diet was associated with - lower greenhouse gas emissions (2.2%, each SD increase in the index);  - lower land use (2.3%, each SD increase in the index)  not associated with  - total water use | [25] |
| Caldeira 2024 | Brazil | 2017–2018 | Modelling study | - | 57,920 households | EAT-Lancet diet | a. Current diet b. Brazilian Dietary Guidelines | Brazilian Household Budget Survey 2017/18 , and the Footprints of Foods and Culinary Preparations Consumed in Brazil database | Life cycle assessment database | Carbon footprint and water footprint | - | Adopting the EAT-Lancet diet would be associated with - lower carbon footprint (–64% compared to current diet–21% compared to Brazilian Dietary Guidelines)  - lower water footprint (–46% compared to current diet –6% compared to Brazilian Dietary Guidelines) | [144] |
| Conti 2024 | Italy | 2009 | Cross-sectional study | - | 40 Italian nursing homes | The highest EAT-Lancet score (14, 16, 18, 20, 22, 24, 26, and 28) | The lowest EAT-Lancet score | An internal kitchen operated on two 28-day cyclical menus (summer & winter) | Life cycle assessment database | Greenhouse gas emissions | - | Higher adherence to the EAT-Lancet score was  - associated with lower greenhouse gas emissions (approximately 18% in summer, 13% in winter) | [145] |
| DeCesaro 2024 | Global | 2020–2070 | Modeling study | - | General population of all ages | EAT-Lancet diet | Current diet | Current diets from Our World in  Data (OWID) | Standardized cumulative environmental pressure index | Greenhouse gas emissions, nutrient pollution, land/sea disturbance, water use | - | Higher adherence to the EAT-Lancet diet was associated with  - lower greenhouse gas emissions, nutrient pollution, land/sea disturbance, and water use globally  - higher environmental production pressures in low-income countries due to increased food needs | [146] |
| Lengle 2024 | Norway | 2010–2011 | Comparative analysis | 1,787 | General population/adults, females and males aged 18–70 years | EAT-Lancet diet | National food based dietary guidelines | 24-h dietary recalls Norkost 3 survey (2010–2011). | Life cycle assessment database | Water use and land use | - | Higher adherence to the EAT-Lancet diet was associated with - lower water use (32%); - lower land use (61%) | [147] |
| Eberle 2024 | Germany | 2017–2019 | Modelling study | - | General population of all ages | a. EAT-Lancet diet (flexitarian diet scenario)  b. EAT-Lancet diet (vegetarian diet scenario)  c. EAT-Lancet diet (vegan diet scenario) | Current diet | FAO | Life cycle assessment database | Climate change, land use, terrestrial biodiversity impacts, water consumption, water scarcity footprint | - | Higher adherence to the EAT-Lancet would be  - associated with lower water consumption and water scarcity footprint;  - associated with higher climate change, land use, and terrestrial biodiversity impacts | [148] |
| Kesse-Guyot 2024 | France | 2014 | Modelling study | 29,413 | General population/adults, females and males aged 53.5 years (mean) | Relatively higher 1/4 EAT-Lancet score (range from 0–140) | Relatively lower 1/4 EAT-Lancet score | Self-administered validated semi-quantitative FFQ | Dialecte database | Greenhouse gas emissions and land occupation [adjusted mean value (95% CI)] | - | Higher adherence to the EAT-Lancet diet would be associated with  - lower greenhouse gas emissions;  - lower land occupation | [149] |
| Li 2024 | Global | 2019 | Modelling study | - | Household | EAT-Lancet diet | Current diet | FAOSTAT, World Bank Global Consumption Database, and consumer expenditure surveys from high-income countries | Physical trade flow approach (proposed by Kastner et al.) | Greenhouse gas emissions | - | Higher adherence to the EAT-Lancet diet would be associated with  - lower greenhouse gas emissions (17%) | [150] |
| Li 2024 | China | Projection to 2030 | Modelling study | - | General populations, urban and rural residents | EAT-Lancet diet | Current diet (urban vs rural) | Food expenditure and consumption from the China Household Survey, Rural Statistical Yearbook, and Statistical Yearbook; dietary cost inferred from market prices at the provincial level | IPCC emission-factor method | Greenhouse gas emissions | - | Higher adherence to the EAT-Lancet diet was associated with  - lower greenhouse gas emissions (30.24%) | [151] |
| Liu 2024 | China | 1990–2020 | Modelling study | - | Household | EAT-Lancet diet | a. Chinese Dietary Guidelines b. Mediterranean diet c. WHO recommendations diet d. DASH diet | Urban area: the urban household surveys and the integrated household income and expenditure survey in China (https://data.stats. gov.cn/index.htm) Rural area: the China Statistical Yearbook and the China Rural Statistical Yearbook (http://www.stats.gov.cn/sj/ndsj/) | Life cycle assessment database | Water footprint, land footprint, and carbon footprint | - | Adopting the EAT-Lancet diet would be associated with  Urban area - lower carbon footprint (compared to all the compared diets);  - lower land footprint (compared to all the compared diets);  - lower water footprint (compared to all the compared diets)   Rural area - lower carbon footprint (compared to all the compared diets);  - lower land footprint (compared to all the compared diets);  - lower water footprint (compared to all the compared diets) | [152] |
| Payró 2024 | The Netherlands | 2012–2016 | Cross-sectional study | 4,313 | General population/adults and children, aged 1-79 years | EAT-Lancet diet | Current diet | 24-h dietary recalls (two times) | the Dutch National Institute for Public Health and the Environment LCI database, Agri-footprint 5.0, and the GLOBIO biodiversity model | Greenhouse gas footprint, land footprint, eutrophication footprint, blue water footprint, and biodiversity footprint | - | Adopting the EAT-Lancet diet was associated with - lower greenhouse gas footprint (–20.5%);  - lower land footprint (–19.9%);  - lower eutrophication footprint (–6.5%);  - lower biodiversity footprint (–23.9%);  - higher blue water footprint (7.6%) | [153] |
| Rulli 2024 | Global | - | Modeling study | - | General population of all ages | EAT-Lancet diet | Current diet | FAO Food Balance Sheets | Agro-hydrological model | Blue water use, rainfed and irrigated area, unsustainable irrigation, cultivated land | Country-level dietary preferences and religious constraints, production system | Higher adherence to the EAT-Lancet diet was associated with  - lower blue water consumption (–74% to –82%)  - lower irrigated land area (–63% to –68%)  - lower unsustainable irrigation (–40%)  - lower cultivated land (–37% to –40%) | [154] |
| Unar-Munguía, 2024 | Mexico | 2023 | Comparative analysis | 1,337 | General population/adults, females and males aged ≥ 19 years | EAT-Lancet diet | a. current diet b. Mexican Healthy and Sustainable Dietary Guidelines (HSDG 2023) | A 24-h dietary recall | Global Livestock Environ- mental Assessment Model (GLEAM tool) developed by FAO | Land use, water footprint, carbon footprint | - | Compared to current diet, higher adherence to the EAT-Lancet diet was associated with  - lower land use (46.3%);  - lower water footprint (11.8%);  - lower carbon footprint (57.4%)  Compared to HSDG 2023, higher adherence to the EAT-Lancet diet was associated with  - lower land use (23.2%);  - lower water footprint (16.7%);  - lower carbon footprint (35.5%) | [155] |
| Ye 2024 | China | 2019 | Modelling study | - | General population of all ages | EAT-Lancet diet | a. Chinese dietary guidelines (CDGs)  b. WHO guidelines | National Bureau of Statistics of China (NBSC) and Food and Agriculture Organization (FAO) statistics | Population attributable fraction (PAF) approach and life-cycle assessment | Greenhouse gas emissions, freshwater consumption, agricultural land occupation, acidifying emissions and eutrophying emissions | - | In five regions: north, central, east, south, and southwest, higher adherence to EAT-Lancet diet would be  associated with  - lower greenhouse gas emissions (Compared to CDGs);  - higher greenhouse gas emissions (Compared to WHO guidelines);  - higher agricultural land occupation (Compared to WHO guidelines);  - lower acidifying emissions (Compared to CDGs and WHO guidelines)  - lower eutrophying emissions (Compared to CDGs);  - higher eutrophying emissions (Compared to WHO guidelines) | [85] |
| Carvalho 2025 | Portugal | 2015–2016 | Cross-sectional study | 3,852 | General population, aged 3 months–84 years | Relatively higher 1/3 EAT-Lancet diet score, T1–T3 [mean 21.9 (95% CI 21.5 to 22.4), mean 35.0 (95% CI 34.7 to 35.2), mean 50.2 (95% CI 49.5 to 50.9)]  T2 VS.T1, or T3 VS. T2, or T3 VS.T1 | Relatively lower 1/3 EAT-Lancet diet score | Two non-consecutive dietary interviews | the SHARP-Indicators Database (SHARP-ID) and the FoodEx2 classifcation  system | Greenhouse gas emissions and land use (adjusted OR) | Age and sex group | Higher adherence to the EAT-Lancet diet would be associated with  - lower greenhouse gas emissions (OR T3 VS. T1: 1.31, 95% CI: 1.26 to 1.37);  - lower land use (OR T3 VS. T1: 1.25, 95% CI: 1.21 to 1.29) | [87] |
| Watanabe 2025 | Japan | 2011–2012 | Cross-sectional study | 8,043 | General population/adults, females and males aged ≥ 65 years | Relatively higher 1/4 EAT-Lancet diet score, Q1–Q4 (≤ 25, 26–27, 28–30, and ≥ 31) | Relatively lower 1/4 EAT-Lancet diet score | A 47-item semi-quantitative FFQ | the 2012 National Survey of Family Income and Expenditure in Japan | Greenhouse gas emissions | Age, sex, population density, body mass index, family structure, economic status, educational attainment, smoking status, alcohol consumption status, physical activity, sitting time, sleep time, denture use, medication use, number of chronic diseases, frailty status, energy intake, green tea consumption, and coffee consumption | Higher adherence to the EAT-Lancet diet would be generally associated with greenhouse gas emissions  The PHD score at which the mean diet-related GHGE plateaued was approximately 29–31 points | [70] |
| Çelik 2025 | Turkey | 2004–2024 | Cross-sectional study | - | - | EAT-Lancet Diet | Current diet (Regional traditional menus) | Cookbook recipes; Google Trends selection | life cycle assessment (LCA); Su-EATABLE LIFE (SEL) database | Carbon footprint, and water footprint | - | Higher adherence to the EAT-Lancet diet was associated with  - lower carbon footprint  - lower water footprint | [156] |
| Chiriacò 2025 | Global | Projection to 2050 | Modelling study | - | - | EAT-Lancet diet | Current diet | FAOSTAT consumption;  EAT-­ Lancet global benchmark diet suggestions; SSP2 (IPCC 2021) | Global Forest Resource Assessment of the Food and Agriculture Organization of the United Nations (FAO-­ FRA 2020); IPCC 2006, 2019b | Greenhouse gas emissions and land use | - | Higher adherence to the EAT-Lancet diet was associated with:  - lower greenhouse gas emissions overall from global diet (−49%)  - higher greenhouse gas emissions from vegetable oil production: +87% from land use change (1163–1210 Mt CO₂/year)  - higher land use change pressure, with 115–120 Mha of forests potentially deforested | [157] |
| Conrad 2025 | United States | 2011–2018 | Cross-sectional study | 18,522 | General population/adults, aged ≥20 years | Relatively higher 1/4 EAT-Lancet diet score Q1–Q4 (4–9, 10, 11, and 12–14) | Relatively lower 1/4 EAT-Lancet diet score | NHANES,  2011–2018; 24‑h recall | The database of Food Impacts on the Environment for Linking to  Diets (dataFIELD) | Greenhouse gas emissions, cumulative energy demand, water scarcity footprint, and land use, | - | Higher adherence to the EAT-Lancet diet (PHDI score) was associated with  - lower greenhouse gas emissions (β = −0.250 to −0.908 kg CO2e per 1 unit increase)  - lower energy demand (β = 2.699 to 0.202 MJ)  - lower land use (β = 0.002 to < 0.001 ha)  - higher water scarcity footprint (343–649 L equivalents) | [158] |
| Crosnier 2025 | Switzerland | 2050 fork-to-farm scenarios; LCA | Modelling study | 10,440,600 | General population of all ages | EAT‑Lancet diet | Swiss Food Pyramid under both conventional & organic production | FAO Food Balance Sheets (FBS) (FAOSTAT, 2023b); FAO’s Crops and Livestock Products dataset (FAOSTAT, 2023a) | Swiss commodity model , and Life Cycle Assessment (LCA) approach | Climate change, qcidification, eutrophication, freshwater, eutrophication, marine, eutrophication, terrestrial, ecotoxicity, freshwater, land use, water use, resource use, fossils | - | Higher adherence to the EAT-Lancet diet was associated with  - lower climate change, ecotoxicity, freshwater  - higher eutrophication, freshwater, water use, resource use, fossils | [159] |
| de Lange 2025 | Bangladesh | 2022-2050 | Modelling study | - | - | EAT-Lancet diet | Current diet | Modular Applied GeNeral Equilibrium Tool  (MAGNET) model | Global multi-regional input–output (MRIO) databases | Freshwater use, nitrogen application, land use, phosphorus application, greenhouse gas emissions | - | Higher adherence to the EAT-Lancet diet was associated with  - lower freshwater use, nitrogen application, greenhouse gas emissions - higher land use, phosphorus application, | [160] |
| Deng 2025 | Global | 2020-2070 | Modeling study | - | General population of all ages | EAT-Lancet diet | Current diet | MAgPIE 4.6.3 model( included FAOSTAT data) | MAgPIE 4.6.3 model | Water use | - | Higher adherence to the EAT-Lancet diet was associated with  - lower water use globally by 708.65 km³/year by 2070  - higher short-term water use in 43 countries (69.8% in Africa and Asia) during early transition | [161] |
| Herforth 2025 | Global | 2021 | Modeling study | - | 30-year-old woman (national averages) | EAT-Lancet diet | Healthy Diet Basket (HDB) | 2021 International Comparison Program (ICP) | A multilevel carbon and water footprint dataset of food commodities | Greenhouse gas emissions, water use | - | Higher adherence to the EAT-Lancet diet was associated with  - lower greenhouse gas emissions (1.45 kg CO₂e/day)  - lower water use (2.34 metric tons/day) | [162] |
| Michailidis 2025 | Denmark | 2030–2100 | Modelling study | - | - | EAT-Lancet diet | Current diet (Business-as-usual diet) | MAGNET general equilibrium model | DayCent process-based  biogeochemical model | Soil CO₂ emissions, N₂O emissions, net greenhouse gas balance | - | Higher adherence to the EAT-Lancet diet was associated with  - lower soil CO₂ emissions (480 Gg CO₂e/year)  - higher soil N₂O emissions (2.1%, 50 Gg CO₂e/year)  - higher net soil greenhouse gas, partially offsetting livestock reductions | [163] |
| Tuyishimire 2025 | Africa) | Recent GENuS dataset, post-2011 | Modelling study | - | General population of all ages | EAT-Lancet diet | Current diet | Global Expanded Nutrient Supply (GENuS) database | IMPACT model (Springmann et al., 2018) | Greenhouse gas emissions, blue water footprint, cropland use | Regional dietary deviations and food supply characteristics | Higher adherence to the EAT-Lancet diet was associated with  - lower Greenhouse gas emissions (up to –65.3% for red meat)  - lower cropland use (2.4–3.5 m²/capita/day)  - lower blue water footprint (up to 495.6 L/capita/day) | [164] |
| Yang 2025 | China | 1952–2023 | Modelling study | - | General population of all ages (Chinese) | EAT-Lancet diet | Current diet | China Agricultural Statistics 1949–2019, China Fishery Statistics  Yearbook (2006–2024), China Fishery Statistics Bulletin 2023, China Rural Statistical Yearbook (1988–2023) ,  China Marine Economic Statistical Bulletin (2018–2023) | lifecycle analyses (LCA) perspective | Carbon footprint, water footprint | Regional consumption patterns and dietary recommendations | Higher adherence to the EAT-Lancet diet was associated with  - lower carbon footprint (reduction depending on province)  - lower water footprint (1.22 m³/kg reduction potential through diet alignment) | [165] |

Abbreviations: BMI, Body mass index; CI, confidence interval; DASH, Dietary Approaches to Stop Hypertension; FAO, Food and Agriculture Organization of the United Nations; FAOSTAT, Food and Agriculture Organization Corporate Statistical Database; FFQ, food frequency questionnaire; HR, hazard ratio; Q, quantile; SD, standard deviation; SE, standard error; T, tertile; WHO, World Health Organization

**Table S5. Summary of articles investigating the nutrient intakes and deficiencies of the EAT-lancet planetary health diet**

| **Study** | **Country** | **Assessment period** | **Study type** | **Cohort size, n** | **Study subject** | **Study diet** | **Compared diet** | **Dietary data sources or assessment method** | **Nutrient calculation method** | **Outcomes** | | **Main findings** | **Reference** |
| --- | --- | --- | --- | --- | --- | --- | --- | --- | --- | --- | --- | --- | --- |
| Lassen 2020 | Denmark | 2011–2013 | Modelling study | 3,189 | General population/adults, females and males aged 15–75 years | EAT-Lancet diet with Danish foods | Nordic nutrition recommendations 2012 | - | Danish National Survey of Diet and Physical Activity 2011–2013 and Nordic nutrition recommendations recommended nutrient density and intake values | Sufficient and deficiency of micronutrients and macronutrients intake [β value (95% CI)] | | Compared with Nordic nutrition recommendations 2012, adherence to EAT-Lancet diet may result in - sufficient: vitamin B_1_, vitamin B_2_, vitamin B_6_, vitamin B_12_, vitamin C, vitamin E, niacin, and folate; - deficiency: vitamin A and vitamin D | [166] |
| Cacau 2021 | Brazil | 2008–2010 | Longitudinal study | 14,779 | General population/adults, females and males, aged 35–74 years | Relatively higher 1/5 EAT-Lancet diet score, Q1–Q5 (range from 0– 150) | Relatively lower 1/5 EAT-Lancet diet score | A 114-item FFQ | United States Department of Agriculture Food Composition Database | Sufficient and deficiency of micronutrients and macronutrients intake | | Higher adherence to EAT-Lancet diet was - positively associated with carbohydrates, vegetable proteins, polyunsaturated fats, fiber, vitamin A, vitamin E, vitamin K, vitamin C, thiamine, folate, iron, phosphorus, potassium, zinc, selenium, magnesium, and copper; - negatively associated with animal protein, total fat, saturated fat, cholesterol, monounsaturated fat, riboflavin, niacin, pyridoxine, vitamin B5 and vitamin B_12_; - the EAT-Lancet diet score was not associated with total energy, total protein, calcium, or sodium | [103] |
| Hanley–Cook 2021 | Congo, Ecuador, Kenya, Sri Lanka, and Vietnam | 2009–2015 | Cross-sectional study | 1,950 | Non-pregnant non-lactating women, aged 15–49 years | Relatively higher 1/4 EAT-Lancet diet score, Q1–Q4 (4–9, 10, 11, and 12–14) | Relatively lower 1/4 EAT-Lancet diet score | A quantitative and comparable single multiple-pass 24-h dietary recall | A probability approach developed by Institute of Medicine (2000) | Probability of micronutrient adequacy [β value (SE)] | | Higher adherence to EAT-Lancet diet was - positively associated with probability of micronutrient adequacy (with minimum intake or adjusted for energy intake); - negatively associated with probability of micronutrient adequacy (without minimum intake) | [167] |
| Kesse–Guyot 2021 | France | 2009–2014 | Cross-sectional study | 29,210 | General population/adults, females and males aged 53.5 (SD 14.0) years | Relatively higher 1/5 EAT-Lancet diet score, Q1–Q5 (≤ 4.35, 4.35–21.46, 21.46–37.67, 37.67–59.74, and > 59.74) | Relatively lower 1/5 EAT-Lancet diet score | A web-based semi-quantitative FFQ | Nutrient intakes, were computed using a published composition table from the NutriNet–Sante cohort study zinc and iron, were computed using a published equation (Access in: https://doi.org/10.3945/jn.112.169904) | Sufficient and deficiency of micronutrients and macronutrients intake | Higher adherence to EAT-Lancet diet was - positively associated with carbohydrates, PUFA, proportion of plant proteins, fiber, vitamin B_9_, vitamin C, vitamin E, calcium, and iron; - negatively associated with lipids, SFA, MUFA, proteins, sodium, vitamin B_12_, heme-iron, and zinc | | [104] |
| Kidd 2021 | New Zealand | 2002 and 2008–2009 | Modelling study | - | 210–237 households | a. Flexitarian diet (modelled from EAT-Lancet diet guideline) b. Vegan diet (modelled from EAT-Lancet diet guideline) | Healthy diet: 2015 New Zealand Eating and Activity Guidelines | Food composition data (FOODfiles) & dietary guidelines | The Concise New Zealand food composition tables, 2018 | Sufficient and deficiency of micronutrients and macronutrients intake | Compared with Healthy diet: 2015 New Zealand Eating and Activity Guidelines, adherence to flexitarian diet (modelled from EAT-Lancet diet guideline) may result in - sufficient: fiber, fat; - deficiency: carbohydrate, sodium  adherence to vegan diet (modelled from EAT-Lancet diet guideline) may result in  - sufficient: fiber, fat; - deficiency: carbohydrate, protein, sodium | | [117] |
| Tucci 2021 | Italy | - | Modelling study | - | - | EAT-Lancet diet [Meet Italian food habits (EAT-IT)] | Italian Dietary Guidelines | EAT-Lancet Commission Reference Diet (adapted by considering the Mediterranean/Italian food habits) | MètaDieta professional 4.1.1 METEDA Srl-Roma, Italy (MetaDieta software ) | Sufficient and deficiency of macronutrients intake | Compared with Italian Dietary Guidelines, adherence to EAT-Lancet diet may result in  - sufficient: lipids, vegetal protein, and fiber, iron, vitamin E, vitamin B_1_, vitamin B_3_, vitamin B_6_, magnesium, phosphorus, and zinc; - deficiency: protein sources (from meat, fish, and dairy), fats, calcium, vitamin A, vitamin B_2_, vitamin B_9_, vitamin B_12_, vitamin C, vitamin D, sodium, chlorine, and potassium | | [168] |
| Berthy 2022 | French | 2009–2021 (ongoing) | Cross-sectional study | 62,382 | General population/adults, females aged 46.6 (SD 25.8) years, males aged 41.7 (SD 24.8) years | Relatively higher 1/5 EAT-Lancet diet score, Q1–Q5 [mean 12.2 (SD 16.3), mean 34.7 (SD 3.7), mean 46.1 (SD 3.1), mean 58.0 (SD 3.9), and mean 81.9 (SD 17.9)] | Relatively lower 1/5 EAT-Lancet diet score | A series of 3 non-consecutive 24-h dietary recalls | Table de composition des aliments, étude NutriNet–Santé (in French). Paris (France): Les éditions INSERM/Economica, 2013 | Sufficient and deficiency of micronutrients and macronutrients intake | Higher adherence to EAT-Lancet diet was - positively associated with carbohydrates, polyunsaturated fats, calcium, iron, fiber, magnesium, manganese, phosphorus, potassium, vitamin A, vitamin B_1_, vitamin B_2_, vitamin B_5_, vitamin B_6_, vitamin B_9_, vitamin C, and vitamin E; - negatively associated with proteins, monounsaturated fats, saturated fats, retinol, selenium, zinc, vitamin B_12_ and vitamin D | | [3] |
| Hendrie 2022 | Australia | 2011–2013 | Cross-sectional study | 5,920 | General population/adults, females and males aged 19–50 years | EAT-Lancet diet | Australian Dietary Guidelines | 24-h dietary recalls (two times) | The Australian Food, Supplement and Nutrient Database 2011–2013 | Sufficient and deficiency of micronutrients and macronutrients intake | Compared with Australian Dietary Guidelines, adherence to EAT-Lancet diet resulted in  - sufficient: thiamin, iron, selenium, linoleic acid, alpha–linolenic acid, and omega-3; - deficiency: protein, dietary fiber, ribofavin, niacin, vitamin A, vitamin B_12_, vitamin C, vitamin D, folate, calcium, phosphorus, zinc, magnesium, iodine, sodium, and potassium | | [169] |
| Lazarova 2022 | Canada | 2004 and 2015 | Cross-sectional study | 14,026 | General population/adults, females and males aged 45–80 | Relatively higher 1/4 EAT-Lancet diet score Q1–Q4 (4–9, 10, 11, and 12–14) | Relatively lower 1/4 EAT-Lancet diet score | 24-h dietary recalls (two times, Automated Multiple-Pass Method) | Health Canada’s Canadian Nutrient File | Sufficient and deficiency of micronutrients and macronutrients intake | Higher adherence to EAT-Lancet diet was - positively associated with carbohydrates, densities of fiber, vitamin C, vitamin B_9_, folacin, magnesium, and iron; - negatively associated with saturated fat | | [6] |
| Vallejo 2022 | Germany | 1985–2021 (ongoing) | Cross-sectional study | 298 | Young adults and adolescents, females and males aged ≥ 15 years | Relatively higher 1/3 EAT-Lancet diet score, male T1–T3 [median 9 range (7–9), median 10 range (10–10), and median 11 range (11–14)], female T1–T3 [median 9 range (6–10), median 11 range (11–11), and median 12 range (12–15)] | Relatively lower 1/3 EAT-Lancet score | 3-day weighed dietary records  (2–5 sets) | Dortmund Nutritional and Anthropometric Longitudinal Designed | Sufficient and deficiency of micronutrients and macronutrients intake | Higher adherence to EAT-Lancet diet was - positively associated with plant protein, fiber, magnesium, vitamin E, vitamin K, and total folic acid; - negatively associated with protein (total and animal), cholesterol, arachidonic acid | | [9] |
| Beal 2023 | Global | 2022 | Comparative analysis | - | General population/adults, females and males aged ≥ 25; women aged 15–49 | EAT-Lancet diet | Globally harmonised recommended diet | FAOSTAT | USDA FoodData Central (FDC) and multiple national and regional food composition tables (FCTs) | Sufficient and deficiency of micronutrients | Compared with French Programme National Nutrition Santé guidelines, adherence to EAT-Lancet diet resulted in - sufficient: –; - deficiency: vitamin B_12_, calcium, iron, and zinc | | [170] |
| Berthy 2023 | France | 2009–2015 | Cross-sectional study | 98,465 | General population/adults, females and males aged ≥ 18 | EAT-Lancet diet: Relatively higher 1/5 EAT-Lancet score, Q1–Q5 (≤ 13, 14–16, 17–19, 20–22, and ≥ 23) | French Programme National Nutrition Santé guidelines | 24-h dietary recalls (three non-consecutive records, including weekends and weekdays, collected twice a year) | Published food composition table (in French) | Sufficient and deficiency of micronutrients and macronutrients intake | Compared with French Programme National Nutrition Santé guidelines, adherence to EAT-Lancet diet resulted in - sufficient: bioavailable zinc, vitamin B_12_, vitamin B_9_ and vitamin C; - deficiency: fiber, vitamin B1, iodine, and magnesium  Higher adherence to EAT-Lancet diet was  - positively associated with β-carotene, calcium, copper, total iron, iodine, potassium, magnesium, manganese, phosphorus, vitamin A, vitamin B_9_, and vitamin C; - negatively associated with cholesterol, vitamin B_12_, and heme-iron | | [171] |
| Cacau 2023 | Ten European cities | 2006–2007 | Cross-sectional study | 1,804 | Adolescents, females and males aged 12.5–17.5 | Relatively higher EAT-Lancet diet score | Relatively lower EAT-Lancet diet score | 24-h dietary recalls (two non-consecutive recalls) | HELENA Dietary Assessment Tool | Sufficient and deficiency of micronutrients and macronutrients intake [β value (95% CI)] | Higher adherence to EAT-Lancet diet was - positively associated with β-carotene, vitamin C, vitamin D, vitamin B_9_, and ferritin; - negatively associated with vitamin A, vitamin E, holo–transcobalamin, and n-3 fatty acids | | [172] |
| Hargous 2023 | Chile | 2016 | Cross-sectional study | 958 | Children (pre-schoolers), females and males aged 3–6 | Relatively higher EAT-Lancet diet score (range from 0– 150) | Relatively lower EAT-Lancet diet score | 24-h dietary recalls  (for one day) | United States Department of Agriculture National Nutrient Database and food labels of packaged products available in Chile during 2016 | Sufficient and deficiency of macronutrients intake [difference (95% CI)] | Higher adherence to EAT-Lancet diet was - positively associated with plant-based protein, total carbohydrates, and total fiber; - negatively associated with saturated fats, trans fats, and animal-based protein | | [173] |
| Macit–Çelebi 2023 | Turkey | 2022–2023 | Cross-sectional study | 1,112 | General population/adults, females and males aged 19–64 | Relatively higher 1/4 EAT-Lancet diet score, Q1–Q4 (0–30.56, 30.56–40.61, 40.61–51.99, and 51.99–98.96) | Relatively lower 1/4 EAT-Lancet diet score | 24-h dietary recalls  (for one day) | Nutrition Information System (BeBiS) program (version 9) | Sufficient and deficiency of micronutrients and macronutrients intake [β value (95% CI)] | Higher adherence to EAT-Lancet diet was - positively associated with fiber, vitamin E vitamin B_9_, and potassium; - negatively associated with calcium and vitamin B_6_ | | [174] |
| Wright 2023 | Norway | 2007–2019 | Cross-sectional study | 1,413 | Children, females and males aged 2–5 | EAT-Lancet diet | Scenario diet based on the Norwegian food-based dietary guidelines | A 125-item semi-quantitative FFQ | KBS database version AE–22 | Sufficient and deficiency of micronutrients and macronutrients intake | Compared with scenario diet based on the Norwegian food-based dietary guidelines, adherence to EAT-Lancet diet resulted in - sufficient: total fats, vitamin E, vitamin B_1_, and vitamin B_9_, iron; - deficiency: protein, total carbohydrates, vitamin A, vitamin D, vitamin B_2_, vitamin B_3_, vitamin B_6_, vitamin B_12_, vitamin C, calcium, zinc, selenium and iodine | | [175] |
| Armes 2024 | India | 2022 | Comparative analysis | - | General population of all ages | EAT-Lancet diet | a. Kanhu Thali (Represents the winter season diet) b. Jhano Thali (Represents the diet during the transition from late summer to the monsoon season) | Nutritics Professional Premium software | Nutritics Professional Premium software | Sufficient and deficiency of micronutrients and macronutrients intake | Compared with Kanhu Thal and Jhano Thali, adherence to EAT-Lancet diet resulted in - sufficient: plant-based protein sources, unsaturated fats, vitamin D, vitamin K, calcium, selenium, and iodine | | [176] |
| Caldeira 2024 | Brazil | 2017–2018 | Modelling study | - | 57,920 households | EAT-Lancet diet | Brazilian Dietary Guidelines | Brazilian Household Budget Survey 2017/18 , and the Footprints of Foods and Culinary Preparations Consumed in Brazil database | the WHO, United States Institute of Medicine, and international Dietary Reference Intakes | Sufficient and deficiency of micronutrients and macronutrients intake | Compared with Brazilian Dietary Guidelines, adherence to EAT-Lancet diet would result in - sufficient: protein and sodium; - deficiency: carbohydrates, total fat, saturated fat, and fiber | | [144] |
| Frank 2024 | USA | 2003–2018 | Cross-sectional study | 33,859 | General population/adults, females and males aged ≥ 20 | Relatively higher 1/5 EAT-Lancet diet score, Q1–Q5 (18.5–54.0, 54.5–62.0, 62.5–69.0, 69.5–77.0, 77.5–125.0) | Relatively lower 1/5 EAT-Lancet diet score | 24-h dietary recalls (2 day recalls United States Department of Agriculture Automated Multiple Pass Method) | Food Patterns Equivalent Database | Sufficient and deficiency of micronutrients and macronutrients intake | Higher adherence to EAT-Lancet diet was - positively associated with iron, fiber, potassium; - negatively associated with potassium | | [28] |
| Gaona–Pineda 2024 | Mexico | 2006, 2012, 2016, and 2018 | Cross- sectional study | 16,520 | Adolescents, females (non-pregnant, non-lactating adolescents) and males aged 12–19 | EAT-Lancet diet | a. GBD optimal intake recommendations b. 2015 Mexican Dietary Guidelines | Semi-quantitative FFQ | Food nutritional composition tables | Sufficient and deficiency of macronutrients intake [β value (95% CI)] | Compared with Chinese Dietary Guidelines, Mediterranean diet, WHO recommendations diet, and DASH diet, adherence to EAT-Lancet diet may result in - sufficient: saturated fats; - deficiency: high-fiber cereals, vegetables, and dairy products | | [177] |
| Guzmán-Castellanos 2024 | Spain | 1999 | Cross-sectional study | 18,656 | General population/adults, aged ≥ 20 years | Relatively higher 1/4 EAT-Lancet diet score, Q1–Q4 (7–18, 19–21, 22–23, and 24–37) | Relatively lower 1/4 EAT-Lancet diet score | A self-administered 136-item FFQ | Spanish food composition tables | Sufficient and deficiency of macronutrients | Higher adherence to EAT-Lancet diet was - positively associated with carbohydrates, fiber, fats, n-6 fatty acids, and MUFAs; - negatively associated with proteins, SFAs, and cholesterol | | [30] |
| Liu 2024 | China | 1990–2020 | Modelling study | - | Household | EAT-Lancet diet | a. Chinese Dietary Guidelines b. Mediterranean diet c. WHO recommendations diet d. DASH diet | Urban area: the urban household surveys and the integrated household income and expenditure survey in China (https://data.stats. gov.cn/index.htm) Rural area: the China Statistical Yearbook and the China Rural Statistical Yearbook (http://www.stats.gov.cn/sj/ndsj/) | Based on the recommended intake level of eighteen beneficial nutrients, including protein, N-3 PUFA, fiber, vitamin A, vitamin B1, vitamin B2, niacin, vitamin C, Vitamin E, Calcium, Phosphorus, Potassium, Magnesium, Iron, Zinc, Selenium, Copper, Manganese.  Higher values of mean adequacy ratio generally represent better nutritional quality | Mean adequacy ratio | Higher adherence to EAT-Lancet diet was - lower: mean adequacy ratio (compared to Chinese Dietary Guidelines, Mediterranean diet, WHO recommendations diet, and DASH diet) | | [152] |
| Mohammadi 2024 | Iran | 2008–2010 | Case-control study | 213 | Recruited from three general hospitals and 19 CRC surgery departments, females and males aged 40–69 years | Relatively higher 1/3 EAT-Lancet diet score, T1–T3 (≤ 53, 54–62, and ≥ 63) | Relatively lower 1/3 EAT-Lancet diet score | A semi-quantitative FFQ | Nutritionist IV | Sufficient and deficiency of macronutrientsand | Higher adherence to EAT-Lancet diet was - positively associated with protein; - negatively associated with none | | [84] |
| Mortas 2024 | Turkey | 2022 | Cross-sectional study | 945 | Young adults, females (582) and males (383) aged 19–30 | EAT-Lancet diet score | Healthy Eating Index–2020 | 24-h dietary recalls | BeBiS program (BeBiS, 7.2 version). | Sufficient and deficiency of macronutrients intake [β value (95% CI)] | Compared with Healthy Eating Index–2020, adherence to EAT-Lancet diet resulted in  - deficiency: saturated fatty acids | | [178] |
| Nicol 2024 | UK | 2015 | Modelling study | - | General population of all ages | EAT-Lancet diet | WHO recommendations diet | McCance and Widdowson’s the Composition of Foods Integrated Dataset 2015 | Iodine concentration data from UK food tables | Sufficient and deficiency of iodine | Compared with WHO recommendations diet, adherence to EAT-Lancet diet resulted in  - deficiency: iodine | | [179] |
| Shojaei 2024 | Iran | 2017–2020 | Cross-sectional study | 6,465 | General population/adults, females and males aged 30–70 years | Relatively higher 1/4 EAT-Lancet diet score, Q1–Q4 [mean 41.5 (SD 2.9), mean 48.6 (SD 1.6), mean 54.5 (SD 1.8), and mean 64.2 (SD 5.3)] | Relatively lower 1/4 EAT-Lancet score | A 130-item FFQ | Nutritionist IV | Sufficient and deficiency of macronutrients and micronutrients | Higher adherence to EAT-Lancet diet was - positively associated with protein, vegetable protein, riboflavin, carbohydrate, polyunsaturated fat, fiber, thiamine, folate, iron, phosphorus, potassium, zinc, selenium, magnesium, copper, vitamin B_5_, vitamin E, vitamin K, and vitamin C;  - negatively associated with animal protein, total fat, cholesterol, saturated fat, niacin, calcium, vitamin B_12_, and vitamin A | | [44] |
| Uriza-Pinzón 2024 | Germany | 2012–2016 | Modelling study | - | General population of all ages | EAT-Lancet diet | Dutch Dietary Guidelines | Portie Online fromDutch Nutritional Center publications (Access in https://portie-online. rivm.nl/ ) | Dutch National Food Consumption Survey (FCS-2016) & Dutch Food Composition Database | Sufficient and deficiency of micronutrients and macronutrients intake | Compared with Dutch Dietary Guidelines, adherence to EAT-Lancet diet may result in  - sufficient: carbohydrates, mono- and disaccharides, sugars, polysaccharides, total protein, animal protein, alpha linolenic acid;  - deficiency: fiber, vegetable protein, total fat, saturated fat, polyunsaturated fat, linoleic acid, eicosapentaenoic acid, docosahexaenoic acid, marine fatty acids, monounsaturated fat, unsaturated fat, cis, trans fat, cholesterol | | [155] |
| Berthy 2025 | France | 2009–2022 | Cross-sectional study | 88,964 | General population/adults, aged ≥ 18 years | Relatively higher 1/5 EAT-Lancet diet score, Q1–Q5 (cut-offs for quintiles of EAT-Lancet diet score were 192.2/25.5/38.3/49.1/62.8/332.2 for females and 148.7/22.6/34.9/44.9/57.7/ 332.2 for males) | Relatively lower 1/5 EAT-Lancet diet score | Three nonconsecutive web-based 24-hour dietary records | - | Sufficient and deficiency of micronutrients and macronutrients intake | Higher adherence to EAT-Lancet diet was - positively associated with plant proteins, carbohydrates, calcium, iron, fibers, iodine, magnesium, manganese, phosphorus, potassium, selenium, sodium, vitamin A, vitamin B_1_, vitamin B_2_, vitamin B_3_, vitamin B_5_, vitamin C, and vitamin E;  - negatively associated with proteins, animal proteins, lipids, MUFA, PUFA, saturated fats, retinol, zinc, vitamin B_6_, and vitamin B_12_ | | [55] |
| Carvalho 2025 | Portugal | 2015–2016 | Cross-sectional study | 3,852 | General population, aged 3 months–84 years | Relatively higher EAT-Lancet diet score | Relatively lower EAT-Lancet diet score | Two non-consecutive dietary interviews | the Portuguese Food Composition Table | Sufficient and deficiency of micronutrients and macronutrients intake | Higher adherence to EAT-Lancet diet was - positively associated with fat and SFA; - negatively associated with animal protein, protein, fiber, and PUFA | | [87] |
| Dehnavi 2025 | Iran | 2022–2023 | Cross-sectional study | 398 | Elderly individuals, aged 60–84 years | Relatively higher 1/3 EAT-Lancet diet score, T1–T3 (≤ 53, 53–61 and ≥ 61) | Relatively higher 1/3 EAT-Lancet diet score, T1–T3 (≤ 53, 53–61 and ≥ 61) | A semi-quantitative 168-item FFQ | Nutritionist 4  software (First Databank, Hearst Corp., San Bruno, CA,  USA) | Sufficient and deficiency of micronutrients and macronutrients intake | Higher adherence to EAT-Lancet diet was - positively associated with carbohydrate, total fiber, iron, vitamin E, vitamin C, and vitamin K; - negatively associated with total fat and SFA | | [57] |
| Guzmán-Castellanos 2025 | Spain | Unknown to May 2022 | Longitudinal cohort analysis | 18,259 | University graduates, adults aged ≥20 years | Relatively higher 1/4  EAT-Lancet diet score, Q1–Q4 (7–18, 19-21, 22-23, and 24–39) | Relatively lower 1/4 EAT-Lancet diet score | Validated 554-item semi-quantitative FFQ | Estimated Average Requirement (EAR) cut-point & probabilistic approach | Sufficient and deficiency of macronutrients and micronutrients | Higher adherence to EAT-Lancet diet was  - positive associated with carbohydrates, dietary fiber, magnesium, potassium, zinc, iron, vitamin B₁, vitamin B₆, vitamin C, vitamin A, vitamin D, vitamin E, folic acid  - negative associated with total energy intake, protein, fat, saturated fatty acids, cholesterol, n-6 fatty acids, iodine, selenium, calcium, phosphorus, chromium, vitamin B₂, vitamin B₃, vitamin B₁₂ | | [180] |
| Jovanović 2025 | Croatia | 2023–2024 | Cross-sectional study | 224 | University students, aged 19–27 years | Relatively higher 1/4  EAT-Lancet diet score, Q1–Q4 (20–45, 46–55, 56–65, and 66–100) | Relatively lower 1/4 EAT-Lancet diet score | 98-item semi-quantitative FFQ | Croatian Food Composition Database | Sufficient and deficiency of macronutrients and micronutrients | Higher adherence to EAT-Lancet diet was  - positive associated with energy, vegetable protein, monounsaturated fat, polyunsaturated fat, omega-3 fatty acids, dietary fiber, alcohol, thiamine, riboflavin, folate, vitamin C, vitamin E, potassium, magnesium, iron, zinc, selenium, flavan-3-ols, flavones, flavanones, anthocyanidins  - negative associated with total protein, animal protein, saturated fat, trans fat, omega-6 fatty acids, vitamin B₁₂ | | [109] |
| Kersting 2025 | Germany | - | Modelling study | - | Children, aged 4–6 years | Updated Optimized Mixed Diet (OMD) score (adjusted according to the PHDI score) | Original  Optimized Mixed Diet (OMD) score | OMD 7-day menu | German Food Code and Nutrient DataBase (BLS) (Bundeslebensmittelschlüssel, BLS, Version II.3) | Sufficient and deficiency of macronutrients and micronutrients | Higher adherence to EAT-Lancet diet was  - positive associated with fiber, vitamin A, vitamin E, vitamin B_2_, vitamin B_6_, folate, calcium, phosphorus, magnesium, iron, zinc  - negative associated with carbohydrates, vitamin B_12_, vitamin C, iodine | | [181] |
| Lei 2025 | China | 1997–2015 | Cross-sectional study | 11,402 | General population/adults, females and males aged ≥ 18 years | Relatively higher 1/4 EAT-Lancet diet score, Q1–Q4 (16–39, 40–44, 45–50, and 51–95) | Relatively lower 1/4 EAT-Lancet diet score | Self-reported 24-h dietary recalls | the Chinese Food Composition Tables | Sufficient and deficiency of macronutrients | Higher adherence to EAT-Lancet diet was - positively associated with fat, protein, potassium, and fiber;  - negatively associated with carbohydrate | | [182] |
| Mansouri 2025 | Iran | 2022 | Cross-sectional study | 109 | Recruited from Motahari and Imam Reza  clinics, aged ≥ 18 years | Relatively higher 1/2 EAT-Lancet diet score (> 74 and ≤ 74) | Relatively lower 1/2 EAT-Lancet diet score | A 168-item FFQ | Mindray device (manufactured  in Japan) with Pars Azmoon kits and FFQ | Sufficient and deficiency of macronutrientsand micronutrients | Higher adherence to EAT-Lancet diet was - positively associated with PUFA;  - negatively associated with none | | [95] |
| Miranda 2025 | France | 2014–2015 | Cross-sectional study | 1,723 | Adults, aged ≥18 years | Relatively higher 1/5 EAT-Lancet diet score, Q1–Q5 (range from 0 to 150) | Relatively lower 1/5 EAT-Lancet diet score | 3 nonconsecutive dietary  Recalls (2 weekdays and 1 weekend) | ANSES-CIQUAL French food composition table version 2016 | Sufficient and deficiency of macronutrients and micronutrients | Higher adherence to the EAT-Lancet diet was  - positive associated with LA, ALA, DHA, EPA+DHA, fibre, vitamin A, vitamin B-6, folate, vitamin B_12_, vitamin C, vitamin D, vitamin E, iodine, selenium, copper, calcium, iron, carbohydrates, total fat, saturated fatty acids  - negative associated with niacin, zinc, sugars, sodium | | [183] |
| Tabatabaei 2025 | Iran | 2018– 2019 | Cross-sectional study | 1,970 | General population/adults, females and males aged ≥ 18 years | Relatively higher 1/3 EAT-Lancet diet score, T1–T3 (–160.44 to –9.81, –9.77 to 10.06, 10.15 to 105.63) | Relatively lower 1/3 EAT-Lancet diet score | A 110-item semi-quantitative FFQ | Nutritionist IV | Sufficient and deficiency of macronutrients | Higher adherence to EAT-Lancet diet was - positively associated with protein, fiber, and PUFA;  - negatively associated with saturated fat, SFA, MUFA, and fat | | [67] |
| Vargas-Quesada 2025 | 8 Latin American countries | 2014–2015 | Cross-sectional study | 6,835 | Urban populations (adolescents and adults) | Relatively higher 1/5 EAT-Lancet diet score, Q1–Q5 (range from 0 to 150) | Relatively lower 1/5 EAT-Lancet diet score | two 24-hour recalls | (NDS-R) soft- ware version 2013 | Sufficient and deficiency of macronutrients and micronutrients | Higher adherence to the EAT-Lancet diet was  - positive associated with carbohydrates, dietary fiber, polyunsaturated fat, vitamin B₁, vitamin B₆, folate, vitamin C, vitamin A, iron, magnesium, zinc  - negative associated with total energy intake, protein, total fat, saturated fat, trans fat, cholesterol, vitamin B₂, vitamin B₃, vitamin B₁₂, vitamin D, calcium | | [184] |
| Wu 2025 | China | 1997–2015 | Cross-sectional study | 14,652 (new-onset cardiometabolic diseases); 15,318 (all-cause mortality) | General population/adults, females and males aged ≥ 18 years | Relatively higher 1/4 EAT-Lancet diet score, Q1–Q4 (PHDI and WISH) | Relatively lower 1/4 EAT-Lancet diet score | 3-day consecutive 24-h dietary recalls, and consumption of cooking oils and condiments was determined at the household level using a food weighing method | the China Food Composition Tables | Sufficient and deficiency of macronutrients | Higher adherence to EAT-Lancet diet (both PHDI and WISH) was - positively associated with carbohydrate, protein, fat (WISH), and dietary fiber;  - negatively associated with saturated fat (PHDI) | | [71] |

Abbreviations: CI, confidence interval; DASH, Dietary Approaches to Stop Hypertension; FFQ, food frequency questionnaire; GBD, Global Burden of Disease; MUFA, monounsaturated fatty acid; PUFA, polyunsaturated fatty acid; Q, quantile; SD, standard deviation; SE, standard error; SFA, saturated fatty acid; T, tertile; WHO, World Health Organization

**Table S6. Summary of articles investigating the affordability of the EAT-lancet planetary health diet**

| **Study** | **Country** | **Assessment period** | **Study type** | **Study subject** | **Study diet** | **Compared diet** | **Dietary data sources or assessment method** | **Calculation methods of economic indicators** | **Outcomes** | **Main findings** | **Reference** |
| --- | --- | --- | --- | --- | --- | --- | --- | --- | --- | --- | --- |
| Hirvonen 2020 | Global | 2011 | Cross-sectional study | 159 countries | EAT-Lancet diet | - | The US Department of Agriculture National Nutrient Database | Food cost data: World Bank's International Comparison Program Income data: World Bank's PovcalNet system | Cost of the EAT-Lancet diet (dollars)  Cost of the EAT-Lancet diet relative to mean daily per capita household (dollars)  The number of people for whom the daily cost of an EAT-Lancet diet is not currently affordable (dollars) | Transition to EAT-Lancet diet may result in - a larger median daily cost of food consumptions in high-income countries than in low-income countries, and the cost was highest in the Latin America and Caribbean region and lowest in sub-Saharan Africa - the aﬀordability, as a proportion of mean daily household income per capita, was 6.1% in high-income countries, 27.5% in upper-middle-income countries, 52.4% in lower-middle-income countries, and 89.1% in low-income countries  - higher cost of food consumptions that exceeds total income for at least 1.58 billion people, out of which 80% are in middle-income countries. The prevalence of individuals with total household income per person below the estimated least-cost of the EAT-Lancet reference diet is highest in sub-Saharan Africa (57.2%) followed by south Asia (38.4%) | [185] |
| Batis 2021 | Mexico | 2011–2018 | Cross-sectional study | 10,086 general population of all ages | EAT-Lancet diet | a. Mexican Dietary Guidelines b. Locals’ current diet | Mexican National Health and Nutrition Survey 2012 survey 24-h diet recall for current diet | Prices of food items: National Institute for Geography and Statistics | Average cost of diets | Transition to EAT-Lancet diet may result in - a 21% lower average cost than that of the Mexican Dietary Guidelines baskets, and 40% lower than that of the current intake baskets (29% lower if the comparison was isocaloric). Findings were similar over time (2011–2018) and by geographic region | [186] |
| Gupta 2021 | India | 2018–2019 | Cross-sectional study | 119,000 households | EAT-Lancet diet | Current diet | Household survey in the TCI’s TARINA program | The primary data: TCI’s TARINA program Prices of food items: market survey The secondary data: Consumer Pyramids Household Survey | Average cost of the EAT-Lancet diet (dollars)  Average or minimum cost of the EAT-Lancet diet relative to actual diet (dollars) | Transition to EAT-Lancet diet may result in - a higher cost of meat, fish, poultry, dairy foods, and fruits (nearly USD $1.00 per person per day of each food to meet the EAT-Lancet diet guideline) in rural India | [187] |
| Kidd 2021 | New Zealand | 2002 and 2008–2009 | Modelling study | 210–237 households | EAT-Lancet diet | a. Current diet b. Healthy diet | Current diet: Survey data from the most recent NZ National Nutrition Survey (2008/2009)  Healthy diet: 2015 New Zealand Eating and Activity Guidelines  EAT diet: Modelled from the EAT-Lancet Commission’s guidelines | Prices of food items: Each item collected from the three largest supermarkets (Pak‘nSave, Countdown and New World) in 2019 spring | Cost of diets (NZ$) | Transition to EAT-Lancet diet may result in - a generally higher cost to households [the current diet scenario had the lowest cost (NZ $584 per fortnight), followed by healthy (NZ $637), EAT-Lancet diet (NZ $728)] | [117] |
| Philippidis 2021 | Global | 2050 | Modelling study | 57 tradable sectors and 140 regions | EAT-Lancet diet | Business-as-usual scenario diet | Global trade analysis project database | Global trade analysis project database | Average per capita food expenditure in 2050: Baseline vs. EAT-Lancet diet (€ per year) | Transition to EAT-Lancet diet may result in - a moderately higher (2.6%) average cost of food consumptions worldwide (to €1044 per capita/year) in 2050. At the regional level, the switch to the healthy diet results in food expenditure savings in Russia, North America, the EU, China and Sub-Saharan Africa. Elsewhere, the diet switch increases food expenditures in India | [118] |
| Gatto 2023 | Global | 2030 | Modelling study | 8 different regions of the world | EAT-Lancet diet | Business-as-usual scenario diet | The FAO global flows of food loss and waste database (FAO, 2019) combined with literature review | - | Prices of an EAT-Lancet diet  Affordability of an EAT-Lancet diet | Transition to EAT-Lancet diet may result in - lower prices of food consumptions in all regions (except sub-Saharan Africa) - an improvement of affordability of food consumptions for all non- agricultural low-skilled workers except those in sub-Saharan Africa and for agricultural low-skilled workers in high-income regions and Southeast Asia - a reduction in affordability of food consumptions for agricultural low-skilled workers in all but high-income regions and Southeast Asia | [133] |
| Headey 2023 | Four East African countries: Ethiopia, Kenya, Tanzania, and Uganda | 2014–2017 | Cross-sectional study | 41,449 households | EAT-Lancet diet | Current diet | The Ethiopia Socioeconomic Survey 2015–16  The Kenya Integrated Household Budget Survey 2015–16  The Tanzania National Panel Survey 2014–15  The Uganda National Household Survey 2016–17  7-day food consumption recall | Total food expenditures and food group expenditures: the reported expenditures for purchased items and the estimated value of own–produced and gifted foods | The percentage that can afford the EAT-Lancet reference diet | Transition to EAT-Lancet diet may result in  - majority of households in East Africa cannot afford the EAT-Lancet diet;  - in rural areas, median food expenditure is typically half the cost of the cheapest reference diet;  - high percentages of the population in four East African countries are unable to afford the EAT-Lancet diet:  ·96% of rural and 91% of urban Ethiopians；  · 88% of rural and 79% of urban Kenyans；  · 90% of rural and 80% of urban Tanzanians；  ·93% of rural and 87% of urban Ugandans；  - small household food budgets are a significant barrier to transitioning to the EAT-Lancet diet in East Africa | [188] |
| Quarpong 2023 | Kenya | 2014 | Cross-sectional study | 242 lactating mothers | EAT-Lancet diet | Current diet | Single multiple pass method 24‐h dietary intake | Prices of food items: markets within the mothers' locality | Cost of mothers' diets | Transition to EAT-Lancet diet may result in higher food costs compared to mothers' current diets  - Current average cost of mothers' diets is 184.6 Kenya shilling (USD $1.6) person/day  - Hypothetical diets within recommended ranges average 357.5 Kenya shilling (USD $3.0) person/day | [17] |
| Aburto 2024 | Mexico | 2012 and 2016 | Cross-sectional study | 14,242 general population of all ages | EAT-Lancet diet | - | 24-h dietary recalls (two times) | the National System of Statistical and Geographical Information | Cost comparison of EAT-Lancet diet index (high diet cost vs. low diet cost) | Transition to EAT-Lancet diet may result in  - a 1/2 relatively lower diet cost had a higher EAT-Lancet diet index (20.4, 95% CI: 20.2-20.6) than 1/2 relatively higher diet cost (19.3, 95%CI: 19.1-19.6) | [189] |
| Cai 2024 | China | 1997–2015 | Prospective cohort study | General population/adults, aged 18–65 years | Relatively higher EAT-Lancet diet score | Relatively lower EAT-Lancet diet score | 3-day 24-hour recall |  | 3-day 24-hour recall | Transition to EAT-Lancet diet may result in - an increase in diet costs of 3·3% (each SD increase in the index) | [25] |
| Caldeira 2024 | Brazil | 2017-2018 | Modelling study | 57,920 households | EAT-Lancet diet | a. Current diet b. Brazilian Dietary Guidelines | Brazilian Household Budget Survey 2017/18, and the Footprints of Foods and Culinary Preparations Consumed in Brazil database | Prices of food items: The unit price (R$/g or R$/ml) was obtained by dividing the total expense value by the quantity purchased [in weight or volume (g or ml)] | Cost comparison of study and comparison diets | Transition to EAT-Lancet diet may result in  - a higher cost of food consumption, Brazilian Dietary Guidelines (BRL $4.9 (95%CI: 4.8 to 4.9) ≈ USD$1.5), current diet (BRL $5.6 (95% CI: 5.6 to 5.7) ≈ USD $1.8), and the EAT-Lancet diet (BRL $6.1 (95%CI: 6.0 to 6.1) ≈ USD$1.9) | [144] |
| Chen 2024 | China | 2021 | Cross-sectional study | 3,150 general population/ aged ≥ 18 | EAT-Lancet diet | a. current diet b. Chinese Food Guide Pagoda diet  c. Flexitarian diet  d. Customized diet taste, | Collected by a professional survey company in China (https://www.lediaocha.com/) | the Consumer Price Index (CPI) data published in the China Statistical Yearbook (2017-2021) to adjust the minimum cost of diets following FAO | Cost comparison of study and comparison diets that consumers were willing to pay | Transition to EAT-Lancet diet may result in  - a generally lower cost on diets (approximately 45 RMB, 47 RMB, 39 RMB, 47 RMB and 16 RMB per day for the average diet, Chinese Food Guide Pagoda diet, EAT-Lancet diet, Flexitarian diet and customized diet taste, respectively) | [190] |
| Cohen 2024 | USA | 2023 | Cross-sectional study | 23 households | EAT-Lancet diet | The 2021 Thrifty Food Plan developed by the United States Department of Agriculture | Purchase receipts/record | Prices of food items: saving receipts for each family and aligning purchase amounts with costs for items purchase bulk from January 27, 2023, to July 20, 2023 | Cost comparison of study and comparison diets | Transition to EAT-Lancet diet may result in - a lower Cost of food consumptions (USD $100.80 per week) than that of 2021 Thrifty Food Plan [USD $196.83 per week)] | [191] |
| Headey 2024 | Global | 2017 | Modelling study | 137 countries | EAT-Lancet diet | - | - | the International Comparison Program retail food price dataset and purchasing power conversion factors | Share of the population in 137 countries that cannot afford the EAT-Lancet diet | Transition to EAT-Lancet diet may result in  -2.13 to 3.02 billion people globally being unable to afford the cost of the diet | [192] |
| Li 2024 | China | 1998–2021 (observed) + scenarios to 2030 | Modeling study | General populations, urban and rural residents | EAT-Lancet diet | Chinese Dietary Guidelines (CDG) | Food expenditure and consumption from the China Household Survey, Rural Statistical Yearbook, and Statistical Yearbook; dietary cost inferred from market prices at the provincial level | Affordability evaluated via food expenditure share (FAE) and food affordability index (FAI); future affordability predicted using grey prediction model (GM(1)) and Random Forest, under varying price and income scenarios | Affordability of an EAT-Lancet diet | Transition to EAT-Lancet diet may result in:  - From 1998 to 2021, unaffordability rate dropped from 71.32% to 2.03% (≈2.9 million people)  - In 2021, EAT-Lancet diet more affordable than CDG (CDG unaffordability = 30.82%)  - 13% income increase would make EAT-Lancet diet affordable for entire population, even under highest price scenario | [151] |
| Rulli 2024 | Global | 2015–2019 | Modeling study | General populations | EAT-Lancet diet | Current national diets | FAO Food Balance Sheets | Linear optimization model; Agro-hydrological simulation | Prices of an EAT-Lancet diet; Affordability of an EAT-Lancet diet | Transition to EAT-Lancet diet may result in:  - Global median cost rose from Int$3.15 to Int$3.27/day (+4.5%).  - Regional range: Int$1.03–7.37/day across countries.  - Sub-Saharan Africa: diet cost increased 40.5% (Int$2.00 → 2.81/day), highlighting affordability concerns | [154] |
| Unar-Munguía, 2024 | Mexico | 2023 | Comparative analysis | 1,337 general population/adults, females and males aged ≥ 19 years | EAT-Lancet diet | a. current diet (Mexican National Health and Nutrition Survey 2016)  2023  b. Mexican Healthy and Sustainable Dietary Guidelines (HSDG 2023) | A 24-h dietary recall | National Institute of Statistics and Geography (National Institute for Geography and Statistics, acronym of its name in Spanish) in MXN for the year 2016 | Cost comparison of study and comparison diets | Compared to current diet, transition to EAT-Lancet diet may result in  - a 13.8 per-capita cost (2016 MXN per day)  Compared to HSDG 2023 diet, transition to EAT-Lancet diet may result in  - a 3.6 per-capita cost (2016 MXN per day) | [155] |
| Ye 2024 | China | 2019 | Modelling study | General population of all ages | EAT-Lancet diet | a. Chinese dietary guidelines (CDGs)  b. WHO guidelines | National Bureau of Statistics of China (NBSC) and Food and Agriculture Organization (FAO) statistics | National Bureau of Statistics of China (NBSC) | Cost comparison of study and comparison diets | Compared to CDGs, transition to EAT-Lancet diet in 5 regions (north region, central region, east region, south region, and southwest region) may result in  - a lower cost of recommended intake (per capita daily income)  Compared to WHO guidelines, transition to EAT-Lancet diet in 5 regions may result in  - a lower cost of recommended intake (per capita daily income) | [85] |
| Caldeira 2025 | Brazil | 2017–2018 | Cross-sectional study | 57 ,920 households | Relatively higher EAT-Lancet diet score | Relatively lower EAT-Lancet diet score | 2017/18 Brazilian Household Budget Survey (7‑day household food purchase diaries); Brazilian Food Composition Table (TBCA) | Cost/100 kcal and total daily cost; linear regression of cost (BRL) on 1-point increments in EAT-Lancet score, stratified by income tertile | Prices of an EAT-Lancet diet Affordability of an EAT-Lancet diet | Transition to EAT-Lancet diet may result in - A BRL 0.38 decrease in diet cost for every 1-point increase in total EAT-Lancet score - Moving from the lowest to the highest adherence tertile cuts daily diet cost by BRL 1.10 in middle-income households and BRL 1.58 in the lowest-income households - National mean diet cost ≈ BRL 0.65 per 100 kcal; Northeast and rural areas show the lowest cost | [193] |
| Dehnavi 2025 | Iran | 2022–2023 | Cross-sectional study | 398 adults, aged ≥ 60 years | Relatively higher EAT-Lancet diet score | Relatively lower EAT-Lancet diet score | 168‑item semi‑quantitative FFQ; Planetary-Health-Diet Index computed from EAT-Lancet targets | Daily diet cost (Rials) = grams consumed × 2022 average retail price (Iran Statistics Center & market audit) | Average cost of the EAT-Lancet diet (dollars)  Affordability of an EAT-Lancet diet | Transition to EAT-Lancet diet may result in - A 21% lower mean daily food cost in the highest vs lowest adherence tertile (1,975,000 Rials vs 2,503,000 Rials) - Each 10-point increase in the Planetary-Health-Diet Index reduces daily cost by ≈ 343 870 Rials after energy adjustment - Cost advantage is lost in single-person and very-low-income households (β not significant) | [194] |
| Deng 2025 | 176 countries | 2020–2070 | Modelling study | - | EAT-Lancet diet | Business-as-usual diet (BaU) | FAOSTAT data linked to MAgPIE land-use model | MAgPIE Model: income and food expenditure data (% of income; €/cap year) | Average per capita food expenditure in 2050: Baseline vs. EAT-Lancet diet (€ per year) Affordability of an EAT-Lancet diet | Transition to EAT-Lancet diet may result in - A 2.6 % rise in global average per-capita food expenditure by 2050 (from € 1 018 to € 1 044 year⁻¹) - Short-term (to 2030) affordability worsens by up to +13% of household income in many emerging economies - By 2070, affordability improves by +9% to +63% across all income groups as prices fall and incomes rise | [161] |
| Herforth 2025 | Global (173 countries) | 2021 (ICP price round) | Cross-sectional study | - | EAT-Lancet diet | Healthy Diet Basket (HDB) | 2021 International Comparison Program (ICP) | 2021 purchasing power parity (PPP)-adjusted diet cost (US$ / person day) | Cost of the EAT-Lancet diet (dollars) Affordability of an EAT-Lancet diet | Transition to EAT-Lancet diet may result in - An average cost of US$ 4.48 person⁻¹ day⁻¹ for the EAT-Lancet basket (median US$ 4.27), versus US$ 3.68 for the HDB - Approximately 2.8 billion people worldwide cannot afford the least-cost EAT-Lancet basket at 2021 prices - In low-income countries, the basket cost equals >100 % of current average food spending, signaling severe affordability constraints | [162] |
| Morales-Ríos 2025 | Mexico | 2018 | Cross-sectional study | 74,991 nationally representative households | EAT-Lancet diet | Current diet | Mexican National Household Income and Expenditure Survey (MNHIES 2018) | Demand System (LA/AIDS) developed by Deaton and Muellbauer | Prices of an EAT-Lancet diet; Affordability of an EAT-Lancet diet | Transition to EAT-Lancet diet may result in:  - Vegetable intake increased 9.4–28.3% under 10–30% subsidy  - To meet dietary recommendations, prices would need to drop by 39.7% (fruits), 20.0% (vegetables), 118.7% (legumes)  - Fish/seafood intake also below target, requiring large price reduction (exact % not specified) | [195] |
| Rochefort 2025 | Canada | 2015–2017 | Cross-sectional study | 1,147 adults, aged 18–65 years | Relatively higher EAT-Lancet diet score | Relatively lower EAT-Lancet diet score | Dietary intake via repeated 24-h recalls using web platform (R24W); food prices linked to Nielsen retail scanner database (Québec, 2015–2016) | Energy-adjusted total diet cost estimated per individual; multivariable linear regression modeled diet cost across EAT-Lancet adherence quartiles | Average cost of the EAT-Lancet diet (CAD); Affordability of an EAT-Lancet diet | Transition to EAT-Lancet diet may result in:  - Diet cost increased by +$1.00 CAD/day when moving from 25th to 75th percentile adherence  - Cost of vegetables: +$1.60/day; fish/plant protein: +$1.40; fruits: +$0.90; free sugars: +$1.60; whole grains: +$0.40; poultry/eggs: −$1.20 (saving) | [196] |

Abbreviation: FAO, Food and Agriculture Organization of the United Nations

**Table S7. Summary of articles investigating the gap between current global diets and the EAT-lancet planetary health diet**

| **Study** | **Country** | **Assessment period** | **Study type** | **Cohort size, n** | **Study subject** | **Study diet** | **Compared diet** | **Diet exposures** | **Dietary data sources or assessment method** | **Outcomes** | **Main findings** | **Reference** |
| --- | --- | --- | --- | --- | --- | --- | --- | --- | --- | --- | --- | --- |
| Willett 2019 | 195 countries and territories | 2016 | Comparative analysis | Over 200 million | General population of all ages | Current diet | EAT-Lancet diet | The quantity consumed from each food groups | Dietary recall, household budget, and FFQs | Diet gap (%) between current diets and EAT-Lancet diet intakes of food groups and nutrients | Compared to the EAT‐Lancet recommendations, the study diet contains Global results: - excess: red meat, starchy vegetables, and eggs  - insufficient: poultry, total dairy, fish, vegetables, fruit, legumes, whole grains, and nuts East Asia Pacifc results: - excess: red meat, starchy vegetables, eggs, and fish - insufficient: poultry, total dairy, vegetables, fruit, legumes, whole grains, and nuts  South Asia results:  - excess: starchy vegetables  - insufficient: red meat, eggs, poultry, total dairy, fish, vegetables, fruit, legumes, whole grains, and nuts  Sub-Saharan Africa results:  - excess: red meat and starchy vegetables  - insufficient: eggs, poultry, total dairy, fish, vegetables, fruit, legumes, whole grains, and nuts  Latin America and Caribbean results:  - excess: red meat, starchy vegetables, eggs, and poultry  - insufficient: total dairy, fish, vegetables, fruit, legumes, whole grains, and nuts  Middle East and North Africa results:  - excess: red meat, starchy vegetables, eggs, and poultry  - insufficient: total dairy, fish, vegetables, fruit, legumes, whole grains, and nuts  Europe and central Asia results:  - excess: red meat, starchy vegetables, eggs, poultry, and total dairy  - insufficient: fish, vegetables, fruit, legumes, whole grains, and nuts  North America results:  - excess: red meat, starchy vegetables, eggs, poultry, and total dairy  - insufficient: fish, vegetables, fruit, legumes, whole grains, and nuts | [197] |
| Athare 2020 | India | 2011–2012 | Cross-sectional study | - | 182,771 households | Current diet (11 types of Indian current diets) | EAT-Lancet diet | The energy intake from each food groups | Type 1 and Type 2 schedules (30-day recall) | Difference in food groups | Indian diets deviate from a healthy reference diet either with too much or too little consumption of certain food groups. Compared to the EAT‐Lancet recommendations, the study diet contains - higher: cereals, sugar, and dairy products  - lower: fruits and vegetables, pulses, and nuts | [198] |
| Batlle–Bayer 2020 | Spain | 2019–2020 | Modelling study | - | Household and food-away-from-home consumptions | Current diet | EAT-Lancet diet | The quantity consumed from each food groups | Spanish Ministry of Agriculture | Difference in food groups | Compared to the EAT‐Lancet recommendations, the study diet contains - higher: red meat and energy intake  - lower: plant-based food products | [110] |
| Poole 2020 | USA | 2014–2015 | Cross-sectional study | - | 1,207 School (elementary, middle and high school) | Current diet | EAT-Lancet diet | The quantity consumed from each food groups | A five days of lunch menus during a specific week | Difference in food groups | Compared to the EAT‐Lancet recommendations, the study diet contains - excess: dairy, fruit, refined grains, red meat, and starchy vegetables  - insufficient: whole grains, legumes, vegetables, and nuts | [199] |
| Sharma 2020 | India | 2011–2012 | Cross-sectional study | - | 102,000 Household consumption | Current diet | EAT-Lancet diet | The energy intake from each food groups | 30-day and 7-day recalls | Difference in food groups | Compared to the EAT‐Lancet recommendations, the study diet contains - excess: cereals  –not enough: proteins, fruits, and vegetables | [200] |
| Springmann 2020 | 85 countries | 2019 | Modelling study | - | - | Current diet | EAT-Lancet diet | Sufficient and deficiency of food components intake | Online repository of National food based dietary guidelines maintained by the Food and Agriculture Organization of the United Nations | Percentage difference between current diet and EAT-Lancet diet intakes of food groups | Compared to the EAT‐Lancet recommendations, the study diets in different regions contain Global results: - higher: eggs, sugar, meat, poultry, red meat, and processed meat  - lower: legumes, whole grains, milk, fish, nuts and seeds, fruits, and vegetables Europe results: - higher: eggs, sugar, meat, poultry, red meat, and processed meat  - lower: legumes, whole grains, milk, fish, nuts and seeds, fruits, and vegetables North America results: - higher: eggs, sugar, meat, poultry, red meat, and processed meat  - lower: legumes, whole grains, milk, fish, nuts and seeds, fruits, and vegetables Near East results: - higher: eggs, sugar, meat, red meat, processed meat, and vegetables  - lower: legumes, whole grains, milk, fish, nuts and seeds, poultry and fruits Asian Pacific results: - higher: eggs, sugar, meat, poultry, red meat, and processed meat  - lower: legumes, whole grains, milk, fish, nuts and seeds, fruits, and vegetables Latin America results: - higher: eggs, sugar, meat, red meat, and processed meat  - lower: legumes, whole grains, milk, fish, nuts and seeds, fruits, poultry, and vegetables Africa results: - higher: eggs, sugar, meat, poultry, red meat, and processed meat  - lower: legumes, whole grains, milk, fish, nuts and seeds, fruits, and vegetables | [201] |
| Batis 2021 | Mexico | 2011–2018 | Cross-sectional study | 10,086 | General population of all ages | Current diet | EAT-Lancet diet | The energy intake from each food groups | 24-h–diet recall | Difference in food groups | Compared to the EAT‐Lancet recommendations, the study diet contains - higher: animal protein sources, dairy, discretionary food, fats and oils, sauces and spreads, and sugar‐sweetened beverages  - lower: fruit, vegetables, grains and starchy vegetables, legumes, and nuts | [186] |
| Castellanos–Gutiérrez 2021 | Mexico | 2012 and 2016 | Cross-sectional study | 4,478 | General population/adults, females and males aged ≥ 20 years | Current diet | EAT-Lancet diet | The energy intake from each food groups | 5-step multiple-pass 24-h dietary recall | Diet gap (%) between current diets and EAT-Lancet diet intakes of food groups | Mexican adults have a diet that is far from EAT-Lancet diet. Compared to the EAT‐Lancet recommendations, the study diet contains - higher: energy intake, grains, dairy, added sugars, red meat, chicken and other poultry, eggs, and processed meats  - lower: fish, high-fiber grains, fruits, vegetables, legumes, nuts, tubers and starchy vegetables, added fats, dark green vegetables, and red and orange vegetables | [202] |
| Colombo 2021 | Sweden | 2016–2017 | Cross-sectional study | 3,099 | Adolescents, girls and boys aged 10–18 years | Current diet | EAT-Lancet diet | The energy intake from each food groups | web-based 24-h recall | Difference in food groups | The average relative deviation for all food groups between the EAT diet and the optimized diets (i.e., the sum of absolute relative deviations divided by the number of food groups compared) was 134, 136, 127, and 181 percent for the “Omni+”, “Pesc+”, “Veg+” and “Plant” diets, respectively. Compared to the EAT‐Lancet recommendations, the study diet contains - higher: potatoes, dairy foods, eggs, fish and added sugars  - lower: whole grain foods, vegetables, fruits, legumes, nuts, and added fats | [203] |
| de Pee 2021 | Indonesia | 2018 | Modelling study | - | - | Current diet | EAT-Lancet diet | The energy intake from each food groups | Food balance sheets from FAO and Indonesia Household Income and Expenditure Survey | Difference in food groups | The EAT-Lancet planetary health diet had a much more diversity than the current diet.  Compared to the EAT‐Lancet recommendations, the study diet contains - lower: rice  - insufficient: dairy, vegetable oil, and pulses, nuts, and seeds | [115] |
| Kassem 2021 | Denmark | 2009–2013 | Observational Study | - | General population of all ages | Current diet | EAT-Lancet diet | The quantity consumed and energy from each food groups | FAOSTAT | Difference in food groups | Compared to the EAT‐Lancet recommendations, the study diet contains - higher: animal fats (89%), meat (68%), eggs (68%), milk and milk products (62%), and sugars (63%) - insufficient: pulses, nuts, oil crops (454%) and crop oils (116%) | [116] |
| Kidd 2021 | New Zealand | 2002 and 2008–2009 | Modelling study | - | 210–237 households | Current diet | a. Flexitarian diet (modelled from EAT-Lancet diet guideline) b. Vegan diet (modelled from EAT-Lancet diet guideline) | The quantity consumed and energy from each food groups | Current diet: Survey data from the most recent NZ National Nutrition Survey (2008/2009)  Healthy diet: 2015 New Zealand Eating and Activity Guidelines  EAT diet: Modelled from the EAT-Lancet ‘Planetary Diet’ guidelines | Difference in food groups and nutrients | Compared to the flexitarian diet (modelled from EAT-Lancet diet guideline), the study diet contains - higher: saturated fat, carbohydrate, and sodium  - lower: fiber  Compared to the vegan diet (modelled from EAT-Lancet diet guideline), the study diet contains  - higher: saturated fat, carbohydrate, and sodium  - lower: fat and fiber | [117] |
| Kretschmer 2021 | Germany | - | Cross-sectional study | 40 | Undergraduate student | Current diet | EAT-Lancet diet | The consumption frequency of each food groups | Online scale consists of ﬁve closed–ended questions with a three–point Likert | Difference in food groups | Compared to the EAT‐Lancet recommendations, the study diet contains - higher: vegetables and fruits and starchy vegetables  - lower: whole grains, dairy foods, animal soured protein, plant soured protein, fatty acids, and added sugar | [204] |
| Llanaj 2021 | Albania | 2015–2016 | Cross-sectional study | 298 | Young adults, aged 18–24 year | Relatively 1/3 lower adherence to EAT-Lancet diet | Relatively 1/3 higher adherence to EAT-Lancet diet | The quantity consumed from each food groups | 24-h recalls | Percentages and numbers of participants in group of low, moderate or high adherence to EAT-Lancet diet | - Low adherence to EAT-Lancet diet: females (28, 11.1%) and males (5, 13.5%) - Moderate adherence to EAT-Lancet diet: females (175, 69.4%) and males (25, 67.6%)  - High adherence to EAT-Lancet diet: females (49, 19.4%) and males (7, 18.9%) | [205] |
| Llanaj 2021 | Hungary | 2013–2015 | Cross-sectional study | 703 | Participants, aged 20–64 years | Relatively 1/3 lower adherence to EAT-Lancet diet | Relatively 1/3 higher adherence to EAT-Lancet diet | The quantity consumed from each food groups | Two 24-h recalls | Percentages and numbers of participants in group of low, moderate or high adherence to EAT-Lancet diet | - Low adherence to EAT-Lancet diet: 324, 90.3%  - Moderate adherence to EAT-Lancet diet: 35, 9.7%  - High adherence to EAT-Lancet diet: 0, 0% | [206] |
| Sheng 2021 | China | 2019 | Comparative analysis | - | General population of all ages | Current diet | EAT-Lancet diet | The quantity consumed from each food groups | The China Statistical Yearbook (National Bureau of Statistics of China, 1998–2020) | Difference in food groups and nutrients | Compared to the EAT‐Lancet recommendations, the study diet contains - excess: meat  - insufficient: whole grain, fruit, nut, and milk  - higher: protein and fat  - lower: carbohydrate | [73] |
| Tepper 2021 | Israel | - | Comparative analysis | 348 | Men and women, aged 20–45 years | Current diet | EAT-Lancet diet | The quantity consumed from each food groups | A 115-item FFQ | Difference in food groups | Compared to the EAT‐Lancet recommendations, the study diet contains - excess: vegetables, fruits, dairy, poultry, saturated fat, and added sugar - insufficient: nuts  - higher: meat, eggs, fish, unsaturated fats - lower: grains, starchy vegetables, legumes | [207] |
| Vitale 2021 | Italy | 2015–2017 | Comparative analysis | - | General population of all ages | Current diet | EAT-Lancet diet | The quantity consumed from each food groups | FAOSTAT | Difference in food groups | Compared to the EAT‐Lancet recommendations, the study diet contains - excess: beef and pork meat eggs, milk and dairy products, animal fat, tropical oils, and sugars  - higher: poultry, ﬁsh,and non-tropical oils  - lower: fruits, vegetables, legumes, and nuts  - insufficient: beef and pork meat, animal fat, and sugar | [120] |
| Ali 2022 | Gimbia | 2015–2016 | Cross-sectional study | 12,713 | Households in The Gambia (data collected from both rural and urban areas) | Current diet | EAT-Lancet diet | The total energy, the quantity consumed and energy from each food groups | 7-day recall questionnaire | Difference in food groups | Adherence to the EAT-Lancet dietary recommendations) was very low, with a mean composite index of 10.1 out of 48. Compared to the EAT‐Lancet recommendations, the study diet contains - excess: refined grains and added sugars  - insufficient: fruits, vegetables, nuts, dairy, poultry, and beef and lamb | [208] |
| Bäck 2022 | Finland | 2015–2016 | Cross-sectional study | 862 | Children (pre-schoolers), aged 3– 4: 460 aged 5–6: 402 | Current diet | EAT-Lancet diet | The mean energy intake and quantity consumed from each food groups | 3-day and 2-day food records AivoDiet software | Difference in food groups and nutrients | Compared to the EAT‐Lancet recommendations, the study diet contains  - excess: red meat, dairy foods, tubers, and added sugars  - insufficient: whole grains, vegetables, legumes, nuts, and unsaturated oils | [209] |
| Cleghorn 2022 | New Zealand | 2008–2009 | Modelling study | The New Zealand population alive in 2011 (N = 4:4 million) | General population/adults (with separate optimization for Māori males, Māori females, non-Māori males, and non-Māori females) | Current diet | EAT-Lancet diet | The quantity consumed from each food groups | FFQ from the 2008/09 NZ Adult Nutrition Survey | Difference in food groups | Compared to the EAT‐Lancet recommendations, the study diet contains - excess: red meat, processed meat, sugar-sweetened beverages, and sodium  - insufficient: fruit, vegetables, and polyunsaturated fat | [122] |
| Dhar 2022 | Bangladesh, India, Pakistan, Sri Lanka, Nepal, Bhutan | 1960–2010 | Modelling study | - | General population of all ages | Current diet | EAT-Lancet diet | The quantity consumed from each food groups | Food balance sheets from FAO | Difference in food groups | Compared to the EAT‐Lancet recommendations, the study diets in 6 nations contain  Bangladesh results: - excess: cereals and starchy roots  - insufficient: oil crops and pulses, vegetables, fruits, and other plant products India results: - excess: cereals  - insufficient: oil crops and pulses, vegetables, fruits, and other plant products Pakistan results: –excess: cereals  - insufficient: oil crops and pulses, vegetables, fruits, milk and dairy Sri Lanka results: - excess: cereals, oil crops and pulses  - insufficient: vegetables, fruits, and other plant products Nepal results: - excess: cereals and starchy roots  - insufficient: oil crops and pulses, vegetables, fruits, and other plant products Bhutan results: - excess: cereals and starchy roots  - insufficient: oil crops and pulses, vegetables, fruits, and other plant products | [123] |
| Hendrie 2022 | Australia | 2011–2013 | Comparative analysis | 5,920 | General population/adults, females and males aged 19–50 years | Current diet | EAT-Lancet diet | The quantity consumed and energy intake from each food groups | 24‐h dietary recalls | Difference in food groups and nutrients | Compared to the EAT‐Lancet recommendations, the study diet contains - higher: dairy and red meat  - lower: whole grains, meat and alternatives, and unsaturated fats | [169] |
| Ibarrola–Rivas 2022 | Mexico | 2016 | Comparative analysis | 3,646 | General population of all ages | Current diet | EAT-Lancet diet | The quantity consumed and energy intake from each food groups | 24‐h dietary recalls | Difference in food groups | Compared to the EAT‐Lancet recommendations, the study diet contains - excess: red meat, processed foods, and sugars  - insufficient: fruits, vegetables, legumes, and nuts | [210] |
| Mapes 2022 | 142 countries | 2010 | Modelling study | - | General population of all ages | Current diet | EAT-Lancet diet | The quantity consumed from each food groups | FAOSTAT | Difference in food groups | Compared to the EAT‐Lancet recommendations, the study diet contains - excess: sugars and meats  - insufficient: legumes, nuts, seeds, and grains (especially in low-income regions) | [211] |
| Marchioni 2022 | Brazil | 2017–2018 | Cross-sectional study | 46,164 | General population, females and males aged ≥ 10 | Current diet | EAT-Lancet diet | Sufficient and deficiency of food components intake [mean (95% CI)] | 24‐h dietary recalls  (two times) | Difference in food groups | Adherence to the EAT-Lancet diet is low in Brazil, with the average EAT-Lancet diet score being about 30% of the maximum possible score. Particularly, for five Brazilian regions, North 29.9%, Northeast 28.8%, Southeast 31.6%, South 29.9%, Middle-East 32.3% (Higher adherence was observed in women, older age groups, and higher–income individuals) | [7] |
| Read 2022 | USA | 2012 | Modelling study | - | - | Current diet | EAT-Lancet diet | The energy intake from each food groups | USEEIO version 2.0 input-output model | Difference in food groups | Compared to the EAT‐Lancet recommendations, the study diet contains - higher: meat, dairy products, and added fats  - lower: fruits, grains, nuts, and vegetables | [125] |
| Stubbendorff 2022 | Sweden | 1991–1996 | Cross-sectional study | 22,421 | General population/adults, aged 45–73 (men aged 46–73 years and women aged 45–73 years) | Relatively lower 1/5 EAT-Lancet diet score | Relatively higher 1/5 EAT-Lancet diet score, Q1–Q5 (≤ 13, 14–16, 17–19, 20–22, and ≥ 23) | The quantity consumed from each food groups | 7-day food diary and 168-item FFQ | Difference in food groups | Low compliance with the EAT-Lancet dietary recommendations among the Swedish population women had higher adherence to the EAT-Lancet diet, with a mean score of 18.5 (SD, ± 3.3), while the mean score for men was 16.8. Compared to the EAT‐Lancet recommendations, the average current diet contains - excess: poultry and fish  - insufficient: legumes, nuts, whole grains, pork, beef and lamb, vegetable, and fruit | [8] |
| Tucci 2022 | Italy | - | Modelling study | - | General population of all ages | Current diet | EAT-Lancet diet | The quantity consumed from each food groups | Italian National Food Consumption Survey 2005–06 data | Difference in food groups | Compared to the EAT‐Lancet recommendations, the study diet contains - higher: white meat, dairy, and sugars  - lower: nuts and legumes | [129] |
| Tuninetti 2022 | 172 countries | 1961–2018 | Comparative analysis | - | General population of all ages | Current diet | EAT-Lancet diet | The quantity consumed and energy intake from each food groups | FAOSTAT | Difference in food groups | Compared to the EAT‐Lancet recommendations, the study diet contains - excess: animal products, fats, and sugars (in high-income countries)  - insufficient: legumes, nuts, and fruits (in both high- and low-income countries) | [130] |
| Young 2022 | UK | 2015–2019 | Secondary analysis | 328 | General population of all ages | Current diet | EAT-Lancet diet | Adherence to EAT-Lancet diet (evaluated using the World Index for Sustainability and Health) | European Prospective Investigation into Cancer AND NUTRITION-Norfolk FFQ FETA software | Difference in food groups and nutrients | Approximately 40%of the participants met the EAT-Lancet dietary guidelines. Compared to the EAT‐Lancet recommendations, the study diet contains - excess: red meat, whole grains, fish, poultry, and SFAs  - insufficient: nuts, legumes, eggs, unsaturated fats, and vegetables | [212] |
| Bellamy 2023 | UK | - | Observational study | 113 | General population of all ages | Current diet (diets for randomly selected control group) | EAT-Lancet diet (diets of Community Supported Agriculture members) | The energy intake from each food groups and quantity consumed from nutrients | 24‐h dietary recalls (Intake24 software) | Difference in food groups | Compared to the EAT‐Lancet recommendations, the control group diet contains - excess: meat (beef, lamb and pork), dairy, sugars, fat, saturated fat, protein and carbohydrates  - insufficient: vegetable and legume | [131] |
| Berthy 2023 | France | 2009–1015 | Cross-sectional study | 98,465 | General population/adults, females and males aged ≥ 18 | Current diet | EAT-Lancet diet | The total energy intake, the quantity consumed from each food groups | 24‐h dietary recalls  (3 non-consecutive, 2 weekdays and 1 weekend day) | Difference in food groups and nutrients | Compared to the EAT‐Lancet recommendations, the study diet contains - higher: animal protein  - lower: whole grains, fruits, vegetables, legumes, nuts, seafood, red meat, pork, poultry, eggs, sugars, vitamin B9, vitamin C, calcium, and fiber | [171] |
| Campirano 2023 | Mexico | 2004–2006 | Observational study | 1,908 | General population/adults, females and males aged 19–59 | Current diet | EAT-Lancet diet | Sufficient and deficiency of food components intake [difference (95% CI)] | A 234-item semiquantitative FFQ | Difference in food groups and nutrients | The study indicates a moderate adherence (representing 57.5%) to the EAT-Lancet recommendations in Mexico. Compared to the EAT‐Lancet recommendations, the study diet contains - excess: saturated fat, legumes, nuts, and added sugars  - insufficient: unsaturated fats, poultry, eggs, fish, dairy, vegetables, and cereals high in fiber | [213] |
| Delgermaa 2023 | Mongolia | 2019 | Observational study | 11,197 | General population of all ages | Current diet | EAT-Lancet diet | The quantity consumed from each food groups | Urban areas: 30-day dietary record  rural areas: 7-day dietary record | Difference in food groups and nutrients | Compared to the EAT‐Lancet recommendations, the study diet contains - excess: red meat  - insufficient: vegetables and fruits | [214] |
| Ganpule 2023 | North and South India | 2018–2019 | Cross-sectional study | 8,762 | General population/adults, females and males aged 52.4 ± 11.7 (SD) years | Current diet | EAT-Lancet diet | The quantity consumed and energy from each food group | UDAY cohort study a FFQ containing 23 groups | Likelihood of deficit or excess in consumption compared to the EAT‐Lancet recommendations | More than 80% of the participants consumed all nine EAT‐Lancet recommended food groups in varied quantities, except for the non‐vegetarian group. Compared to the EAT‐Lancet recommendations, the study diet contains - excess: dairy and added fats  - insufficient: vegetables and fruits | [215] |
| Gatto 2023 | Global | 2030 | Modelling study | - | Different regions of the world | Business-as-usual scenario diet (2030) | EAT-Lancet diet | The total calories and intake across- all commodity groups. | The FAO global flows of food loss and waste database (2019) combined with literature review | Difference to the EAT-Lancet reference diet | Compared to the EAT‐Lancet recommendations, the study diets in different regions contain  Global results: excessive intake across- all commodity groups (cereals, Horticulture, fats, sugars, meat—ruminants, meat—non- ruminants, dairies, and fish). Total intake in the business-as-usual is 20% higher in 2020. Regional results: - excess: calories, cereals, fats, sugars, meat—ruminants in all regions (EU27, SEA, INDA, NAMO, LAC, REUCA, MENA, and SSA)  - excess: meat—non- ruminants in all regions except for SSA, and fish in all regions except for MENA and SSA - excess: horticulture in EU27, SEA, REUCA, and SSA  - insufficient: horticulture in INDA, NAMO, LAC, and MENA  - excess: dairies in EU27, NAMO, LAC, and REUCA  - insufficient: dairies in SEA, INDA, MENA, and SSA | [133] |
| Geibel 2023 | Denmark and Ireland | 2050 | Modelling study | - | Regions and countries | Benchmark diet 2050 | EAT-Lancet planetary health diet | The total calories and intake of all food groups. | Global trade analysis project 10 database | Difference in food groups | Compared to the EAT‐Lancet recommendations, the study diets in different regions contain  Ireland results: - excess: total calories, vegetable oils, sugar, beef, lamb, pork, poultry, milk and dairy products, and eggs  - insufficient: wheat, other grains, rice, and horticulture Denmark results: Compared to the EAT‐Lancet recommendations, the study diet contains: - excess: Total calories, sugar, beef, lamb, pork, poultry, milk and dairy products, eggs, fish  - insufficient: wheat, other grains, rice, horticulture, vegetable oils | [134] |
| Headey 2023 | Four East African countries: Ethiopia, Kenya, Tanzania, and Uganda | 2014–2017 | Cross-sectional study | - | 41,449 households | Current diet | EAT-Lancet diet | The total energy, the quantity consumed and energy from each food group | The Ethiopia Socioeconomic Survey 2015–16, the Kenya Integrated Household Budget Survey 2015–16, the Tanzania National Panel Survey 2014–15, and the Uganda National Household Survey 2016–17  7-day food consumption recall. | Difference to the EAT-Lancet reference diet | Compared to the EAT‐Lancet recommendations, the study diet contains - excess: calories, starchy staples, and refined grains  - insufficient: diversity | [188] |
| Ludwig–Borycz 2023 | US (Minnesota) | 2017–2018 | Cross-sectional study | 1,308 | General population/adults, females and males aged 22.1 ± 2.0 (SD) years | Current diet | EAT-Lancet diet | Consumption quantities by food group | EAT 2010–2018 a 149-item semi-quantitative FFQ | Difference in food groups | Compared to the EAT‐Lancet recommendations, the study diet contains - excess: meat, eggs, and added sugar  - insufficient: whole grains, fish, legumes, soya, nuts and plant-based proteins | [216] |
| Nomura 2023 | Japan | 2019 | Cross-sectional study | 4,927 | General population/adults, females and males aged ≥ 20 years | Current diet | EAT-Lancet diet | Average dietary intake by food group | The Japan National Health and Nutrition Survey semi-weighed household dietary records | Diet gap (%) of the global reference of the PHD | Compared to the EAT‐Lancet recommendations, the study diet contains - excess: red meat  - insufficient: – | [217] |
| Quarpong 2023 | Kenya | 2014 | Cross-sectional study | 242 | Lactating mothers | Current diet | EAT-Lancet diet | Intake of 11 EAT‐Lancet food groups | Single multiple pass method 24‐h dietary intake | Comparison of cost of EAT-Lancet diet and mothers' current diets | Compared to the EAT‐Lancet recommendations, the study diet contains - excess: grains  - insufficient: fruits and vegetables  - within recommendations: tubers, fish, beef and dairy  - closer to lower bounds: chicken, eggs, legumes and nuts | [17] |
| Schön 2023 | Germany | - | Modelling study | - | General population (entire population of Hesse, Germany) | Current diet | EAT-Lancet diet recommended by the EAT-Lancet Commission, adjusted to 2150 kcal (86%) | The quantity consumed from each food groups | - | Difference in food groups | Compared to the EAT‐Lancet recommendations, the study diet contains - excess: meat and dairy  - insufficient: fruits, vegetables, nuts, and legumes | [138] |
| Stewart 2023 | the United Kingdom | 1986–2017 | Modelling study | - | General population (UK population) | Current diet | EAT-Lancet diet | The quantity consumed from each food groups | FAO | Difference in food groups | UK population moved closer towards the EAT-Lancet Diet over the period 1986–2017, even though the compliance with the EAT-Lancet diet was still very low.  Compared to the EAT‐Lancet recommendations, the study diet contains - excess: beef, lamb, pork, tubers, starchy vegetables, and dairy products  - insufficient: fruits, vegetables, nuts, and legumes | [139] |
| Stone 2023 | USA | - | Modelling study | - | General population (study population from Des Moines Metropolitan Statistical Area, a six–county area in central Iowa with a 2020 population of ≈ 700,000) | Current diet | EAT-Lancet diet | The quantity consumed from each food groups | FoodCarbonScope™ software | Difference in food groups | Compared to the EAT‐Lancet recommendations, the study diet contains - excess: meat and dairy  - insufficient: legumes, fruits and vegetables | [140] |
| Wright 2023 | Norway | 2007–2019 | Cross-sectional study | 1,413 | Children, females and males aged 2 years | Current diet | EAT-Lancet diet | The quantity consumed and energy from 11 EAT-Lancet food groups | A semi-quantitative FFQ | Difference in food groups and nutrients | Compared to the EAT‐Lancet recommendations, the study diet contains - excess: fruit (include juice), dairy products, red meat, eggs, fish, sweets, total fats, vitamin E, vitamin B1, vitamin B9, and iron  - insufficient: grain products, whole–grain, potatoes, vegetables, legumes, nuts, added vegetable fats, poultry, drinking water, protein, total carbohydrates, vitamin A, vitamin D, vitamin B2, vitamin B3, vitamin B6, vitamin B12, vitamin C, calcium, zinc, selenium or iodine | [175] |
| Abe–Inge 2024 | Canada | - | Observational study | - | General population, females and males aged ≥ 2 | Current diet | EAT-Lancet diet | The quantity consumed from each food groups | FAOSTAT | Difference in food groups | Compared to the EAT‐Lancet recommendations, the study diet contains - higher: red meat, added sugar, dairy, roots and tubers, eggs and chicken  - lower: vegetables, legumes, nuts, and oils (added fats) | [218] |
| Bakman 2024 | Brazil | 2017–2018 | Comparative analysis | - | General population/adults and adolescents, aged over 10 years | Current diet | EAT-Lancet diet | The quantity consumed from each food groups | the Family Budget Research 2017–2018 | Difference in food groups | Compared to the EAT‐Lancet recommendations, the study diet contains - higher: rice, cereals, beans, bovine meat, pork meat. and poultry meat - lower: vegetables, potatoes, fruits, cashew nuts, breads and pasta, fishes, eggs, dairy products, and soybean grain and products | [142] |
| Beckmann 2024 | Thailand | 1961 and 2020 | Comparative analysis | - | - | Thai Food Pattern (TFP) in 1961 and 2020 | EAT-Lancet diet | The quantity consumed from each food groups | Food balance sheets from FAO | Difference in food groups | Compared to the EAT‐Lancet recommendations, the study diet contains - excess: sugar (+452%), red meat (+220%), grains (+143%), saturated oils (+20%) and eggs (+19%） - insufficient: vegetables (–63%), and unsaturated oils (–61%) | [219] |
| Bennett 2024 | UK &USA | The National Diet and Nutrition Survey (NDNS): 2016–2019; National Health and Nutrition Examination Survey (NHANES): 2017–2018 | Comparative analysis | UK = 1780;  USA = 4339 | Participants, aged ≥ 18 years | Current diet | EAT-Lancet diet | The quantity consumed from each food group | NDNS: 4-day weighed food diaries ; NHANES : two 24-h recalls | Diet gap (%) between current diets and EAT-Lancet diet intakes of food groups and nutrients across racial groups | Compared to the EAT-Lancet recommendations，  White group diet contains:  - higher: red & processed meat, pork, dairy, potatoes, added sugar  - lower: vegetables, fruit, unsaturated oils, legumes, nuts, whole grains, fish.  Black group diet contains:  - higher: red & processed meat, pork, poultry, added sugar.  - lower: vegetables, fruit, unsaturated oils, legumes, nuts, whole grains, fish  Asian group diet contains (overall best adherence among racial groups):  - higher: red & processed meat, pork, poultry, eggs, dairy, potatoes, added sugar  - lower: vegetables, fruit, unsaturated oils, legumes, nuts, whole grains, fish  Other group diet contains:  - higher: red & processed meat, pork, poultry, eggs, added sugar  - lower: vegetables, fruit, unsaturated oils, legumes, nuts, whole grains, fish | [220] |
| Bertoldo 2024 | USA | 2022 | Comparative analysis | - | 19 universities dining programs | Current diet | EAT-Lancet diet | The quantity consumed from each food groups | University dining programs in an Excel spreadsheet | Difference in food groups | Compared to the EAT‐Lancet recommendations, the study diet contains  - excess: beef (+ 557%), pork (+487%), poultry (+279%), and eggs (+193%)  - insufficient: legumes and nuts (–61%) and vegetables (–32%). | [143] |
| Caldeira 2024 | Brazil | 2017–2018 | Modelling study | - | 57,920 households | Current diet | EAT-Lancet diet | The quantity consumed from each food groups | Brazilian Household Budget Survey 2017/18 , and the Footprints of Foods and Culinary Preparations Consumed in Brazil database | Difference in food groups and nutrients | Compared to the EAT‐Lancet recommendations, the study diet contains - higher: carbohydrates, fiber, red meat, fruit, vegetables, grains and starchy vegetables, dairy, protein foods, fats and oils, and discretionary food - lower: protein, total fat, saturated fat, added sugar, sodium, sauces, dressings, spreads, sugar, and sugar-sweetened beverages | [144] |
| Chen 2024 | China | 2021 | Cross-sectional study | 3,150 | General population/ adults, females and males aged ≥ 18 | Current diet | EAT-Lancet diet | The quantity consumed from each food groups | Collected by a professional survey company in China (https://www.lediaocha.com/) | Difference in food groups | Compared to the EAT‐Lancet recommendations, the study diet contains  - higher: total grains, pork, beef and lamb, eggs, fish, vegetables, and fruits  - lower: chicken and other poultry, whole milk or equivalents, legumes, and vegetable oils | [190] |
| Colombo 2024 | Sweden | 2010–2011 | Modelling study | 1,797 | General population/adults, females and males aged 18–80 | Nationally representative Swedish dietary | EAT-Lancet diet | The energy contribution from each food groups | Swedish Food Agency’s Food composition database version Riksmaten Vuxna 2010–11 | Difference in food groups | Compared to the EAT‐Lancet recommendations, the study diet contains - excess: red and processed meat, and potatoes  - insufficient: fruits and vegetables | [141] |
| Eberle 2024 | Germany | 2017-2019 | Modelling study | - | General population of all ages | Current diet | EAT-Lancet diet | The quantity consumed from each food groups | FAO | Difference in food groups | Compared to the EAT‐Lancet recommendations, the study diet contains  - excess: dairy products, meat, and added sugar  - insufficient: vegetables | [148] |
| Gaona–Pineda 2024 | Mexico | 2006, 2012, 2016, and 2018 | Cross-sectional study | 16,520 | Adolescents, females (non-pregnant, non-lactating adolescents) and males aged 12–19 | Current diet | EAT-Lancet diet | The quantity consumed from each food groups | Semi-quantitative FFQ | Difference in food groups | Approximately 40% of the participants met the EAT-Lancet dietary guidelines.  Compared to the EAT‐Lancet recommendations, the study diet contains - excess: nuts and seeds, red meats, and added sugars, fiber and saturated fats  - insufficient: fruits, high-˙ cereals, and vegetables | [177] |
| Gu 2024 | Global | 2018 | Cross-sectional study | 171 countries (representing 98% of global population) | General population/adults, aged 25–75 years | Global EAT-Lancet diet score mean score | EAT-Lancet diet maximum score | The mean score from each food groups | Food and Agriculture  Organization’s (FAO) Food Balance Sheets and Global Dietary Database (GDD) | Percentage that achieves 100% point by food groups | Compared to the EAT‐Lancet recommendations, the study diet contains - excess: - - insufficient: nuts and seeds, non-soy legumes, soybeans/soy foods, and added unsaturated fat | [81] |
| Hoteit 2024 | Lebanon | 2022 | Cross-sectional study | 444 | Men and women, aged 18–64 years | Current diet | EAT-Lancet diet | The quantity consumed from each food groups | A 157-item semiquantitative FFQ | Difference in food groups | Compared to the EAT‐Lancet recommendations, the study diet contains - excessive: added sugar, beef, lamb, pork, grains, chicken and other poultry, and fruits  - insufficient: vegetables, dairy products, fish, nuts, and unsaturated oils, and whole grains | [221]  ˙ |
| Lengle 2024 | Norwegian | 2010–2011 | Cross-sectional study | 1,787 | General population/adults, females and males aged 18–70 | Current diet | EAT-Lancet diet | The energy contribution from each food groups | 24-h dietary recalls (two times) Nutrition calculation software system (KBS) | Difference in food groups | Compared to the EAT‐Lancet recommendations, the study diet contains - excess: potatoes, fruit, red meat, white meat, eggs, fish and shellfish, dairy, milk products, snacks, and sweets  - insufficient: grains, vegetables, legumes, all nuts, and cooking fats | [147] |
| Navruz-Varl 2024 | Türkiye | 2023 | Cross-sectional study | 601 | 582 females and 19 males | Current diet | EAT-Lancet diet | Adherence to EAT-Lancet diet (percentage of people in relatively higher 1/3 EAT-Lancet diet score) | 24‐h dietary recall | The percentage distribution of individuals in the EAT-Lancet diet score T3 group | About 20.2% – 52% of the participants from different Turkey’s geographical regions met the EAT-Lancet dietary T3 tertile score | [222] |
| Pitt 2024 | Sweden | 1998–2019 | Observational study | 68,175 | Two population-based cohorts: the Cohort of Swedish Men the Cohort of Swedish Men and the Swedish Mammography Cohort | Current diet | EAT-Lancet diet (Relatively higher 1/5 EAT-Lancet score, Q1–Q4 (≤ 6, 7–8, 9–10, and ≥ 11) | The energy contribution from each food groups | A 96-item FFQ | Difference in food groups | Low adherence group (≤ 6 points): 28.7% men, 7.4% women. High adherence group (≥ 11 points): 1.6% men, 9.2% women. Compared to the EAT‐Lancet recommendations, the study diet contains: - higher: grains, potatoes, dairy products, red meat, eggs, and added sugars  - lower: vegetables, fruit, and nuts/peas | [39] |
| Richter 2024 | Germany | 2008–2011 | Cross-sectional study | 7,987 | General population/adults, females and males aged 18–79 | Current diet | EAT-Lancet diet | The quantity consumed from each food groups | A semi-quantitative FFQ | Percentage that achieves 100 point by food groups | Compared to the EAT-Lancet recommendations, the study diet contains  - excess: whole grains, milk equivalents, red meat, eggs, and added sugar  - insufficient: vegetables, nuts, legumes, and fish | [223] |
| Crosnier 2025 | Switzerland | 2018–2050 | Modelling study | - | General population of all ages | Current diet | EAT-Lancet diet | The quantity consumed from each food groups | FAO Food Balance Sheets & Swiss agricultural statistics and Life-Cycle Assessment model | Diet gap (%) between current diets and EAT-Lancet intakes of food groups | Compared to the EAT-Lancet recommendations, the study diet contains - higher: sugar (raw equivalent), milk equivalent, beef, lamb and pork, chicken and other poultry, eggs, wine and alcoholic beverages  - lower: cereals, seeds, vegetable oil, fruits, fish, seafood, nuts, pulses, potatoes, vegetables | [224] |
| de Lange 2025 | Bangladesh | 2022–2050 | Modelling study | – | General population of all ages | Current diet | EAT-Lancet diet | The quantity consumed from each food groups | Updated national consumption file mapped to GTAP–MAGNET sectors | Difference in food groups | Compared to the EAT-Lancet recommendations, the study diet contains - higher: cereals, roots, tubers - lower: vegetables, fruits, legumes, nuts, oil seeds, sugar, meat, fish , egg, red meat, poultry, fish, dairy | [160] |
| Fischer 2025 | 12 countries on 5 continents | 2016–2020 | Comparative analysis | – | General population of all ages | Current diet | EAT-Lancet diet | The energy intake from each food groups | FAO Food Balance Sheets (kcal cap⁻¹ d⁻¹) 2016—2020 | Difference in food groups | Compared to the EAT-Lancet recommendations, the food supplies contain - higher: grains, animal products and sugar - lower: vegetables, legumes, nuts & seeds; many countries also fall short in fruit and seafood | [225] |
| Hunziker 2025 | Argentina | Córdoba FFQ 2014 & National Survey of Household Income and Expenditure (NSHIE) data 2017–2018 | Cross-sectional study | 1,390 (human subjects) & 21,547 (households) | Participants, aged≥ 2 years & national households | Current diet | EAT-Lancet diet | The quantity consumed from each food groups | 127-item FFQ & 7-day expenditure survey & FAO balance-sheet | Difference in food groups | Compared to the EAT-Lancet recommendations, the study diet contains - higher: meat (13.4 times), starchy vegetables (2.4 times), eggs (2.3 times) - lower: vegetables, legumes, nuts and fish | [226] |
| Kersting 2025 | Germany | – | Comparative analysis | – | Children & adolescents, aged between 2—18 years | Optimised Mixed Diet (OMD) | EAT-Lancet diet | The energy consumed from each food groups | OMD 7-day menu scored with Planetary-Health-Diet Index (PHDI) | Difference in food groups & PHDI score | Compared to the EAT-Lancet recommendations, the study diet contains - higher: tubers & potatoes, dairy, red meat, animal fat, added sugars - lower: nuts & peanuts, legumes, whole-grains, green vegetables | [181] |
| Li 2024 | China | 1998–2021 (observed) + scenarios to 2030 | Modelling study | – | General populations, urban and rural residents | Current diet (Chinese Dietary Guidelines (CDG)) | EAT-Lancet diet | The quantity consumed from each food groups | Food expenditure and consumption from the China Household Survey, Rural Statistical Yearbook, and Statistical Yearbook; dietary cost inferred from market prices at the provincial level | Difference in food groups | EAT-Lancet shift narrows the urban-rural affordability gap;  Compared to the EAT-Lancet recommendations, the study diet contains - higher: meat, sugar and fat - lower: vegetables, fruit, legumes and nuts; | [151] |
| Samarathunga 2025 | Australia | 2011–2012 | Cross-sectional study | 463 (2–3 years );  776 (4–8 years) | Participants, aged 2–8 years | Current diet (current children’s diet) | EAT-Lancet diet | The quantity consumed from each food groups | Two 24-h recalls, weighted for national representativeness | Difference in food groups | Compared to the EAT-Lancet recommendations, the study diet contains - higher: red meat, dairy, added sugar, poultry, fruit - lower: whole-grains, vegetables, eggs, fish, legumes, nuts, unsaturated oils | [227] |

Abbreviations: CI, confidence interval; FAO, Food and Agriculture of the United Nations; FAOSTAT, Food and Agriculture Organization Corporate Statistical Database; FFQ, food frequency questionnaire; Q, quantile; SD, standard deviation; T, tertile

**Appendix 4 Key excluded studies**

**Table S8. Key excluded studies and reasons**

| **Study** | **Reason** | **Title** |
| --- | --- | --- |
| Garcia 2019 | Wrong outcomes | Eatlancet vs yes2meat: the digital backlash to the planetary health diet |
| Batlle-Baye 2020 | Wrong exposures | Nutritional and environmental co-benefits of shifting to “Planetary Health” Spanish tapas |
| Blackstone 2020 | Wrong outcomes | Comparing the Recommended Eating Patterns of the EAT-Lancet Commission and Dietary Guidelines for Americans: Implications for Sustainable Nutrition |
| Drewnowski 2020 | Unavailable data | Analysing the affordability of the EAT–Lancet diet |
| Goulding 2020 | Wrong design | The affordability of a healthy and sustainable diet: an Australian case study |
| Horikawa 2020 | Wrong exposures | Nutrient adequacy of Japanese schoolchildren on days with and without a school lunch by household income |
| Mirzaie-Nodoushan 2020 | Wrong exposures | Reducing water footprints through healthy and reasonable changes in diet and imported products |
| Moberg 2020 | Wrong exposures | Benchmarking the Swedish Diet Relative to Global and National Environmental Targets—Identiﬁcation of Indicator Limitations and Data Gaps |
| Yin 2020 | Wrong exposures | Diet shift: Considering environment, health and food culture |
| Zagmutt 2020 | Unavailable data | The eat-lancet commission’s dietary composition may not prevent noncommunicable disease mortality |
| Dimosthenopoulos 2021 | Wrong exposures | The beneficial short-term effects of a high-protein/ low-carbohydrate diet on glycaemic control assessed by continuous glucose monitoring in patients with type 1 diabetes |
| Toti 2021 | Wrong exposures | Longitudinal Effects of Dietary Advice on Wheelchair Basketball Athletes: Nutritional and Environmental Aspects |
| Trijsburg 2021 | Wrong exposures | Method for the Development of WISH, a Globally Applicable Index for Healthy Diets from Sustainable Food Systems |
| Chen 2022 | Wrong outcomes | How Far Are We from the Planetary Health Diet? A Threshold Regression Analysis of Global Diets |
| Conzuelo 2022 | Wrong exposures | Protein Quality Changes of Vegan Day Menus with Different Plant Protein Source Compositions |
| Franca 2022 | Wrong exposures | The EAT-Lancet Commission’s Planetary Health Diet Compared With the Institute for Health Metrics and Evaluation Global Burden of Disease Ecological Data Analysis |
| Hermelink 2022 | Wrong outcomes | A healthy diet for a growing population: a case study of Arua, Uganda |
| Kaartinen 2022 | Wrong exposures | Partial replacement of red and processed meat with legumes: a modelling study of the impact on nutrient intakes and nutrient adequacy on the population level |
| Liu 2022 | Wrong exposures | Dietary Transition Determining the Tradeoff Between Global Food Security and Sustainable Development Goals Varied in Regions |
| Paris 2022 | Wrong exposures | Changing dietary patterns is necessary to improve the sustainability of Western diets from a One Health perspective |
| van Selm 2022 | Wrong exposures | Circularity in animal production requires a change in the EAT-Lancet diet in Europe |
| Ambroz ̇y 2023 | Wrong exposures | Nutritional Status, Intentions and Motivations towards Adopting a Planetary Health Diet—A Cross-Sectional Study |
| Cundiff 2023 | Wrong design | The EAT-Lancet Commission’s Planetary Health Diet Compared With the Institute for Health Metrics and Evaluation Global Burden of Disease Ecological Data Analysis |
| Ferreira 2023 | Wrong outcomes | Adherence to the EAT-Lancet diet and its relation with food insecurity and income in a Brazilian population-based sample |
| Guedes 2023 | Wrong exposures | Promoting plant-based eating in meat-centric meal contexts: a field study |
| Kabasakal-Cetin 2023 | Wrong outcomes | Association between eco-anxiety, sustainable eating and consumption behaviors and the EAT-Lancet diet score among university students |
| Parker 2023 | Wrong exposures | Cross-sectional measurement of adherence to a proposed sustainable and healthy dietary pattern among United States adults using the newly developed Planetary Health Diet Index for the United States |
| Pauw 2023 | Unavailable data | Measuring changes in diet deprivation: New indicators and methods |
| Prag 2023 | Wrong exposures | Scenarios for Reducing Greenhouse Gas Emissions from Food Procurement for Public School Kitchens in Copenhagen |
| Rehner 2023 | Wrong outcomes | The Effect of a Planetary Health Diet on the Human Gut Microbiome: A Descriptive Analysis |
| Rieger 2023 | Wrong exposures | From fork to farm: Impacts of more sustainable diets in the EU-27 on the agricultural sector |
| Rosane 2023 | Unavailable data | Development of a methodology to compare and evaluate health and sustainability aspects of dietary intake across countries |
| Álvarez-Álvarez 2024 | Wrong exposures | Environmental impact of the diet of young Portuguese and its relationship with adherence to the Mediterranean Diet |
| Álvarez-Álvarez 2024 | Wrong exposures | The Evolution of Dietary Consumption in the Spanish Adult Population and Its Relationship with Environmental Sustainability |
| Asif 2024 | Wrong exposures | Development and evaluation of a web-based diet quality screener for vegans (VEGANScreener): a cross-sectional, observational, multicenter, clinical study |
| Bashiri 2024 | Wrong exposures | A Culturally Acceptable Shift in Diet to Reduce Land Footprint: an Optimization Study for Estonia |
| Bôto 2024 | Wrong exposures | Development of the Dietary Pattern Sustainability Index (DIPASI): A novel multidimensional approach for assessing the sustainability of an individual’s diet |
| Braga 2024 | Wrong outcomes | Feasibility of Using an Artiﬁcial Intelligence-based Telephone Application for Dietary Assessment and Nudging to Improve the Quality of Food Choices of Female Adolescents in Vietnam: Evidence from a Randomized Pilot Study |
| Cacau 2024 | Wrong exposures | Adherence to the EAT-Lancet sustainable diet and ultra-processed food consumption: findings from a nationwide population-based study in Brazil |
| Canyolu 2024 | Wrong exposures | Validity and reliability of the Sustainable healthy Diet (SHED) index by comparison with EAT-Lancet diet, Mediterranean diet in Turkish adults |
| Colizzi 2024 | Unavailable data | The EAT-Lancet Planetary Health Diet: Impact on Cardiovascular Disease and the Environment in the EPIC Cohort |
| Gonçalves 2024 | Unavailable data | The planetary health diet is associated with slower cognitive decline — but tied to income |
| Harrison 2024 | Wrong exposures | Effects of a cafeteria-based sustainable diet intervention on the adherence to the eatlancet planetary health diet and greenhouse gas emissions of consumers: a quasiexperimental study at a large German hospital |
| Humpenöder 2024 | Unavailable data | Food matters: Dietary shifts increase the feasibility of 1.5°C pathways in line with the Paris Agreement |
| Kibayashi 2024 | Wrong exposures | The Association between the Planetary Health Diet with a Regular Consumption of Breakfast and a Well‐Balanced Diet: A Cross‐Sectional Analysis Involving Japanese Male Engineering Students at a University in Hyogo Prefecture |
| Li 2024 | Wrong exposures | Livestock sector can threaten planetary boundaries without regionally differentiated strategies |
| Meier 2024 | Wrong exposures | Bridging Nutritional and Environmental Sustainability Within Planetary Boundaries in Food Life Cycle Assessments: SWOT Review and Development of the Planet Health Conformity IndexBridging Nutritional and Environmental Sustainability Within Planetary Boundaries in Food Life Cycle Assessments: SWOT Review and Development of the Planet Health Conformity Index |
| Murat-Ringot 2024 | Wrong exposures | An Innovative One Health Approach: BIOQUALIM, a Transdisciplinary Research Action Protocol—From Cultivated Biodiversity to Human Health Prevention |
| Navruz-Varli 2024 | Wrong exposures | Shift Work, Shifted Diets: An Observational Follow-Up Study on Diet Quality and Sustainability among Healthcare Workers on Night Shifts |
| Ojeda-Rodríguez 2024 | Wrong exposures | Higher Adherence to the EAT-Lancet Diets After a Lifestyle Intervention in a Pediatric Population with Abdominal Obesity |
| Pan 2024 | Wrong exposures | Is Nutrient Quality of the Locally-Existing, EAT-*Lancet*-like Plant-Based Diet Better or Worse than the Average Diet in Taiwan? An Example of Local Translation |
| Reese 2024 | Unavailable data | “Climate-friendly” diets from an allergy point of view |
| Richter 2024 | Wrong exposures | Evaluating the diet in Germany with two indices focusing on healthy eating and planetary healthy eating using nationwide cross‑sectional food intake data from DEGS1 (2008–2011) |
| Rochefort 2024 | Wrong design | Are the 2019 Canada’s Food Guide Recommendations on Healthy Food Choices Consistent with the EAT-Lancet Reference Diet from Sustainable Food Systems? |
| Sardo 2024 | Wrong exposures | Optimized crop distributions in Egypt increase crop productivity and nutritional standards, reducing the irrigation water requirement |
| Skeaﬀ 2024 | Wrong exposures | Alignment of dietary patterns with EAT-Lancet recommendations: a cross-sectional study of plant-based energy and nutrient intakes in New Zealand adolescent |
| Tucci 2024 | Wrong outcomes | The MED_EAT-IT approach: A modelling study to develop feasible, sustainable and nutritionally targeted dietary patterns based on the Planetary health diet |
| Unger 2024 | Unavailable data | Comment on methodological shortcomings of an analysis evaluating the eat-lancet healthy reference diet and type 2 diabetes incidence by Lopez et al. |
| Zhan 2024 | Wrong exposures | Dietaryindex: a user-friendly and versatile R package for standardizing dietary pattern analysis in epidemiological and clinical studies |
| Bashiri 2025 | Wrong exposures | Integrating multi-criteria decision-making with multi-objective optimization for sustainable diet design |
| de Amicis 2025 | Wrong exposures | How sustainable are hypocaloric and balanced diets for weight loss? |
| de Carli 2025 | Wrong exposures | EAT-Lancet Diet Components Acquisition According to Food Insecurity and Poverty Status in Brazil: An Analysis of National Household Budget Survey 2017–2018 |
| Hanslian 2025 | Wrong exposures | Effects of nutritional counseling on dietary patterns in patients with mild cognitive impairment: insights from the BrainFit-Nutrition study |
| Hargous 2025 | Wrong design | Changes in Children’s Adherence to Sustainable Healthy Diets During the Implementation of Chile’s Food Labelling and Advertising Law: A Longitudinal Study (2016–2019) |
| Hwalla 2025 | Wrong exposures | Developing sustainable food-based dietary guidelines for Lebanon: integrating health, economic resilience, and sustainability |
| Kaimila 2025 | Wrong exposures | Pulse and legume consumption is associated with a more optimal nutrient intake and a higher EAT‑Lancet index in a representative UK population |
| Kersting 2025 | Wrong exposures | Replacing cow’s milk with plant-based drinks: consequences for nutrient intake of young children on a balanced diet in Germany |
| Kytta 2025 | Wrong exposures | Integrating nutrition into environmental impact assessments reveals limited sustainable food options within planetary boundaries☆ |
| Orkusz 2025 | Wrong exposures | Children’s Diets and Planetary Health: A Study in Wroclaw, Poland, and Sydney, Australia |
| Rochefort 2025 | Wrong design | Are dietary changes over eight years in the prospective NutriNet-Sante cohort consistent with the EAT-Lancet reference diet? |

**Appendix 5** **The Newcastle-Ottawa Scale quality assessment table for meta-analysis**

**Table S9. Risk of bias assessment of studies on population health**

| **Study** | **Study type** | **Selection** | **Comparability** | **Outcome** | **All** | **Quality assessment** |
| --- | --- | --- | --- | --- | --- | --- |
| Knuppel 2019 | Prospective cohort | 3 | 2 | 2 | 7 | High quality |
| Berthy 2022 | Prospective cohort | 3 | 2 | 2 | 7 | High quality |
| Ibsen 2022 | Prospective cohort | 3 | 2 | 3 | 8 | High quality |
| Lazarova 2022 | Prospective cohort | 3 | 2 | 2 | 7 | High quality |
| Vallejo 2022 | Prospective cohort | 2 | 2 | 3 | 7 | High quality |
| Stubbendorff 2022 | Prospective cohort | 4 | 2 | 3 | 9 | High quality |
| Xu 2022 | Prospective cohort | 2 | 2 | 3 | 7 | High quality |
| Colizz 2023 | Prospective cohort | 3 | 2 | 3 | 8 | High quality |
| Karavasiloglou 2023 | Prospective cohort | 2 | 2 | 3 | 8 | High quality |
| Langmann 2023 | Prospective cohort | 3 | 2 | 3 | 8 | High quality |
| Langmann 2023 | Prospective cohort | 3 | 2 | 3 | 8 | High quality |
| López 2023 | Prospective cohort | 2 | 2 | 1 | 5 | Low quality |
| Ren 2023 | Prospective cohort | 3 | 2 | 2 | 7 | High quality |
| Xiao 2023 | Prospective cohort | 3 | 2 | 2 | 7 | High quality |
| Ye 2023 | Prospective cohort | 3 | 2 | 3 | 8 | High quality |
| Zhang 2023 | Prospective cohort | 4 | 2 | 3 | 9 | High quality |
| Zhang 2023 | Prospective cohort | 4 | 2 | 3 | 9 | High quality |
| Zhang 2023 | Prospective cohort | 4 | 2 | 3 | 9 | High quality |
| Bui 2024 | Prospective cohort | 2 | 2 | 3 | 7 | High quality |
| Cai 2024 | Prospective cohort | 3 | 2 | 2 | 7 | High quality |
| Chen 2024 | Prospective cohort | 3 | 2 | 3 | 8 | High quality |
| Gonçalves 2024 | Prospective cohort | 2 | 2 | 3 | 7 | High quality |
| Guzmán-Castellanos 2024 | Prospective cohort | 3 | 2 | 2 | 7 | High quality |
| Klapp 2024 | Prospective cohort | 3 | 2 | 2 | 7 | High quality |
| Li 2024 | Prospective cohort | 3 | 2 | 3 | 8 | High quality |
| Liu 2024 | Prospective cohort | 3 | 2 | 3 | 8 | High quality |
| Lu 2024 | Prospective cohort | 3 | 2 | 3 | 8 | High quality |
| Morcel 2024 | Prospective cohort | 2 | 2 | 1 | 5 | Low quality |
| Pitt 2024 | Prospective cohort | 3 | 2 | 3 | 8 | High quality |
| Quartiroli 2024 | Prospective cohort | 2 | 2 | 3 | 7 | High quality |
| Ren 2024 | Prospective cohort | 3 | 2 | 3 | 8 | High quality |
| Sawicki 2024 | Prospective cohort | 2 | 2 | 3 | 7 | High quality |
| Sotos-Prieto 2024 | Prospective cohort | 3 | 2 | 3 | 8 | High quality |
| Teixeira 2024 | Prospective cohort | 3 | 2 | 2 | 7 | High quality |
| van Soest 2024 | Prospective cohort | 2 | 2 | 3 | 7 | High quality |
| Wu 2024 | Prospective cohort | 3 | 2 | 3 | 8 | High quality |
| Ye 2024 | Prospective cohort | 3 | 2 | 3 | 8 | High quality |
| Zhang 2024 | Prospective cohort | 3 | 2 | 3 | 8 | High quality |
| Zhang 2024 | Prospective cohort | 4 | 2 | 3 | 9 | High quality |
| Zhang 2024 | Prospective cohort | 3 | 2 | 3 | 8 | High quality |
| Zhao 2024 | Prospective cohort | 3 | 2 | 3 | 8 | High quality |
| Berthy 2025 | Prospective cohort | 3 | 2 | 3 | 8 | High quality |
| Chen 2025 | Prospective cohort | 3 | 2 | 3 | 8 | High quality |
| Han 2025 | Prospective cohort | 3 | 2 | 3 | 8 | High quality |
| Hu 2025 | Prospective cohort | 3 | 2 | 3 | 8 | High quality |
| Karavasiloglou 2025 | Prospective cohort | 3 | 2 | 3 | 8 | High quality |
| Martins 2025 | Prospective cohort | 2 | 2 | 3 | 7 | High quality |
| Nair 2025 | Prospective cohort | 2 | 2 | 3 | 7 | High quality |
| Samuelsson 2025 | Prospective cohort | 2 | 2 | 3 | 7 | High quality |
| Shan 2025 | Prospective cohort | 3 | 2 | 2 | 7 | High quality |
| Tang 2025 | Prospective cohort | 3 | 2 | 2 | 7 | High quality |
| Watanabe 2025 | Prospective cohort | 2 | 2 | 3 | 7 | High quality |
| Wu 2025 | Prospective cohort | 3 | 2 | 2 | 7 | High quality |
| Zhan 2025 | Prospective cohort | 3 | 2 | 3 | 8 | High quality |
| Cacau 2021 | Cross-sectional | 3 | 2 | 2 | 7 | High quality |
| Cacau 2022 | Cross-sectional | 3 | 2 | 2 | 7 | High quality |
| Marchioni 2022 | Cross-sectional | 3 | 2 | 2 | 7 | High quality |
| Cacau 2023 | Cross-sectional | 3 | 2 | 1 | 6 | High quality |
| Quarpong 2023 | Cross-sectional | 2 | 2 | 2 | 6 | High quality |
| de Oliveira Neta 2024 | Cross-sectional | 2 | 2 | 1 | 5 | Low quality |
| Frank 2024 | Cross-sectional | 3 | 2 | 2 | 7 | High quality |
| Huang 2024 | Cross-sectional | 2 | 2 | 1 | 5 | Low quality |
| Kamrani 2024 | Cross-sectional | 2 | 2 | 2 | 6 | Low quality |
| Masip 2024 | Cross-sectional | 2 | 2 | 2 | 6 | Low quality |
| Rodrigues 2024 | Cross-sectional | 1 | 2 | 2 | 5 | Low quality |
| Shojaei 2024 | Cross-sectional | 2 | 2 | 2 | 6 | Low quality |
| Teixeira 2024 | Cross-sectional | 2 | 2 | 2 | 6 | Low quality |
| van Soest 2024 | Cross-sectional | 1 | 2 | 2 | 5 | Low quality |
| Dehnavi 2025 | Cross-sectional | 2 | 2 | 2 | 6 | Low quality |
| Jiang 2025 | Cross-sectional | 3 | 2 | 2 | 7 | High quality |
| Lan 2025 | Cross-sectional | 3 | 2 | 2 | 7 | High quality |
| Nair 2025 | Cross-sectional | 2 | 2 | 2 | 6 | Low quality |
| Tabatabaei 2025 | Cross-sectional | 3 | 2 | 2 | 7 | High quality |
| Tan 2025 | Cross-sectional | 2 | 2 | 2 | 6 | Low quality |

**Table S10. Risk of bias assessment of studies on environmental sustainability**

| **Study** | **Study type** | **Selection** | **Comparability** | **Outcome** | **All** | **Quality assessment** |
| --- | --- | --- | --- | --- | --- | --- |
| Cacau 2021 | Cross-sectional | 3 | 2 | 2 | 7 | High quality |
| Kesse-Guyot 2021 | Cross-sectional | 3 | 2 | 2 | 7 | High quality |
| Laine 2021 | Cross-sectional | 3 | 1 | 2 | 6 | Low quality |
| Tepper 2022 | Cross-sectional | 2 | 1 | 1 | 4 | Low quality |
| Vallejo 2022 | Cross-sectional | 2 | 2 | 2 | 6 | Low quality |
| Colizz 2023 | Cross-sectional | 3 | 2 | 2 | 7 | High quality |
| Mangone 2023 | Cross-sectional | 3 | 2 | 2 | 7 | High quality |
| Ye 2023 | Cross-sectional | 3 | 2 | 2 | 7 | High quality |
| Bui 2024 | Cross-sectional | 2 | 2 | 3 | 7 | High quality |
| Chen 2024 | Cross-sectional | 3 | 2 | 2 | 7 | High quality |
| Frank 2024 | Cross-sectional | 3 | 2 | 2 | 7 | High quality |
| Shojaei2024 | Cross-sectional | 2 | 2 | 2 | 6 | Low quality |
| Teixeira 2024 | Cross-sectional | 2 | 2 | 2 | 6 | Low quality |
| Jovanovic2025 | Cross-sectional | 2 | 2 | 2 | 6 | Low quality |
| Wu2025 | Cross-sectional | 3 | 2 | 2 | 7 | High quality |
| Zhan2025 | Cross-sectional | 3 | 2 | 2 | 7 | High quality |

**Appendix 6 GRADE assessment**

**Table S11. GRADE assessment of different population health outcomes**

| **Outcome** | **Certainty assessment** | | | | | | | | **Effect** | | **Certainty** |
| --- | --- | --- | --- | --- | --- | --- | --- | --- | --- | --- | --- |
|  | **№ of studies** | **№ of Comparisons** | **Study design** | **Risk of bias** | **Inconsistency** | **Indirectness** | **Imprecision** | **Other considerations** | **Relative** | **Absolute** |  |
|  |  |  |  |  |  |  |  |  | **(95% CI)** | **(95% CI)** |  |
| All-cause mortality | 17 | 18 | Prospective cohorts | not serious | serious^a^ | not serious | not serious | none | **HR 0.80** |  | ⨁◯◯◯ |
|  |  |  |  |  |  |  |  |  | (0.76 to 0.85) |  | Very low |
| Cancer mortality | 7 | 8 | Prospective cohorts | not serious | not serious | not serious | not serious | none | **HR 0.90** |  | ⨁⨁◯◯ |
|  |  |  |  |  |  |  |  |  | (0.86 to 0.95) |  | Low |
| Cardiovascular mortality | 7 | 8 | Prospective cohorts | not serious | not serious | not serious | not serious | none | **HR 0.83** |  | ⨁⨁◯◯ |
|  |  |  |  |  |  |  |  |  | (0.76 to 0.89) |  | Low |
| Lung cancer | 3 | 3 | Prospective cohorts | not serious | not serious | not serious | not serious | none | **HR 0.69** | **-** | ⨁⨁◯◯ |
|  |  |  |  |  |  |  |  |  | (0.56 to 0.84) |  | Low |
| Breast cancer | 3 | 3 | Prospective cohorts | not serious | not serious | not serious | not serious | none | **HR 0.96** |  | ⨁⨁◯◯ |
|  |  |  |  |  |  |  |  |  | (0.81 to 1.14) |  | Low |
| Colorectal cancer | 4 | 4 | Prospective cohorts | not serious | not serious | not serious | not serious | none | **HR 0.87** |  | ⨁⨁◯◯ |
|  |  |  |  |  |  |  |  |  | (0.81 to 0.93) |  | Low |
| Cardiovascular disease/events | 10 | 11 | Prospective cohorts | not serious | serious^a^ | not serious | not serious | none | **HR 0.83** | **-** | ⨁◯◯◯ |
|  |  |  |  |  |  |  |  |  | (0.74 to 0.94) |  | Very low |
| Coronary artery disease | 4 | 4 | Prospective cohorts | not serious | not serious | not serious | not serious | none | **HR 0.83** | **-** | ⨁⨁◯◯ |
|  |  |  |  |  |  |  |  |  | (0.77 to 0.89) |  | Low |
| Myocardial infarction | 4 | 4 | Prospective cohorts | not serious | serious^a^ | not serious | not serious | none | **HR 0.54** | **-** | ⨁◯◯◯ |
|  |  |  |  |  |  |  |  |  | (0.03 to 8.57) |  | Very low |
| Heart failure | 2 | 3 | Prospective cohorts | not serious | not serious | not serious | not serious | none | **HR 0.74** |  | ⨁⨁◯◯ |
|  |  |  |  |  |  |  |  |  | (0.58 to 0.95) |  | Low |
| Stroke | 8 | 8 | Prospective cohorts | not serious | not serious | not serious | not serious | none | **HR 0.90** | **-** | ⨁⨁◯◯ |
|  |  |  |  |  |  |  |  |  | (0.84 to 0.97) |  | Low |
| Hemorrhagic stroke | 3 | 4 | Prospective cohorts | not serious | not serious | not serious | not serious | none | **HR 0.86** | **-** | ⨁⨁◯◯ |
|  |  |  |  |  |  |  |  |  | (0.38 to 1.93) |  | Low |
| Ischemic stroke | 4 | 4 | Prospective cohorts | not serious | not serious | not serious | not serious | none | **HR 0.93** | **-** | ⨁⨁◯◯ |
|  |  |  |  |  |  |  |  |  | (0.80 to 1.07) |  | Low |
| Type 2 diabetes | 8 | 8 | Prospective cohorts | not serious | serious^a^ | not serious | not serious | publication bias strongly suspected^c^ | **HR 0.75** | **-** | ⨁◯◯◯ |
|  |  |  |  |  |  |  |  |  | (0.57 to 0.98) |  | Very low |
| Systolic blood pressure | 3 | 3 | Cross-sectional studies | not serious | not serious | not serious | serious^b^ | none | **-** | **β –0.05 (–0.18, 0.07)** | ⨁◯◯◯ |
|  |  |  |  |  |  |  |  |  |  | mm Hg | Very low |
| Diastolic blood pressure | 3 | 3 | Cross-sectional studies | not serious | not serious | not serious | serious^b^ | none | **-** | **β –0.02 (–0.07, 0.03)** | ⨁◯◯◯ |
|  |  |  |  |  |  |  |  |  |  | mm Hg | Very low |
| HDL-c | 3 | 3 | Prospective cohorts | not serious | serious^a^ | not serious | serious^b^ | none | **-** | **β 0.55 (–0.50, 1.60)** | ⨁◯◯◯ |
|  |  |  |  |  |  |  |  |  |  | mmol/L | Very low |
| Triglycerides | 4 | 4 | Cross-sectional studies | not serious | not serious | not serious | serious^b^ | none | **-** | **β –0.14 (–0.64, 0.36)**  mmol/L | ⨁◯◯◯ |
|  |  |  |  |  |  |  |  |  |  |  | Very low |
| LDL-c | 3 | 3 | Cross-sectional studies | not serious | serious^a^ | not serious | serious^b^ | publication bias strongly suspected^c^ | **-** | **β –0.16 (–0.66, 0.34)** | ⨁◯◯◯ |
|  |  |  |  |  |  |  |  |  |  | mmol/L | Very low |
| HDL-c | 4 | 4 | Cross-sectional studies | not serious | not serious | not serious | serious^b^ | none | **-** | **β 0.07 (–0.13, 0.27)**  mmol/L | ⨁◯◯◯ |
|  |  |  |  |  |  |  |  |  |  |  | Very low |
| Overweight/Obesity | 4 | 6 | Cross-sectional studies | not serious | serious^a^ | not serious | not serious | publication bias strongly suspected^c^ | **OR 0.87** | **-** | ⨁◯◯◯ |
|  |  |  |  |  |  |  |  |  | (0.46 to 0.99) |  | Very low |
| BMI | 4 | 4 | Cross-sectional studies | not serious | serious^a^ | not serious | serious^b^ | none | - | **β –0.06 (–0.26, 0.14)** | ⨁◯◯◯ |
|  |  |  |  |  |  |  |  |  |  | kg/m^2^ | Very low |
| Waist circumference | 4 | 6 | Cross-sectional studies | not serious | serious^a^ | not serious | serious^b^ | none | **-** | **β –0.10 (–0.27, 0.06)** | ⨁◯◯◯ |
|  |  |  |  |  |  |  |  |  |  | cm | Very low |
| Metabolic dysfunction-associated steatotic liver disease (MASLD) | 2 | 4 | Prospective cohorts | not serious | not serious | not serious | not serious | none | **HR 0.80** | **-** | ⨁⨁◯◯ |
|  |  |  |  |  |  |  |  |  | (0.71 to 0.92) |  | Low |
| Depression | 5 | 5 | Cross-sectional studies | not serious | not serious | not serious | not serious | none | **OR 0.73** | **-** | ⨁⨁◯◯ |
|  |  |  |  |  |  |  |  |  | (0.65 to 0.81) |  | Low |

Abbreviations: CI: confidence interval; HR: hazard ratio; OR: odds ratio

* a. Rated down for inconsistency (I^2^≥50% and P<0.1)

b. Rated down for imprecision (95% CIs overlap no effect (i.e., CI includes RR of 1.0)=1 unless large populations (simple size≥2000) and very low incidence; and the same logic for continuous outcomes (i.e., effect estimates overlapped minimally important differences, MID)

c. Rated down for publication bias (Pegger<0.05)

**Appendix 7 Forest plots of the EAT-Lancet planetary health diet and population health and environment outcomes**

**Figure S1-1. Random effects meta-analysis of prospective cohort studies on the EAT-Lancet diet and mortality.** Squares denote hazard ratios [HRs] for individual studies weighted by sample size, diamonds represent pooled HRs with 95% confidence intervals, and the horizontal lines indicate the 95% confidence intervals for individual studies.

**Figure S1-2. Random effects meta-analysis of prospective cohort studies on the EAT-Lancet diet and cancer.** Squares denote hazard ratios [HRs] for individual studies weighted by sample size, diamonds represent pooled HRs with 95% confidence intervals, and the horizontal lines indicate the 95% confidence intervals for individual studies.

**Figure S1-3. Random effects meta-analysis of prospective cohort studies on the EAT-Lancet diet and cardiovascular disease****.** Squares denote hazard ratios [HRs] for individual studies weighted by sample size, diamonds represent pooled HRs with 95% confidence intervals, and the horizontal lines indicate the 95% confidence intervals for individual studies).

**Figure S1-4. Random effects meta-analysis of prospective cohort studies on the EAT-Lancet diet and cerebrovascular disease.** Squares denote hazard ratios [HRs] for individual studies weighted by sample size, diamonds represent pooled HRs with 95% confidence intervals, and the horizontal lines indicate the 95% confidence intervals for individual studies.

**Figure S1-5. Random effects meta-analysis of prospective cohort studies on the EAT-Lancet diet and glucose related outcomes.** Squares denote hazard ratios [HRs] for individual studies weighted by sample size, diamonds represent pooled HRs with 95% confidence intervals, and the horizontal lines indicate the 95% confidence intervals for individual studies.

**Figure S1-6. Random effects meta-analysis of cross-sectional studies on the EAT-Lancet diet and glucose related outcomes.** Square denotes odds ratios [ORs] for individual study outcomes weighted by sample size, and the horizontal line indicates the 95% confidence intervals for the study.

**Figure S1-7. Random effects meta-analysis of cross-sectional studies on the EAT-Lancet diet and glucose related outcomes****.** Square denotes regression coefficients [β] for individual studies weighted by sample size, diamonds represent pooled β with 95% confidence intervals, and the horizontal lines indicate the 95% confidence intervals for individual studies.

**Figure S1-8. Random effects meta-analysis of cross-sectional studies on the EAT-Lancet diet and blood pressure related outcomes studies.** Square denotes odds ratios [ORs] for individual study outcomes weighted by sample size, and the horizontal line indicates the 95% confidence intervals for the study.

**Figure S1-9a. Random effects meta-analysis of prospective cohort studies on the EAT-Lancet diet and blood pressure related outcomes****.** Squares denote regression coefficients [β] for individual studies weighted by sample size, diamonds represent pooled β with 95% confidence intervals, and the horizontal lines indicate the 95% confidence intervals for individual studies.

**Figure S1-9b. Random effects meta-analysis of cross-sectional studies on the EAT-Lancet diet and blood pressure related outcomes.** Squares denote regression coefficients [β] for individual studies weighted by sample size, and diamonds indicate pooled β with 95% confidence intervals.

**Figure S1-10a. Random effects meta-analysis of prospective cohort studies on the EAT-Lancet diet and blood lipid related outcomes.** Squares denote regression coefficients [β] for individual studies weighted by sample size, diamonds represent pooled β with 95% confidence intervals, and the horizontal lines indicate the 95% confidence intervals for individual studies.

**Figure S1-10b. Random effects meta-analysis of cross-sectional studies on the EAT-Lancet diet and blood lipid related outcomes.** Squares denote regression coefficients [β] for individual studies weighted by sample size, diamonds represent pooled β with 95% confidence intervals, and the horizontal lines indicate the 95% confidence intervals for individual studies.

**Figure S1-11. Random effects meta-analysis of prospective cohort studies on the EAT-Lancet diet and body weight related outcomes.** Squares denote hazard ratios [HRs] for individual studies weighted by sample size, diamonds represent pooled HRs with 95% confidence intervals, and the horizontal lines indicate the 95% confidence intervals for individual studies.

**Figure S1-12. Random effects meta-analysis of cross-sectional studies on the EAT-Lancet diet and body weight related outcomes.** Squares denote odds ratios [ORs] for individual study outcomes weighted by sample size, diamonds represent pooled ORs with 95% confidence intervals, and the horizontal lines indicate the 95% confidence intervals for individual studies.

**Figure S1-13. Random effects meta-analysis of prospective cohort studies on the EAT-Lancet diet and body weight related outcomes.** Square denotes prevalence ratio [PR] with 95% confidence interval, diamonds represent pooled PR with 95% confidence intervals, and the horizontal lines indicate the 95% confidence interval for individual studies.

**Figure S1-14a. Random effects meta-analysis of prospective cohort studies on the EAT-Lancet diet and body weight related outcomes.** Squares denote regression coefficients [β] for individual studies weighted by sample size, diamonds represent pooled β with 95% confidence intervals, and the horizontal lines indicate the 95% confidence intervals for individual studies.

**Figure S1-14b. Random effects meta-analysis of cross-sectional studies on the EAT-Lancet diet and body weight related outcomes.** Squares denote regression coefficients [β] for individual studies weighted by sample size, diamonds represent pooled β with 95% confidence intervals, and the horizontal lines indicate the 95% confidence intervals for individual studies.

**Figure S1-15. Random effects meta-analysis of prospective cohort studies on the EAT-Lancet diet and liver disease.** Squares denote hazard ratios [HRs] for individual studies weighted by sample size, diamonds represent pooled HRs with 95% confidence intervals, and the horizontal lines indicate the 95% confidence intervals for individual studies.

**Figure S1-16. Random effects meta-analysis of cross-sectional studies on the EAT-Lancet diet and immune system disease.** Squares denote odds ratios [ORs] for individual study outcomes weighted by sample size, diamonds represent pooled ORs with 95% confidence intervals, and the horizontal lines indicate the 95% confidence intervals for individual studies.

**Figure S1-17. Random effects meta-analysis of prospective cohort studies on the EAT-Lancet diet and mental disorders.** Squares denote hazard ratios [HRs] for individual studies weighted by sample size, diamonds represent pooled HRs with 95% confidence intervals, and the horizontal lines indicate the 95% confidence intervals for individual studies.

**Figure S1-18. Random effects meta-analysis of cross-sectional studies on the EAT-Lancet diet and mental disorders.** Square denotes odds ratios [ORs] for individual study outcomes weighted by sample size, and the horizontal line indicates the 95% confidence intervals for the study.

**Figure S1-19. Random effects meta-analysis of prospective cohort studies on the EAT-Lancet diet and mental disorders.** Squares denote regression coefficients [β] for individual studies weighted by sample size, diamonds represent pooled β with 95% confidence intervals, and the horizontal lines indicate the 95% confidence intervals for individual studies.

**Figure S1-20. Random effects meta-analysis of cross-sectional studies on the EAT-Lancet diet and greenhouse gas emissions.** Squares denote mean differences [mdiff] with 95% confidence intervals, diamond represents pooled mdiff with 95% confidence intervals, and the horizontal lines indicate the 95% confidence intervals for individual studies.

**Figure S1-21. Random effects meta-analysis of cross-sectional studies on the EAT-Lancet diet and greenhouse gas emissions.** Squares denote regression coefficients [β] for individual studies weighted by sample size, diamond represents pooled β with 95% confidence intervals, and the horizontal lines indicate the 95% confidence intervals for individual studies.

**Figure S1-22. Random effects meta-analysis of cross-sectional studies on the EAT-Lancet diet and land use.** Squares denote mean differences [mdiff] with 95% confidence intervals, diamond represents pooled mdiff with 95% confidence intervals, and the horizontal lines indicate the 95% confidence intervals for individual studies.

**Figure S1-23. Random effects meta-analysis of cross-sectional studies on the EAT-Lancet diet and land use.** Squares denote regression coefficients [β] for individual studies weighted by sample size, diamond represents pooled β with 95% confidence intervals, and the horizontal lines indicate the 95% confidence intervals for individual studies.

**Figure S1-24. Random effects meta-analysis of cross-sectional studies on the EAT-Lancet diet and water use.** Squares denote mean differences [mdiff] with 95% confidence intervals, diamonds represent pooled mdiff with 95% confidence intervals, and the horizontal lines indicate the 95% confidence intervals for individual studies.

**Figure S1-25. Random effects meta-analysis of cross-sectional studies on the EAT-Lancet diet and water use.**

Squares denote regression coefficients [β] for individual studies weighted by sample size, diamond represents pooled β with 95% confidence intervals, and the horizontal lines indicate the 95% confidence intervals for individual studies.

**Appendix 8 Funnel plots of population health and environment outcomes**

**Figure S2-1. Funnel plot of hazard ratio for all-cause mortality from prospective cohort studies**

**Figure S2-2. Funnel plot of hazard ratio for cancer mortality from prospective cohort studies**

**Figure S2-3. Funnel plot analysis of hazard ratio for cardiovascular mortality from prospective cohort studies**

**Figure S2-4. Funnel plot analysis of hazard ratio for lung cancer from prospective cohort studies**

**Figure S2-5. Funnel plot analysis of hazard ratio for breast cancer from prospective cohort studies**

**Figure S2-6. Funnel plot analysis of hazard ratio for colorectal cancer from prospective cohort studies**

**Figure S2-7. Funnel plot analysis of hazard ratio for cardiovascular disease or events from prospective cohort studies**

**Figure S2-8. Funnel plot analysis of hazard ratio for coronary artery disease from prospective cohort studies**

**Figure S2-9. Funnel plot analysis of hazard ratio for myocardial infarction from prospective cohort studies**

**Figure S2-10. Funnel plot analysis of hazard ratio for heart failure from prospective cohort studies**

**Figure S2-11. Funnel plot analysis of hazard ratio for stroke from prospective cohort studies**

**Figure S2-12. Funnel plot analysis of hazard ratio for hemorrhagic stroke from prospective cohort studies**

**Figure S2-13. Funnel plot analysis of hazard ratio for ischemic stroke from prospective cohort studies**

**Figure S2-14. Funnel plot analysis of hazard ratio for type 2 diabetes from prospective cohort studies**

**Figure S2-15. Funnel plot analysis of regression coefficients (β) for systolic blood pressure from cross-sectional studies**

**Figure S2-16. Funnel plot analysis of regression coefficients (β) for diastolic blood pressure from cross-sectional studies**

**Figure S2-17. Funnel plot analysis of regression coefficients (β) for HDL-C from prospective cohort studies**

**Figure S2-18. Funnel plot analysis of regression coefficients (β) for triglycerides from cross-sectional studies**

**Figure S2-19. Funnel plot analysis of regression coefficients (β) for LDL-C from cross-sectional studies**

**Figure S2-20. Funnel plot analysis of regression coefficients (β) for HDL-C from cross-sectional studies**

**Figure S2-21. Funnel plot analysis of odds ratio for overweight or obesity from cross-sectional studies**

**Figure S2-22. Funnel plot analysis of regression coefficients (β) for BMI from cross-sectional studies**

**Figure S2-23. Funnel plot analysis of regression coefficients (β) for waist circumference from cross-sectional studies**

**Figure S2-24. Funnel plot analysis of hazard ratio for metabolic dysfunction-associated steatotic liver disease (MASLD) from prospective cohort studies**

**Figure S2-25. Funnel plot analysis of odds ratio for depression from cross-sectional studies**

**Figure S2-26. Funnel plot analysis of mean differences (mdiff) for greenhouse gas emissions from cross-sectional studies**

**Figure S2-27. Funnel plot analysis of regression coefficients (β) for greenhouse gas emissions from cross-sectional studies**

**Figure S2-28. Funnel plot analysis of mean differences (mdiff) for land use from cross-sectional studies**

**Figure S2-29. Funnel plot analysis of regression coefficients (β) for land use from cross-sectional studies**

**Figure S2-30. Funnel plot analysis of mean differences (mdiff) for water footprint from cross-sectional studies**

**Figure S2-31. Funnel plot analysis of mean differences (mdiff) for blue water use from cross-sectional studies**

**Figure S2-32. Funnel plot analysis of regression coefficients (β) for total water footprint from cross-sectional studies**

**Appendix 9 Sensitivity analysis**

**Figure S3-1. ‘Leave-one-out’ sensitivity analysis of hazard ratio for all-cause mortality from prospective cohort studies**

**Figure S3-2. ‘Leave-one-out’ sensitivity analysis of hazard ratio for cancer mortality from prospective cohort studies**

**Figure S3-3. ‘Leave-one-out’ sensitivity analysis of hazard ratio for cardiovascular mortality from prospective cohort studies**

**Figure S3-4. ‘Leave-one-out’ sensitivity analysis of hazard ratio for lung cancer from prospective cohort studies**

**Figure S3-5. ‘Leave-one-out’ sensitivity analysis of hazard ratio for breast cancer from prospective cohort studies**

**Figure S3-6. ‘Leave-one-out’ sensitivity analysis of hazard ratio for colorectal cancer from prospective cohort studies**

**Figure S3-7. ‘Leave-one-out’ sensitivity analysis of hazard ratio for cardiovascular disease or events from prospective cohort studies**

**Figure S3-8. ‘Leave-one-out’ sensitivity analysis of hazard ratio for coronary artery disease from prospective cohort studies**

**Figure S3-9. ‘Leave-one-out’ sensitivity analysis of hazard ratio for myocardial infarction from prospective cohort studies**

**Figure S3-10. ‘Leave-one-out’sensitivity analysis of hazard ratio for heart failure from prospective cohort studies**

**Figure S3-11. ‘Leave-one-out’ sensitivity analysis of hazard ratio for stroke from prospective cohort studies**

**Figure S3-12. ‘Leave-one-out’ sensitivity analysis of hazard ratio for hemorrhagic stroke from prospective cohort studies**

**Figure S3-13. ‘Leave-one-out’ sensitivity analysis of hazard ratio for ischemic stroke from prospective cohort studies**

**Figure S3-14. ‘Leave-one-out’ sensitivity analysis of hazard ratio for type 2 diabetes from prospective cohort studies**

**Figure S3-15. ‘Leave-one-out’ sensitivity analysis of regression coefficients (β) for systolic blood pressure from cross-sectional studies**

**Figure S3-16. ‘Leave-one-out’ sensitivity analysis of regression coefficients (β) for diastolic blood pressure from cross-sectional studies**

**Figure S3-17. ‘Leave-one-out’ sensitivity analysis of regression coefficients (β) for HDL-C from prospective cohort studies**

**Figure S3-18. ‘Leave-one-out’ sensitivity analysis of regression coefficients (β) for triglycerides from cross-sectional studies**

**Figure S3-19. ‘Leave-one-out’ sensitivity analysis of regression coefficients (β) for LDL-C from cross-sectional studies**

**Figure S3-20. ‘Leave-one-out’ sensitivity analysis of regression coefficients (β) for HDL-C from cross-sectional studies**

**Figure S3-21. ‘Leave-one-out’ sensitivity analysis of odds ratio for overweight or obesity from cross-sectional studies**

**Figure S3-22. ‘Leave-one-out’ sensitivity analysis of odds ratio for BMI from cross-sectional studies**

**Figure S3-23. ‘Leave-one-out’ sensitivity analysis of odds ratio for waist circumference from cross-sectional studies**

**Figure S3-24. ‘Leave-one-out’ sensitivity analysis of hazard ratio for metabolic dysfunction-associated steatotic liver disease (MASLD) from prospective cohort studies**

**Figure S3-25. ‘Leave-one-out’ sensitivity analysis of odds ratio for depression from cross-sectional studies**

**Figure S3-26. ‘Leave-one-out’ sensitivity analysis of mean differences (mdiff) for greenhouse gas emissions from cross-sectional studies**

**Figure S3-27. ‘Leave-one-out’ sensitivity analysis of regression coefficients (β) for greenhouse gas emissions**

**from cross-sectional studies**

**Figure S3-28. ‘Leave-one-out’ sensitivity analysis of mean differences (mdiff) for land use from cross-sectional studies**

**Figure S3-29. ‘Leave-one-out’ sensitivity analysis of regression coefficients (β) for land use from cross-sectional studies**

**Figure S3-30. ‘Leave-one-out’ sensitivity analysis of mean differences (mdiff) for water footprint from cross-sectional studies**

**Figure S3-31. ‘Leave-one-out’ sensitivity analysis of mean differences (mdiff) for blue water use from cross-sectional studies**

**Figure S3-32. ‘Leave-one-out’ sensitivity analysis of regression coefficients (β) for total water footprin from cross-sectional studies**

**References**

[1] Knuppel A, Papier K, Key TJ, Travis RC. EAT-Lancet score and major health outcomes: the EPIC-Oxford study. Lancet. 2019;394:213-4.

[2] Cacau LT, Benseñor IM, Goulart AC, Cardoso LO, Lotufo PA, Moreno LA, et al. Adherence to the Planetary Health Diet Index and Obesity Indicators in the Brazilian Longitudinal Study of Adult Health (ELSA-Brasil). Nutrients. 2021;13.

[3] Berthy F, Brunin J, Allès B, Fezeu LK, Touvier M, Hercberg S, et al. Association between adherence to the EAT-Lancet diet and risk of cancer and cardiovascular outcomes in the prospective NutriNet-Santé cohort. The American journal of clinical nutrition. 2022;116:980-91.

[4] Cacau LT, Benseñor IM, Goulart AC, Cardoso LO, Santos IS, Lotufo PA, et al. Adherence to the EAT-Lancet sustainable reference diet and cardiometabolic risk profile: cross-sectional results from the ELSA-Brasil cohort study. European journal of nutrition. 2023;62:807-17.

[5] Ibsen DB, Christiansen AH, Olsen A, Tjønneland A, Overvad K, Wolk A, et al. Adherence to the EAT-Lancet Diet and Risk of Stroke and Stroke Subtypes: A Cohort Study. Stroke. 2022;53:154-63.

[6] Lazarova SV, Sutherland JM, Jessri M. Adherence to emerging plant-based dietary patterns and its association with cardiovascular disease risk in a nationally representative sample of Canadian adults. The American journal of clinical nutrition. 2022;116:57-73.

[7] Marchioni DM, Cacau LT, De Carli E, Carvalho AM, Rulli MC. Low Adherence to the EAT-Lancet Sustainable Reference Diet in the Brazilian Population: Findings from the National Dietary Survey 2017-2018. Nutrients. 2022;14.

[8] Stubbendorff A, Sonestedt E, Ramne S, Drake I, Hallström E, Ericson U. Development of an EAT-Lancet index and its relation to mortality in a Swedish population. The American journal of clinical nutrition. 2022;115:705-16.

[9] Montejano Vallejo R, Schulz CA, van de Locht K, Oluwagbemigun K, Alexy U, Nöthlings U. Associations of Adherence to a Dietary Index Based on the EAT-Lancet Reference Diet with Nutritional, Anthropometric, and Ecological Sustainability Parameters: Results from the German DONALD Cohort Study. The Journal of nutrition. 2022;152:1763-72.

[10] Xu C, Cao Z, Yang H, Hou Y, Wang X, Wang Y. Association Between the EAT-Lancet Diet Pattern and Risk of Type 2 Diabetes: A Prospective Cohort Study. Frontiers in nutrition. 2021;8:784018.

[11] Cacau LT, Hanley-Cook GT, Vandevijvere S, Leclercq C, De Henauw S, Santaliestra-Pasias A, et al. Association between adherence to the EAT-Lancet sustainable reference diet and cardiovascular health among European adolescents: the HELENA study. European journal of clinical nutrition. 2024;78:202-8.

[12] Colizzi C, Harbers MC, Vellinga RE, Verschuren WMM, Boer JMA, Biesbroek S, et al. Adherence to the EAT-Lancet Healthy Reference Diet in Relation to Risk of Cardiovascular Events and Environmental Impact: Results From the EPIC-NL Cohort. Journal of the American Heart Association. 2023;12:e026318.

[13] Karavasiloglou N, Thompson AS, Pestoni G, Knuppel A, Papier K, Cassidy A, et al. Adherence to the EAT-Lancet reference diet is associated with a reduced risk of incident cancer and all-cause mortality in UK adults. One earth (Cambridge, Mass). 2023;6:1726-34.

[14] Langmann F, Ibsen DB, Tjønneland A, Olsen A, Overvad K, Dahm CC. Adherence to the EAT-Lancet diet is associated with a lower risk of type 2 diabetes: the Danish Diet, Cancer and Health cohort. European journal of nutrition. 2023;62:1493-502.

[15] Langmann F, Ibsen DB, Tjønneland A, Olsen A, Overvad K, Dahm CC. Adherence to the EAT-Lancet diet in midlife and development in weight or waist circumference after five years in a Danish cohort. Dialogues in health. 2023;3:100151.

[16] López GE, Batis C, González C, Chávez M, Cortés-Valencia A, López-Ridaura R, et al. EAT-Lancet Healthy Reference Diet score and diabetes incidence in a cohort of Mexican women. European journal of clinical nutrition. 2023;77:348-55.

[17] Quarpong W, Wakoli S, Oiye S, Williams AM. Interpreting alignment to the EAT-Lancet diet using dietary intakes of lactating mothers in rural Western Kenya. Maternal & child nutrition. 2023;19:e13512.

[18] Ren X, Yu C, Peng L, Gu H, Xiao Y, Tang Y, et al. Compliance with the EAT-Lancet diet and risk of colorectal cancer: a prospective cohort study in 98,415 American adults. Frontiers in nutrition. 2023;10:1264178.

[19] Xiao Y, Peng L, Xu Z, Tang Y, He H, Gu H, et al. Association between adherence to Eat-Lancet diet and incidence and mortality of lung cancer: A prospective cohort study. Cancer science. 2023;114:4433-44.

[20] Ye YX, Geng TT, Zhou YF, He P, Zhang JJ, Liu G, et al. Adherence to a Planetary Health Diet, Environmental Impacts, and Mortality in Chinese Adults. JAMA network open. 2023;6:e2339468.

[21] Zhang S, Dukuzimana J, Stubbendorff A, Ericson U, Borné Y, Sonestedt E. Adherence to the EAT-Lancet diet and risk of coronary events in the Malmö Diet and Cancer cohort study. The American journal of clinical nutrition. 2023;117:903-9.

[22] Zhang S, Stubbendorff A, Ericson U, Wändell P, Niu K, Qi L, et al. The EAT-Lancet diet, genetic susceptibility and risk of atrial fibrillation in a population-based cohort. BMC medicine. 2023;21:280.

[23] Zhang S, Stubbendorff A, Olsson K, Ericson U, Niu K, Qi L, et al. Adherence to the EAT-Lancet diet, genetic susceptibility, and risk of type 2 diabetes in Swedish adults. Metabolism: clinical and experimental. 2023;141:155401.

[24] Bui LP, Pham TT, Wang F, Chai B, Sun Q, Hu FB, et al. Planetary Health Diet Index and risk of total and cause-specific mortality in three prospective cohorts. The American journal of clinical nutrition. 2024;120:80-91.

[25] Cai H, Talsma EF, Chang Z, Wen X, Fan S, Van't Veer P, et al. Health outcomes, environmental impacts, and diet costs of adherence to the EAT-Lancet Diet in China in 1997-2015: a health and nutrition survey. The Lancet Planetary health. 2024;8:e1030-e42.

[26] Chen H, Wang X, Ji JS, Huang L, Qi Y, Wu Y, et al. Plant-based and planetary-health diets, environmental burden, and risk of mortality: a prospective cohort study of middle-aged and older adults in China. The Lancet Planetary health. 2024;8:e545-e53.

[27] de Oliveira Neta RS, Lima S, Medeiros MFA, Araújo DBM, Bernardi N, de Araújo A, et al. The EAT-Lancet diet associated cardiovascular health parameters: evidence from a Brazilian study. Nutrition journal. 2024;23:116.

[28] Frank SM, Jaacks LM, Avery CL, Adair LS, Meyer K, Rose D, et al. Dietary quality and cardiometabolic indicators in the USA: A comparison of the Planetary Health Diet Index, Healthy Eating Index-2015, and Dietary Approaches to Stop Hypertension. PloS one. 2024;19:e0296069.

[29] Gomes Gonçalves N, Cacau LT, Ferreira NV, Lotufo PA, Goulart AC, Viana MC, et al. Adherence to the planetary health diet and cognitive decline: findings from the ELSA-Brasil study. Nature aging. 2024;4:1465-76.

[30] Guzmán-Castellanos KB, Zazpe I, Santiago S, Bes-Rastrollo M, Martínez-González M. Planetary Health Diet and Cardiovascular Disease Risk in the Seguimiento Universidad de Navarra (SUN) Cohort. Nutrients. 2024;17.

[31] Huang S, He Q, Wang X, Choi S, Gong H. Associations of the planetary health diet index (PHDI) with asthma: the mediating role of body mass index. BMC public health. 2024;24:2305.

[32] Kamrani F, Kachouei AA, Sobhani SR, Khosravi M. Nourishing the mind: how the EAT-Lancet reference diet (ELD) and MIND diet impact stress, anxiety, and depression. BMC psychiatry. 2024;24:709.

[33] Klapp R, Laxamana JA, Shvetsov YB, Park SY, Kanehara R, Setiawan VW, et al. The EAT-Lancet Diet Index Is Associated with Lower Obesity and Incidence of Type 2 Diabetes in the Multiethnic Cohort. The Journal of nutrition. 2024.

[34] Peng X, Fan Z, Wei J, Liu R, Lou X, Hu J, et al. Fresh-Cooked but Not Cold-Stored Millet Exhibited Remarkable Second Meal Effect Independent of Resistant Starch: A Randomized Crossover Trial. Nutrients. 2024;16.

[35] Liu F, Si C, Chen L, Peng Y, Wang P, Wang X, et al. EAT-Lancet Diet Pattern, Genetic Predisposition, Inflammatory Biomarkers, and Risk of Lung Cancer Incidence and Mortality. Molecular nutrition & food research. 2024;68:e2400448.

[36] Lu X, Wu L, Shao L, Fan Y, Pei Y, Lu X, et al. Adherence to the EAT-Lancet diet and incident depression and anxiety. Nature communications. 2024;15:5599.

[37] Masip G, Nielsen DE. Relationships between the Planetary Health Diet Index, its food groups, and polygenic risk of obesity in the CARTaGENE cohort. Nutrition & metabolism. 2024;21:116.

[38] Morcel J, Béghin L, Michels N, De Ruyter T, Drumez E, Cailliau E, et al. Nutritional and physical fitness parameters in adolescence impact cardiovascular health in adulthood. Clinical nutrition (Edinburgh, Scotland). 2024;43:1857-64.

[39] Pitt S, Kałuża J, Widenfalk A, Åkesson A, Wolk A. Adherence to the EAT-Lancet diet in relation to mortality and exposure to food contaminants in population-based cohorts of Swedish men and women. Environment international. 2024;184:108495.

[40] Quartiroli M, Roncallo C, Pala V, Simeon V, Ricceri F, Venturelli E, et al. Adherence to Diet Quality Indices and Breast Cancer Risk in the Italian ORDET Cohort. Nutrients. 2024;16.

[41] Ren X, Xiao Y, Xiang L, Peng L, Tang Y, He H, et al. Adherence to the EAT-Lancet diet reduces the risk of head and neck cancers in 101,755 American adults: a prospective cohort study. Public health. 2024;234:191-8.

[42] Rodrigues M, Padrão P, Castro Mendes F, Moreira A, Moreira P. The Planetary Health Diet and Its Association with Asthma and Airway Inflammation in School-Aged Children. Nutrients. 2024;16.

[43] Sawicki CM, Ramesh G, Bui L, Nair NK, Hu FB, Rimm EB, et al. Planetary health diet and cardiovascular disease: results from three large prospective cohort studies in the USA. The Lancet Planetary health. 2024;8:e666-e74.

[44] Shojaei S, Dehnavi Z, Irankhah K, Fatemi SF, Sobhani SR. Adherence to the planetary health diet index and metabolic syndrome: cross-sectional results from the PERSIAN cohort study. BMC public health. 2024;24:2988.

[45] Sotos-Prieto M, Ortolá R, Maroto-Rodriguez J, Carballo-Casla A, Kales SN, Rodríguez-Artalejo F. Association between Planetary Health Diet and Cardiovascular Disease: A Prospective Study from the UK Biobank. European journal of preventive cardiology. 2024.

[46] Teixeira B, Afonso C, Severo M, Oliveira A. Are the EAT-Lancet dietary recommendations associated with future cardiometabolic health? - Insights from the Generation XXI cohort from childhood into early adolescence. The American journal of clinical nutrition. 2024.

[47] Teixeira B, Afonso C, Severo M, Carvalho C, Torres D, Lopes C, et al. Exploring dietary patterns and their association with environmental sustainability and body mass index in children and adolescents: Insights from the National Food, Nutrition and Physical Activity Survey 2015-2016. The Science of the total environment. 2024;945:174051.

[48] van Soest APM, van de Rest O, Witkamp RF, de Groot L. The association between adherence to the EAT-Lancet diet and cognitive ageing. Age and ageing. 2024;53:ii39-ii46.

[49] Wu H, Wei J, Wang S, Chen L, Zhang J, Wang N, et al. Dietary pattern modifies the risk of MASLD through metabolomic signature. JHEP reports : innovation in hepatology. 2024;6:101133.

[50] Xia B, Li Y, Hu L, Xie P, Mi N, Lv L, et al. Healthy eating patterns associated with reduced risk of inflammatory bowel disease by lowering low-grade inflammation: evidence from a large prospective cohort study. BMC medicine. 2024;22:589.

[51] Ye YX, Chen JX, Li Y, Lai YW, Lu Q, Xia PF, et al. Adherence to a planetary health diet, genetic susceptibility, and incident cardiovascular disease: a prospective cohort study from the UK Biobank. The American journal of clinical nutrition. 2024;120:648-55.

[52] Zhang S, Marken I, Stubbendorff A, Ericson U, Qi L, Sonestedt E, et al. The EAT-Lancet Diet Index, Plasma Proteins, and Risk of Heart Failure in a Population-Based Cohort. JACC Heart failure. 2024;12:1197-208.

[53] Zhang JJ, Ye YX, Dorajoo R, Khor CC, Chang XL, Yu HC, et al. APOE Genotype Modifies the Association between Midlife Adherence to the Planetary Healthy Diet and Cognitive Function in Later Life among Chinese Adults in Singapore. The Journal of nutrition. 2024;154:252-60.

[54] Zhao W, Chen Q, Zhang Q, Li S, Zhao J, Chen W, et al. Association of adherence to the EAT-Lancet diet with risk of dementia according to social economic status: a prospective cohort in UK Biobank. GeroScience. 2024.

[55] Berthy F, Allès B, Fezeu LK, Lairon D, Pointereau P, Touvier M, et al. Adherence to the EAT-Lancet reference diet and risk of type 2 diabetes: results from the NutriNet-Santé cohort study. International journal of epidemiology. 2025;54.

[56] Chen H, Yang Q, Zheng H, Tan J, Xie J, Xu M, et al. Planetary health diet index and mortality among US cancer survivors: mediating roles of systemic immune-inflammation index and neutrophil-to-lymphocyte ratio. Nutrition journal. 2025;24:28.

[57] Dehnavi MK, Tabaeifard R, Abbasi H, Hajian PN, Motlagh AD, Bellissimo N, et al. Adherence to planetary health diet index in relation to dietary diversity score and anthropometric indices among Iranian older adults. BMC public health. 2025;25:1865.

[58] Han S, Yan C, Zhang Z, Han Y, Wang Q, Cheng S, et al. Examining the link between adherence to the planetary health diet pattern and mortality in the us: a prospective cohort study. European journal of nutrition. 2025;64:79.

[59] Hu FL, Liu JC, Li DR, Xu YL, Liu BQ, Chen X, et al. EAT-Lancet diet pattern, genetic risk, and risk of colorectal cancer: a prospective study from the UK Biobank. The American journal of clinical nutrition. 2025;121:1017-24.

[60] Jiang C, Choi S, Gong H. From planetary health diet (PHD) to mental health: Higher PHD index protects against depression among the U.S. population. Journal of psychiatric research. 2025;183:31-8.

[61] Karavasiloglou N, Thompson AS, Pestoni G, Suter F, Papier K, Cassidy A, et al. Higher adherence to the EAT-Lancet reference diet is inversely associated with mortality in a UK population of cancer survivors. BMC medicine. 2025;23:286.

[62] Lan Y, Chen L, Lin Z, Tang H, Zhang X. Association of planetary health diet index with depression and mortality in the United States. BMC psychiatry. 2025;25:556.

[63] Martins LB, Gamba M, Stubbendorff A, Gasser N, Löbl L, Stern F, et al. Association between the EAT-Lancet Diet, Incidence of Cardiovascular Events, and All-Cause Mortality: Results from a Swiss Cohort. The Journal of nutrition. 2025;155:483-91.

[64] Nair NK, Bui LP, Sawicki CM, Kandula NR, Kanaya AM, Lee KH, et al. Adherence to the EAT-Lancet Planetary Health Diet and Cardiometabolic Risk Markers in the Mediators of Atherosclerosis in South Asians Living in America (MASALA) Study. Current developments in nutrition. 2025;9:107468.

[65] Samuelsson J, Glans I, Stubbendorff A, Ericson U, Palmqvist S, Hansson O, et al. Associations between the EAT-Lancet planetary health diet and incident dementia. The journal of prevention of Alzheimer's disease. 2025;12:100166.

[66] Shan Y, Bertrand KA, Petrick JL, Sheehy S, Palmer JR. Planetary Health Diet Index in relation to mortality in a prospective cohort study of United States Black females. The American journal of clinical nutrition. 2025;121:589-96.

[67] Tabatabaei GA, Mohammadifard N, Haghighatdoost F, Rafiee H, Abbasi M, Najafi F, et al. Adherence to the Eat-Lancet diet and its association with depression and anxiety among Iranian adults: a cross-sectional multicentric study. Frontiers in nutrition. 2025;12:1524652.

[68] Tan JX, Li QZ, Mo YX, Zhou HP, Miao L, Ruan GT, et al. Evaluating and modifying the PHDI for depression prevention: insights from NHANES 2005-2018. Frontiers in nutrition. 2025;12:1601129.

[69] Tang L, Yu X, Qiu C, Lu Y, Wang Y, Liu F, et al. Adherence to the planetary health diet is associated with slower cognitive decline: a prospective cohort analysis of Chinese older adults. The international journal of behavioral nutrition and physical activity. 2025;22:56.

[70] Watanabe D, Yoshida T, Nanri H, Watanabe Y, Goto C, Ishikawa-Takata K, et al. Adherence to a Planetary Health Diet, Diet-related Greenhouse Gas Emissions, Functional Disability, and Mortality in Older Adults. The journals of gerontology Series A, Biological sciences and medical sciences. 2025;80.

[71] Wu M, Lv Y, Liu W, Liu K, Wang Y, Cui Z, et al. Exploring Environmental and Cardiometabolic Impacts Associated with Adherence to the Sustainable EAT-Lancet Reference Diet: Findings from the China Health and Nutrition Survey. Environmental health perspectives. 2025;133:57028.

[72] Zhan J, Bui L, Hodge RA, Zimmer M, Pham T, Rose D, et al. Planetary Health Diet Index Trends and Associations with Dietary Greenhouse Gas Emissions, Disease Biomarkers, Obesity, and Mortality in the United States (2005-2018). The American journal of clinical nutrition. 2025;121:580-8.

[73] Sheng FF, Wang JJ, Chen KZ, Fan SG, Gao HX. Changing Chinese Diets to Achieve a Win-Win Solution for Health and the Environment. China & World Economy. 2021;29:34-52.

[74] Koelman L, Herpich C, Norman K, Jannasch F, Börnhorst C, Schulze MB, et al. Adherence to Healthy and Sustainable Dietary Patterns and Long-Term Chronic Inflammation: Data from the EPIC-Potsdam Cohort. The journal of nutrition, health & aging. 2023;27:1109-17.

[75] Neta RSD, Lima S, de Medeiros MFA, Neta A, Jacob MCM, Marchioni DML, et al. Adherence to the EAT-Lancet Dietary Recommendations for a Healthy and Sustainable Diet-The Case of the Brazuca Natal Study. Sustainability. 2023;15.

[76] Suikki T, Maukonen M, Kaartinen NE, Harald K, Bäck S, Sares-Jäske L, et al. Associations of EAT-Lancet Planetary Health Diet or Finnish Nutrition Recommendations with changes in obesity measures: a follow-up study in adults. Food & nutrition research. 2023;67.

[77] Zhang W, He Y, Wang C, Chen F, Jiang B, Li W. Adherence to Healthy Dietary Patterns and Glioma: A Matched Case-Control Study. Nutrients. 2023;15.

[78] Ataei Kachouei A, Mohammadifard N, Haghighatdoost F, Hajihashemi P, Zarepur E, Nouhi F, et al. Adherence to EAT-Lancet reference diet and risk of premature coronary artery diseases: a multi-center case-control study. European journal of nutrition. 2024;63:2933-42.

[79] Chang Z, Biesbroek S, Cai H, Fan S, Ni Y, Wen X, et al. Heterogeneity in diet-related non-communicable disease risks in a Chinese population. European journal of nutrition. 2024;63:2975-86.

[80] Davila-Cordova E, Salas-Huetos A, Valle-Hita C, Fernández de la Puente M, Martínez M, Palau-Galindo A, et al. Healthy and unhealthy dietary patterns and sperm quality from the Led-Fertyl study. Andrology. 2024.

[81] Gu X, Bui LP, Wang F, Wang DD, Springmann M, Willett WC. Global adherence to a healthy and sustainable diet and potential reduction in premature death. Proceedings of the National Academy of Sciences of the United States of America. 2024;121:e2319008121.

[82] Venegas Hargous C, Orellana L, Corvalan C, Strugnell C, Allender S, Bell C. Chilean children's adherence to sustainable healthy diets and its associations with sociodemographic and anthropometric factors: a cross-sectional study. European journal of nutrition. 2024;63:2459-75.

[83] Huang S, Hu H, Gong H. Association between the Planetary Health Diet Index and biological aging among the U.S. population. Frontiers in public health. 2024;12:1482959.

[84] Mohammadi F, Alijani S, Abdollahi N, Mashoufi A, Nouri M, Soltanii M, et al. The association between Planetary Health Diet Index and the risk of colorectal cancer: a case-control study. Scientific reports. 2024;14:26546.

[85] Ye B, Xiong Q, Yang J, Huang Z, Huang J, He J, et al. Adoption of region-specific diets in China can help achieve gains in health and environmental sustainability. Nature food. 2024;5:764-74.

[86] Cai YW, Gao JW, Wu MX, Xie YX, You S, Liao GH, et al. Adherence to EAT-Lancet diet, biological aging, and life expectancy in the UK Biobank: a cohort study. The American journal of clinical nutrition. 2025;122:29-38.

[87] Carvalho C, Correia D, Lopes C, Torres D. Adherence to the EAT-Lancet Planetary Health Diet in Portugal and its associations with socioeconomic and lifestyle factors. European journal of nutrition. 2025;64:152.

[88] Damigou E, Downs SM, Chrysohoou C, Barkas F, Tsioufis C, Pitsavos C, et al. Sustainable, planetary healthy dietary patterns are associated with lower 20-year incidence of cardiovascular disease: the ATTICA study (2002-2022). European journal of clinical nutrition. 2025;79:536-43.

[89] Aznar de la Riera MDC, Ortolá R, Fabre-Estremera B, Buño-Soto A, Rodríguez-Artalejo F, Sotos-Prieto M. Association between the Planetary Health Diet Index and growth differentiation factor-15: the Seniors ENRICA-2 cohort. GeroScience. 2025.

[90] Deng K, Shen L, Xue Z, Li BY, Tang J, Zhao H, et al. Association of the EAT-Lancet diet, serial measures of serum proteome and gut microbiome, and cardiometabolic health: a prospective study of Chinese middle-aged and elderly adults. The American journal of clinical nutrition. 2025;121:567-79.

[91] Gong H, Zhang K, Choi S, Huang S. The association between Planetary Health Diet Index (PHDI) and chronic obstructive pulmonary disease (COPD): the mediating role of dietary inflammatory index (DII). BMC pulmonary medicine. 2025;25:53.

[92] Harrison L, Herrmann A, Quitmann C, Stieglbauer G, Zeitz C, Reininghaus U, et al. Effects of a cafeteria-based sustainable diet intervention on wellbeing at a large German hospital: a quasi-experimental study. BMC public health. 2025;25:2047.

[93] Lei L, Qin H, Chen Y, Sun Y, Yin W, Tong S. Association Between Adherence to EAT-Lancet Diet and Risk of Hypertension: An 18-Year National Cohort Study in China. Journal of the American Nutrition Association. 2024:1-10.

[94] Li X, Li J, Ren X, Xia T, Arah OA, Chen L. The associations of long working hours and unhealthy diet with cardiometabolic outcomes and mortality in US workers. Preventive medicine. 2025;195:108275.

[95] Mansouri F, Shateri Z, Shoja M, Jahromi SE, Nouri M, Babajafari S. The association between the planetary health diet index and the risk of sarcopenia and protein-energy wasting in patients with chronic kidney disease. Journal of health, population, and nutrition. 2025;44:153.

[96] Qiu X, Shen S, Jiang N, Lu D, Feng Y, Yang G, et al. Adherence to the planetary health diet index and metabolic dysfunction-associated steatotic liver disease: a cross-sectional study. Frontiers in nutrition. 2025;12:1534604.

[97] Samuelsson J, Stubbendorff A, Marseglia A, Lindberg O, Dartora C, Shams S, et al. A comparative study of the EAT-Lancet diet and the Mediterranean diet in relation to neuroimaging biomarkers and cognitive performance. Alzheimer's & dementia : the journal of the Alzheimer's Association. 2025;21:e70191.

[98] Tang H, Zhang X, Luo N, Huang J, Yang Q, Lin H, et al. Temporal trends in the planetary health diet index and its association with cardiovascular, kidney, and metabolic diseases: A comprehensive analysis from global and individual perspectives. The journal of nutrition, health & aging. 2025;29:100520.

[99] Vargas-Quesada R, Monge-Rojas R, Romero-Zúñiga JJ, Araya-Bastias C, Kovalskys I, Herrera-Cuenca M, et al. Adherence to the EAT-Lancet Diet is Not Associated With Weight Status in a Latin American Urban Multicentric Study. 2025;2025:9615321.

[100] Wei Q, Yang M, Zhu Z, Xiang L, Wang Y, Peng L, et al. Adherence to the EAT-Lancet diet reduces the risk of renal cancer: Results from a population-based prospective study. Public health. 2025;244:105739.

[101] Ye X, Lou C, Shen Z. Plasma Metabolite Profiles Linked to EAT-Lancet Diet and Risk of Inflammatory Bowel Disease: A Prospective Cohort Study From UK Biobank. Molecular nutrition & food research. 2025;69:e70059.

[102] Zhang Z, Huang Z, Xi Y, Han S, Ye X, Zhu H, et al. Association between adherence to the EAT-Lancet diet and risk of microvascular complications in type 2 diabetes: A cohort study. Diabetes, obesity & metabolism. 2025;27:3858-68.

[103] Cacau LT, De Carli E, de Carvalho AM, Lotufo PA, Moreno LA, Bensenor IM, et al. Development and Validation of an Index Based on EAT-Lancet Recommendations: The Planetary Health Diet Index. Nutrients. 2021;13.

[104] Kesse-Guyot E, Rebouillat P, Brunin J, Langevin B, Allès B, Touvier M, et al. Environmental and nutritional analysis of the EAT-Lancet diet at the individual level: insights from the NutriNet-Sante study. Journal of Cleaner Production. 2021;296.

[105] Laine JE, Huybrechts I, Gunter MJ, Ferrari P, Weiderpass E, Tsilidis K, et al. Co-benefits from sustainable dietary shifts for population and environmental health: an assessment from a large European cohort study. The Lancet Planetary health. 2021;5:e786-e96.

[106] Tepper S, Kissinger M, Avital K, Shahar DR. The Environmental Footprint Associated With the Mediterranean Diet, EAT-Lancet Diet, and the Sustainable Healthy Diet Index: A Population-Based Study. Frontiers in nutrition. 2022;9:870883.

[107] Mangone L, Sacerdote C, Laine J, Masala G, Bendinelli B, Panico S, et al. Food, Health, and Mitigation of Climate change in Italy. Epidemiologia e prevenzione. 2023;47:32-8.

[108] Frank SM, Jaacks LM, Meyer K, Rose D, Adair LS, Avery CL, et al. Dietary quality and dietary greenhouse gas emissions in the USA: a comparison of the planetary health diet index, healthy eating index-2015, and dietary approaches to stop hypertension. The international journal of behavioral nutrition and physical activity. 2024;21:36.

[109] Kenđel Jovanović G, Krešić G, Dujmić E, Pavičić Žeželj S. Adherence to the Planetary Health Diet and Its Association with Diet Quality and Environmental Outcomes in Croatian University Students: A Cross-Sectional Study. Nutrients. 2025;17.

[110] Batlle-Bayer L, Aldaco R, Bala A, Puig R, Laso J, Margallo M, et al. Environmental and nutritional impacts of dietary changes in Spain during the COVID-19 lockdown. The Science of the total environment. 2020;748:141410.

[111] Bozeman JF, Springfield S, Theis TL. Meeting EAT-Lancet Food Consumption, Nutritional, and Environmental Health Standards: A U.S. Case Study across Racial and Ethnic Subgroups. Environmental justice (Print). 2020;13:160-72.

[112] Forber KJ, Rothwell SA, Metson GS, Jarvie HP, Withers PJA. Plant-based diets add to the wastewater phosphorus burden. Environmental Research Letters. 2020;15.

[113] Prag AA, Henriksen CB. Transition from Animal-Based to Plant-Based Food Production to Reduce Greenhouse Gas Emissions from Agriculture-The Case of Denmark. Sustainability. 2020;12.

[114] Semba RD, de Pee S, Kim B, McKenzie S, Nachman K, Bloem MW. Adoption of the 'planetary health diet' has different impacts on countries' greenhouse gas emissions. Nature food. 2020;1:481-4.

[115] de Pee S, Hardinsyah R, Jalal F, Kim BF, Semba RD, Deptford A, et al. Balancing a sustained pursuit of nutrition, health, affordability and climate goals: exploring the case of Indonesia. The American journal of clinical nutrition. 2021;114:1686-97.

[116] Kassem R, Jepsen MR, Salhofer SP. The water consumption of different diets in Denmark. Journal of Cleaner Production. 2021;286.

[117] Kidd B, Mackay S, Vandevijvere S, Swinburn B. Cost and greenhouse gas emissions of current, healthy, flexitarian and vegan diets in Aotearoa (New Zealand). BMJ nutrition, prevention & health. 2021;4:275-84.

[118] Philippidis G, Ferrer-Pérez H, Gracia-de-Rentería P, M'Barek R, López AIS. Eating your greens: a global sustainability assessment. Resources Conservation and Recycling. 2021;168.

[119] Vanham D, Guenther S, Ros-Baró M, Bach-Faig A. Which diet has the lower water footprint in Mediterranean countries? Resources, conservation, and recycling. 2021;171:105631.

[120] Vitale M, Giosuè A, Vaccaro O, Riccardi G. Recent Trends in Dietary Habits of the Italian Population: Potential Impact on Health and the Environment. Nutrients. 2021;13.

[121] Cambeses-Franco C, Feijoo G, Moreira MT, González-García S. Co-benefits of the EAT-Lancet diet for environmental protection in the framework of the Spanish dietary pattern. The Science of the total environment. 2022;836:155683.

[122] Cleghorn C, Nghiem N, Mhurchu CN. Assessing the Health and Environmental Benefits of a New Zealand Diet Optimised for Health and Climate Protection. Sustainability. 2022;14.

[123] Dhar AR, Oita A, Matsubae K. Food Nitrogen Footprint of the Indian Subcontinent Toward 2050. Frontiers in nutrition. 2022;9:899431.

[124] Jha CK, Singh V, Stevanovic M, Dietrich JP, Mosnier A, Weindl I, et al. The role of food and land use systems in achieving India's sustainability targets. Environmental Research Letters. 2022;17.

[125] Read QD, Hondula KL, Muth MK. Biodiversity effects of food system sustainability actions from farm to fork. Proceedings of the National Academy of Sciences of the United States of America. 2022;119:e2113884119.

[126] Sun Z, Scherer L, Tukker A, Spawn-Lee SA, Bruckner M, Gibbs HK, et al. Dietary change in high-income nations alone can lead to substantial double climate dividend. Nature food. 2022;3:29-37.

[127] Sun Z, Scherer L, Zhang Q, Behrens P. Adoption of plant-based diets across Europe can improve food resilience against the Russia-Ukraine conflict. Nature food. 2022;3:905-10.

[128] Taherzadeh O, Kanemoto K. Differentiated responsibilities of US citizens in the country's sustainable dietary transition. Environmental Research Letters. 2022;17.

[129] Tucci M, Martini D, Marino M, Del Bo C, Vinelli V, Biscotti P, et al. The Environmental Impact of an Italian-Mediterranean Dietary Pattern Based on the EAT-Lancet Reference Diet (EAT-IT). Foods (Basel, Switzerland). 2022;11.

[130] Tuninetti M, Ridolfi L, Laio F. Compliance with EAT-Lancet dietary guidelines would reduce global water footprint but increase it for 40% of the world population. Nature food. 2022;3:143-51.

[131] Bellamy AS, Furness E, Mills S, Clear A, Finnigan SM, Meador E, et al. Promoting dietary changes for achieving health and sustainability targets. Frontiers in Sustainable Food Systems. 2023;7.

[132] Clay N, Charlton K, Stefoska-Needham A, Heffernan E, Hassan HIC, Jiang XT, et al. What is the climate footprint of therapeutic diets for people with chronic kidney disease? Results from an Australian analysis. Journal of Human Nutrition and Dietetics. 2023.

[133] Gatto A, Kuiper M, van Meijl H. Economic, social and environmental spillovers decrease the benefits of a global dietary shift. Nature food. 2023;4:496-507.

[134] Geibel I, Freund F. The effects of dietary changes in Europe on greenhouse gas emissions and agricultural incomes in Ireland and Denmark. Environmental Research Letters. 2023;18.

[135] Kopainsky B, Kapmeier F. Dynamic implications of the biological link between bovine milk and meat production for operationalizing the planetary health diet. Nature food. 2023;4:1070-4.

[136] Navarre N, Schrama M, de Vos C, Mogollón JM. Interventions for sourcing EAT<i>-Lancet</i> diets within national agricultural areas: A global analysis. One earth (Cambridge, Mass). 2023;6:31-40.

[137] Neff RA, Ramsing RJ, Kim BF. Commercial weight-loss diets, greenhouse gas emissions and freshwater consumption. Journal of human nutrition and dietetics : the official journal of the British Dietetic Association. 2023;36:2268-79.

[138] Schön AM, Böhringer M. Land Consumption for Current Diets Compared with That for the Planetary Health Diet-How Many People Can Our Land Feed? Sustainability. 2023;15.

[139] Stewart K, Balmford A, Scheelbeek P, Doherty A, Garnett EE. Changes in greenhouse gas emissions from food supply in the United Kingdom. Journal of Cleaner Production. 2023;410.

[140] Stone TF, Thompson JR, Rosentrater KA, Liebman M. Modeling a localized metropolitan food system in the Midwest USA: Life cycle impacts of scenarios for Des Moines, Iowa. The Science of the total environment. 2023;865:161095.

[141] Eustachio Colombo P, Elinder LS, Nykänen EA, Patterson E, Lindroos AK, Parlesak A. Developing a novel optimisation approach for keeping heterogeneous diets healthy and within planetary boundaries for climate change. European journal of clinical nutrition. 2024;78:193-201.

[142] Bakman T, Hoffmann BS, Portugal-Pereira J. A recipe for change: Analyzing the climate and ecosystem impacts of the Brazilian diet shift. The Science of the total environment. 2024;930:172568.

[143] Bertoldo J, Fammartino A, Egan S, Neff RA, Grekin R, Wolfson JA. Evaluating Food Procurement against the EAT-Lancet Planetary Health Diet in a Sample of U.S. Universities. International journal of environmental research and public health. 2024;21.

[144] Caldeira TCM, Vandevijvere S, Swinburn B, Mackay S, Claro RM. Differences in the cost and environmental impact between the current diet in Brazil and healthy and sustainable diets: a modeling study. Nutrition journal. 2024;23:71.

[145] Conti A, Opizzi A, Binala JG, Cortese L, Barone-Adesi F, Panella M. Evaluation of the Climate Impact and Nutritional Quality of Menus in an Italian Long-Term Care Facility. Nutrients. 2024;16.

[146] DeCesaro JM, Allison EH, Clawson G, Frazier M, Gephart JA, Hicks CC, et al. The distribution of environmental pressures from global dietary shift. Environmental Research Letters. 2024;19.

[147] Lengle JM, Michaelsen Bjøntegaard M, Hauger Carlsen M, Jafarzadeh S, Frost Andersen L. Environmental impact of Norwegian self-selected diets: comparing current intake with national dietary guidelines and EAT-Lancet targets. Public health nutrition. 2024;27:e100.

[148] Eberle U, Mumm N. Reduction potential of German environmental food impacts due to a planetary health diet. International Journal of Life Cycle Assessment. 2024;29:1727-37.

[149] Kesse-Guyot E, Berthy F, Berlivet J, Perraud E, Touvier M, Hercberg S, et al. Alignment between greenhouse gas emissions reduction and adherence the EAT-Lancet diet: A modeling study based on the NutriNet-Santé cohort. The Science of the total environment. 2024;951:175470.

[150] Li YX, He P, Shan YL, Li Y, Hang Y, Shao S, et al. Reducing climate change impacts from the global food system through diet shifts. Nature Climate Change. 2024.

[151] Li M, Wang YN, Zhao SN, Chen W, Liu Y, Zheng HR, et al. Improving the affordability and reducing greenhouse gas emissions of the EAT- Lancet diet in China. Sustainable Production and Consumption. 2024;52:445-57.

[152] Liu MH, Fang CL, Liao X, Bai Y, Wu QY, Liu ZT, et al. Multiple environmental and nutritional effects of changing food consumption in urban and rural China. Environmental Impact Assessment Review. 2024;107.

[153] Payró C, Taherzadeh O, van Oorschot M, Koch J, Marselis S. Consumer resistance diminishes environmental gains of dietary change. Environmental Research Letters. 2024;19.

[154] Rulli MC, Sardo M, Ricciardi L, Govoni C, Galli N, Chiarelli DD, et al. Meeting the EAT-Lancet 'healthy' diet target while protecting land and water resources. Nature Sustainability. 2024;7.

[155] Unar-Munguía M, Cervantes-Armenta MA, Rodríguez-Ramírez S, Bonvecchio Arenas A, Fernández Gaxiola AC, Rivera JA. Mexican national dietary guidelines promote less costly and environmentally sustainable diets. Nature food. 2024;5:703-13.

[156] Çelik ZM, Barcın-Güzeldere HK, Ede-Çintesun E, Bayram HM. From plate to planet: nutritional and environmental sustainability of Turkish cuisine across the regions of Türkiye. International journal of environmental health research. 2025;35:1972-87.

[157] Chiriacò MV, Galli N, Latella M, Rulli MC. Pressure on Global Forests: Implications of Rising Vegetable Oils Consumption Under the EAT-Lancet Diet. Global change biology. 2025;31:e70077.

[158] Conrad Z, Thorne-Lyman AL, Wu S, DiStaso C, Korol M, Love DC. Are healthier diets more sustainable? A cross-sectional assessment of 8 diet quality indexes and 7 sustainability metrics. The American journal of clinical nutrition. 2025;121:315-23.

[159] Crosnier A, Baudry G, Jeangros L, Thalmann P. Environmental impact assessment of nutritional guidelines under organic agriculture in Switzerland. 2025;Volume 9 - 2025.

[160] de Lange T, van Dijk M, Kuiper M, van Zeist WJ, Bartelings H, Mizan A, et al. Socio-economic, environmental and health impacts of dietary transformation in Bangladesh. Environmental Research Letters. 2025;20.

[161] Deng Z, Hu Y, Wang X, Li C, Wang J, He P, et al. Transitioning to healthy and sustainable diets has higher environmental and affordability trade-offs for emerging and developing economies. Nature communications. 2025;16:3948.

[162] Herforth AW, Bai Y, Venkat A, Masters WA. The Healthy Diet Basket is a valid global standard that highlights lack of access to healthy and sustainable diets. Nature food. 2025;6:622-31.

[163] Michailidis V, Lugato E, Panagos P, Grados D, Freund F, Jones A, et al. How do diet shifts affect the greenhouse gas balance of agricultural soils? Denmark as a case study. Agricultural Systems. 2025;224.

[164] Tuyishimire A, Yin J, Lin J, Liu M, Tao J, Muhirwa F. Can adopting the EAT-Lancet diet bring synergistic benefits in nutrition and sustainability? Evidence from African countries. Resources, Conservation and Recycling. 2025;215:108173.

[165] Yang W. Dynamic Trends in Aquatic Product Supply and Consumption in China: Implications for Sustainable Diets and Environmental Impact Reduction. Foods (Basel, Switzerland). 2025;14.

[166] Lassen AD, Christensen LM, Trolle E. Development of a Danish Adapted Healthy Plant-Based Diet Based on the EAT-Lancet Reference Diet. Nutrients. 2020;12.

[167] Hanley-Cook GT, Argaw AA, de Kok BP, Vanslambrouck KW, Toe LC, Kolsteren PW, et al. EAT-Lancet diet score requires minimum intake values to predict higher micronutrient adequacy of diets in rural women of reproductive age from five low- and middle-income countries. The British journal of nutrition. 2021;126:92-100.

[168] Tucci M, Martini D, Del Bo C, Marino M, Battezzati A, Bertoli S, et al. An Italian-Mediterranean Dietary Pattern Developed Based on the EAT-Lancet Reference Diet (EAT-IT): A Nutritional Evaluation. Foods (Basel, Switzerland). 2021;10.

[169] Hendrie GA, Rebuli MA, James-Martin G, Baird DL, Bogard JR, Lawrence AS, et al. Towards healthier and more sustainable diets in the Australian context: comparison of current diets with the Australian Dietary Guidelines and the EAT-Lancet Planetary Health Diet. BMC public health. 2022;22:1939.

[170] Beal T, Ortenzi F, Fanzo J. Estimated micronutrient shortfalls of the EAT-Lancet planetary health diet. The Lancet Planetary health. 2023;7:e233-e7.

[171] Berthy F, Brunin J, Allès B, Reuzé A, Touvier M, Hercberg S, et al. Higher adherence to the EAT-Lancet reference diet is associated with higher nutrient adequacy in the NutriNet-Santé cohort: a cross-sectional study. The American journal of clinical nutrition. 2023;117:1174-85.

[172] Cacau LT, Hanley-Cook GT, Huybrechts I, De Henauw S, Kersting M, Gonzalez-Gross M, et al. Relative validity of the Planetary Health Diet Index by comparison with usual nutrient intakes, plasma food consumption biomarkers, and adherence to the Mediterranean diet among European adolescents: the HELENA study. European journal of nutrition. 2023;62:2527-39.

[173] Venegas Hargous C, Orellana L, Strugnell C, Corvalan C, Allender S, Bell C. Adapting the Planetary Health Diet Index for children and adolescents. The international journal of behavioral nutrition and physical activity. 2023;20:146.

[174] Macit-Çelebi MS, Bozkurt O, Kocaadam-Bozkurt B, Köksal E. Evaluation of sustainable and healthy eating behaviors and adherence to the planetary health diet index in Turkish adults: a cross-sectional study. Frontiers in nutrition. 2023;10:1180880.

[175] Wright EC, van Oort B, Bjøntegaard MM, Carlsen MH, Andersen LF. Environmental and nutritional assessment of young children's diets in Norway: comparing the current diet with national dietary guidelines and the EAT-Lancet reference diet. European journal of nutrition. 2023;62:3383-96.

[176] Armes S, Bhanjdeo A, Chakraborty D, Kaur H, Ray S, Rao N. Aligning Santal Tribe Menu Templates with EAT-Lancet Commission's Dietary Guidelines for Sustainable and Healthy Diets: A Comparative Analysis. Nutrients. 2024;16.

[177] Gaona-Pineda EB, López-Olmedo N, Moreno-Macías H, Shamah-Levy T. Three approaches to assessing dietary quality in Mexican adolescents from 2006 to 2018 with data from national health and nutrition surveys. Public health nutrition. 2024;27:e97.

[178] Mortaş H, Navruz-Varlı S, Bilici S. Adherence to the Planetary Health Diet and Its Association with Diet Quality in the Young Adult Population of Türkiye: A Large Cross-Sectional Study. Nutrients. 2024;16.

[179] Nicol K, Nugent AP, Woodside JV, Hart KH, Bath SC. Iodine and plant-based diets: a narrative review and calculation of iodine content. The British journal of nutrition. 2024;131:265-75.

[180] Guzmán-Castellanos KB, Neri SS, García IZ, Hernández-Hernández A, Valdés-Mas M, Bes-Rastrollo M, et al. Planetary health diet, mediterranean diet and micronutrient intake adequacy in the Seguimiento Universidad de Navarra (SUN) cohort. European journal of nutrition. 2025;64:149.

[181] Kersting M, Kalhoff H, Zahn K, Belgardt A, Cacau LT, Moreno LA, et al. How to improve sustainability of nutrient dense diets for children and adolescents: an exemplary assessment in Germany. European journal of nutrition. 2024;64:11.

[182] Lei L, Qin H, Chen Y, Sun Y, Yin W, Tong S. Association Between Adherence to EAT-Lancet Diet and Risk of Hypertension: An 18-Year National Cohort Study in China. Journal of the American Nutrition Association. 2025;44:40-9.

[183] Miranda AR, Vieux F, Maillot M, Verger EO. How Do the Indices based on the EAT-Lancet Recommendations Measure Adherence to Healthy and Sustainable Diets? A Comparison of Measurement Performance in Adults from a French National Survey. Current developments in nutrition. 2025;9:104565.

[184] Vargas-Quesada R, Monge-Rojas R, Romero-Zúñiga JJ, Arriola Aguirre R, Kovalskys I, Herrera-Cuenca M, et al. Adherence to the EAT-Lancet diet and its association with micronutrient intake in the urban population of eight Latin American countries. Nutrition research (New York, NY). 2025;139:136-48.

[185] Hirvonen K, Bai Y, Headey D, Masters WA. Affordability of the EAT-Lancet reference diet: a global analysis. Lancet Glob Health. 2020;8:e59-e66.

[186] Batis C, Marrón-Ponce JA, Stern D, Vandevijvere S, Barquera S, Rivera JA. Adoption of healthy and sustainable diets in Mexico does not imply higher expenditure on food. Nature food. 2021;2:792-801.

[187] Gupta S, Vemireddy V, Singh DK, Pingali P. Ground truthing the cost of achieving the EAT lancet recommended diets: Evidence from rural India. Global food security. 2021;28:100498.

[188] Headey DD, Ecker O, Comstock AR, Ruel MT. Poverty, price and preference barriers to improving diets in sub-Saharan Africa. Global food security. 2023;36:100664.

[189] Aburto TC, Salgado JC, Rodríguez-Ramírez S, Rivera JA, Barquera S, Batis C. Adherence to the EAT-Lancet index is associated with lower diet costs in the Mexican population. Nutrition journal. 2024;23:108.

[190] Chen KZ, Yu LY, Lin W, Ortega DL. What does it take to revolutionize Chinese diets: evidence from a choice experiment on urban Chinese consumers. China Agricultural Economic Review. 2024;16:850-70.

[191] Cohen DA, Puttock E, Montes M, Lopez K, Labisi T, Voorhees A, et al. An Affordable and Sustainable Thrifty-Like Meal Plan, FoodRx, That Meets the Recommended Dietary Allowances. Journal of urban health : bulletin of the New York Academy of Medicine. 2024;101:364-70.

[192] Headey D, Hirvonen K, Alderman H. Estimating the cost and affordability of healthy diets: How much do methods matter? Food policy. 2024;126:102654.

[193] Caldeira TCM, Nassif L, Sousa TM, Maia EG, Fagioli HB, Canella DS, et al. Cost Associated with Adherence to the EAT-Lancet Score in Brazil. Nutrients. 2025;17.

[194] Dehnavi MK, Abbasi H, Hajian PN, Motlagh AD, Azadbakht L. Adherence to the planetary health diet reduces dietary costs by 21% supporting affordable healthy eating among older adults in Iran. Scientific reports. 2025;15:9586.

[195] Morales-Ríos JE, Unar-Munguía M, Batis C, Quiroz-Reyes JA, Sánchez-Ortiz NA, Colchero MA. Simulating price subsidies on healthy foods in Mexico. Public health nutrition. 2025;28:e74.

[196] Rochefort G, Paquette MC, Robitaille J, Lemieux S, Provencher V, Lamarche B. Increased costs associated with greater adherence to the EAT-Lancet Commission reference diet in the province of Québec: the PREDISE Study. The British journal of nutrition. 2025;133:837-44.

[197] Willett W, Rockström J, Loken B, Springmann M, Lang T, Vermeulen S, et al. Food in the Anthropocene: the EAT-Lancet Commission on healthy diets from sustainable food systems. Lancet. 2019;393:447-92.

[198] Athare TR, Pradhan P, Kropp JP. Environmental implications and socioeconomic characterisation of Indian diets. The Science of the total environment. 2020;737:139881.

[199] Poole MK, Musicus AA, Kenney EL. Alignment Of US School Lunches With The EAT-Lancet Healthy Reference Diet's Standards For Planetary Health. Health affairs (Project Hope). 2020;39:2144-52.

[200] Sharma M, Kishore A, Roy D, Joshi K. A comparison of the Indian diet with the EAT-Lancet reference diet. BMC public health. 2020;20:812.

[201] Springmann M, Spajic L, Clark MA, Poore J, Herforth A, Webb P, et al. The healthiness and sustainability of national and global food based dietary guidelines: modelling study. BMJ (Clinical research ed). 2020;370:m2322.

[202] Castellanos-Gutiérrez A, Sánchez-Pimienta TG, Batis C, Willett W, Rivera JA. Toward a healthy and sustainable diet in Mexico: where are we and how can we move forward? The American journal of clinical nutrition. 2021;113:1177-84.

[203] Eustachio Colombo P, Elinder LS, Lindroos AK, Parlesak A. Designing Nutritionally Adequate and Climate-Friendly Diets for Omnivorous, Pescatarian, Vegetarian and Vegan Adolescents in Sweden Using Linear Optimization. Nutrients. 2021;13.

[204] Kretschmer S, Dehm S. Sustainability Transitions in University Food Service-A Living Lab Approach of Locavore Meal Planning and Procurement. Sustainability. 2021;13.

[205] Llanaj E, Hanley-Cook GT. Adherence to healthy and sustainable diets is not differentiated by cost, but rather source of foods among young adults in Albania. The British journal of nutrition. 2021;126:591-9.

[206] Llanaj E, Vincze F, Kósa Z, Bárdos H, Diószegi J, Sándor J, et al. Deteriorated Dietary Patterns with Regards to Health and Environmental Sustainability among Hungarian Roma Are Not Differentiated from Those of the General Population. Nutrients. 2021;13.

[207] Tepper S, Geva D, Shahar DR, Shepon A, Mendelsohn O, Golan M, et al. The SHED Index: a tool for assessing a Sustainable HEalthy Diet. European journal of nutrition. 2021;60:3897-909.

[208] Ali Z, Scheelbeek PFD, Felix J, Jallow B, Palazzo A, Segnon AC, et al. Adherence to EAT-Lancet dietary recommendations for health and sustainability in the Gambia. Environmental research letters : ERL [Web site]. 2022;17:104043.

[209] Bäck S, Skaffari E, Vepsäläinen H, Lehto R, Lehto E, Nissinen K, et al. Sustainability analysis of Finnish pre-schoolers' diet based on targets of the EAT-Lancet reference diet. European journal of nutrition. 2022;61:717-28.

[210] Ibarrola-Rivas MJ, Unar-Munguia M, Kastner T, Nonhebel S. Does Mexico have the agricultural land resources to feed its population with a healthy and sustainable diet. Sustainable Production and Consumption. 2022;34:371-84.

[211] Mapes BR, Prager SD, Béné C, Gonzalez CE. Healthy and sustainable diets from today to 2050-The role of international trade. PloS one. 2022;17:e0264729.

[212] Young HA. Adherence to the EAT-Lancet Diet: Unintended Consequences for the Brain? Nutrients. 2022;14.

[213] Campirano F, López-Olmedo N, Ramírez-Palacios P, Salmerón J. Sustainable Dietary Score: Methodology for Its Assessment in Mexico Based on EAT-Lancet Recommendations. Nutrients. 2023;15.

[214] Delgermaa D, Yamaguchi M, Nomura M, Nishi N. Assessment of Mongolian dietary intake for planetary and human health. PLOS global public health. 2023;3:e0001229.

[215] Ganpule A, Dubey M, Pandey H, Green R, Brown KA, Srinivasapura Venkateshmurthy N, et al. Dietary patterns in North and South India: a comparison with EAT-Lancet dietary recommendations. Journal of human nutrition and dietetics : the official journal of the British Dietetic Association. 2023;36:2170-9.

[216] Ludwig-Borycz E, Neumark-Sztainer D, Larson N, Baylin A, Jones AD, Webster A, et al. Personal, behavioural and socio-environmental correlates of emerging adults' sustainable food consumption in a cross-sectional analysis. Public health nutrition. 2023;26:1306-16.

[217] Nomura M, Yamaguchi M, Inada Y, Nishi N. Current dietary intake of the Japanese population in reference to the planetary health diet-preliminary assessment. Frontiers in nutrition. 2023;10:1116105.

[218] Abe-Inge V, Kwofie EM, Kubow S, Orsat V, Ulimwengu J. Uncovering the gap: Assessing the compliance of the canadian food availability with dietary recommendations and its impact on the environment. Global Food Security-Agriculture Policy Economics and Environment. 2024;40.

[219] Beckmann A, Strassner C, Kwanbunjan K. Thailand - how far are we from achieving a healthy and sustainable diet? A longitudinal ecological study. The Lancet regional health Southeast Asia. 2024;29:100478.

[220] Bennett G, Gibney ER. An investigation of diet quality across racial groups in the United Kingdom and United States considering nutritional adequacy, disease risk, and environmental sustainability: a secondary analysis of NDNS and NHANES datasets. Journal of nutritional science. 2024;13:e93.

[221] Hoteit M, Khattar M, Malli D, Antar E, Al Hassani Z, Abdallah M, et al. Dietary Intake among Lebanese Adults: Findings from the Updated LEBANese natiONal Food Consumption Survey (LEBANON-FCS). Nutrients. 2024;16.

[222] Navruz-Varlı S, Mortaş H, Çelik MN. Sociodemographic Trends in Planetary Health Diets among Nutrition Students in Türkiye: Bridging Classroom to Kitchen. Nutrients. 2024;16.

[223] Richter A, Loss J, Kuhn DA, Moosburger R, Mensink GBM. Evaluating the diet in Germany with two indices focusing on healthy eating and planetary healthy eating using nationwide cross-sectional food intake data from DEGS1 (2008-2011). European journal of nutrition. 2024;63:2943-56.

[224] Crosnier A, Baudry G, Jeangros L, Thalmann P. Environmental impact assessment of nutritional guidelines under organic agriculture in Switzerland. Frontiers in Sustainable Food Systems. 2025;9.

[225] Fischer I, Narayan KM, Siegel KR. Food Supply And Dietary Guidelines Mismatch: Policy Recommendations To Improve Human And Planetary Health. Health affairs (Project Hope). 2025;44:467-74.

[226] Hunziker C, Arrieta EM, Diaz MP, León AE. A Comparative Study Between the Dietary Pattern in Argentina and International Recommendations. Foods (Basel, Switzerland). 2025;14.

[227] Samarathunga N, Spence A, Grimes C, Russell CG, Lacy KE. Comparing Australian children's dietary intakes with the Eat-Lancet Commission Planetary Health Diet targets and Australian Dietary Guidelines: a cross-sectional study. European journal of nutrition. 2024;64:56.
